# Sustainable Synthesis of Polyfluoro-Pyrimido [1,2-*a*] Benzimidazole Derivatives Using a Tandem Strategy—Ultrasound and an Integrated Continuous Flow System

**DOI:** 10.1021/acs.joc.4c03123

**Published:** 2025-03-07

**Authors:** Vijay Thavasianandam Seenivasan, Nian-Qi Chen, Karthick Govindan, Alageswaran Jayaram, Yu-Chen Lin, Chien-Hung Li, Wei-Yu Lin

**Affiliations:** † Department of Medicinal and Applied Chemistry, Kaohsiung Medical University, Kaohsiung 80708, Taiwan ROC; ‡ Department of Medical Research, Kaohsiung Medical University Hospital, Kaohsiung 80708, Taiwan ROC; § Drug Development and Value Creation Research Centre, Kaohsiung Medical University, Kaohsiung 80708, Taiwan ROC

## Abstract

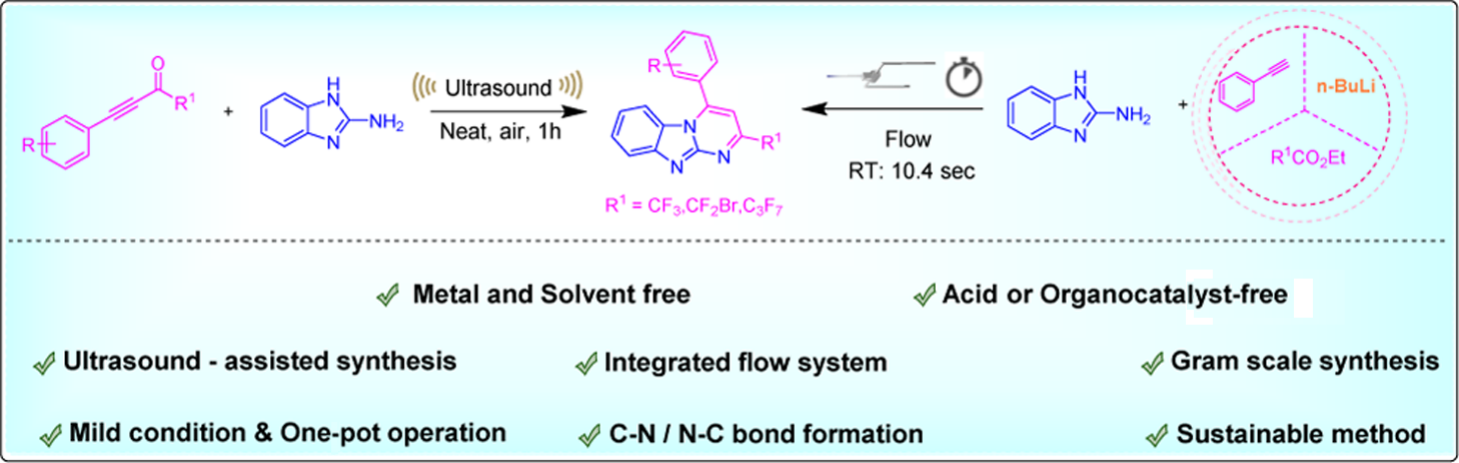

We have developed a novel ultrasound technique that generates significant amounts of CF_3_-substituted benzo[4,5] imidazo [1,2-*a*]pyrimidine analogues from easily accessible starting materials in an ecologically friendly and efficient approach. This method is notably helpful for producing physiologically relevant compounds containing the imidazopyrimidine unit, which serves as a versatile building block for the synthesis of *N*-fused heterocycles and is devoid of metals, solvents, additives, and catalysts. Additionally, utilizing ultrasound in an open-air environment, a range of polyfluoro-ynones were successfully reacted with 2-aminobenzimidazole, generating a diverse array of polyfluoroimidazo[1,2-*a*]pyrimidine derivatives. Furthermore, by employing an integrated flow system approach, we were able to synthesize polyfluoro-substituted benzo[4,5]imidazo[1,2-*a*]pyrimidine derivatives from alkynes with a much shorter reaction time. Gram-scale synthesis proved this method’s scalability and highlighted its potential for synthetic and industrial applications. The straightforward nature of the process, broad compatibility with various functional groups, and substantial sustainability advantages collectively underscore its significance.

## Introduction

**Figure 1:**
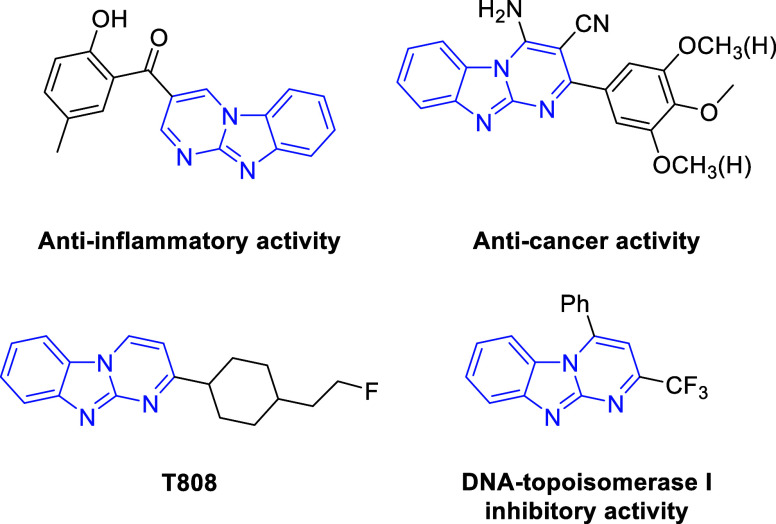
Biologically active molecules with a core of the pyrimido[1,2-*a*]imidazole unit.

**Scheme 1:**
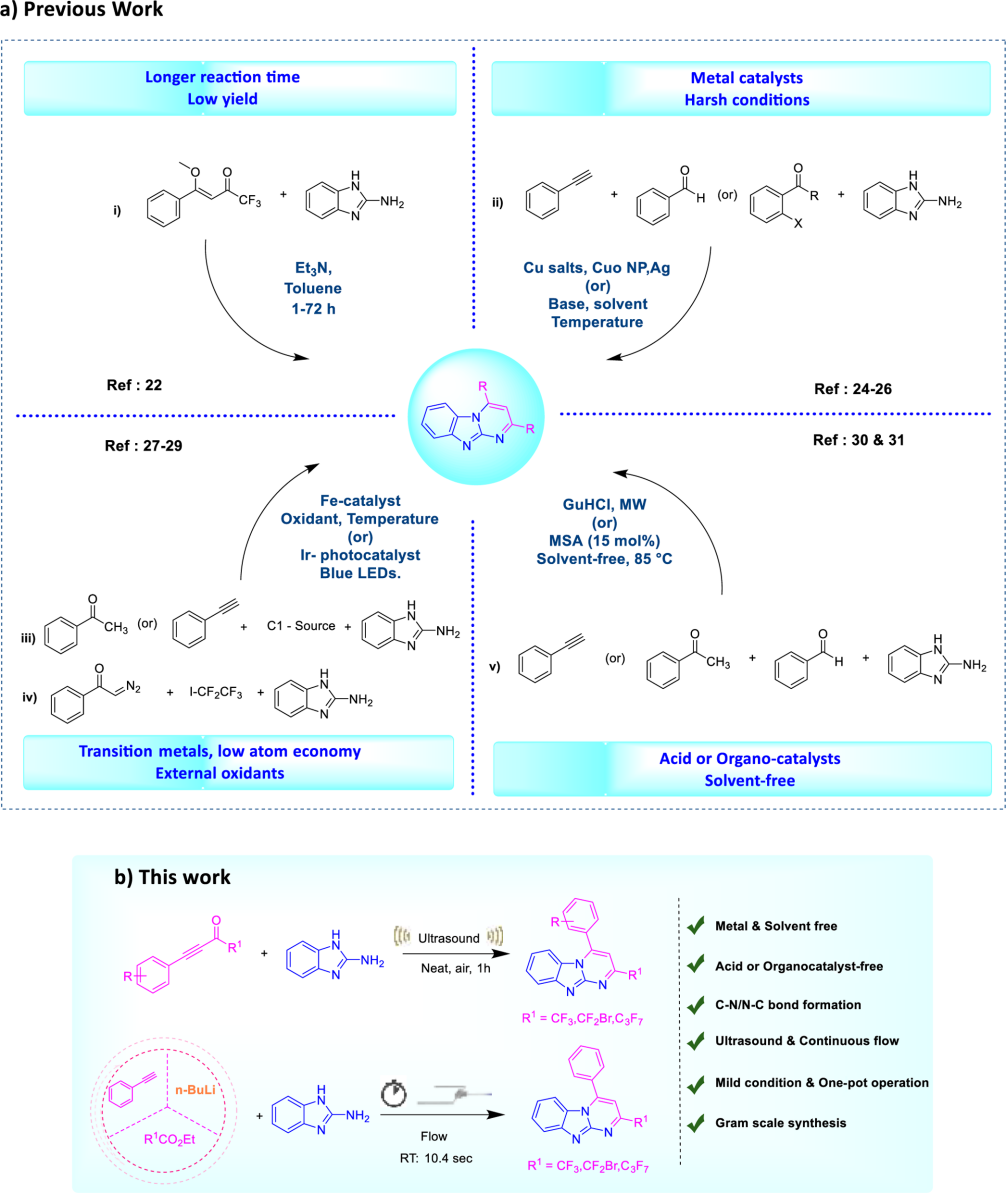
(a) Previous Methods for the Synthesis of the pyrimido[1,2-*a*] Benzimidazole Unit. (b) This Work

Numerous fields of chemistry have demonstrated a great deal of interest in *N*-fused imidazo heterocyclic molecules.^1−4^ In order to improve the possibility of developing novel lead compounds, the insertion of a trifluoromethyl (CF_3_) group plays a critical role in enhancing the parent molecule’s permeability, lipophilicity, and metabolic stability.^5−8^ According to recent estimates, at least one fluorine atom can be found in approximately 20% of prescribed and clinically authorized medications. Depending on the sales period, 30–50% of the most popular drugs additionally consist of fluorine.^9^ CF_3_-substituted *N*-fused heterocyclic compounds have been widely used in biochemistry, agrochemistry, materials science, and medicine in recent years.^10−16^ Because of their remarkable biological effects, benzo[4,5]imidazo[1,2-*a*]pyrimidine derivatives have garnered special attention among them.^17−19^ For example, they showed excellent anti-inflammatory activity against TNF-α and IL-6,^12^ anticancer properties^20^ (T808 is a particular PET tracer for imaging of tau pathologies),^21^ and significant DNA-topoisomerase I inhibitory activity.^22^ (Figure 1). These scaffolds have important biological implications; nevertheless, they also serve as organic fluorophores with excellent optical characteristics.^23^ The demand for efficient and valuable synthetic techniques to create natural and biomimetic pyrimido[1,2-*a*]benzimidazole units is rising due to the significance of these molecules. Zanatta et al. synthesized 2-CF_3_-pyrimido[1,2-*a*] benzimidazoles in 2016 through a cyclo-condensation process involving 2-aminobenzimidazole and 4-alkoxyvinyl trifluoromethyl ketones [Scheme 1a(i)].^22^ The synthesis of pyrimido[1,2-*a*] benzimidazole units in the past decade has primarily relied on coupling reactions involving 2-aminobenzimidazole, aldehydes, and alkynes, with functionalized pyrimido[1,2-*a*]benzimidazole units being the result of these processes, which are carried via regioselective catalyzed by transition metals (Cu/Ag/CuO NP) [Scheme 1a(ii)].^24−26^ Highly 6-endo-dig cyclization, which involves intramolecular N–H bond activation and C–N formation. Nevertheless, the demand for high temperatures, long reaction times, transition metals, and low yields frequently places limitations on these methods. Yuan et al. recently described a reaction that uses DMF as the carbon source, with 2-aminobenzimidazole and acetophenone as substrates in the presence of an iron-catalyzed [3 + 2 + 1] intermolecular cycloaddition [Scheme 1a(iii)].^27^ Employing the same techniques, Ma et al. reported *N*,*N*-dimethyl aminoethanol as the carbon synthon under an iron-catalyst with TfOH [Scheme 1a(iii)].^28^ The same group developed a photochemical formal [3 + 2 + 1] annulation strategy using α-diazoketones as denitrogenated synthons under an Ir-photocatalyst [Scheme 1a(iv)].^29^ Despite these developments, there still exist concerns to be resolved, such as the requirement for catalysts containing transition metals, external oxidants, and the usage of solvents and hazardous substances. For instance, the Jeong group devised a one-pot multicomponent system that is catalyzed by molybdate sulfuric acid (MSA) and effectively produced three new bonds (two C–N and one C–C) in a single operation, leading to the rapid production of pyrimido[1,2-*a*]benzimidazole units [Scheme 1a(iv)].^30^ Through an identical methodology, the Anupam Jana group reported a solvent-free, substoichiometric synthesis of pyrimido[1,2-*a*]benzimidazole units, mediated by guanidine hydrochloride with microwave-assisted synthesis of pyrimido[1,2-*a*]benzimidazole units starting from easily accessible aryl aldehydes, aryl methyl ketones, and benzimidazole [Scheme 1a(v)].^31^ However, these processes still require acid or organocatalyst to achieve the desired outcomes. Therefore, to overcome the abovementioned problems with complex catalytic systems, high temperatures, prolonged reaction times, and solvents, in recent years, techniques like sonochemistry,^32−35^ mechanochemistry, microwave synthesis, and continuous-flow chemistry have been more concentrated on developing a methodology that helps to increase the overall sustainability, lower the *E*-factor, and improve the Eco-Scale score.^31,36,37^ The pharmaceutical industry has recently shown an intense interest in continuous-flow technologies due to their advantages over traditional batch methods. Despite recent advances in continuous-flow chemistry, our group has made significant progress in this area.^38−42^ These techniques offer a strong platform for chemical innovation because they provide several benefits, such as minimizing reaction volumes, improving mass and heat transfer, in situ operation, speeding up reactions, and being easily scalable. All these advantages are beneficial for sustainable chemical processes,^43−45^ hence our long-standing interest in developing sustainable routes to CF_3_–*N*-heterocyclic compounds in an ultrasound-assisted and continuous-flow process.

We now report a catalyst- and solvent-free, sonochemistry-assisted, atom-economical synthetic route for CF_3_-substituted benzo[4,5]imidazo[1,2-*a*]pyrimidine using CF_3_-ynones and 2-aminobenzimidazole, and expansion of the established continuous-flow methodology to the integrated flow system was also examined to achieve the desired product from phenylacetylene as a simple precursor by producing an unstable intermediate, to the synthesis of polyfluoro-*N*-fused heterocyclic derivatives (Scheme 1b). Moreover, these benefits include overall sustainability, scalability, and precise reaction conditions, which minimize the need for numerous laboratory operations and reduce waste chemical generation, time, and cost by subsequently transforming two or more reactions in a one-pot operation. Interestingly, this cascade reaction produces the required CF_3_-substituted benzo[4,5]imidazo[1,2-*a*]pyrimidine by initially undergoing condensation following intramolecular 6-endo-dig cyclization. This process facilitates the creation of C–N/N–C bonds; the possible mechanism is illustrated in Scheme S11 (Supporting Information). This protocol has excellent tolerance for different functional groups, a high Eco Scale score, and a mild, straightforward approach in ultrasound-assisted and integrated flow systems.

## Results and Discussion

**Scheme 2:**
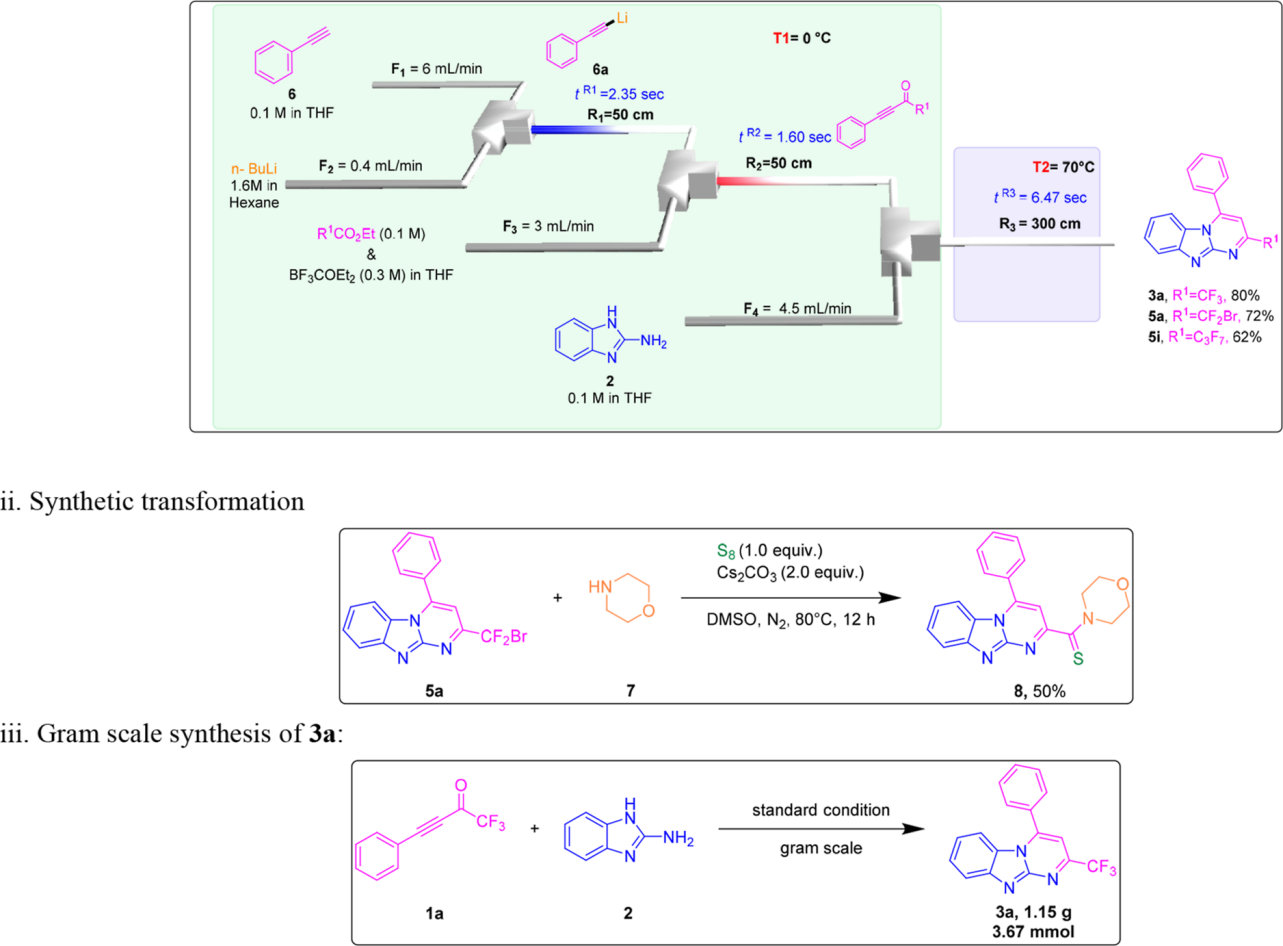
Integrated Flow System, Synthetic Transformation, and Gram Scale Synthesis of **3a**

To verify our hypothesis, we initially conducted an intramolecular 6-endo-dig cyclization between CF_3_-ynone **1a** and 2-aminobenzimidazole **2** under ultrasound irradiation. Surprisingly, we obtained our expected 4-phenyl-2-(trifluoromethyl) imidazo [1,2-*a*]pyrimidine **3a** in 30 min, with a 78% yield (Table 1, entry 1) and single-crystal XRD study confirmed the structure of **3a**. The equivalent of **1a** and the ultrasonic irradiation time were examined (Table 1, entries 2 and 3). The reaction yield was enhanced by raising the equivalent of **1a**, according to the result (entry 3). To clarify the importance of ultrasonic irradiation, a lower yield was obtained by shortening the reaction time (Table 1, entry 4). When the reaction was assisted by mechanical stirring without the use of a solvent, an 82% yield was obtained. However, it was noted that a sticky and thick mass was formed during the process (Table 1, entry 5). To overcome this issue, the reaction used 1,4-dioxane as a solvent medium, resulting in an extension of the reaction time by 180 min (Table 1, entry 6). Thereafter, an investigation was undertaken to assess the impact of the 1,4-dioxane as a solvent on ultrasonic irradiation, leading to the successful attainment of a 78% yield of **3a** in 15 min (Table 1, entry 7). Upon extending the irradiation duration to 60 min, the desired product **3a** was obtained in a 94% yield (Table 1, entry 8). The optimization results reveal the crucial role of ultrasound in boosting the reaction and ensuring a homogeneous reaction mixture. During the reaction, cold water was added to the ultrasonic bath to prevent temperature increase and maintain the reaction at room temperature. The established optimized reaction conditions, as delineated in entry 3 of Table 1, have been ascertained to represent the optimal parameters for achieving the ideal reaction conditions. The substrate scope was examined under ideal conditions to show off this method’s versatility, and the outcomes are summarized in Table 2. Several CF_3_-ynones **1a**–**1f** with electron-donating and electron-withdrawing groups at the para position of the benzene ring exhibited remarkable reactivity in the standard reaction. The corresponding CF_3_-substituted benzo[4,5]imidazo[1,2-*a*]pyrimidines **3a**–**3f** were successfully transformed in good to excellent yields (85–95%). Particularly, the sterically hindered substrate **1g** (C2 position) worked well and afforded the desired product **3g** in a 75% yield. Gratifyingly, different substituents on the phenyl ring may utilize this approach. The findings established that disubstituted derivatives **1h** and **1i** successfully produced the desired compounds **3h** and **3i** with high efficiency, achieving good yields of 75% and 85%, respectively. Subsequently, the positions of the substitutions on the phenyl ring of the ynone derivatives **1j**–**1l**, specifically at the C3 and C4 positions (CN and OH groups), were tested, resulting in **3j**–**3l** in yields ranging from 60 to 84%. Notably, the reaction with 4-phenyl substituted CF_3_-ynone **1m** proceeded, affording the targeted product **3m** in a 55% yield, likely due to the solid nature of **1m**. To boost the yield of **3m**, the reaction was conducted using 1,4-dioxane as the solvent (liquid-assisted ultrasonic irradiation), significantly increasing the yield to an impressive 92%. It is noteworthy that heterocyclic derivatives, such as thiophene, was tolerated well in this reaction since **3n** formed effectively with a moderate yield of 68%. Furthermore, we applied this approach for the 4-styryl-incorporated ynone, which was transformed to the appropriate product **3o** at a lower yield of 28% with less conversion observed. The formation of a cyclized compound **3p** and **3q** was not observed; those may be due to less electrophilicity of phenyl- or methyl-substituted ynones instead of CF_3_-ynones. Unfortunately, the CF_3_-ynone **1a** did not react with benzo[*d*]thiazol-2-amine to attain the desired product **3r**. Having successful results, we expanded our strategy to employ CF_2_Br-ynone **4a** and 2-aminobenzimidazole **2** via an ultrasound-assisted process to synthesize the targeted cyclized derivatives **5a** at a 75% yield, as shown in Table 3. We conducted an investigation of the tolerance of the technique by examining derivatives of CF_2_Br-ynones that included different functional groups (4-Me, 4-OMe, 4-Cl, and 4-Br) associated with their phenyl ring **4b**–**4e**. Fortunately, we produced the appropriate products **5b**–**5e** in good to excellent yields ranging from 82 to 90%. The synthesis of the cyclized product **5f** with an outstanding yield of 89% was reported when the disubstituted substrates worked smoothly. In addition, the substituent containing a thiophene ring provides promising results of **5g** in a 78% yield. Unfortunately, the 4-styryl incorporated product **5h** was unsuitable for this transformation due to the unreaction of **4h**. Also, perfluoroalkyl-substituted ynone **4i** afforded the corresponding product **5i** in a moderate yield of 70%. After successfully demonstrating the versatility of our substrate range, we evaluated the practicality of our protocol by attempting to scale up the synthesis of product **3a** without compromising the yield of 92% (Scheme 2,iii). Furthermore, we focused on the continuous flow approach and proved how effectively it worked for a one-pot synthesis in an environmentally benign manner and with enhanced selectivity. To do this, the flow setup used a T-shaped micromixer (M1, Φ = 500 μm) and one tubing reactor (R_1_, Φ = 800 μm); the rate of the reactants, reaction temperature, and reactor volumes were all altered in a second optimization (Table S1, see the Supporting Information). Following an extensive investigation of many factors, the ideal conditions were ultimately determined to be a flow rate of 20 μL min^–1^ in EtOH and a residence time of 25.1 min at 70 °C, yielding an 81–90% isolated yield **3a**, **3c**, and **3f** (Scheme S8, see the Supporting Information). After successfully establishing the continuous flow approach, our attention shifts to an integrated flow system to synthesize lithium acetylide intermediate **6a** in situ from phenylacetylene **6**.^35,40^ This intermediate is subsequently combined with R^1^CO_2_Et to produce polyfluoro-containing ynones **1a**, **4a**, and **4i**. The related R^1^-ynones were reacted with 2-aminobenzimidazole **2** under mild conditions and a much shorter reaction time of ∼11 s to synthesize **3a**, **5a**, and **5i** with moderate to good yields (Scheme S9; for more details, see the Supporting Information). Furthermore, we explored the derivatization of the product of **5a** and were able to achieve methanethione incorporating *N*-heterocycle **8** at a yield of 50% (Scheme 2ii).^46,47^

**Table 1:**
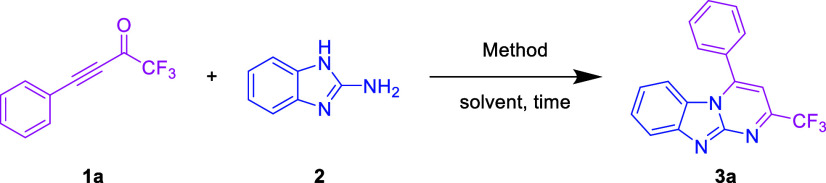
Optimal Conditions^a^^,^^b^

entry	1a (equiv)	solvent	method	time (min)	yield 3a (%)^b^
1	1.0	neat	ultrasound	30	78
2	1.0	neat	ultrasound	60	90
3	1.2	neat	ultrasound	60	95
4	1.2	neat	ultrasound	30	83
5	1.2	neat	batch	60	82
6	1.2	1,4- dioxane	batch	180	96
7	1.2	1,4- dioxane	ultrasound	15	78
8	1.2	1,4- dioxane	ultrasound	60	94

a The reactions were performed on a 0.20 mmol scale of compound 2.

b Isolated yield.

**Table 2:**
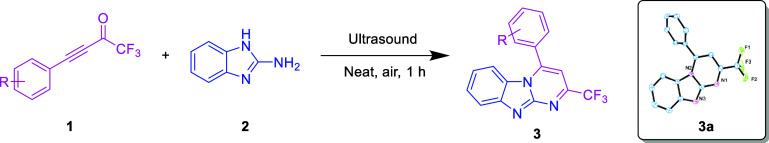

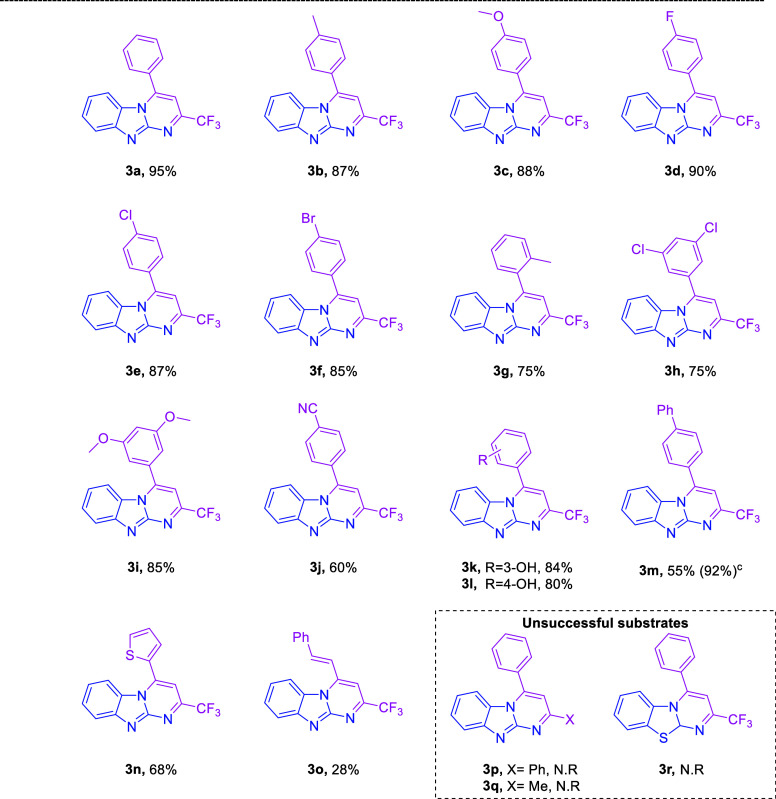
Reaction Scope of CF_3_-Ynones^a^

a The reactions were performed with **1** (0.24 mmol, 1.2 equiv) and **2** (0.20 mmol, 1.0 equiv) in neat, open air under ultrasound for 1 h.

b Isolated yield.

c 1,4-dioxane as the solvent for 1 h.

**Table 3:**
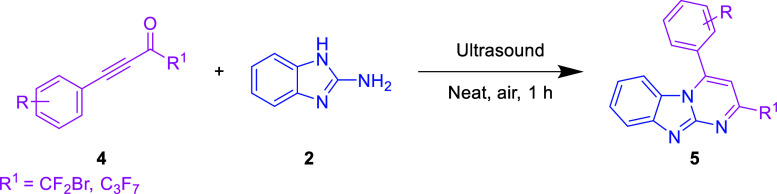

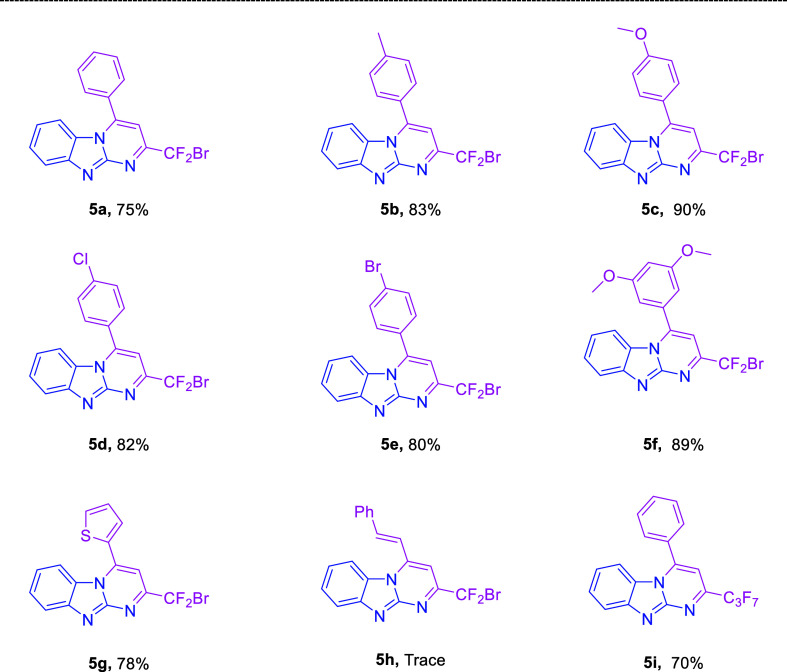
Reaction Scope of CF_2_Br-Ynones^a^,^b^

a The reactions were performed with **4** (0.24 mmol, 1.2 equiv) and **2** (0.20 mmol, 1.0 equiv) in neat, open air under ultrasound for 1 h.

b Isolated yield.

## Conclusions

In conclusion, employing easily available CF_3_-ynones and 2-amino benzimidazole via sonochemistry, we have established a sustainable, solvent- and metal-free, atom-economical synthetic method for the efficient synthesis of CF_3_-substituted benzo[4,5]imidazo[1,2-*a*]pyrimidine derivatives. This approach, which produces compounds with high functional group tolerance, utilizes a cascade reaction of condensation, followed by intramolecular cyclization. It is distinguished by its simpler reaction conditions and environmentally friendly approach. The procedure is further improved by using a continuous-flow technique, which shows its potential for commercial use by allowing the production of typical CF_3_–*N*-fused heterocycles in 25.1 min. In addition, in situ generation of perfluoro contains ynones and their subsequent coupling with 2-aminobenzimidazole to obtain the desired cyclized product with high yields in a much shorter reaction time, under the transition metal-free conditions. Furthermore, the resultant CF_2_Br-pyrimido[1,2-*a*] benzimidazole units serve as valuable building blocks for drug development. All things considered, this strategy offers a significant contrast to current techniques, aligning with sustainable manufacturing methods and adhering to green chemistry principles.

## Experimental Section

The experimental details are presented in the Supporting Information.

### Characterization Data for Compounds **3a–3n**, **5a–5i**, and **8**

#### 4-Phenyl-2-(trifluoromethyl)benzo[4,5]imidazo[1,2-*a*]pyrimidine **(3a)**

The title compound was synthesized according to the general procedure, purified by column chromatography (Hexane/EtOAc = 8/2), and obtained as a yellow solid (59.5 mg, 95%); mp 191–193 °C; ^1^H NMR (400 Hz, CDCl_3_): δ 8.03 (d, *J* = 8.0 Hz, 1H), 7.76–7.64 (m, 5H), 7.56–7.52 (m, 1H), 7.16–7.12 (m, 1H), 7.10 (s, 1H), 6.79 (d, *J* = 8.0 Hz, 1H); ^13^C{^1^H}-NMR (100 MHz, CDCl_3_): δ 152.0 (q, *J* = 36.0 Hz), 152.0, 149.9, 145.8, 131.9, 131.6, 129.8, 128.2, 127.2, 122.8, 120.5 (q, *J* = 275.0 Hz), 119.2, 115.2, 103.6 (d, *J* = 2.0 Hz). ^19^F{^1^H}-NMR (376 MHz, CDCl_3_): δ −68.75 (s, 3F). HRMS (HR-ESI) *m*/*z*: [M]+ calcd for C_17_H_10_F_3_N_3_ 313.0827; found, 313.0828.

#### 4-(*p*-Tolyl)-2-(trifluoromethyl)benzo[4,5]imidazo[1,2-*a*]pyrimidine **(3b)**

The title compound was synthesized according to the general procedure, purified by column chromatography (Hexane/EtOAc = 8/2), and obtained as a yellow solid (56.9 mg, 87%); mp 175–177 °C; ^1^H NMR (400 Hz, CDCl_3_): δ 8.04 (d, *J* = 8.0 Hz, 1H), 7.56–7.47 (m, 5H), 7.17–7.13 (m, 1H), 7.05 (s, 1H), 6.89 (d, *J* = 12.0 Hz, 1H), 2.57 (s, 3H); ^13^C{^1^H}-NMR (100 MHz, CDCl_3_): δ 152.4, 152.0 (q, *J* = 37.0 Hz), 149.9, 145.5, 142.5, 130.4, 128.7, 128.1, 127.2, 122.7, 121.1, 120.5 (q, *J* = 274.0 Hz), 115.3, 103.7 (d, *J* = 2.0 Hz), 21.8; ^19^F{^1^H}-NMR (376 MHz, CDCl_3_): δ −68.65 (s, 3F). HRMS (HR-ESI) *m*/*z*: [M + H]^+^ calcd for C_18_H_13_F_3_N_3_ 328.1061; found, 328.1050.

#### 4-(4-Methoxyphenyl)-2-(trifluoromethyl)benzo[4,5]imidazo[1,2-*a*]pyrimidine **(3c)**

The title compound was synthesized according to the general procedure, purified by column chromatography (Hexane/EtOAc = 8/2), and obtained as a yellow solid (60.4 mg, 88%); mp. 168–170 °C; ^1^H NMR (400 Hz, CDCl_3_): δ 8.06 (d, *J* = 8.0 Hz, 1H), 7.63–7.61 (m, 2H), 7.59–7.55 (m, 1H), 7.21–7.17 (m, 3H), 7.11 (s, 1H), 7.00 (d, *J* = 12.0 Hz, 1H), 3.98 (s, 3H); ^13^C{^1^H}-NMR (100 MHz, CDCl_3_): δ 162.2, 152.8, 152.7 (q, *J* = 38.0 Hz), 149.1, 143.0, 130.2, 127.9, 126.9, 123.2, 121.6 (q, *J* = 275.0 Hz),120.2, 115.5, 115.2, 105.1, 55.8; ^19^F{^1^H}- NMR (376 MHz, CDCl_3_): δ −68.75 (s, 3F). HRMS (HR-ESI) *m*/*z*: [M + H]^+^ calcd for C_18_H_13_F_3_N_3_O 344.1012; found, 344.1002.

#### 4-(4-Fluorophenyl)-2-(trifluoromethyl)benzo[4,5]imidazo[1,2-*a*]pyrimidine **(3d)**

The title compound was synthesized according to the general procedure, purified by column chromatography (Hexane/EtOAc = 8/2), and obtained as a yellow solid (59.6 mg, 90%); mp. 160–162 °C; ^1^H NMR (400 Hz, CDCl_3_): δ 8.03 (d, *J* = 8.0 Hz, 1H), 7.73–7.69 (m, 2H), 7.59–7.55 (m, 2H), 7.43–7.39 (m, 2H), 7.22–7.18 (m, 1H), 7.11 (s, 1H), 6.83 (d, *J* = 8.0 Hz, 1H); ^13^C{^1^H}-NMR (100 MHz, CDCl_3_): δ 166.1, 163.5, 152.1 (q, *J* = 37.0 Hz), 149.1, 143.7,, 130.8 (d, *J* = 8.0 Hz),127.8127.4 (d, *J* = 4.0 Hz), 126.9, 117.2, 123.4, 120.3 (q, *J* = 275.0 Hz), 117.4(d, *J* = 22.0 Hz),115.2104.9; ^19^F{^1^H}- NMR (376 MHz, CDCl_3_): δ −68.75 (s, 3F), −106.37 (s, 1F); HRMS (HR-ESI) *m*/*z*: [M + H]^+^ calcd for C_17_H_10_F_4_N_3_ 332.0811; found, 332.0811.

#### 4-(4-Chlorophenyl)-2-(trifluoromethyl)benzo[4,5]imidazo[1,2-*a*]pyrimidine **(3e)**

The title compound was synthesized according to the general procedure, purified by column chromatography (Hexane/EtOAc = 8/2), and obtained as a yellow solid (60.5 mg, 89%); mp. 210–212 °C; ^1^H NMR (400 Hz, CDCl_3_): δ 8.04 (d, *J* = 8.0 Hz, 1H), 7.71–7.65 (m, 4H), 7.59–7.55 (m, 1H), 7.24–7.19 (m, 1H), 7.11 (s, 1H), 6.88 (d, *J* = 8.0 Hz, 1H); ^13^C{^1^H}-NMR (100 MHz, CDCl_3_): δ 152.5 (d, *J* = 37.0 Hz), 151.2149.1, 143.8, 138.6, 130.3, 129.8, 129.6, 127.9, 126.8, 123.5, 121.2(q, *J* = 274.0 Hz), 115.2104.7; ^19^F{^1^H}-NMR (376 MHz, CDCl_3_): δ −68.75 (s, 3F); HRMS (HR-ESI) *m*/*z*: [M + H]^+^ calcd for C_17_H_10_ClF_3_N_3_ 348.0515; found, 348.0500.

#### 4-(4-Bromophenyl)-2-(trifluoromethyl)benzo[4,5]imidazo[1,2-*a*]pyrimidine **(3f)**

The title compound was synthesized according to the general procedure, purified by column chromatography (Hexane/EtOAc = 8/2), and obtained as a yellow solid (66.5 mg, 85%); mp. 205–207 °C; ^1^H NMR (400 Hz, CDCl_3_): δ 8.02 (d, *J* = 8.0 Hz, 1H), 7.76–7.61 (m, 4H), 7.54 (dd, *J* = 16.0 Hz, 8.0 Hz, 1H), 7.20–7.15 (m, 1H), 7.05 (d, *J* = 8.0 Hz, 1H), 6.82 (dd, *J* = 32.0 Hz, 8.0 Hz, 1H); ^13^C{^1^H}-NMR (100 MHz, CDCl_3_): δ 151.9 (q, *J* = 37.0 Hz), 150.7, 149.8, 145.6, 138.4, 131.9, 129.8, 128.2, 127.3, 122.8, 121.08, 120.5 (q, *J* = 275.0 Hz), 115.0, 103.7 (d, *J* = 9.0 Hz); ^19^F{^1^H}- NMR (376 MHz, CDCl_3_): δ −68.74 (s, 3F); HRMS (HR-ESI) *m*/*z*: [M + H]^+^ calcd for C_17_H_10_BrF_3_N_3_ 392.0010; found, 392.0008.

#### 4-(*o*-Tolyl)-2-(trifluoromethyl)benzo[4,5]imidazo[1,2-*a*]pyrimidine**(3g)**

The title compound was synthesized according to the general procedure, purified by column chromatography (Hexane/EtOAc = 8/2), and obtained as a yellow solid (49.1 mg, 75%); mp. 170–172 °C; ^1^H NMR (400 Hz, CDCl_3_): δ 8.11 (d, *J* = 8.0 Hz, 1H), 7.83–7.80 (m, 1H), 7.72–7.65 (m, 3H), 7.59–7.57 (m, 1H), 7.31–7.72 (m, 1H), 7.26 (s, 1H), 6.62 (d, *J* = 8.0 Hz, 1H), 2.22 (s, 3H); ^13^C{^1^H}-NMR (100 MHz, CDCl_3_): δ 152.29 (q, *J* = 37.0 Hz), 149.32,145.04, 136.53, 131.87, 131.25, 131.08, 128.64, 127.48, 127.38, 123.48, 120.46 (q, *J* = 275.0 Hz), 120.87, 116.34, 114.31, 103.57, 19.22; ^19^F{^1^H}- NMR (376 MHz, CDCl_3_): δ −68.61 (s, 3F).

#### 4-(3,5-Dichlorophenyl)-2-(trifluoromethyl)benzo[4,5]imidazo[1,2-*a*]pyrimidine **(3h)**

The title compound was synthesized according to the general procedure, purified by column chromatography (Hexane/EtOAc = 8/2), and obtained as a yellow solid (57.2 mg, 75%); mp. 262–264 °C; ^1^H NMR (400 Hz, CDCl_3_): δ 8.05 (d, *J* = 8.0 Hz, 1H), 7.74–7.73 (m, 1H), 7.60–7.57 (m, 1H), 7.55 (d, *J* = 4.0 Hz, 2H), 7.26–7.22 (m, 1H), 7.05 (s, 1H), 6.86 (d, *J* = 8.0 Hz, 1H); ^13^C{^1^H}-NMR (100 MHz, CDCl_3_): δ 151.7 (q, *J* = 37.0 Hz), 149.4, 148.5, 145.9, 136.9, 134.0, 132.0, 127.5, 126.8, 123.5, 121.7, 120.3 (q, *J* = 275.0 Hz), 114.6, 103.7 (d, *J* = 2.0 Hz); ^19^F{^1^H}-NMR (376 MHz, CDCl_3_): δ −68.74 (s, 3F); HRMS (HR-ESI) *m*/*z*: [M + H]^+^ calcd for C_17_H_9_Cl_2_F_3_N_3_ 382.0125; found, 382.0116.

#### 4-(3,5-Dimethoxyphenyl)-2-(trifluoromethyl)benzo[4,5]imidazo[1,2-*a*]pyrimidine **(3i)**

The title compound was synthesized according to the general procedure, purified by column chromatography (Hexane/EtOAc = 8/2), and obtained as a yellow solid (63.5 mg, 85%); mp. 185–187 °C; ^1^H NMR (400 Hz, CDCl_3_): δ 8.03 (d, *J* = 8.0 Hz, 1H), 7.57–7.53 (m, 1H), 7.21–7.17 (m, 1H), 7.07 (s, 1H), 6.95 (d, *J* = 8.0 Hz, 1H), 6.78–6.77 (m, 1H), 6.71 (m, 2H), 3.85 (s, 6H); ^13^C{^1^H}-NMR (100 MHz, CDCl_3_): δ 161.9, 151.7, 151.2 (q, *J* = 37.0 Hz), 149.8, 145.8, 133.1, 127.2, 122.9, 121.1, 120.5 (q, *J* = 275.0 Hz), 115.4, 106.0, 103.6, 103.2 (d, *J* = 2.0 Hz), 55.9; ^19^F{^1^H}- NMR (376 MHz, CDCl_3_): δ −68.74 (s, 3F); HRMS (HR-ESI) *m*/*z*: [M + H]^+^ calcd for C_19_H_15_F_3_N_3_O_2_ 374.1116; found, 374.1107.

#### 4-(2-(Trifluoromethyl)benzo[4,5]imidazo[1,2-*a*]pyrimidin-4-yl)benzonitrile **(3j)**

The title compound was synthesized according to the general procedure, purified by column chromatography (Hexane/EtOAc = 8/2), and obtained as a yellow solid (40.6 mg, 60%); mp. 214–216 °C; ^1^H NMR (400 Hz, CDCl_3_): δ 8.06 (d, *J* = 8.0 Hz, 1H), 8.02 (d, *J* = 8.0 Hz, 2H), 7.83 (d, *J* = 8.0 Hz, 2H), 7.60–7.56 (m, 1H), 7.23–7.19 (m, 1H), 7.06 (s, 1H), 6.73 (d, *J* = 8.0 Hz, 1H); ^13^C{^1^H}-NMR (100 MHz, CDCl_3_): δ 151.8 (q, *J* = 37.0 Hz), 149.4, 145.7, 135.6, 133.6, 129.4, 127.6, 123.5, 121.7, 120.3 (q, *J* = 275.0 Hz), 117.5, 116.1, 114.5, 103.7 (d, *J* = 2.0 Hz); ^19^F{^1^H}-NMR (376 MHz, CDCl_3_): δ −68.75 (s, 3F); HRMS (HR-ESI) *m*/*z*: [M + H]^+^ calcd for C_18_H_10_F_3_N_4_ 339.0857; found, 339.0847.

#### 3-(2-(Trifluoromethyl)benzo[4,5]imidazo[1,2-*a*]pyrimidin-4-yl)phenol **(3k)**

The title compound was synthesized according to the general procedure, purified by column chromatography (Hexane/EtOAc = 8/2), and obtained as a yellow solid (55.3 mg, 84%); mp. 350–352 °C; ^1^H NMR (400 Hz, DMSO-*d*_6_): δ 10.06 (s, 1H), 7.97 (d, *J* = 8.0 Hz, 1H), 7.59–7.50 (m, 2H), 7.46 (s, 1H), 7.25–7.12 (m, 4H), 6.75 (d, *J* = 4.0 Hz, 1H); ^13^C{^1^H}-NMR (100 MHz, DMSO-*d*_6_): δ 158.0, 152.6, 150.5 (q, *J* = 36.0 Hz), 149.9, 145.1, 132.5, 130.7, 126.9, 122.1, 120.6 (q, *J* = 275.0 Hz), 120.0, 118.3, 115.2, 114.9, 103.1 (d, *J* = 2.0 Hz); ^19^F{^1^H}-NMR (376 MHz, DMSO-*d*_6_): δ −67.40 (s, 3F); HRMS (HR-ESI) *m*/*z*: [M + H]^+^ calcd for C_17_H_11_F_3_N_3_O 330.0854; found, 330.0846.

#### 4-(2-(Trifluoromethyl)benzo[4,5]imidazo[1,2-*a*]pyrimidin-4-yl)phenol **(3l)**

The title compound was synthesized according to the general procedure, purified by column chromatography (Hexane/EtOAc = 8/2), and obtained as a yellow solid (52.7 mg, 80%); mp. 365–367 °C; ^1^H NMR (400 Hz, DMSO-*d*_6_): δ 10.32 (s, 1H), 7.96 (d, *J* = 12.0 Hz, 1H), 7.63 (d, *J* = 4.0 Hz, 2H), 7.59–7.55 (m, 1H), 7.39 (s, 1H), 7.26–7.22 (m, 1H), 7.06 (d, *J* = 12.0 Hz, 2H), 6.92 (d, *J* = 12.0 Hz, 1H); ^13^C{^1^H}-NMR (100 MHz, DMSO-*d*_6_): δ 160.7, 153.8, 151.0, 150.4 (q, *J* = 51.0 Hz), 130.6, 127.6, 127.2, 122.4, 121.3, 121.1 (q, *J* = 275.0 Hz), 120.5, 116.5, 115.7, 103.9 (d, *J* = 2.0 Hz); ^19^F{^1^H}-NMR (376 MHz, DMSO-*d*_6_): δ −67.44 (s, 3F); HRMS (HR-ESI) *m*/*z*: [M + H]^+^ calcd for C_17_H_11_F_3_N_3_O 330.0854; found, 330.0846.

#### 4-([1,1′-Biphenyl]-4 yl)-2-(trifluoromethyl)benzo[4,5]imidazo[1,2-*a*]pyrimidine **(3m)**

The title compound was synthesized according to the general procedure, purified by column chromatography (Hexane/EtOAc = 8/2), and obtained as a yellow solid (42.8 mg, 55%); mp. 170–172 °C; ^1^H NMR (400 Hz, CDCl_3_): δ 8.05 (d, *J* = 8.0 Hz, 1H), 7.92 (d, *J* = 8.0 Hz, 2H), 7.76–7.72 (m, 4H), 7.57–7.53 (m, 3H), 7.49–7.47 (m, 1H), 7.19–7.14 (m, 1H), 7.10 (s, 1H), 7.00 (d, *J* = 4.0 Hz, 1H); ^13^C{^1^H}-NMR (100 MHz, CDCl_3_): δ 151.9, 151.8 (q, *J* = 37.0 Hz), 150.0, 145.8, 144.8, 139.5, 130.3, 129.4, 129.3, 128.8, 128.3, 127.4, 127.3, 122.9, 121.3, 120.6 (q, *J* = 274.0 Hz), 115.4, 103.6 (d, *J* = 2.0 Hz); ^19^F{^1^H}- NMR (376 MHz, CDCl_3_): δ −68.79 (s, 3F); HRMS (HR-ESI) *m*/*z*: [M + H]^+^ calcd for C_23_H_15_F_3_N_3_ 390.1218; found, 390.1214.

#### 4-(Thiophen-2-yl)-2-(trifluoromethyl)benzo[4,5]imidazo[1,2-*a*]pyrimidine **(3n)**

The title compound was synthesized according to the general procedure, purified by column chromatography (Hexane/EtOAc = 8/2), and obtained as a yellow solid (43.4 mg, 68%); mp. 208–210 °C; ^1^H NMR (400 Hz, CDCl_3_): δ 8.05 (d, *J* = 8.0 Hz, 1H), 7.79 (dd, *J* = 1.2 Hz, 1.2 Hz, 1H), 7.59–7.55 (m, 2H), 7.38 (dd, *J* = 4.0 Hz, 4.0 Hz, 1H), 7.24–7.20 (m, 1H), 7.17 (s, 1H), 7.06 (d, *J* = 8.0 Hz, 1H); ^13^C{^1^H}-NMR (100 MHz, CDCl_3_): δ 151.4 (q, *J* = 37.0 Hz), 149.9, 145.8, 145.3, 130.9, 130.5, 130.4, 128.5, 127.4, 123.0, 121.3, 120.4 (q, *J* = 275.0 Hz), 115.0, 105.2 (d, *J* = 2.0 Hz); ^19^F{^1^H}-NMR (376 MHz, CDCl_3_): δ −68.70 (s, 3F); HRMS (HR-EI) *m*/*z*: [M + H]^+^ calcd for C_15_H_9_F_3_N_3_S 320.0469; found, 320.0461.

#### (*E*)-4-Styryl-2-(trifluoromethyl)benzo[4,5]imidazo[1,2-*a*]pyrimidine **(3o)**

The title compound was synthesized according to the general procedure, purified by column chromatography (Hexane/EtOAc = 8/2), and obtained as a red solid (19 mg, 28%); mp 216–218 °C; ^1^H NMR (400 Hz, CDCl_3_): δ 8.11 (d, *J* = 8.0 Hz, 2H), 7.81 (d, *J* = 16.0 Hz, 1H), 7.74–7.72 (m, 2H), 7.68 (s, 1H), 7.65 (d, *J* = 8.0 Hz, 1H), 7.57–7.47 (m, 4H), 7.31 (s, 1H); ^13^C{^1^H}-NMR (100 MHz, CDCl_3_): δ 150.5, 150.0 (q, *J* = 48.0 Hz), 146.0, 141.5, 134.5, 131.1, 129.6, 128.1, 127.9, 127.3, 123.5, 123.4 (q, *J* = 275.0 Hz), 121.4, 118.2, 115.3, 99.9 (d, *J* = 2.0 Hz); ^19^F{^1^H}- NMR (376 MHz, CDCl_3_): δ −68.66 (s, 3F); HRMS (HR-ESI) *m*/*z*: [M + H]^+^ calcd for C_19_H_13_F_3_N_3_ 340.1061; found, 340.1052.

#### 2-(Bromodifluoromethyl)-4-phenylbenzo[4,5]imidazo[1,2-*a*]pyrimidine **(5a)**

The title compound was synthesized according to the general procedure, purified by column chromatography (Hexane/EtOAc = 8/2), and obtained as a yellow solid (55.95 mg, 75%); mp. 168–170 °C; ^1^H NMR (400 Hz, CDCl_3_): δ 8.02 (d, *J* = 8.0 Hz, 1H), 7.75–7.63 (m, 5H), 7.54–7.50 (m, 1H), 7.14–7.10 (m, 1H), 7.04 (s, 1H), 6.77 (d, *J* = 8.0 Hz, 1H); ^13^C{^1^H}-NMR (100 MHz, CDCl_3_): δ 157.0 (t, *J* = 28.0 Hz), 151.8, 149.7, 145.9, 131.8, 129.8, 128.2, 127.1, 122.7, 121.1, 118.9, 115.8 (t, *J* = 305.0 Hz), 115.1, 102.6 (t, *J* = 3.0 Hz); ^19^F{^1^H}-NMR (376 MHz, CDCl_3_): δ −53.09 (s, 2F); HRMS (HR-ESI) *m*/*z*: [M]^+^ calcd for C_17_H_10_BrF_2_N_3_ 373.0026; found, 373.0016.

#### 2-(Bromodifluoromethyl)-4-(*p*-tolyl)benzo[4,5]imidazo[1,2-*a*]pyrimidine **(5b)**

The title compound was synthesized according to the general procedure, purified by column chromatography (Hexane/EtOAc = 8/2), and obtained as a yellow solid (64.2 mg, 83%); mp 145–147 °C; ^1^H NMR (400 Hz, CDCl_3_): δ 8.01 (d, *J* = 8.0 Hz, 1H), 7.50 (dd, *J* = 16.0 Hz, 12.0 Hz, 5H), 7.14–7.10 (m, 1H), 7.01 (s, 1H), 6.89 (d, *J* = 12.0 Hz, 1H), 2.56 (s, 3H); ^13^C{^1^H}-NMR (100 MHz, CDCl_3_): δ 153.5 (t, *J* = 28.0 Hz), 152.1, 149.8, 146.0, 142.4, 130.4, 128.8, 128.1, 127.0, 122.5, 121.0, 115.9 (t, *J* = 305.0 Hz), 115.2, 102.6 (t, *J* = 2.0 Hz), 21.8; ^19^F{^1^H}- NMR (376 MHz, CDCl_3_): δ −53.06 (s, 2F); HRMS (HR-ESI) *m*/*z*: [M + H]^+^ calcd for C_18_H_13_BrF_2_N_3_ 388.0261; found, 388.0249.

#### 2-(Bromodifluoromethyl)-4-(4-methoxyphenyl)benzo[4,5]imidazo[1,2-*a*]pyrimidine **(5c)**

The title compound was synthesized according to the general procedure, purified by column chromatography (Hexane/EtOAc = 8/2), and obtained as a white solid (72.5 mg, 90%); mp. 160–162 °C; ^1^H NMR (400 Hz, CDCl_3_): δ 8.04 (d, *J* = 8.0 Hz, 1H), 7.60–7.57 (m, 2H), 7.56–7.52 (m, 1H), 7.19–7.7.13 (m, 3H), 7.01 (s, 1H), 6.97 (d, *J* = 8.0 Hz, 1H), 3.98 (s, 3H); ^13^C{^1^H}-NMR (100 MHz, CDCl_3_): δ 161.2, 157.8, 156.8 (t, *J* = 28.0 Hz), 149.8, 145.9, 129.7, 126.8, 123.7, 122.3, 120.9, 115.8 (t, *J* = 304.0 Hz), 115.0, 114.9, 102.5 (t, *J* = 3.0 Hz), 55.6; ^19^F{^1^H}-NMR (376 MHz, CDCl_3_): δ −53.03 (s, 2F); HRMS (HR-EI) *m*/*z*: [M + H]^+^ calcd for C_18_H_13_BrF_2_N_3_O 404.0210; found, 404.0196.

#### 2-(Bromodifluoromethyl)-4-(4-chlorophenyl)benzo[4,5]imidazo[1,2-*a*]pyrimidine **(5d)**

The title compound was synthesized according to the general procedure, purified by column chromatography (Hexane/EtOAc = 8/2), and obtained as a yellow solid (66.7 mg, 82%); mp. 158–160 °C; ^1^H NMR (400 Hz, CDCl_3_): δ 8.03 (d, *J* = 8.0 Hz, 1H), 7.71–7.52 (m, 5H), 7.20–7.15 (m, 1H), 7.02 (s, 1H), 6.85 (d, *J* = 8.0 Hz, 1H); ^13^C{^1^H}-NMR (100 MHz, CDCl_3_): δ 157.0 (t, *J* = 28.0 Hz), 150.5, 149.6, 146.0, 138.8, 130.2, 129.8, 127.3, 122.9, 121.4, 118.7, 115.7 (t, *J* = 305.0 Hz), 114.9, 102.7 (t, *J* = 3.0 Hz); ^19^F{^1^H}-NMR (376 MHz, CDCl_3_): δ −53.21 (s, 2F); HRMS (HR-ESI) *m*/*z*: [M + H]^+^ calcd for C_17_H_10_BrClF_2_N_3_ 407.9714; found, 407.9705.

#### 2-(Bromodifluoromethyl)-4-(4-bromophenyl)benzo[4,5]imidazo[1,2-*a*]pyrimidine **(5e)**

The title compound was synthesized according to the general procedure, purified by column chromatography (Hexane/EtOAc = 8/2), and obtained as a yellow solid (72.1 mg, 80%); mp 170–172 °C; ^1^H NMR (400 Hz, CDCl_3_): δ 8.02 (d, *J* = 8.0 Hz, 1H), 7.74–7.63 (m, 4H), 7.52 (d, *J* = 8.0 Hz, 1H), 7.14–7.10 (m, 1H), 7.04 (s, 1H), 6.77 (d, *J* = 8.0 Hz, 1H); ^13^C{^1^H}-NMR (100 MHz, CDCl_3_): δ 157.0 (t, *J* = 28.0 Hz), 150.5, 149.6, 146.0, 138.3, 130.2, 130.0, 129.8, 127.3, 122.9, 121.4, 115.7 (t, *J* = 305.0 Hz), 114.9, 102.7 (t, *J* = 3.0 Hz); ^19^F{^1^H}-NMR (376 MHz, CDCl_3_): δ −53.11 (s, 2F); HRMS (HR-ESI) *m*/*z*: [M + Na]^+^ calcd for C_17_H_9_Br_2_ClF_2_N_3_Na 473.9029; found, 473.1137.

#### 2-(Bromodifluoromethyl)-4-(3,5-dimethoxyphenyl)benzo[4,5]imidazo[1,2-*a*]pyrimidine **(5f)**

The title compound was synthesized according to the general procedure, purified by column chromatography (Hexane/EtOAc = 8/2), and obtained as a yellow solid (77.2 mg, 89%); mp. 170–172 °C; ^1^H NMR (400 Hz, CDCl_3_): δ 8.03 (d, *J* = 12.0 Hz, 1H), 7.57–7.53 (m, 1H), 7.21–7.17 (m, 1H), 7.07 (s, 1H), 6.96–6.94 (m, 1H), 6.77 (m, 1H), 6.71 (m, 2H), 3.85 (s, 6H); ^13^C{^1^H}-NMR (100 MHz, CDCl_3_): δ 161.9, 157.5 (t, *J* = 28.0 Hz), 151.9, 148.9, 144.4, 132.9, 127.6, 126.9, 123.15, 117.1, (t, *J* = 305.0 Hz), 112.5, 106.0, 103.7, 103.0, 55.9; ^19^F{^1^H}-NMR (376 MHz, CDCl_3_): δ −53.09 (s, 2F); HRMS (HR-EI) *m*/*z*: [M + H]^+^ calcd for C_19_H_15_BrF_2_N_3_O_2_ 434.0315; found, 434.0306.

#### 2-(Bromodifluoromethyl)-4-(thiophen-2-yl)benzo[4,5]imidazo[1,2-*a*]pyrimidine **(5g)**

The title compound was synthesized according to the general procedure, purified by column chromatography (Hexane/EtOAc = 8/2), and obtained as a yellow solid (59.1 mg, 78%); mp. 228–230 °C; ^1^H NMR (400 Hz, CDCl_3_): δ 8.03 (d, *J* = 8.0 Hz, 1H), 7.78 (d, *J* = 4.0 Hz, 1H), 7.58–7.53 (m, 2H), 7.38–7.36 (m, 1H), 7.22–7.18 (m, 1H),7.15 (s, 1H), 7.03 (d, *J* = 4.0 Hz, 1H); ^13^C{^1^H}-NMR (100 MHz, CDCl_3_): δ 156.5 (t, *J* = 28.0 Hz), 149.6, 145.9, 145.1, 131.0, 130.5, 130.3, 128.5, 127.2, 122.9, 121.2, 115.6 (t, *J* = 304.0 Hz), 114.9, 104.3 (t, *J* = 3.0 Hz); ^19^F{^1^H}-NMR (376 MHz, CDCl_3_): δ −53.13 (s, 2F); HRMS (HR-ESI) *m*/*z*: [M + H]^+^ calcd for C_15_H_9_BrF_2_N_3_S 379.9668; found, 379.9656.

#### 2-(3,3,3,3,3,3,3-Heptafluoro-3λ^8^-prop-1-yn-1-yl)-4-phenylbenzo[4,5]imidazo[1,2-*a*]pyrimidine **(5i)**

The title compound was synthesized according to the general procedure, purified by column chromatography (Hexane/EtOAc = 8/2), and obtained as a yellow solid (57.8 mg, 70%); mp. 184–186 °C; ^1^H NMR (400 Hz, CDCl_3_) δ 8.05 (d, *J* = 8.0 Hz, 1H), 7.78–7.63 (m, 5H), 7.57–7.53 (m, 1H), 7.17–7.13 (m, 1H), 7.07 (s, 1H), 6.81 (d, *J* = 8.0 Hz, 1H); ^13^C{^1^H}-NMR (100 MHz, CDCl_3_): δ 152.0 (t, *J* = 24.0 Hz), 151.6, 149.9, 145.9, 131.9, 131.6, 129.8, 128.3, 127.3, 122.9, 121.3, 115.8 (t, *J* = 357.0 Hz), 115.2, 104.7 (t, *J* = 3.0 Hz); ^19^F {^1^H}-NMR (376 MHz, CDCl_3_): δ −79.92 (s, 3F), −115.04 (s, 2F), −125.53 (s, 2F); HRMS (HR-ESI) *m*/*z*: [M + H]^+^ calcd for C_19_H_11_F_7_N_3_ 414.0841; found, 414.0829.

#### Morpholino(4-phenylbenzo[4,5]imidazo[1,2-*a*]pyrimidin-2-yl)methanethione **(8)**

The title compound was synthesized according to the general procedure, purified by column chromatography (Hexane/EtOAc = 7/3), and obtained as a yellow solid (37.4 mg, 50%); mp 272–274 °C; ^1^H NMR (400 Hz, CDCl_3_): δ 8.05 (d, *J* = 12.0 Hz, 1H), 7.58–7.70 (m, 5H), 7.57–7.53 (m, 1H), 7.33–7.31 (m, 1H), 7.16–7.12 (m, 1H), 6.82 (d, *J* = 8.0 Hz, 1H), 4.52 (t, *J* = 4.0 Hz, 2H), 4.06–4.02 (m, 4H), 3.96–3.95 (m, 2H); ^13^C{^1^H}-NMR (100 MHz, CDCl_3_): δ 193.9, 160.4, 149.9, 131.4, 129.5, 128.4, 127.5, 126.5, 122.0, 120.4, 114.8, 109.3, 67.1, 66.5, 52.8, 49.8; HRMS (HR-EI) *m*/*z*: [M + H]^+^ calcd for C_21_H_19_N_4_OS 375.1279; found, 375.1268.

## Data Availability

The data underlying this study are available in the published article and its Supporting Information.

## Supporting Information Available

Experimental procedures, characterization data, spectra (^1^H,^13^ C{^1^H}, and ^19^F NMR), and X-ray crystal data (PDF)
