# Supplementary material for: Sustainable Synthesis of Polyfluoro-Pyrimido [1,2-a] Benzimidazole Derivatives Using a Tandem Strategy—Ultrasound and an Integrated Continuous Flow System
Source: J Org Chem. 2025 Mar 7;90(11):4018–27. doi: 10.1021/acs.joc.4c03123 (PMC11934136; doi:10.1021/acs.joc.4c03123)
Supplement: Supplementary file 1 — jo4c03123_si_001.pdf [file jo4c03123_si_001.pdf]

Supporting Information for

Sustainable Synthesis of Polyfluoro-Pyrimido [1,2-*a*] Benzimidazole Derivatives Using Tandem Strategy - Ultrasound and Integrated Continuous Flow System

Vijay Thavasianandam Seenivasan,[a] Nian-Qi Chen,[a] Karthick Govindan,[a] Alageswaran Jayaram,[a] Yu-Chen Lin,[a] Chien-Hung Li, [a] Wei-Yu Lin * [a,b,c]

[a] Department of Medicinal and Applied Chemistry, Kaohsiung Medical University, Kaohsiung 80708, Taiwan, ROC.

[b] Department of Medical Research, Kaohsiung Medical University Hospital, Kaohsiung 80708, Taiwan, ROC.

[c] Drug Development and Value Creation Research Centre, Kaohsiung Medical University, Kaohsiung 80708, Taiwan, ROC.

*Corresponding author: Wei-Yu Lin, wylin@kmu.edu.tw

List of Contents

1	**General information**	**S3**
2	**Synthesis of starting materials (1aa-1am)**	**S4**
3	**Synthesis of 2-(2,2-dichlorovinyl)thiophene (1an)**	**S4**
4	**Synthesis of (*E*)-(4,4-dibromobuta-1,3-dien-1-yl)benzene (1ao)**	**S4**
5	**Synthesis of CF_3-ynones (1a-1o)**	**S5**
6	**Synthesis of CF_2Br-ynones (4a-4i)**	**S5**
7	**Experimental procedure for CF_3-substituted benzo [4,5]imidazo [1,2-a] pyrimidine (3a-3o)**	**S5**
8	**Experimental procedure for polyfluoro-substituted benzo[4,5]imidazo[1,2-a]pyrimidine (5a-5i)**	**S6**
9	**General procedure for the integrated continuous flow approach & synthetic transformation**	**S7-S10**
9.1	**Optimization table of continuous flow method**	**S7**
9.2	**Synthesis of the CF_3-substituted benzo[4,5]imidazo[1,2-a] pyrimidine derivatives in continuous flow method (3a, 3c, and 3f)**	**S7**
9.3	**Synthesis of the polyfluoro-substituted benzo[4,5]imidazo[1,2-a]pyrimidine derivatives in integrated continuous flow method (3a, 5a, and 5i)**	**S8**
9.4	**Experimental procedure for morpholino(4-phenyl benzo[4,5]imidazo[1,2-a]pyrimidin-2-yl)methanethione (8)**	**S10**
9.5	**Possible reaction mechanism**	**S10**
10	**Check CIF file of compound 3a**	**S12-S15**
11	**Characterization data of starting materials**	**S16-S17**
12	**References**	**S18**
13	**^1H and ^{13}C spectra**	**S18-S120**

1. General information

All chemicals were purchased from commercial providers (Sigma Aldrich, Alfa Aesar, TCI, and Matrix Scientific) and used directly without further purification unless otherwise noted. Well-cleaned and oven-dried glassware was used for the experiments. The reaction was monitored by Thin Layer Chromatography (TLC), purchased as pre-coated with silica gel 60 F254 from Merck. Column chromatography was carried out using the silica gel 230-400 mesh (purchased from Merck) with a mixture of ethyl acetate/hexane or hexane as the eluent. ^{1}H NMR spectra were recorded on 400 MHz, ^{13}C-NMR spectra were recorded on 100 MHz and Varian mercury and Jeol spectrometer using $CDCl_3$ or DMSO-d_6 as solvent. The spectra were recorded and presented in chemical shifts (ppm) with tetramethylsilane (TMS) used as an internal standard. Multiplicities were provided in s (singlet), d (doublet), t (triplet), q (quartet), bs (broad singlet), m (multiplet), and dd (doublet of doublet), Coupling constants (*J*) were reported in Hz. All the compounds were characterized by ESI mass on Thermo Finnigan (TRACEGC- POLARISQ) and HRMS (QTOF-ESI) on the JMS-700 spectrometer. Melting points were determined using Fargo instruments.

Flow setup:

Continuous flow setups such as T-shaped mixers were purchased from Sanko Seiki company, stainless tubes, and peeks were purchased from Idex Health & science. Harvard syringe pumps model-11 elite were used for the reaction.

Ultrasound setup:

Ultrasound setup from Delta Ultrasonic cleaner DC900H (frequency: 40 KHz) was used for the reaction.

2. Synthesis of starting materials (1aa-1am): [1]

According to the literature procedure, the starting materials (**1aa-1am**) were synthesized on a 3.0 mmol scale and obtained a 50-80% yield. The ^{1}H-NMR spectra of (**1aa-1am**) were matched with previous literature. Other starting materials (**1ah, 1al,** and **1ak**) were characterized by NMR and HRMS the detailed data are shown in page S15.

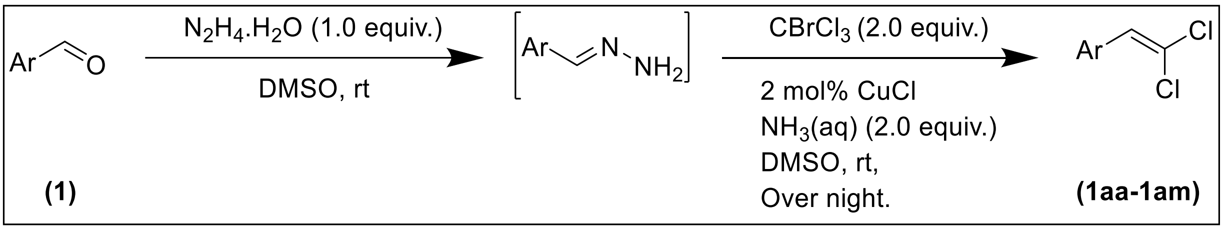

Scheme S1 General procedures for the synthesis of starting materials (**1aa-1am**)

3. Synthesis of 2-(2,2-dichlorovinyl)thiophene (1an): [2]

According to the literature procedure, the starting material (**1an**) was synthesized on a 3.0 mmol scale and obtained a 30 % yield. The ^{1}H-NMR spectra of (**1an**) matched with previous literature.

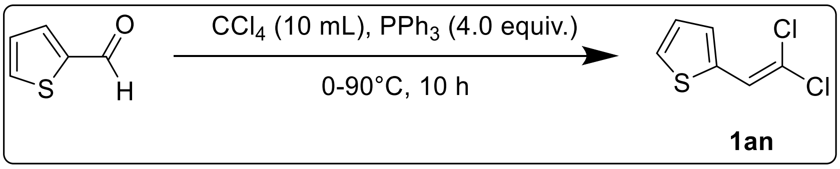

Scheme S2 General procedures for the synthesis of 2-(2,2-dichlorovinyl) thiophene (**1an**)

4. Synthesis of (*E*)-(4,4-dibromobuta-1,3-dien-1-yl)benzene (1ao): [3]

According to the literature procedure, the starting material (**1ao**) was synthesized on a 3.0 mmol scale and obtained a 80% yield. The ^{1}H-NMR spectra of (**1ao**) matched with previous literature.

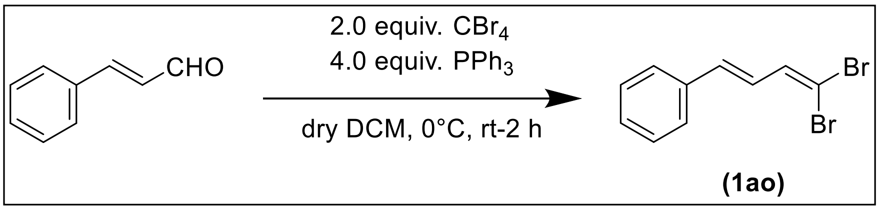

Scheme S3 General procedures for the synthesis of (*E*)-(4,4-dibromobuta-1,3-dien-1-yl)benzene (**1ao**)

5. Synthesis of CF_3-ynones (1a-1o): [1]

According to the literature procedure, the starting materials (**1a-1o**) were synthesized on a 2.0 mmol scale and obtained a 45 - 80 % yield. The ^{1}H-NMR spectra of (**1a-1o**) were matched with previous literature. Other starting materials (**1h, 1i, 1l, 1k, 1l, 1m,** and **1o**) were characterized by NMR and HRMS the detailed data are shown on pages S15-S17.

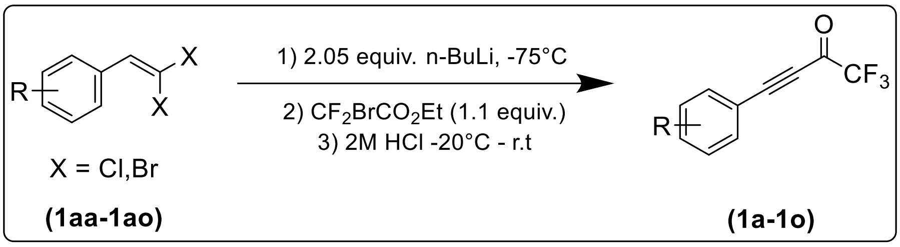

Scheme S4 General procedures for the synthesis of CF_3-ynones (**1a-1o**)

6. Synthesis of CF_2-Br ynones (4a-4i): [4]

According to the literature procedure, the starting materials (**4a-4i**) were synthesized on a 2.0 mmol scale and obtained a 45-80 % yield. The ^{1}H-NMR spectra of (**4a-4i**) were matched with previous literature. Other starting materials (**4f** and **4g**) are characterized by NMR and HRMS the detailed data are shown on page S17.

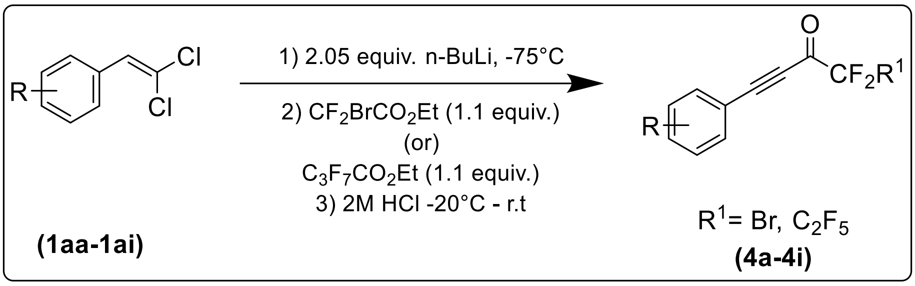

Scheme S5 General procedures for the synthesis of CF_2-Br ynones (**4a-4i**)

7. Experimental procedure for CF_3-substituted benzo[4,5]imidazo[1,2-a]pyrimidine (3a-3o):

A 10 mL vial was charged with **1a-1o** (0.24 mmol, 1.2 equiv.)**, 2** (0.2 mmol, 1.0 equiv.), neat in an open-air atmosphere. Then irradiation under ultrasound for 60 minutes. After the indicated time, the reaction mixture was diluted with 5 mL of water, the aqueous layer was extracted with ethyl acetate (3 x 10 mL), and the combined ethyl acetate layer was washed with brine solution (1 x 5 mL). The final ethyl acetate layer was then dried over $MgSO_4$ and concentrated under reduced pressure to get

the crude product. The obtained crude product was purified using column chromatography by eluting with ethyl acetate/hexane to afford pure CF_3-substituted benzo[4,5]imidazo[1,2-a]pyrimidine derivatives (**3a-3o**) up to 25-95% yields.

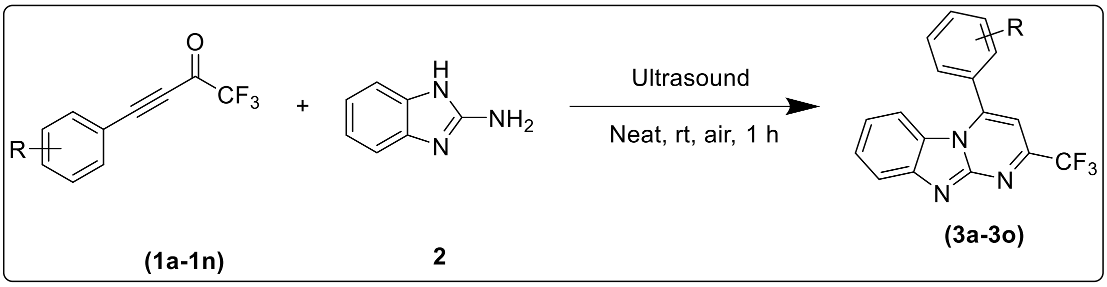

Scheme S6 General procedures for the synthesis of CF_3-substituted benzo[4,5]imidazo[1,2-a]pyrimidine (**3a-3o**)

8. Experimental procedure for polyfluoro-substituted benzo[4,5]imidazo[1,2-a]pyrimidine (5a-5i):

A 10 mL vial was charged with **4a-4i** (0.24 mmol, 1.2 equiv.), **2** (0.2 mmol, 1.0 equiv.), neat in an open-air atmosphere. Then irradiation under ultrasound for 60 minutes. After the indicated time, the reaction mixture was diluted with 5 mL of water, the aqueous layer was extracted with ethyl acetate (3 x 10 mL), and the combined ethyl acetate layer was washed with brine solution (1 x 5 mL). The final ethyl acetate layer was then dried over $MgSO_4$ and concentrated under reduced pressure to get the crude product. The obtained crude product was purified using column chromatography by eluting with ethyl acetate/hexane to afford pure polyfluoro-substituted benzo[4,5]imidazo[1,2-a]pyrimidine derivatives (**5a-5i**) up to 70-91% yields.

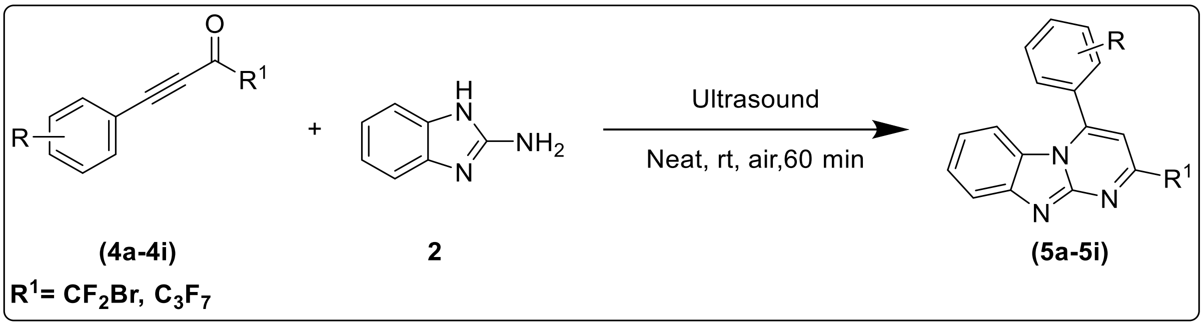

Scheme S7 General procedures for the synthesis of polyfluoro-substituted benzo[4,5]imidazo[1,2-a]pyrimidine (**5a-5i**)

9. General procedure for the integrated continuous flow approach & synthetic transformation:

9.1 Table S1 Optimization conditions of continuous flow method [a,b]

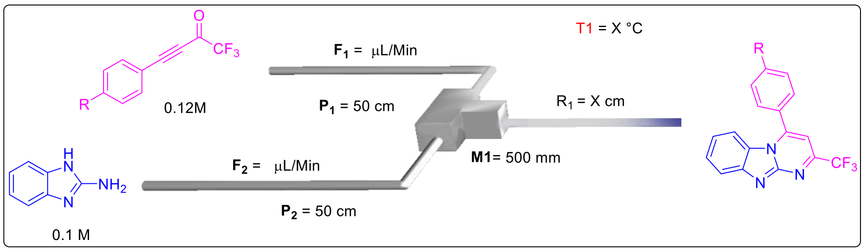

Entry	Flow rate of reactants (F_1:F_2) µL min^{-1}	Length (Volume of R_1) [cm (µL)]	Solvent	Reaction time (min)	Temperature (T1 :*X*°C)	Yield [b] (%)
1	10:10	100 (502)	1,4-dioxane	25.1	25	26
2	10:10	100 (502)	MeOH	25.1	25	N.R
3	10:10	100 (502)	EtOH	25.1	25	15
4	20:20	200 (1004)	EtOH	25.1	50	75
5	20:20	200 (1004)	EtOH	25.1	60	80
6	20:20	200 (1004)	EtOH	25.1	70	87[a]

A continuous flow system consists of two reagents delivering units (P_1, and P_2, Φ= 800µm, length L= 50 cm) T-shaped micromixer (M1, Φ = 500 µm), one microtube reactor (R_1= 100 cm,200 cm, Φ = 800 µm) [a] Reaction was performed under standard optimized conditions (F_1, F_2 = 20 µL/min) and the product was collected for 30 min. [b] isolated yield.

9.2 Synthesis of the CF_3-substituted benzo[4,5]imidazo[1,2-a]pyrimidine derivatives in continuous flow method (3a, 3c, and 3f):

A microreactor system consists of one T-shaped micromixer (**M1**), one microtube reaction (**R_1**), two precooling units **P_1** (inner diameter Φ = 800 µm, length L = 50 cm), **P_2** (Φ= 800 µm, length L= 50 cm). A solution of **1a** (CF_3-ynones) (0.12M in EtOH) (flow rate: F_1 =20 µL/min) and a solution of 2-amino benzimidazole **2** (0.1 M in EtOH) (flow rate: F_2 =20 µL/min) were introduced into **M1** (Φ = 500 µm) by syringe pumps. The resulting solution was passed through microtubing (**R_1**= 200 cm, Φ = 800 µm)

with a reaction temperature of 70°C. After a steady state was reached, the solution was collected for 30 minutes in a vial. The resulting solution was extracted with ethyl acetate (3 mL x 5), and the organic layer was combined and washed with brine solution (5 mL). The organic layer was dried over anhydrous $MgSO_4$, filtered, and concentrated under a vacuum. The crude product was purified by column chromatography (EtOAc/Hexane 3/10 silica gel) and obtained **3a, 3c,** and **3f** in 81-90% yields.

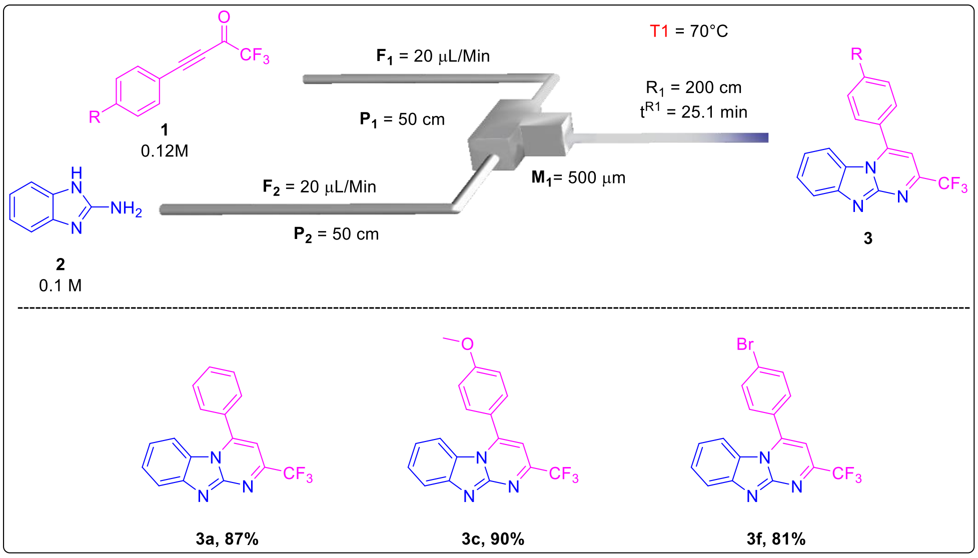

Scheme S8 General procedures for the synthesis of the CF_3-substituted benzo[4,5]imidazo[1,2-a]pyrimidine derivatives in continuous flow method (**3a, 3c,** and **3f**)

9.3 Synthesis of the polyfluoro-substituted benzo[4,5]imidazo[1,2-a]pyrimidine derivatives in integrated continuous flow system (3a, 5a, and 5i):

A microreactor system consist of three T-shaped micromixers (**M1**, **M2** and **M3**), three microtubes reaction (**R_1**, **R_2** and **R_3**) and four precooling units **P_1** (inner diameter = 800 μm, length L = 100 cm), **P_2** (inner diameter = 800 μm, length L = 50 cm), **P_3** (inner diameter = 800 μm, length L = 100 cm), and **P_4** (inner diameter = 800 μm, length L = 100 cm) were used. A solution of alkyne (0.10 M in THF) (flow rate F_1 = 6.0 mL/min) and a solution of *n*-BuLi (1.60 M in hexane) (flow rate F_2 = 0.4 mL/min) were introduced to **M1** (Φ = 0.5 mm) by syringe pumps. The solution was passed through **R_1** (Φ = 0.8 mm, L = 50 cm, 251 μL) and was mixed with a solution of BF_3OEt (0.3 M in THF) and R^1CO_2Et (R^1 = CF_3, CF_2Br, C_3F_7) (0.1 M in THF) (flow rate F_3 = 3.0 mL/min) in **M2** (Φ = 0.5 mm). The solution was passed through **R_2** (Φ = 0.8 mm, L = 50 cm, 251 μL) and was mixed with a solution of 2-amino benzimidazole **2** (0.1 M in EtOH) (flow rate F_4 = 4.5 mL/min) in **M3** (Φ = 0.5 mm). The resulting solution was passed through **R_3** (Φ = 0.8 mm, L = 300 cm, 1506 μL). After a steady state was reached (after 1 min), the

final product solution was collected for 60 seconds in a vial containing a saturated solution of NH_4Cl. The reaction mixture was analysed by GC and reported as a conversion yield of **3a, 5a,** and **5i** in 62-80%.

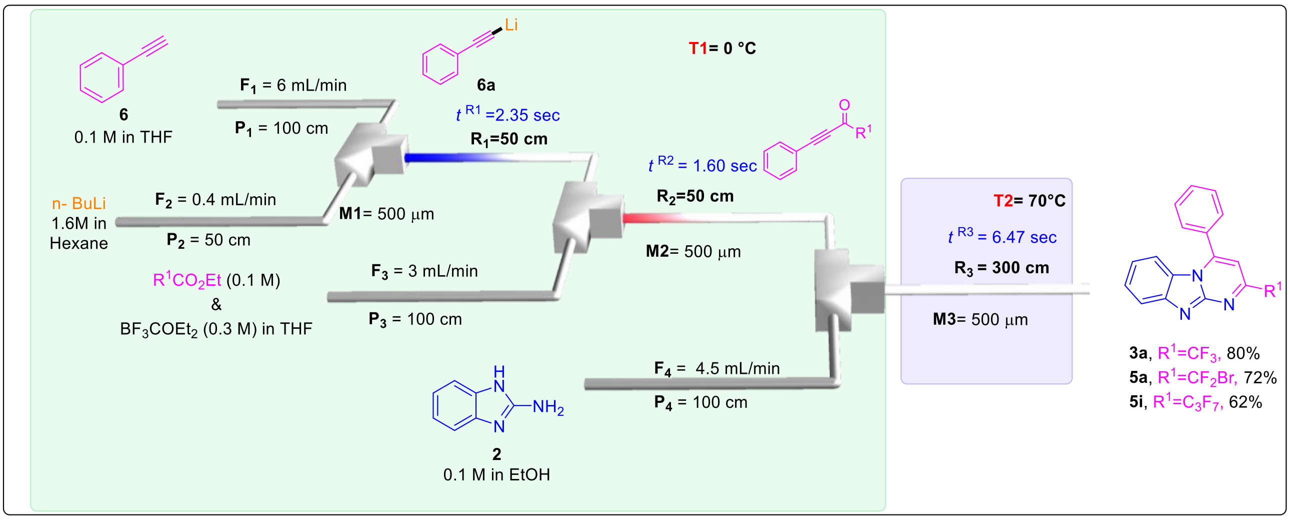

Scheme S9 General procedures for the synthesis of the polyfluoro-substituted benzo [4,5]imidazo [1,2-a]pyrimidine derivatives in integrated continuous flow system (**3a, 5a,** and **5i**)

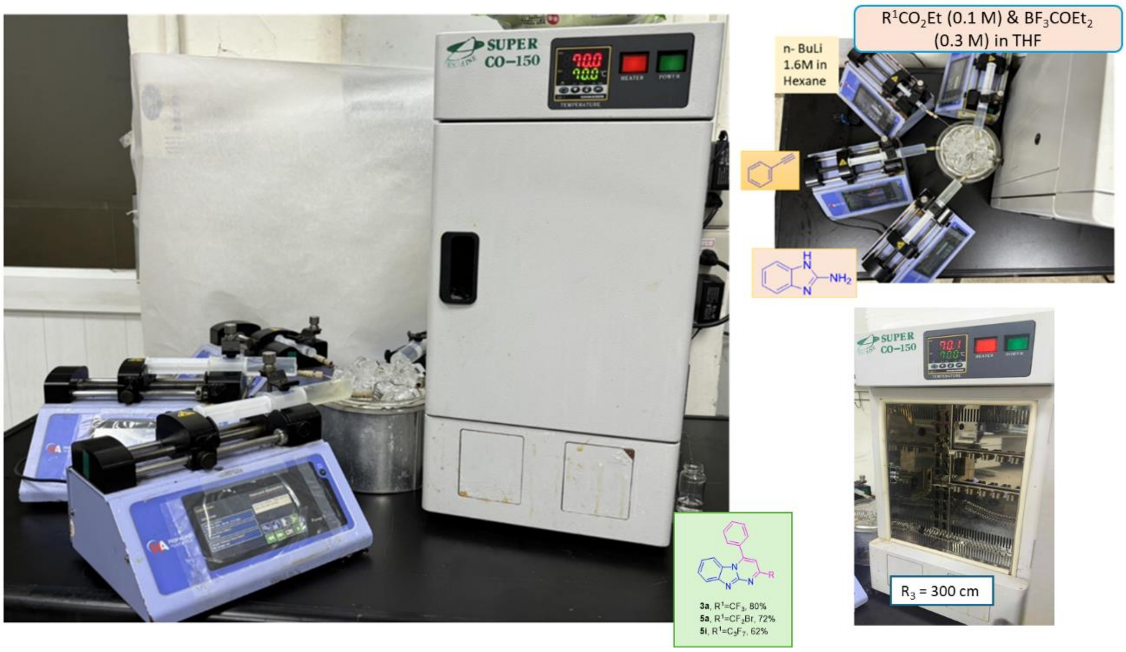

Figure S1 The setup photo of the integrated continuous flow system

9.4 Experimental procedure for morpholino(4-phenylbenzo[4,5]imidazo[1,2-a]pyrimidin-2-yl)methanethione (8):

A 10 mL vial was charged with (**5a**), (0.2 mmol, 1.0 equiv.), morphine (**7**) (0.5 mmol, 2.5 equiv.), S_8 (1.0 equiv.), Cs_2CO_3 (0.4 mmol, 2.0 equiv.) and DMSO (2 mL) in a nitrogen atmosphere. The reaction mixture was allowed to stir at 80 °C in an oil bath for about 12 h. After the indicated time, the reaction mixture was allowed the reaction mixture to reach room temperature, and then quenched with 5 mL of water, the aqueous layer was extracted with ethyl acetate (3 x 10 mL), and the combined ethyl acetate layer was washed with brine solution (1 x 5 mL). The final ethyl acetate layer was then dried over $MgSO_4$ and concentrated under reduced pressure to get the crude product. The obtained crude product was purified using column chromatography by eluting with ethyl acetate/hexane to afford pure morpholino(4-phenylbenzo[4,5]imidazo[1,2-a]pyrimidin-2-yl)methanethione (**8**) in 50 % yield.

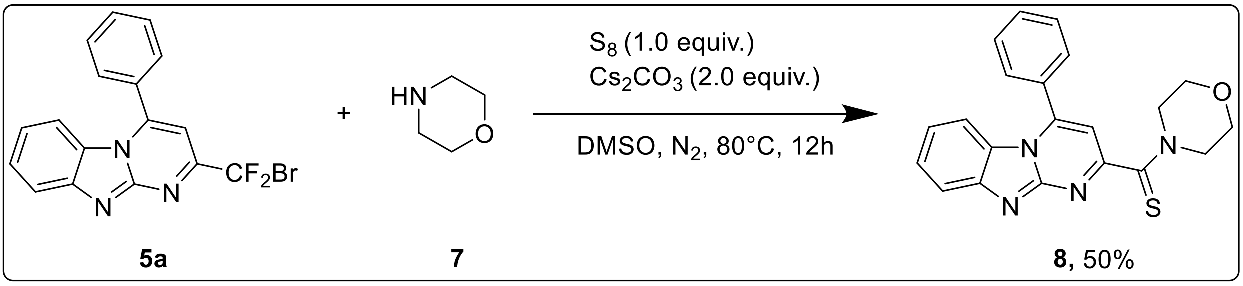

Scheme S10 General procedures for the synthesis of the morpholino(4-phenylbenzo[4,5]imidazo[1,2-a]pyrimidin-2-yl)methanethione (**8**)

9.5 Possible reaction mechanism

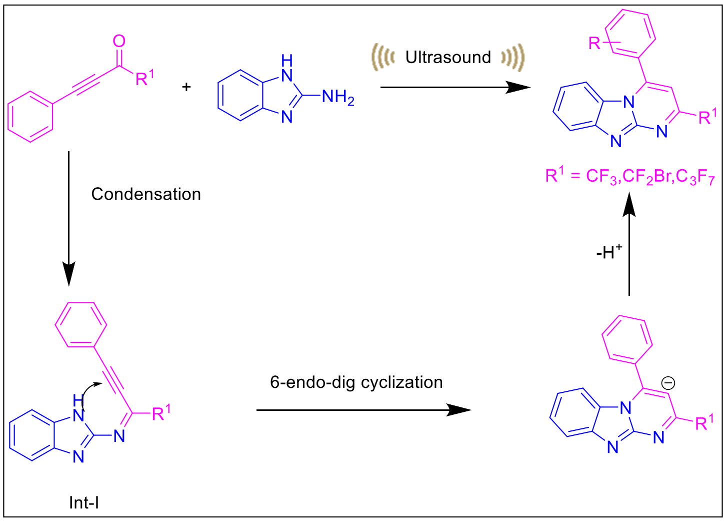

Scheme S11 Possible reaction mechanism

10. Check CIF file of compound 3a:

checkCIF/PLATON report

Structure factors have been supplied for datablock(s) k11212-wyl-b_auto

THIS REPORT IS FOR GUIDANCE ONLY. IF USED AS PART OF A REVIEW PROCEDURE FOR PUBLICATION, IT SHOULD NOT REPLACE THE EXPERTISE OF AN EXPERIENCED CRYSTALLOGRAPHIC REFEREE.

No syntax errors found. CIF dictionary Interpreting this report

Datablock: k11212-wyl-b_auto

Bond precision: C-C = 0.0031 A Wavelength=0.71073

Cell:	a=6.9111(3)	b=10.3617(3)	c=19.4390(5)
	alpha=90	beta=90	gamma=90
Temperature:	130 K		

	Calculated	Reported
Volume	1392.04(8)	1392.04(8)
Space group	P 21 21 21	P 21 21 21
Hall group	P 2ac 2ab	P 2ac 2ab
Moiety formula	C17 H10 F3 N3	C17 H10 F3 N3
Sum formula	C17 H10 F3 N3	C17 H10 F3 N3
Mr	313.28	313.28
Dx,g cm-3	1.495	1.495
Z	4	4
Mu (mm-1)	0.119	0.119
F000	640.0	640.0
F000’	640.37	
h,k,lmax	8,13,24	8,12,24
Nref	3072[1786]	2918
Tmin,Tmax	0.972,0.976	0.470,1.000
Tmin’	0.954	

Correction method= # Reported T Limits: Tmin=0.470 Tmax=1.000AbsCorr = MULTI-SCAN

Data completeness= 1.63/0.95 Theta(max)= 27.088

R(reflections)= 0.0351(2697) wR2(reflections)= 0.0863(2918)

S = 1.046 Npar= 208

The following ALERTS were generated. Each ALERT has the format

test-name_ALERT_alert-type_alert-level.

Click on the hyperlinks for more details of the test.

Alert level G

```
PLAT242_ALERT_2_G Low    'MainMol' Ueq as Compared to Neighbors of  C10       Check
PLAT850_ALERT_4_G Check Flack Parameter Exact Value 0.00 with s.u.  0.20      Check
PLAT912_ALERT_4_G Missing # of FCF Reflections Above STh/L=  0.600  56        Note
PLAT969_ALERT_5_G The 'Henn et al.' R-Factor-gap value ..........  4.149     Note
              Predicted wR2: Based on SigI**2  2.08 or SHELX Weight  8.25
PLAT978_ALERT_2_G Number C-C Bonds with Positive Residual Density.  1         Info
```

0 **ALERT level A** = Most likely a serious problem - resolve or explain

0 **ALERT level B** = A potentially serious problem, consider carefully

0 **ALERT level C** = Check. Ensure it is not caused by an omission or oversight

5 **ALERT level G** = General information/check it is not something unexpected

0 ALERT type 1 CIF construction/syntax error, inconsistent or missing data

2 ALERT type 2 Indicator that the structure model may be wrong or deficient

0 ALERT type 3 Indicator that the structure quality may be low

2 ALERT type 4 Improvement, methodology, query or suggestion

1 ALERT type 5 Informative message, check

It is advisable to attempt to resolve as many as possible of the alerts in all categories. Often the minor alerts point to easily fixed oversights, errors and omissions in your CIF or refinement strategy, so attention to these fine details can be worthwhile. In order to resolve some of the more serious problems it may be necessary to carry out additional measurements or structure refinements. However, the purpose of your study may justify the reported deviations and the more serious of these should normally be commented upon in the discussion or experimental section of a paper or in the "special_details" fields of the CIF. checkCIF was carefully designed to identify outliers and unusual parameters, but every test has its limitations and alerts that are not important in a particular case may appear. Conversely, the absence of alerts does not guarantee there are no aspects of the results needingattention. It is up to the individual to critically assess their own results and, if necessary, seek expert advice.

Publication of your CIF in IUCr journals

A basic structural check has been run on your CIF. These basic checks will be run on all CIFs submitted for publication in IUCr journals (*Acta Crystallographica*, *Journal of Applied Crystallography*, *Journal of Synchrotron Radiation*); however, if you intend to submit to *Acta Crystallographica Section C* or *E* or *IUCrData*, you should make sure that full publication checks arerun on the final version of your CIF prior to submission.

Publication of your CIF in other journals

Please refer to the *Notes for Authors* of the relevant journal for any special instructions relating to CIF submission.

PLATON version of 22/08/2024; check.def file version of 21/08/2024

Datablock k11212-wyl-b_auto - ellipsoid plot

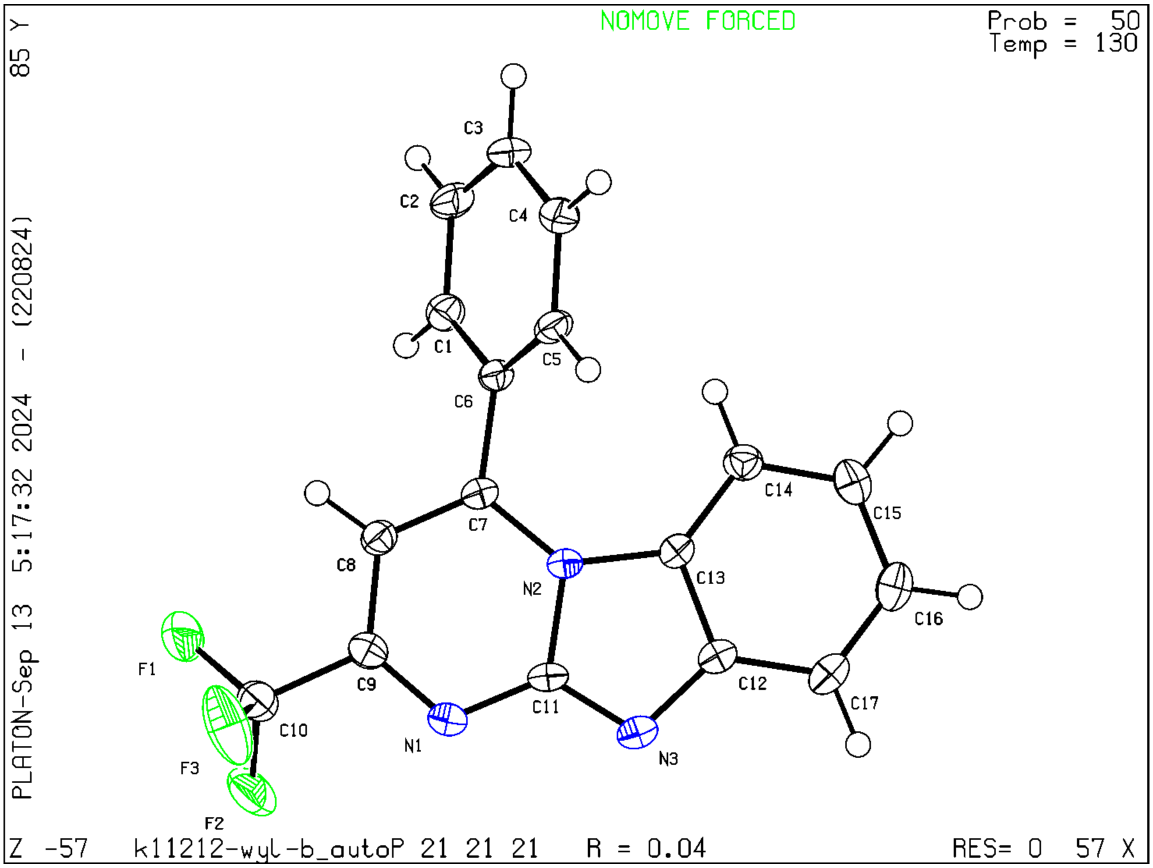

Fig S2 : The crystal structures of the **3a** were determined by single-crystal X-ray diffraction after growing crystals from a DCM/Hexane mixture solution at 273 K. The crystallographic analysis, including atom numbering for **3a**, is displayed in Figure S2. Data collection and refinement parameters are given on page S11. Selected bond distances and angles are given on fig S11. Thermal ellipsoid plots are shown at the 50% probability level.

X-ray crystal structure determinations

Suitable single crystals of the **3a** were obtained from the DCM/hexane mixture solution at room temperature and selected to determine the single-crystal structure. The X-ray diffraction data were collected by using Rigaku Oxford XtaLAB ProII using Mo Kα radiation (λ = 0.71073 A), and the unit cell parameters and the data were determined with a Bruker SMART program package. All non-hydrogen atoms were refined anisotropically. Meanwhile, hydrogen atoms were located at the calculated positions and included in the final stage of refinements with fixed parameters.

11. Characterization data of starting materials

1,3-dichloro-5-(2,2-dichloro vinyl) benzene (1ah): The title compound was synthesized according

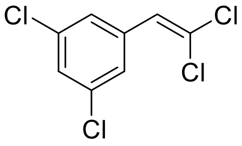

1ah

to the general procedure and obtained purified by column chromatography (Hexane) as a yellow liquid (362.8 mg, 50%); ^{1}H-NMR (400 Hz, $CDCl_3$) δ 7.39 (dd, *J* = 1.6 Hz, 0.4 Hz, 2H), 7.30-7.29 (m, 1H), 6.73 (s, 1H); ^{13}C{^{1}H} -NMR (100 MHz, $CDCl_3$) δ 136.1, 135.2, 128.5, 127.0, 126.2, 124.2; HRMS (HR-ESI) m/z: $[M]^+$ calcd for $C_8H_4Cl_4$ 239.9067; Found 239.9070.

1-(2,2-dichlorovinyl)-3,5-dimethoxybenzene (1ai): The title compound was synthesized

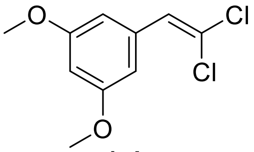

1ai

according to the general procedure and obtained purified by column chromatography (Hexane) as yellow liquid (370.5 mg, 53%); ^{1}H-NMR (400 Hz, $CDCl_3$) δ 6.80 (s, 1H), 6.69 (d, *J* = 2.0 Hz, 2H), 6.44-6.43 (m, 1H), 3.80 (s, 6H); ^{13}C{^{1}H} -NMR (100 MHz, $CDCl_3$) δ 160.7, 135.1, 128.6, 121.5, 106.9, 100.8, 55.5; HRMS (HR-ESI) m/z: $[M]^+$ calcd for $C_{10}H_{10}Cl_2O_2$ 232.0058; Found 232.0052.

3-(2,2-dichloro vinyl)phenol (1ak): The title compound was synthesized according to the general

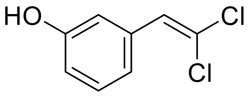

1ak

procedure and purified by column chromatography (Hexane) obtained as a black liquid (283.5 mg, 50%); ^{1}H-NMR (400 Hz, $CDCl_3$) δ 7.73-7.69 (m, 1H), 7.55-7.53 (m, 2H), 7.30-7.27 (m, 1H), 7.26 (s, 1H), 6.41 (bs, 1H); ^{13}C{^{1}H} -NMR (100 MHz, $CDCl_3$) δ 155.5, 135.0, 129.8, 128.3, 121.7, 115.8, 115.3; HRMS (HR-ESI) m/z: $[M]^+$ calcd for $C_8H_6Cl_2O$ 187.9796; Found 187.9789.

4-(3,5-dichlorophenyl)-1,1,1-trifluorobut-3-yn-2-one (1h): The title compound was synthesized

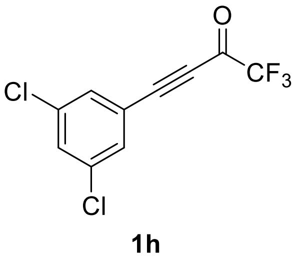

1h

according to the general procedure and purified by column chromatography (Hexane) obtained as a yellow liquid (373.8 mg, 78%); ^{1}H-NMR (400 Hz, $CDCl_3$) δ 7.54 (s, 3H); ^{13}C{^{1}H} -NMR (100 MHz, $CDCl_3$) δ 166.0 (q, *J* = 43.0 Hz), 135.0, 131.8, 130.7, 120.7 (q, *J* = 286.0 Hz), 120.0, 94.9, 82.9; ^{19}F{^{1}H} NMR (376 MHz, $CDCl_3$) δ -77.93 (s, 3F). HRMS (HR-ESI) m/z: $[M]^+$ calcd for $C_{10}H_3Cl_2F_3O$ 265.9513; Found 265.9511.

4-(3,5-dimethoxyphenyl)-1,1,1-trifluorobut-3-yn-2-one (1i): The title compound was

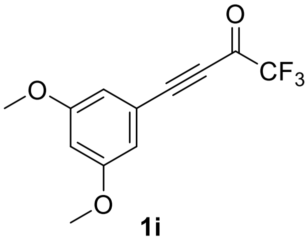

1i

synthesized according to the general procedure and purified by column chromatography (Hexane) obtained as a yellow liquid (402.7 mg, 78%); ^{1}H-NMR (400 Hz, $CDCl_3$) δ 6.70 (d, *J* = 4.0 Hz, 2H), 6.65-6.64 (m, 1H), 3.81 (s, 6H); ^{13}C{^{1}H} -NMR (100 MHz, $CDCl_3$) δ 166.3 (q, *J* = 41.0 Hz), 160.0, 118.3, 114.0 (q, *J* = 287.0 Hz), 105.5, 105.2, 99.7, 81.8, 54.7; ^{19}F{^{1}H} NMR (376 MHz, $CDCl_3$) δ -81.23 (s, 3F). HRMS (HR-ESI) m/z: $[M]^+$ calcd for $C_{12}H_9F_3O_3$ 258.0504; Found 258.0508.

1,1,1-trifluoro-4-(3-hydroxyphenyl) but-3-yn-2-one (1k): The title compound was synthesized

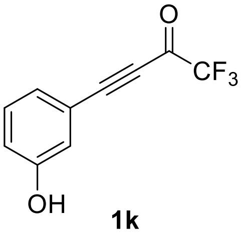

1k

according to the general procedure and purified by column chromatography (Hexane) obtained as a yellow liquid (171.3 mg, 40%); ^{1}H-NMR (400 Hz, $CDCl_3$) δ 7.32-7.29 (m, 1H), 7.23-7.21 (m, 1H), 7.10-7.11 (m, 1H), 7.06-7.04 (m, 1H); ^{13}C{^{1}H} -NMR (100 MHz, $CDCl_3$) δ 167.3 (q, *J* = 42.0 Hz), 156.3, 130.4, 126.5, 120.6, 120.4. 119.1, 115.0 (q, *J* = 287.0 Hz), 100.9, 83.2; ^{19}F{^{1}H} NMR (376 MHz, $CDCl_3$) δ -77.86 (s, 3F). HRMS (HR-ESI) m/z: $[M]^+$ calcd for $C_{10}H_5F_3O_2$ 214.0242; Found 214.0239.

1,1,1-trifluoro-4-(4-hydroxyphenyl)but-3-yn-2-one (1l): The title compound was synthesized

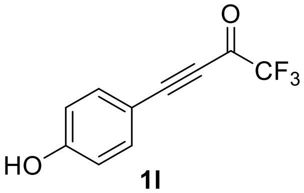

1l

according to the general procedure and purified by column chromatography (Hexane) obtained as a yellow liquid (192.7 mg, 45%); ^{1}H-NMR (400 Hz, $CDCl_3$) δ 7.92-7.89 (m, 1H), 7.59 (d, *J* = 4.0 Hz, 1H), 6.94-6.88 (m, 3H); ^{13}C{^{1}H} -NMR (100 MHz, $CDCl_3$) δ 160.0, 136.8, 130.7 (q, *J* = 274.0 Hz), 116.5, 116.2, 115.5, 102.6, 84.2; ^{19}F{^{1}H} NMR (376 MHz, $CDCl_3$) δ -77.64 (s, 3F). HRMS (HR-ESI) m/z: $[M]^+$ calcd for $C_{10}H_5F_3O_2$ 214.0242; Found 214.0246.

4-([1,1'-biphenyl]-4-yl)-1,1,1-trifluorobut-3-yn-2-one (1m): The title compound was synthesized

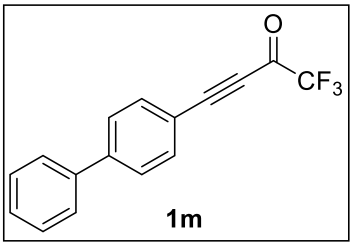

1m

according to the general procedure and purified by column chromatography (Hexane) obtained as a white solid (438.8 mg, 80%); mp. 117-119 °C; ^{1}H-NMR (400 Hz, $CDCl_3$) δ 7.75 (d, *J* = 12.0 Hz, 1H), 7.68 (d, *J* = 8.0 Hz, 2H), 7.64-7.62 (m, 2H), 7.52-7.48 (m, 2H), 7.45-7.42 (m, 2H); ^{13}C{^{1}H} -NMR (100 MHz, $CDCl_3$) δ 167.3 (q, *J* = 42.0 Hz), 145.6, 139.5, 134.7, 129.2, 128.8, 127.7, 127.3, 116.8, 115.1 (q, *J* = 286.0 Hz), 100.9, 84.4; ^{19}F{^{1}H} NMR (376 MHz, $CDCl_3$) δ -80.29 (s, 3F). HRMS (HR-ESI) m/z: $[M+H]^+$ calcd for $C_{16}H_{10}F_3O$ 275.0683; Found 275.0677.

(E)-1,1,1-trifluoro-6-phenylhex-5-en-3-yn-2-one (1o): The title compound was synthesized

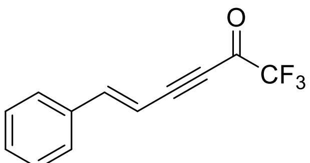

1o

according to the general procedure and purified by column chromatography (Hexane) obtained as a red liquid (179.3 mg, 40%); ^{1}H-NMR (400 Hz, $CDCl_3$) δ 7.43-7.40 (m, 2H), 7.36-7.33 (m, 4H), 6.26 (d, *J* = 8.0 Hz, 1H); ^{13}C{^{1}H} -NMR (100 MHz, $CDCl_3$) δ 152.4, 134.6, 131.4, 129.3, 128.8, 128.4 (q, *J* = 246.0 Hz), 127.7, 103.7, 100.9, 85.5; ^{19}F{^{1}H} NMR (376 MHz, $CDCl_3$) δ -77.68 (s, 3F). HRMS (HR-ESI) m/z: $[M+H]^+$ calcd for $C_{12}H_8F_3O$ 225.0524; Found 225.0527.

1-bromo-4-(3,5-dimethoxyphenyl)-1,1-difluorobut-3-yn-2-one (4f): The title compound was

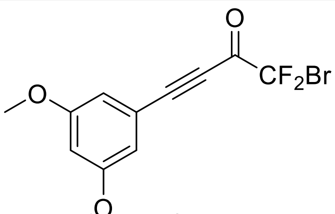

4f

synthesized according to the general procedure and purified by column chromatography (Hexane) obtained as a yellow liquid (497.7 mg, 78%); %); ^{1}H-NMR (400 Hz, $CDCl_3$) δ 7.41-7.40 (m, 2H), 7.27-7.26 (m, 1H), 4.44 (s, 6H); ^{13}C{^{1}H} -NMR (100 MHz, $CDCl_3$) δ 168.4 (t, *J* = 32.0 Hz), 160.9, 119.4, 111.3, 111.3 (t, *J* = 315.0 Hz), 105.9, 100.6, 81.4, 55.6; ^{19}F{^{1}H} NMR (376 MHz, $CDCl_3$) δ -63.09 (s, 2F); HRMS (HR-ESI) m/z: $[M]^+$ calcd for $C_{12}H_9BrF_2O_3$ 317.9703; Found 317.9702.

1-bromo-1,1-difluoro-4-(thiophen-2-yl)but-3-yn-2-one (4g): The title compound was

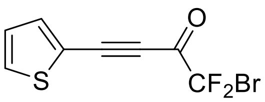

1g

synthesized according to the general procedure and purified by column chromatography (Hexane) obtained as a yellow liquid (212.1 mg, 40%); %); ^{1}H-NMR (400 Hz, $CDCl_3$) δ 7.67-7.65 (m, 2H), 7.16-7.14 (m, 1H);

$^{13}C\{^1H\}$-NMR (100 MHz, $CDCl_3$) δ 168.1 (t, *J* = 32.0 Hz), 139.4, 134.7, 128.4, 112.8 (t, *J* = 315.0 Hz, 1H), 118.1, 96.1, 87.6; $^{19}F\{^1H\}$ NMR (376 MHz, $CDCl_3$) δ -62.64 (s, 2F); HRMS (HR-ESI) m/z: $[M]^+$ calcd for $C_8H_3BrF_2OS$ 263.9056; Found 263.9053.

References:

(1) Muzalevskiy, V. M.; Sizova, Z. A.; Diusenov, A. I.; Shastin, A. V.; Nenajdenko, V. G. Efficient Multigram Approach to Acetylenes and CF3-ynones Starting from Dichloroalkenes Prepared by Catalytic Olefination Reaction (COR). *Eur. J. Org. Chem.* **2020**, *2020*, 4161-4166.

(2) Maddali, L. R.; Meka, S. Cross-coupling reactivity of 1, 1-dichloroalkenes under palladium catalysis: domino synthesis of diarylalkynes. *New J Chem* **2018**, *42*, 4412-4418.

(3) Yamamoto, K.; Bruun, T.; Kim, J. Y.; Zhang, L.; Lautens, M. A new multicomponent multicatalyst reaction (MC) 2R: chemoselective cycloaddition and latent catalyst activation for the synthesis of fully substituted 1, 2, 3-triazoles. *Org. Lett.* **2016**, *18*, 2644-2647.

(4) Li, S.; Zhou, L. gem-Difluoro-Masked o-Quinone Methides Generated by Photocatalytic Radical (3+ 3) Annulation and Their (4+ 1) Cycloaddition with Sulfur Ylides. *Org. Lett.* **2023**, *25*, 8700-8705.

Supplementary Information

^{1}H and ^{13}C spectra

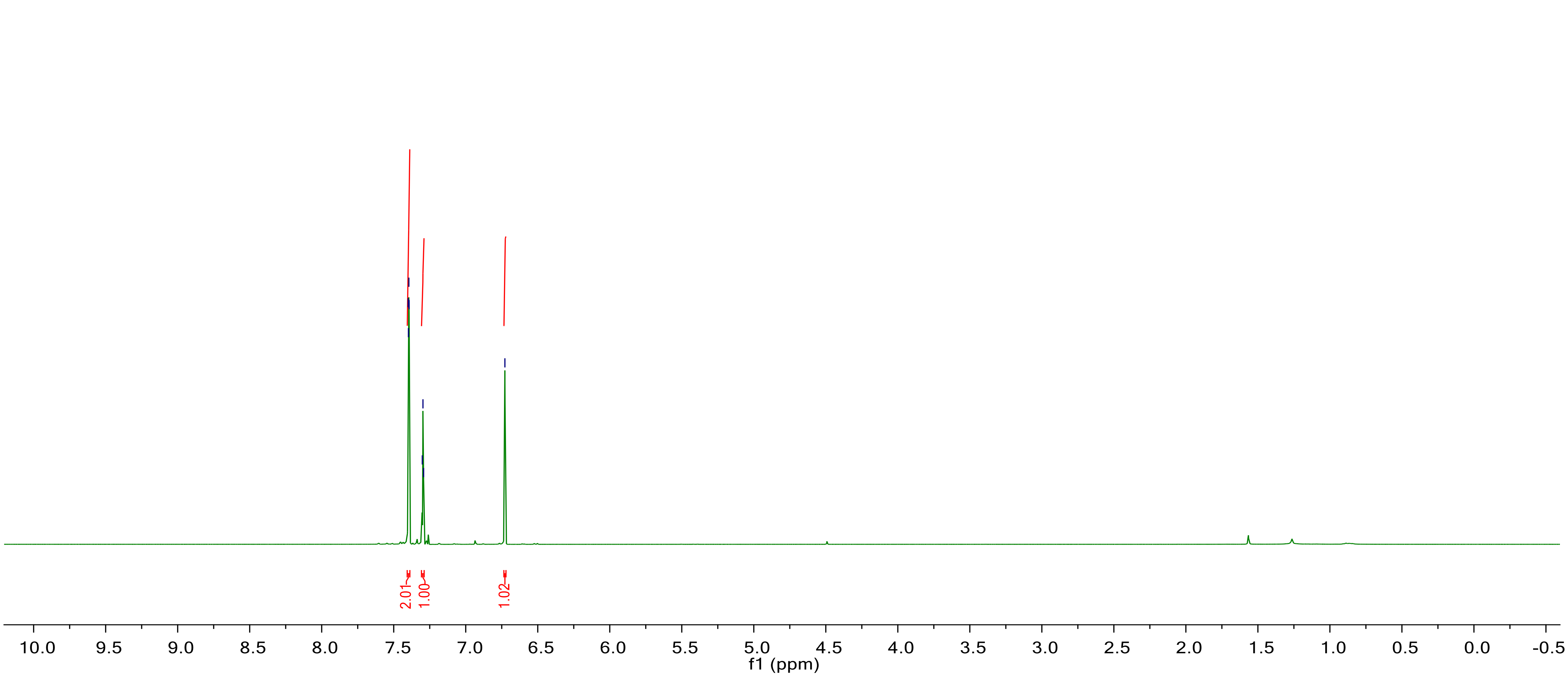

^{1}H NMR spectrum
Solvent: $CDCl_3$
Spectrometer Frequency: 400 MHz

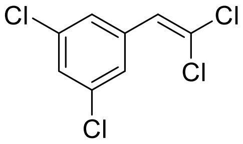

1ah

$^{13}C\ \{^{1}H\}$ NMR spectrum
Solvent: $CDCl_3$
Spectrometer Frequency: 100 MHz

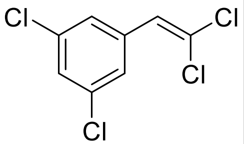

1ah

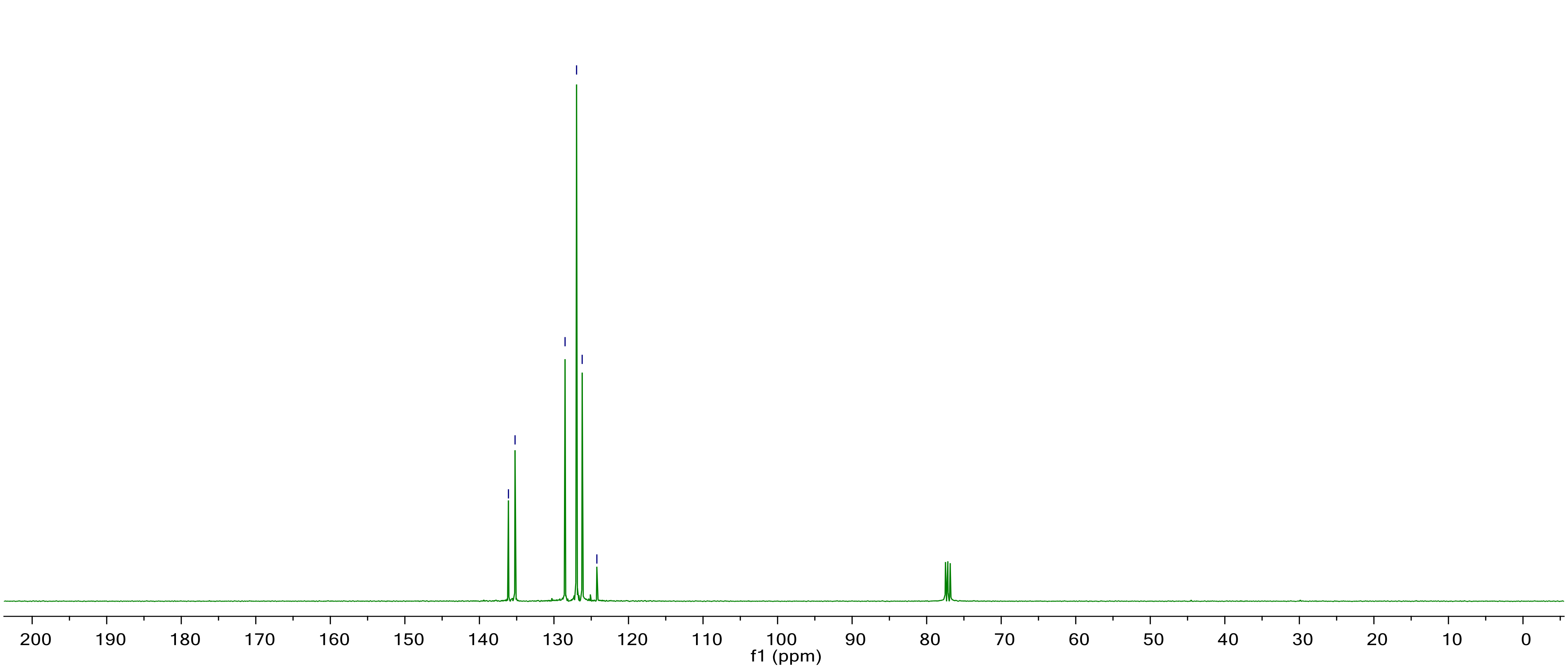

^{1}H NMR spectrum
Solvent: $CDCl_3$
Spectrometer Frequency: 400 MHz

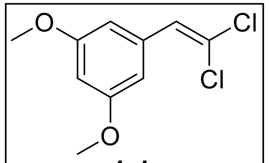

1ai

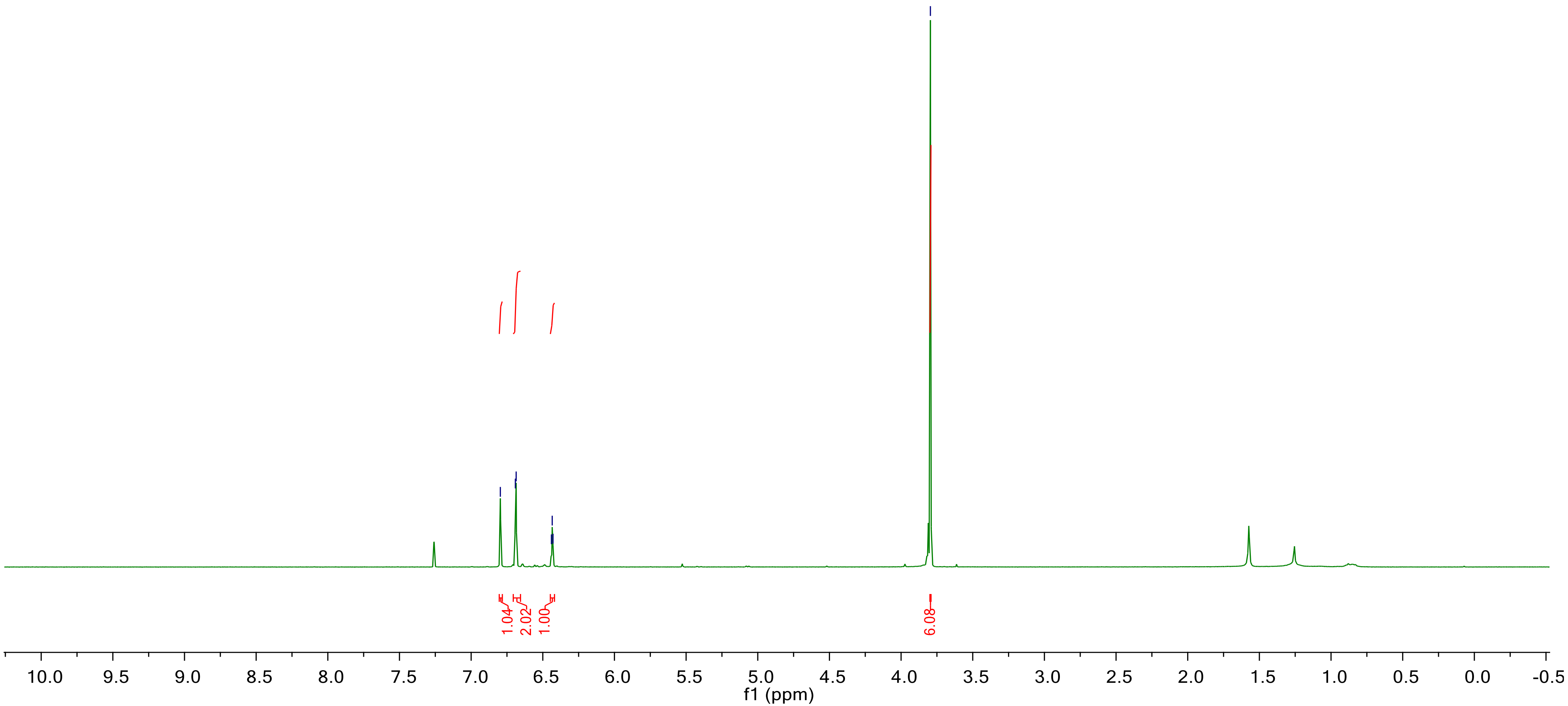

^{13}C {^{1}H} NMR spectrum
Solvent: $CDCl_3$
Spectrometer Frequency: 100 MHz

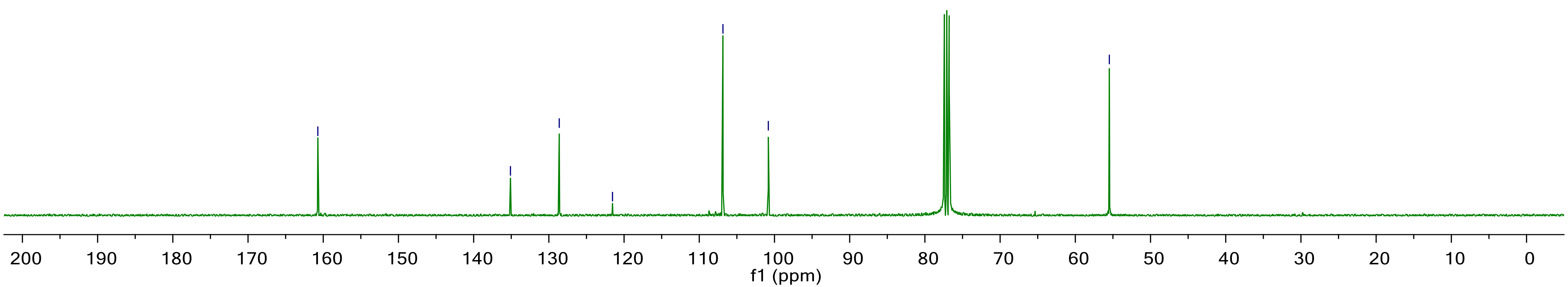

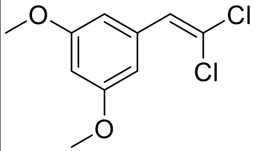

1ai

^{1}H NMR spectrum
Solvent: $CDCl_3$
Spectrometer Frequency: 400 MHz

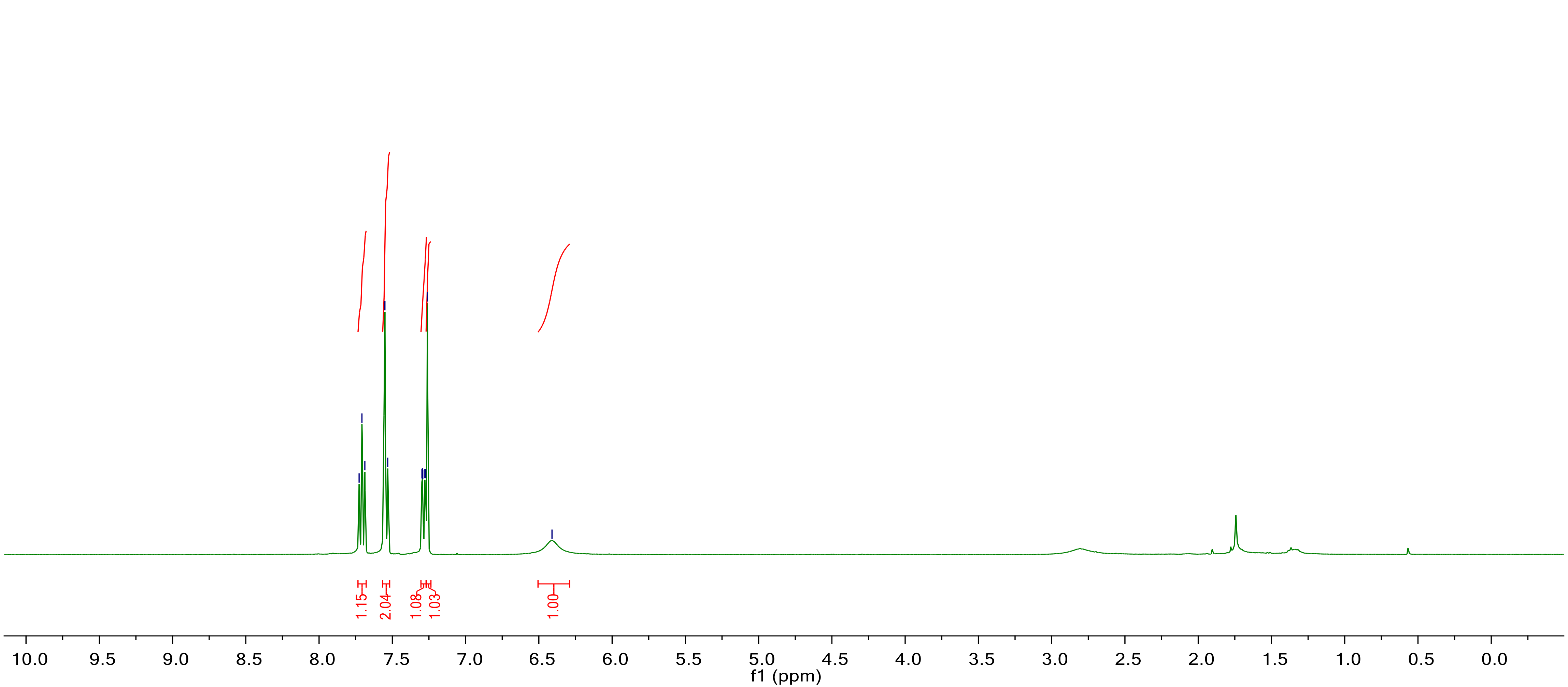

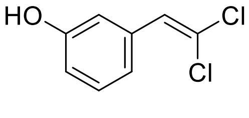

1ak

^{13}C {^{1}H} NMR spectrum
Solvent: $CDCl_3$
Spectrometer Frequency: 100 MHz

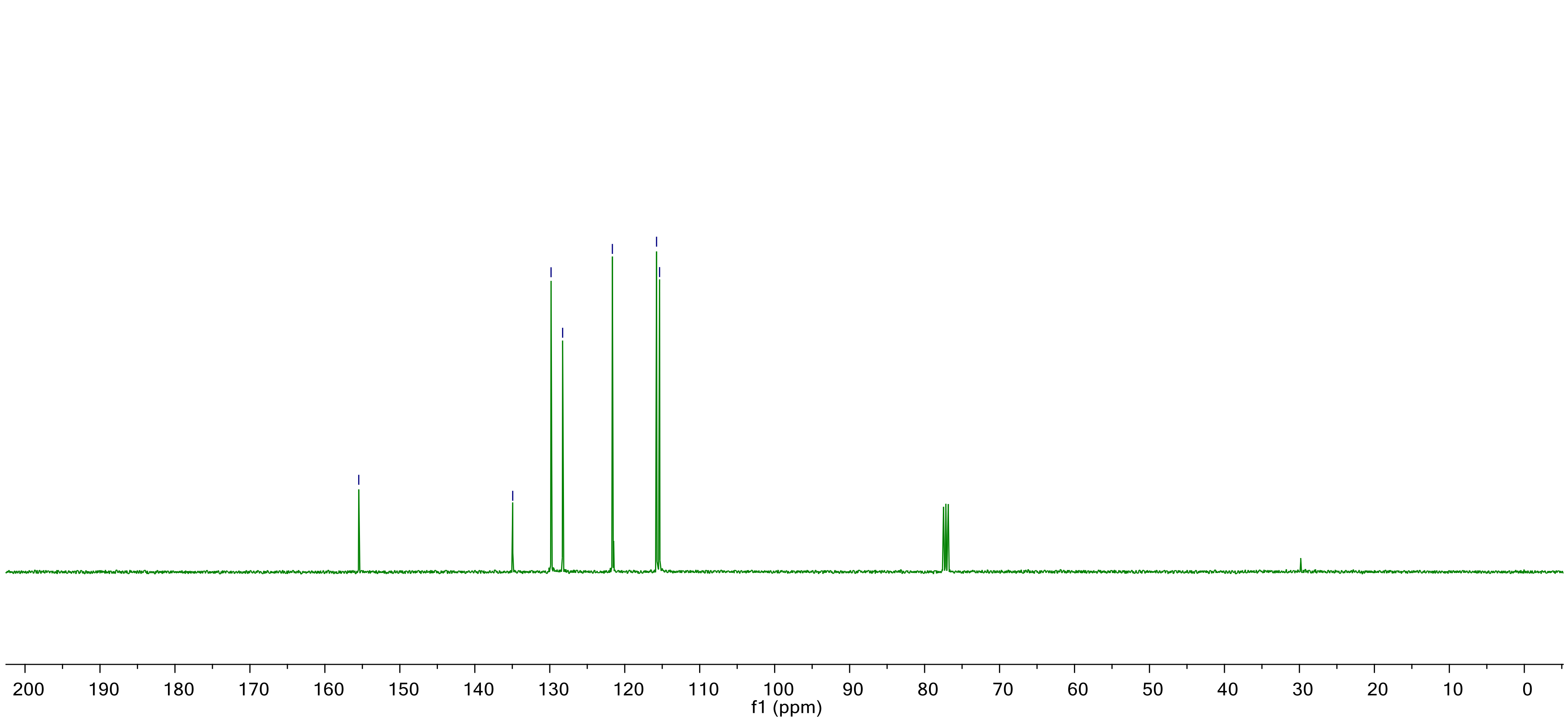

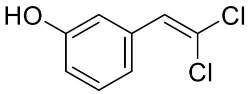

1ak

^{1}H NMR spectrum
Solvent: $CDCl_3$
Spectrometer Frequency: 400 MHz

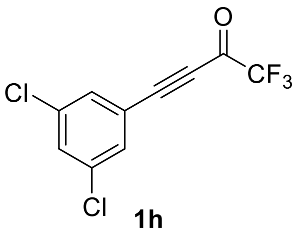

1h

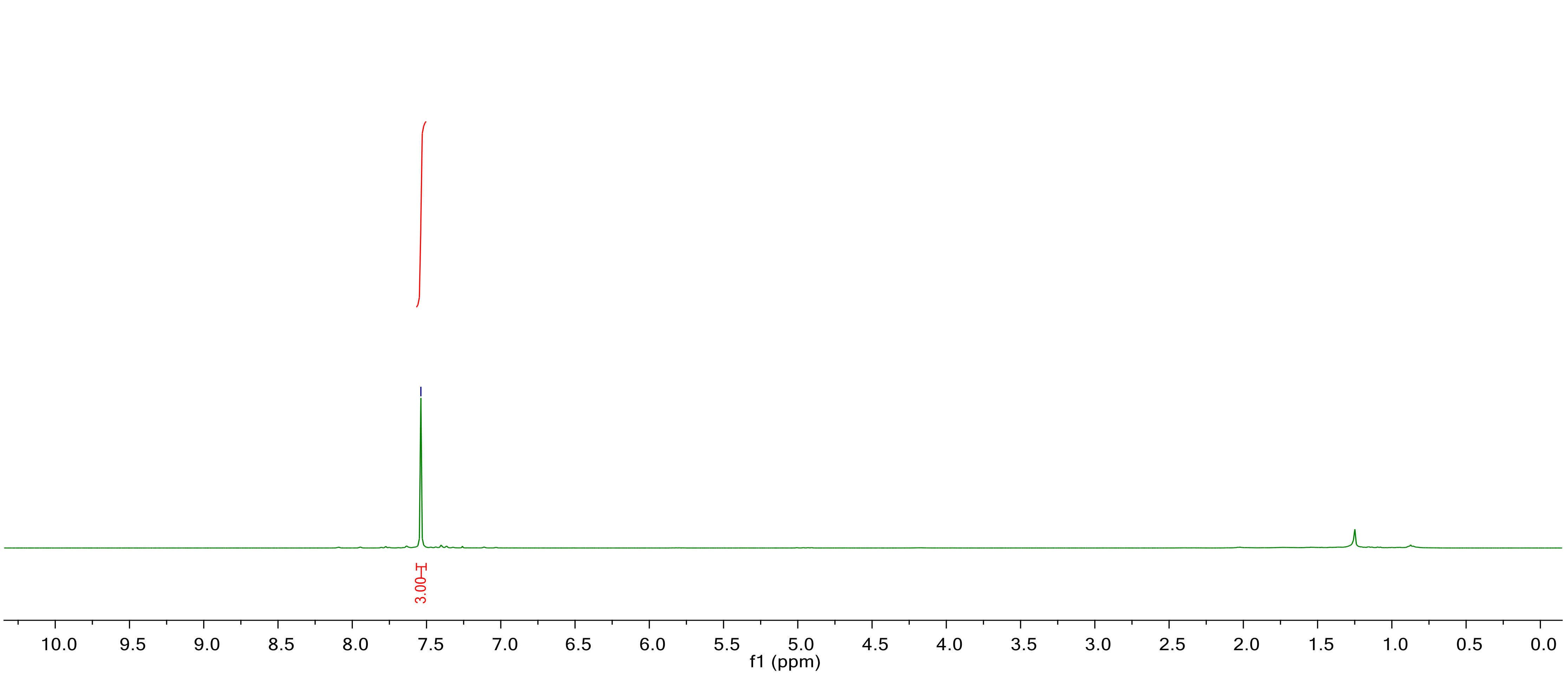

$^{19}F\{^{1}H\}$ NMR spectrum
Solvent:$CDCl_3$
Spectrometer Frequency: 376 MHz

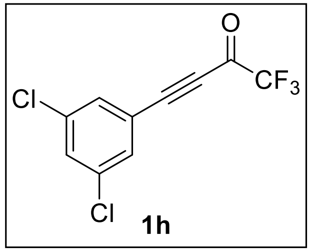

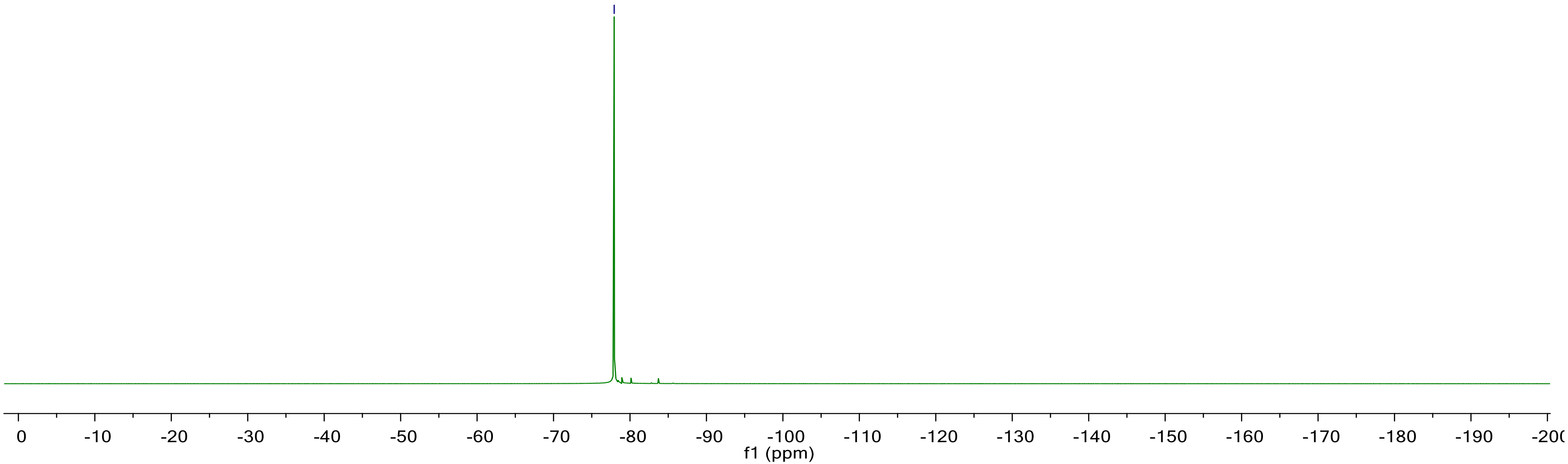

^{13}C {^1H} NMR spectrum
Solvent: $CDCl_3$
Spectrometer Frequency: 100 MHz

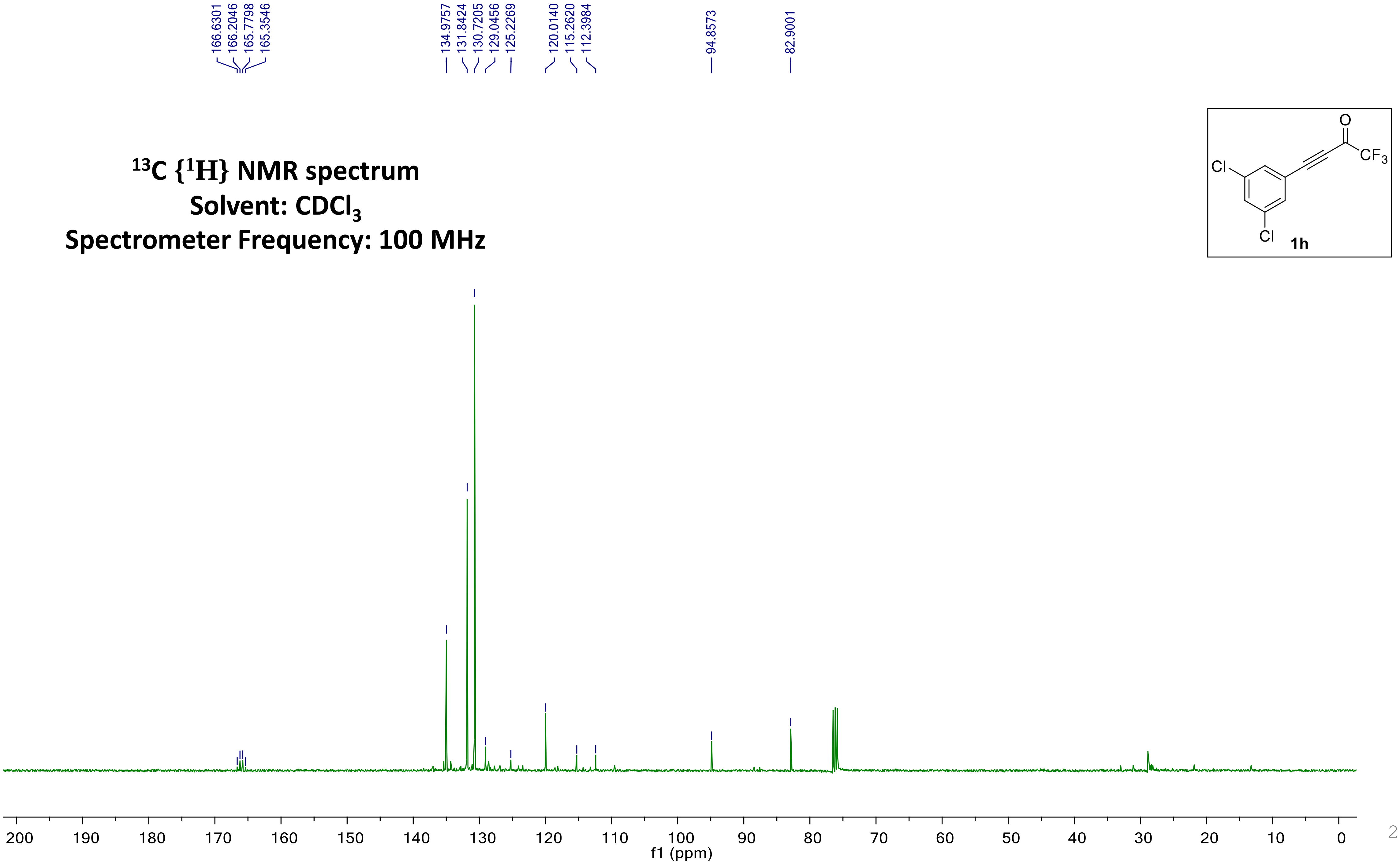

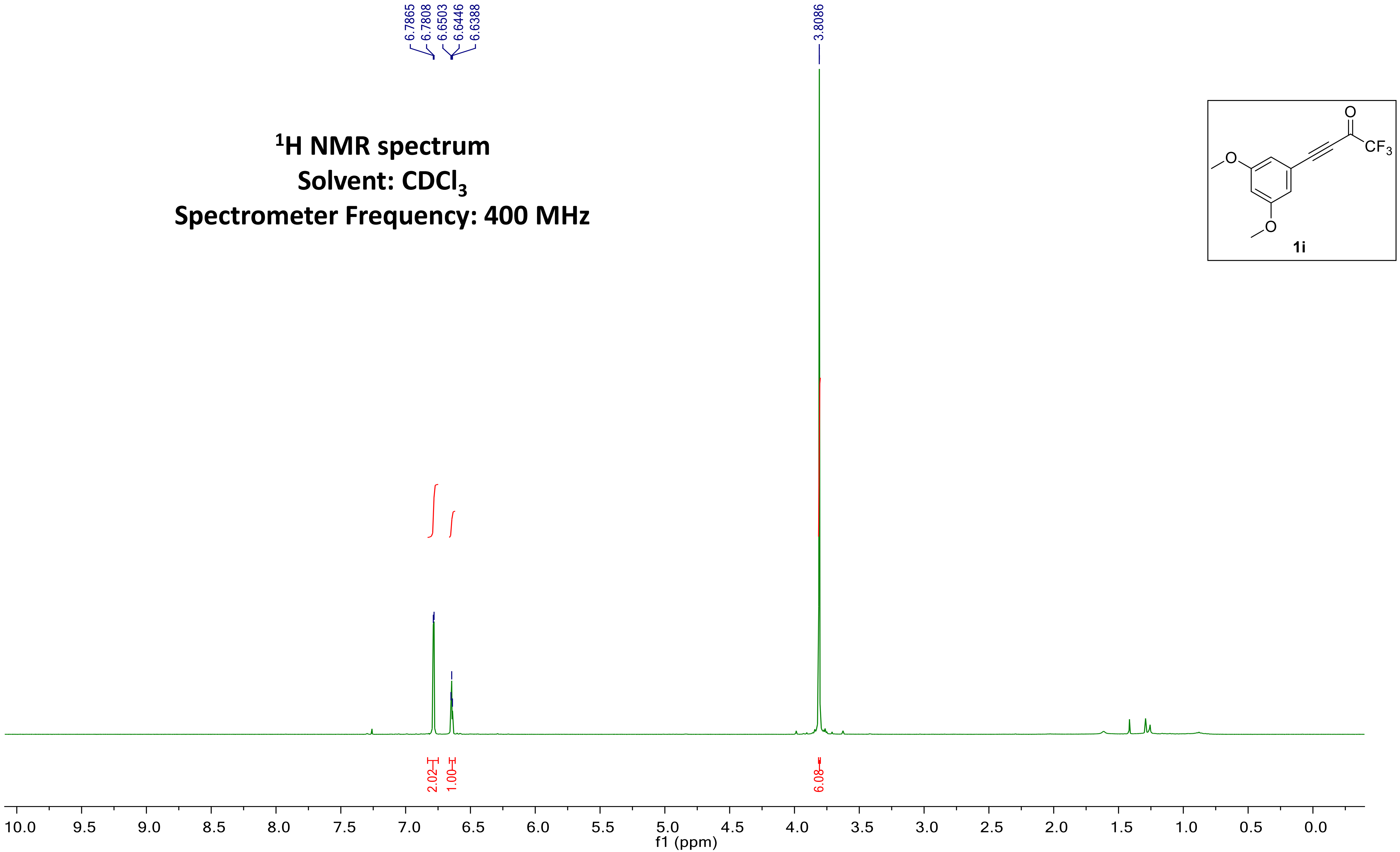
^{1}H NMR spectrum
Solvent: $CDCl_3$
Spectrometer Frequency: 400 MHz
6.7865
6.7808
6.6503
6.6446
6.6388
3.8086
O
CF_3
O
O
1i
2.02
1.00
6.08
10.0 9.5 9.0 8.5 8.0 7.5 7.0 6.5 6.0 5.5 5.0 4.5 4.0 3.5 3.0 2.5 2.0 1.5 1.0 0.5 0.0
f1 (ppm)

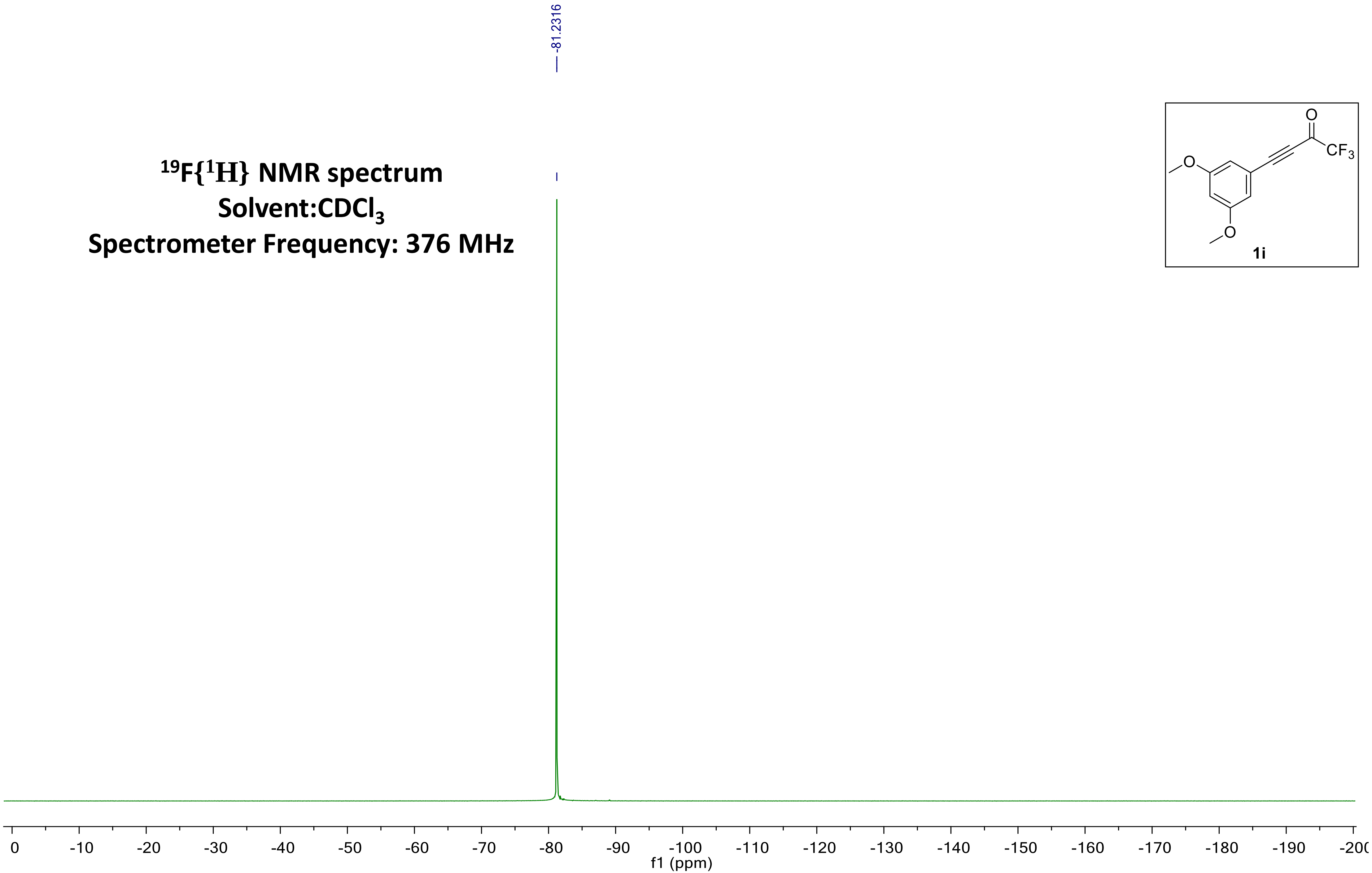
-81.2316
$^{19}F\{^{1}H\}$ NMR spectrum
Solvent:$CDCl_3$
Spectrometer Frequency: 376 MHz
0
-10
-20
-30
-40
-50
-60
-70
-80
-90
-100
-110
-120
-130
-140
-150
-160
-170
-180
-190
-200
f1 (ppm)
O
CF_3
O
O

1i

$^{13}C\ \{^{1}H\}$ NMR spectrum

Solvent: $CDCl_3$

Spectrometer Frequency: 100 MHz

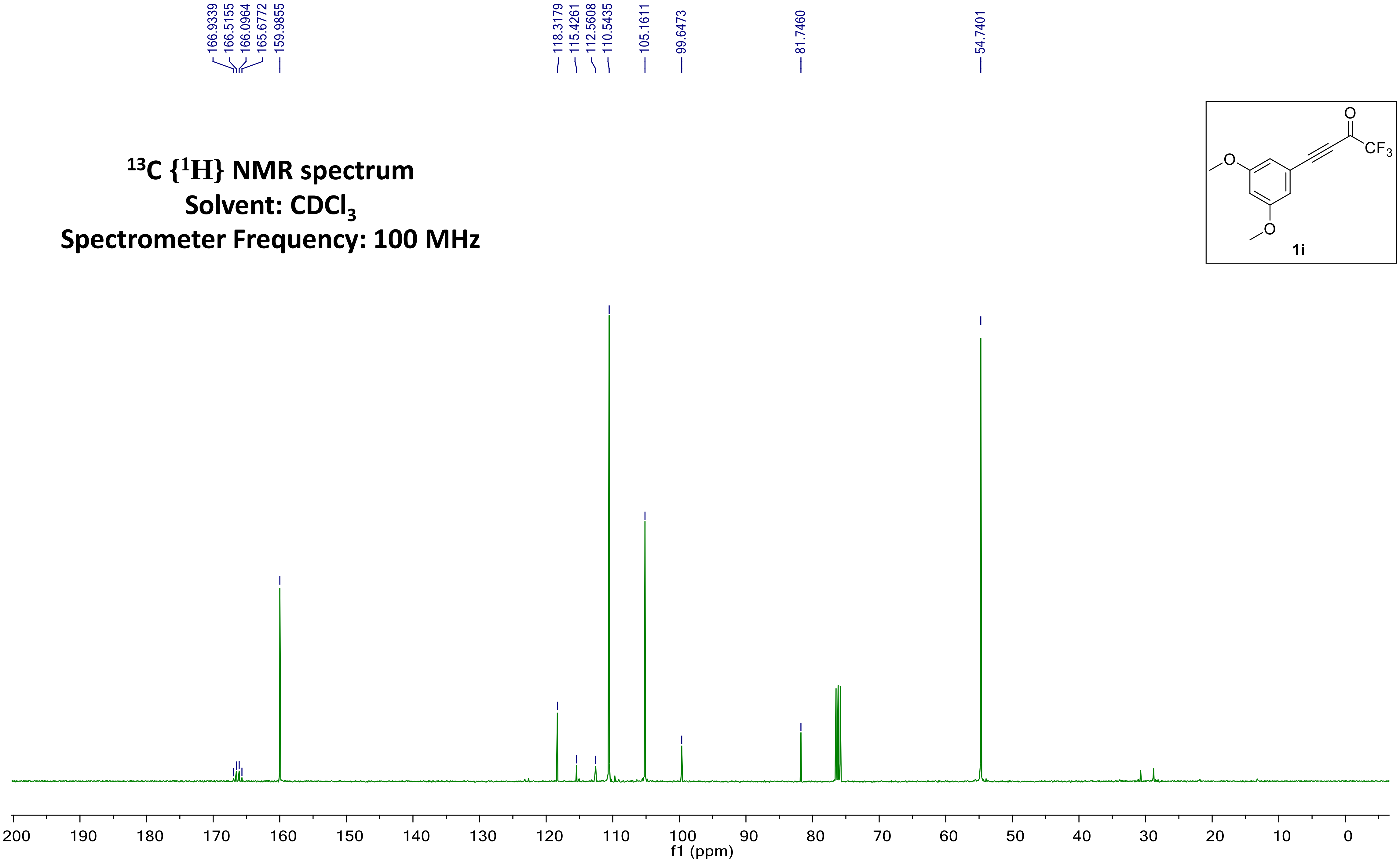

^{1}H NMR spectrum
Solvent: $CDCl_3$
Spectrometer Frequency: 400 MHz

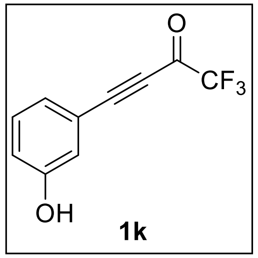

1k

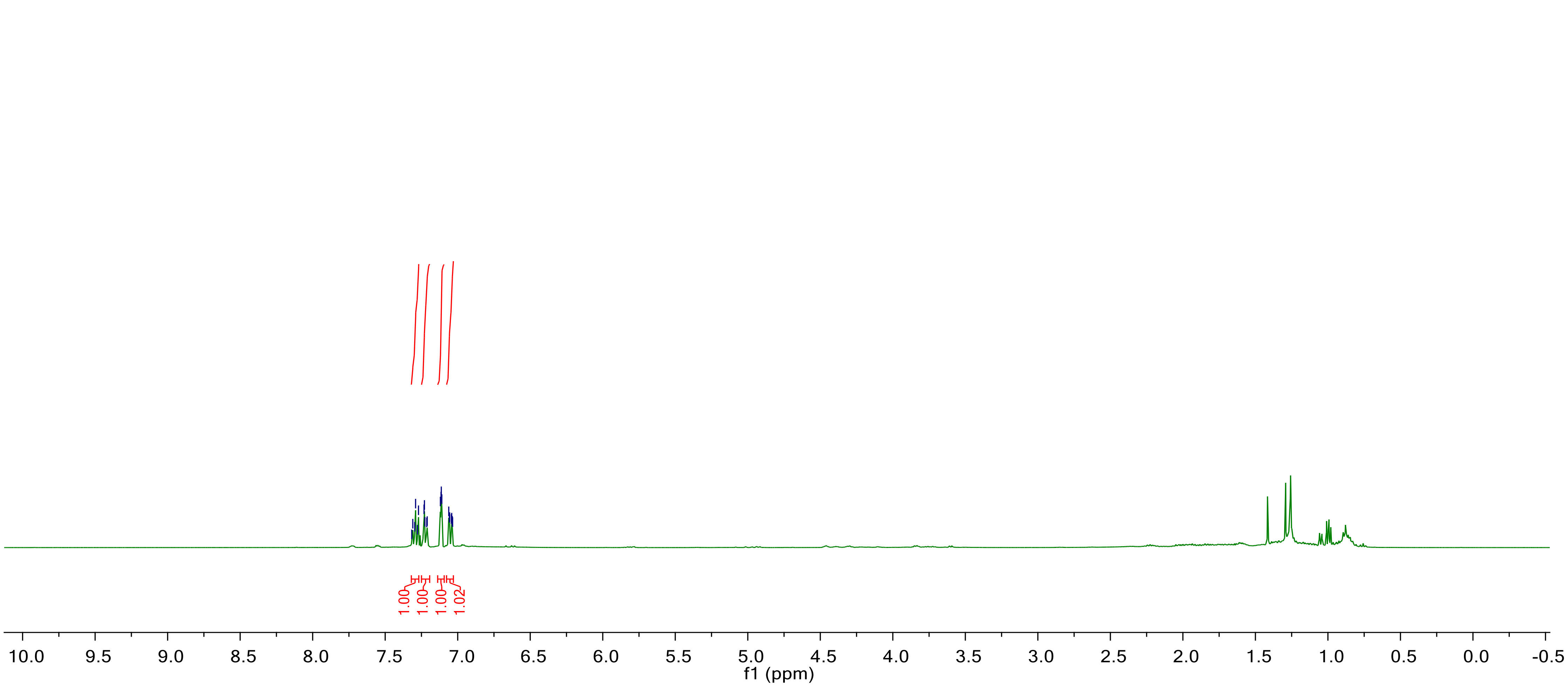

-77.8655

$^{19}F\{^{1}H\}$ NMR spectrum
Solvent:$CDCl_3$
Spectrometer Frequency: 376 MHz

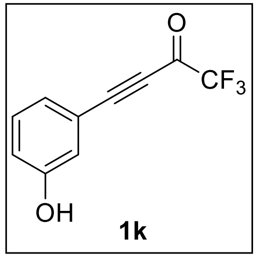

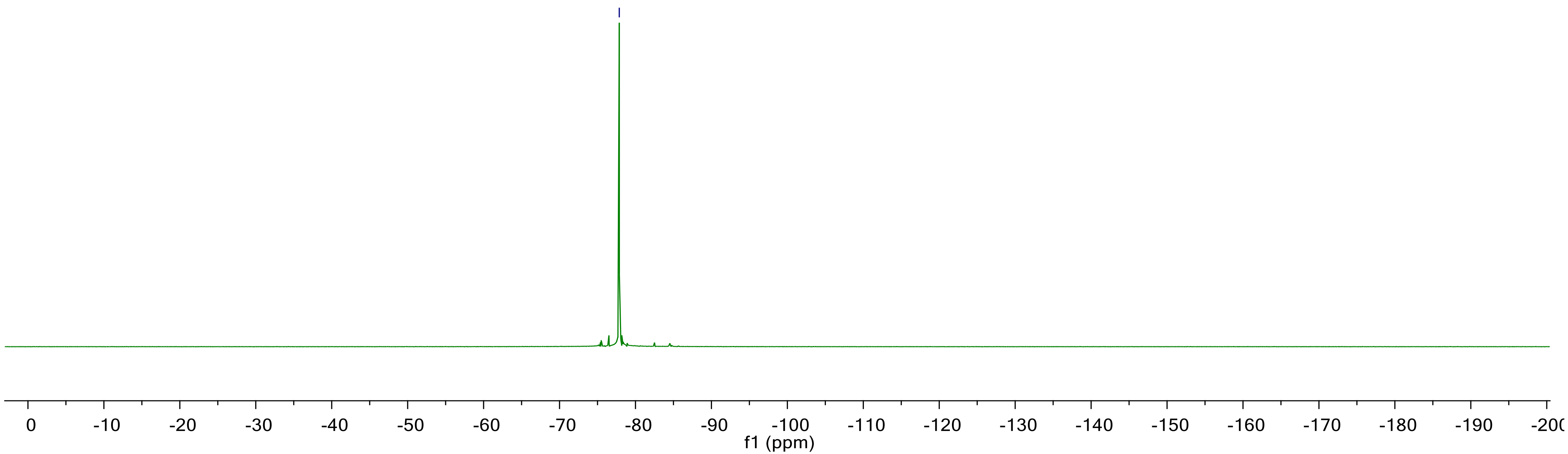

$^{13}C\ \{^1H\}$ NMR spectrum
Solvent: $CDCl_3$
Spectrometer Frequency: 100 MHz

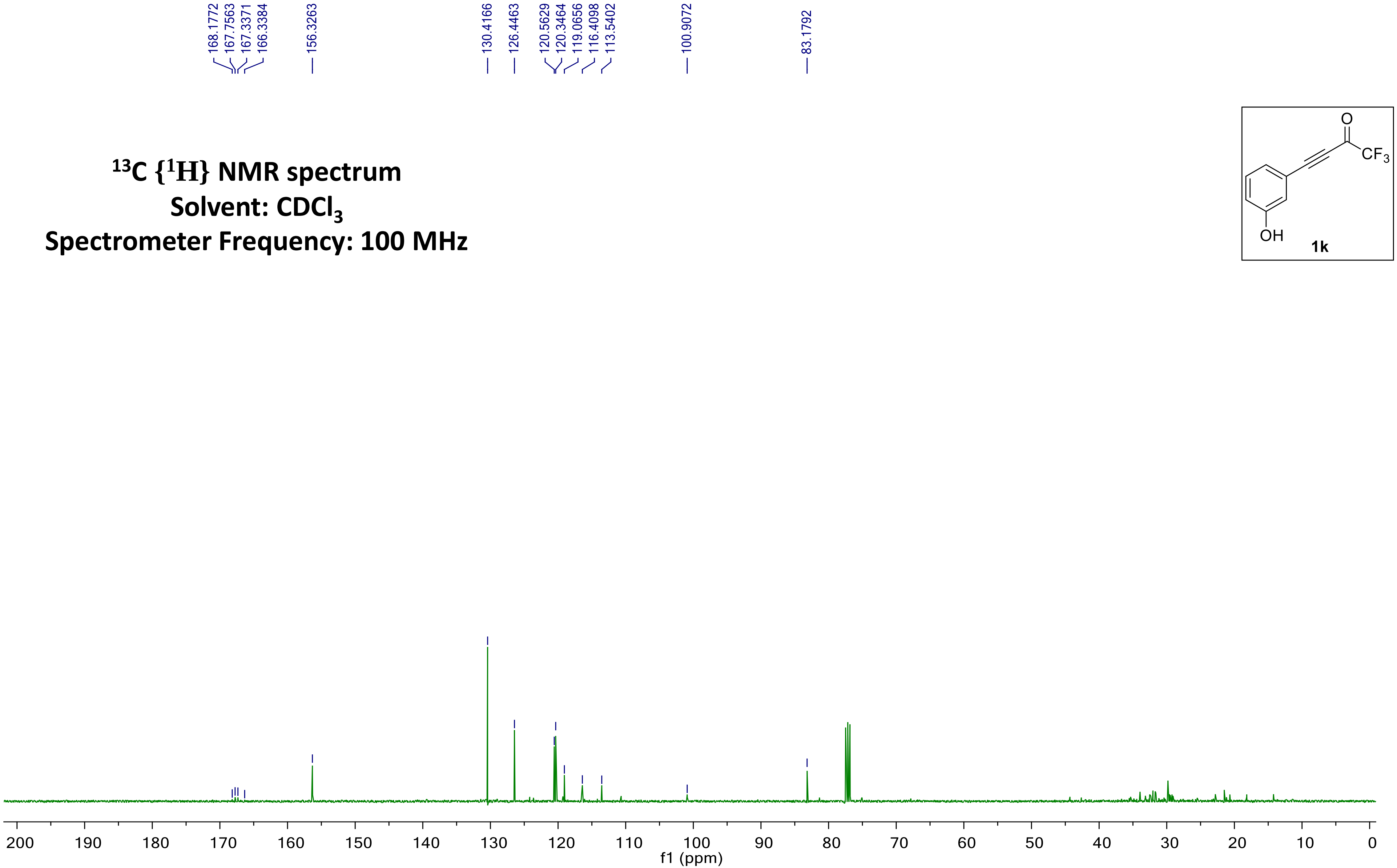

1k

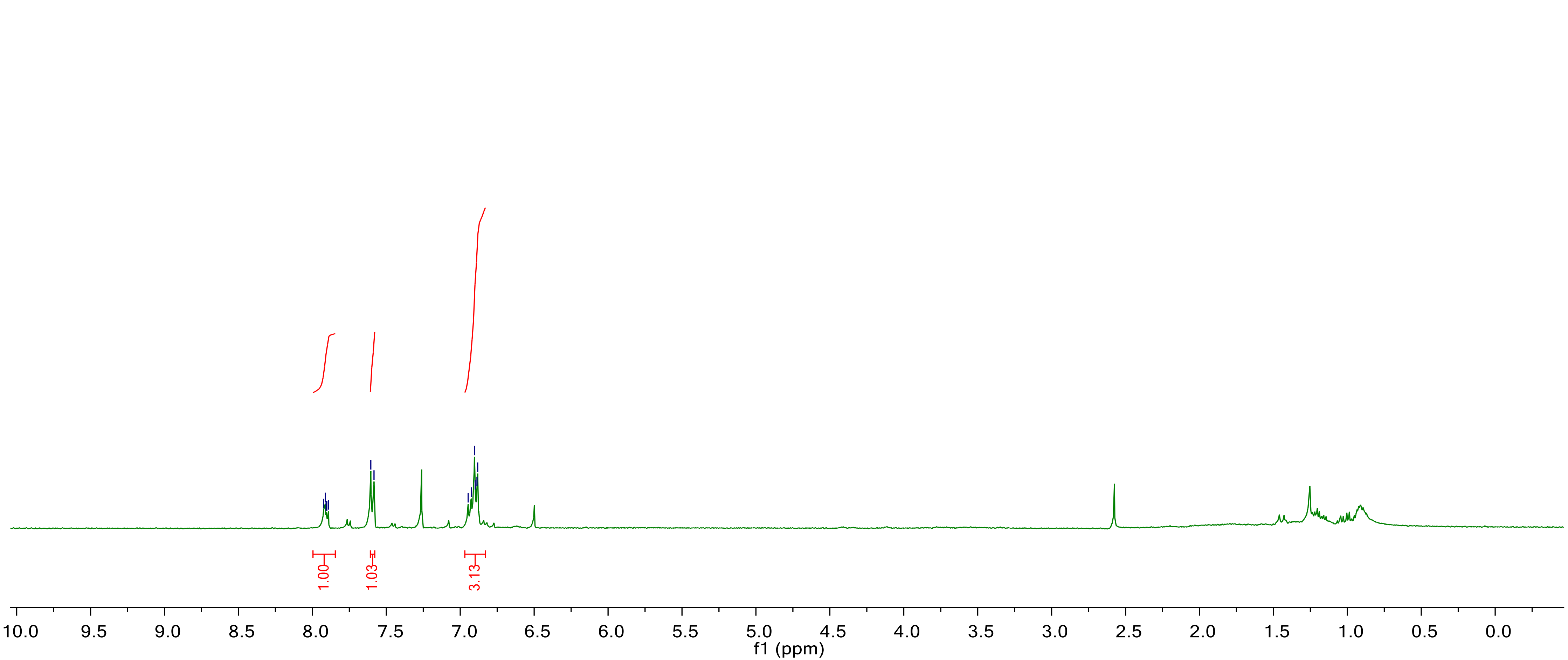

^{1}H NMR spectrum
Solvent: $CDCl_3$
Spectrometer Frequency: 400 MHz

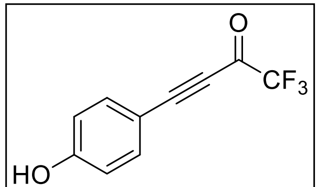

1l

$^{19}F\{^1H\}$ NMR spectrum
Solvent:$CDCl_3$
Spectrometer Frequency: 376 MHz

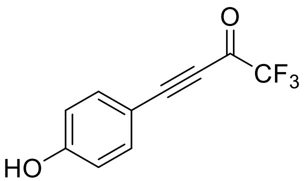

1l

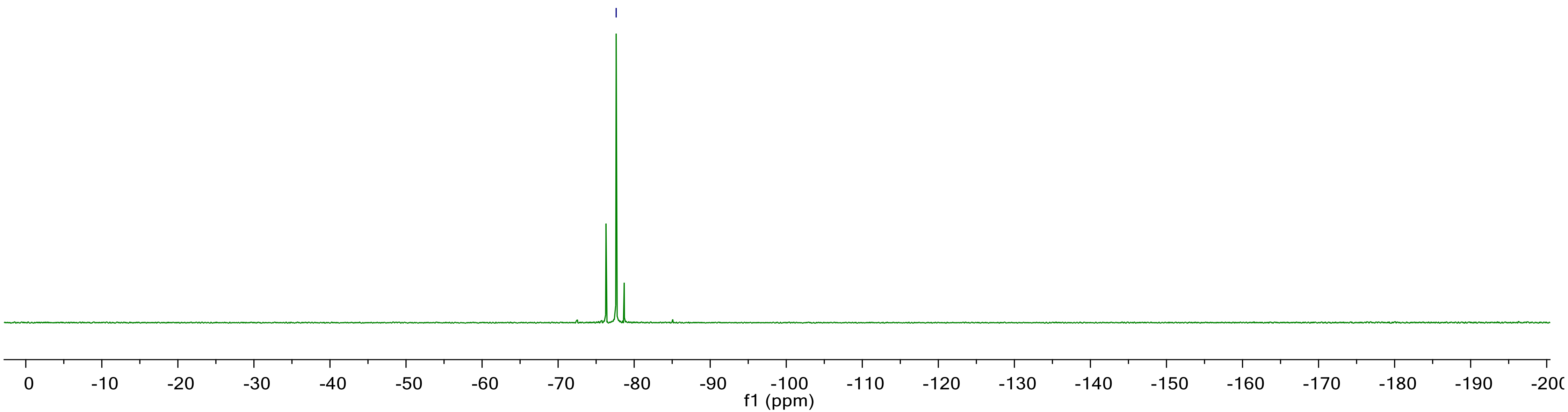

$^{13}C\ \{^{1}H\}$ NMR spectrum
Solvent: $CDCl_3$
Spectrometer Frequency: 100 MHz

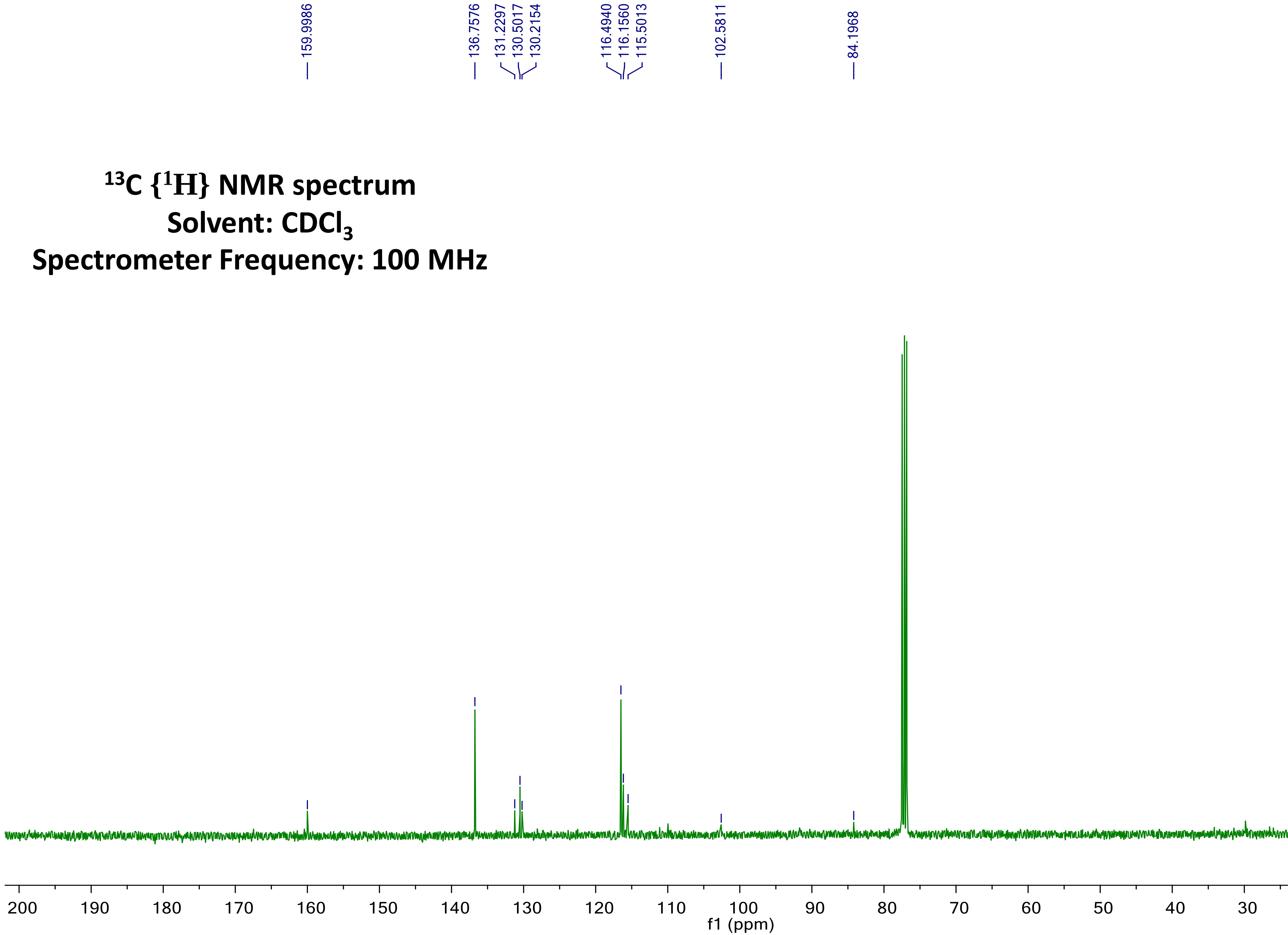

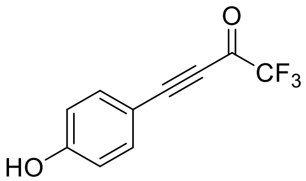

1l

^{1}H NMR spectrum
Solvent: $CDCl_3$
Spectrometer Frequency: 400 MHz

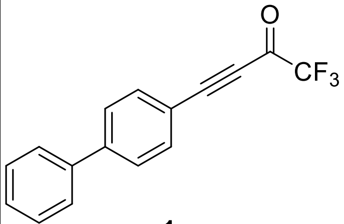

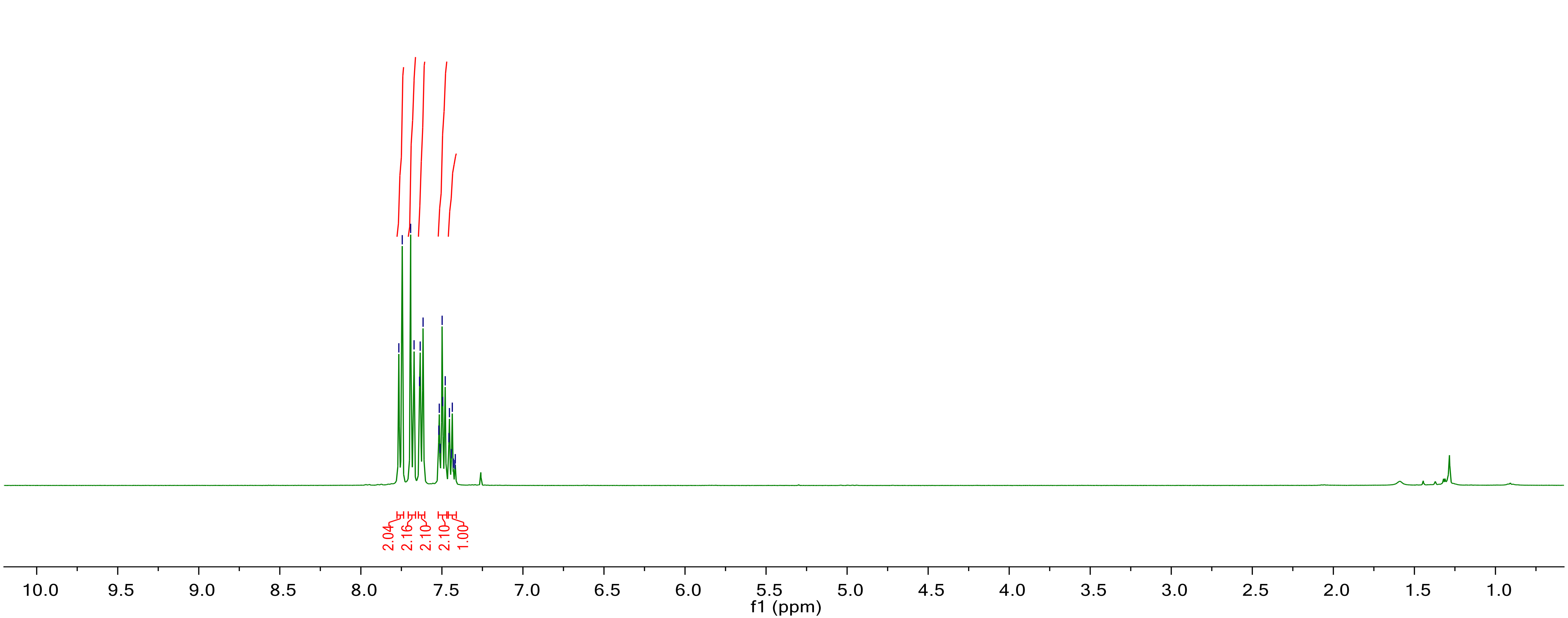

$^{19}F\{^{1}H\}$ NMR spectrum
Solvent:$CDCl_3$
Spectrometer Frequency: 376 MHz

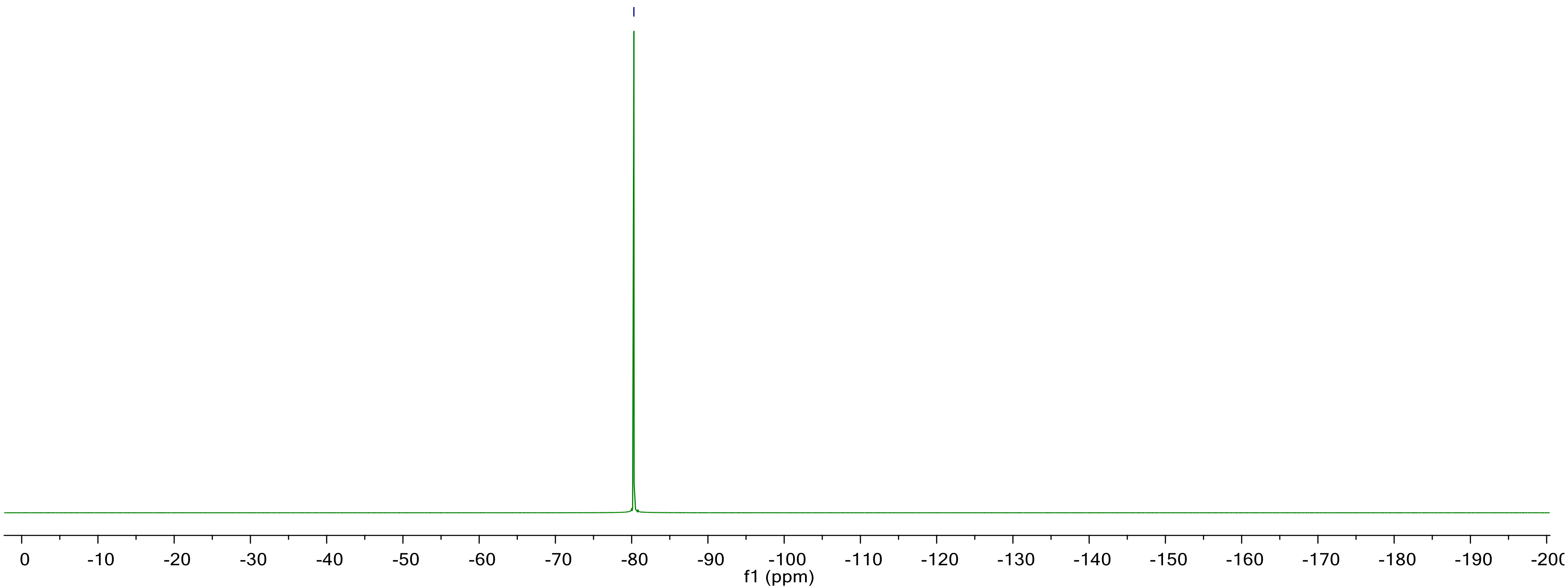

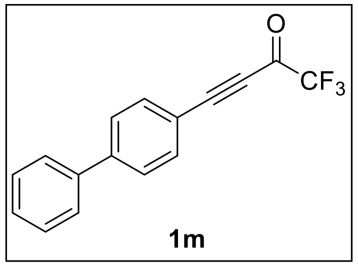

1m

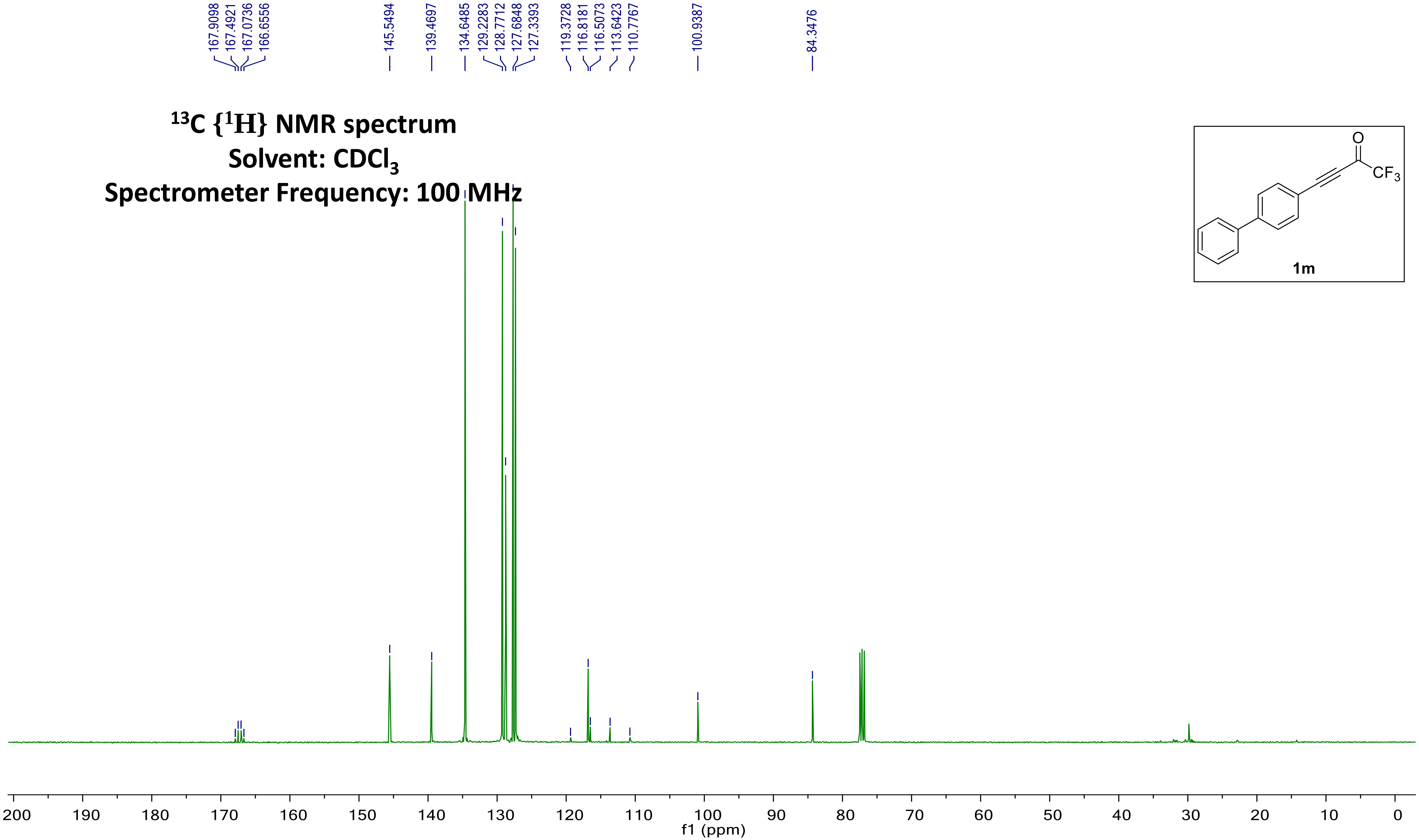
167.9098
167.4921
167.0736
166.6556
145.5494
139.4697
134.6485
129.2283
128.7712
127.6848
127.3393
119.3728
116.8181
116.5073
113.6423
110.7767
100.9387
84.3476
^{13}C {^{1}H} NMR spectrum
Solvent: $CDCl_3$
Spectrometer Frequency: 100 MHz
O
CF_3
1m
200 190 180 170 160 150 140 130 120 110 100 90 80 70 60 50 40 30 20 10 0
f1 (ppm)

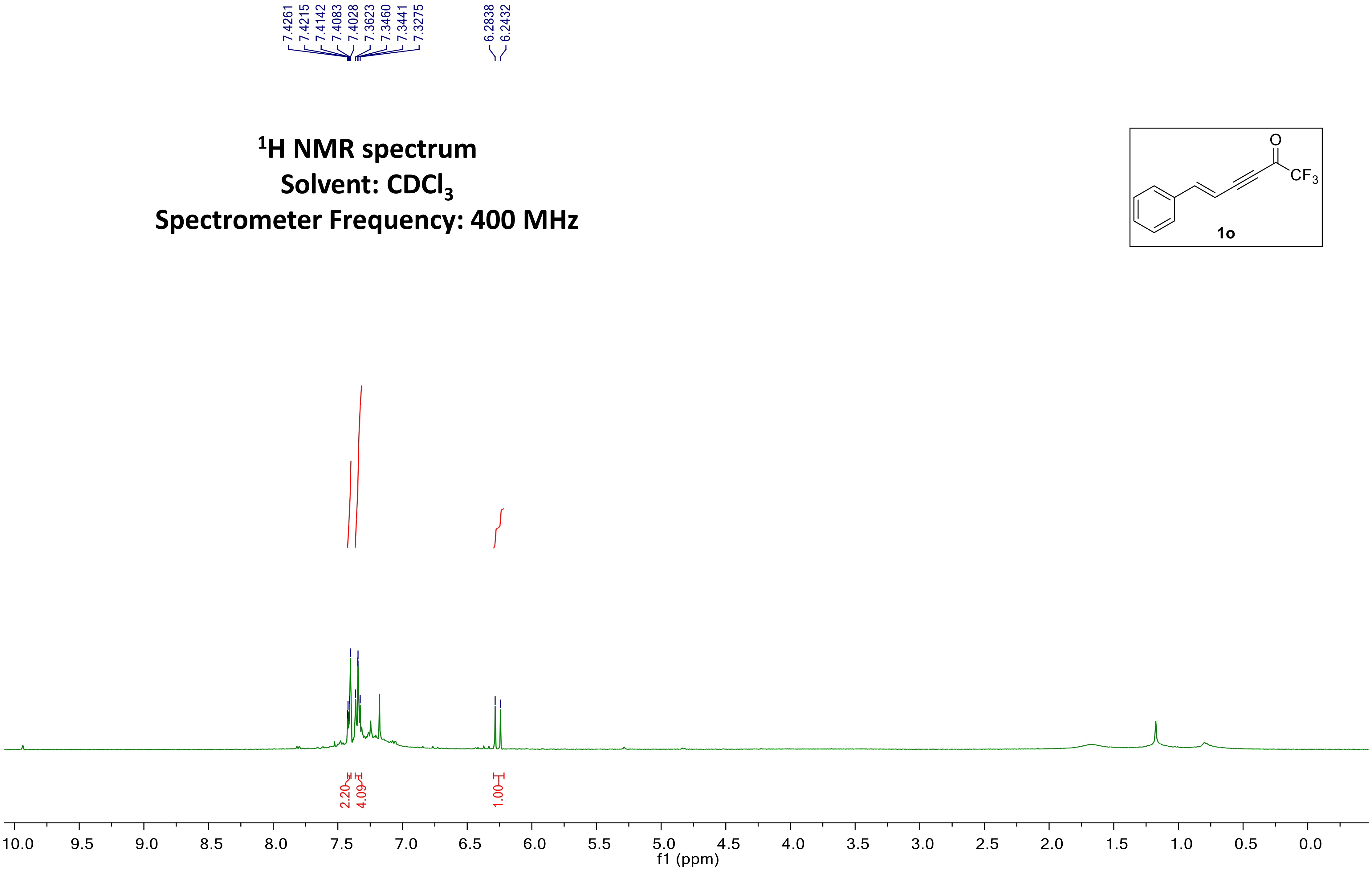

^{1}H NMR spectrum
Solvent: $CDCl_3$
Spectrometer Frequency: 400 MHz

1o

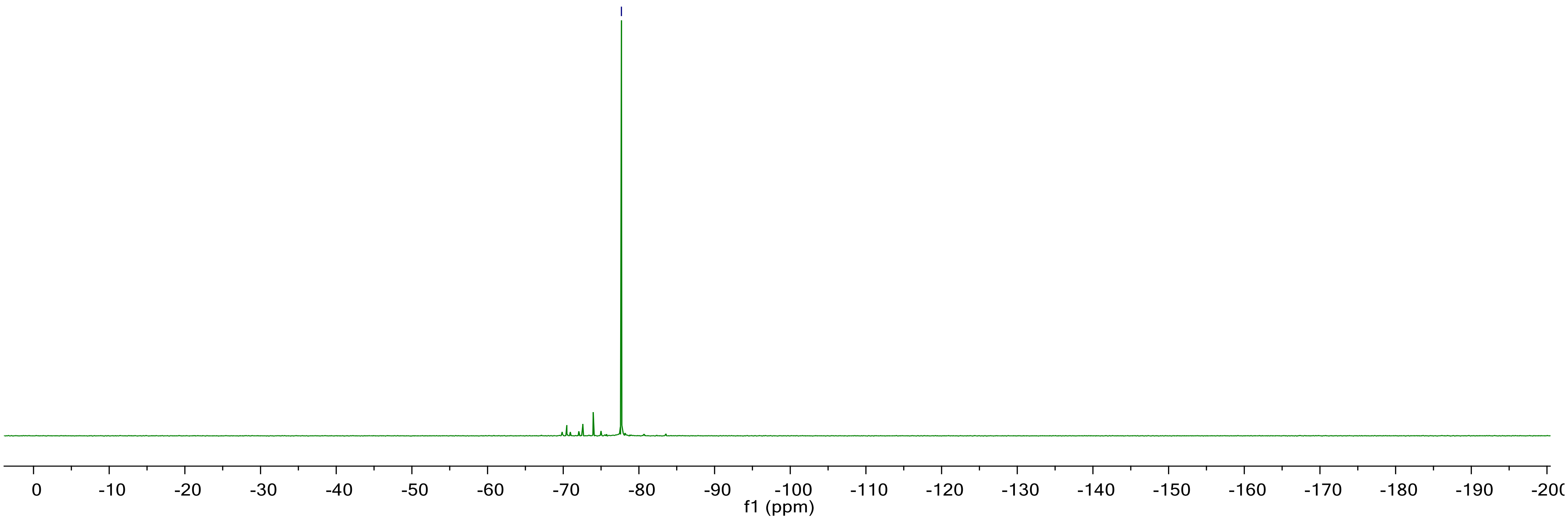

$^{19}F\{^1H\}$ NMR spectrum
Solvent:$CDCl_3$
Spectrometer Frequency: 376 MHz

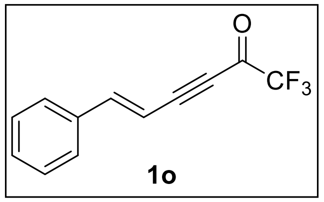

1o

$^{13}C\ \{^{1}H\}$ NMR spectrum
Solvent: $CDCl_3$
Spectrometer Frequency: 100 MHz

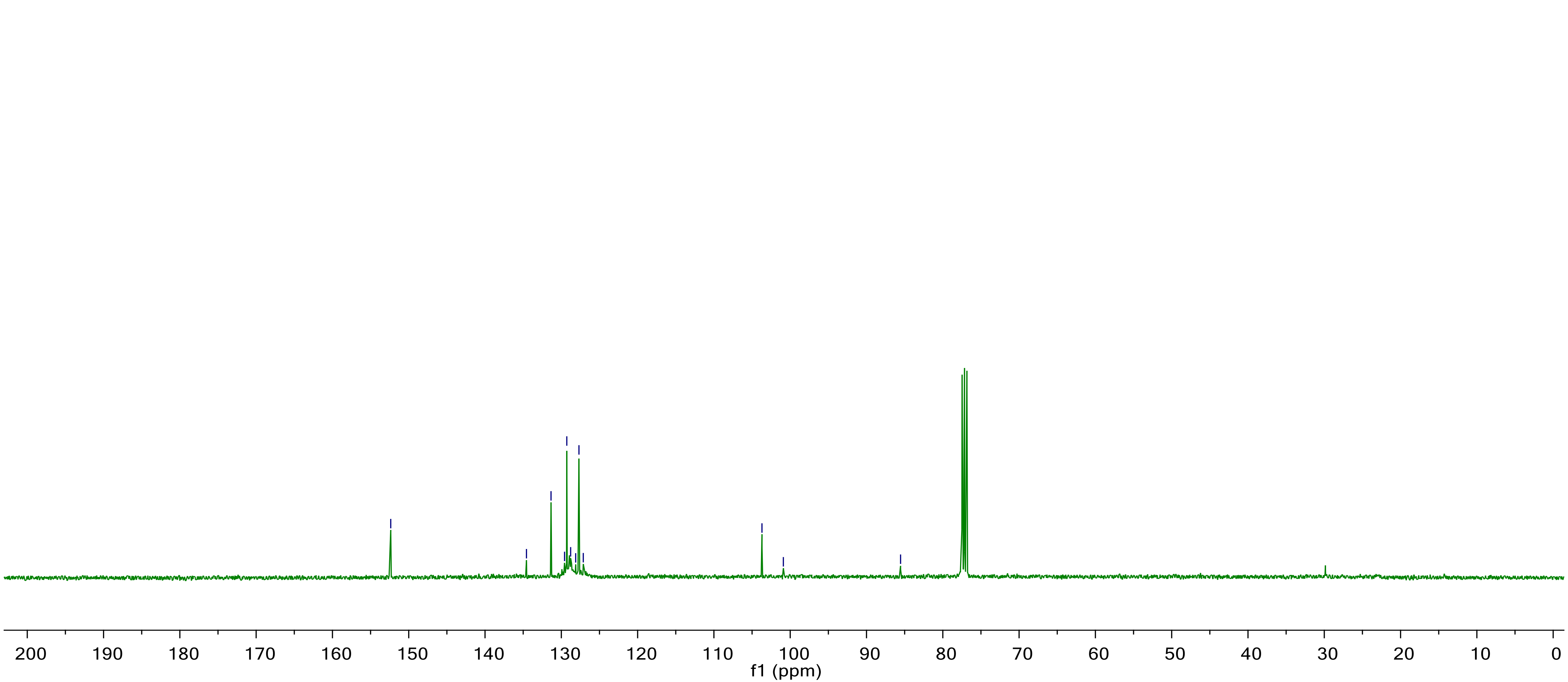

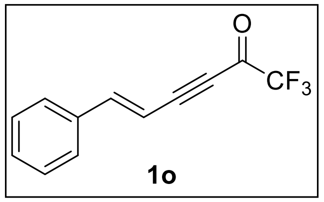

1o

^{1}H NMR spectrum
Solvent: $CDCl_3$
Spectrometer Frequency: 400 MHz

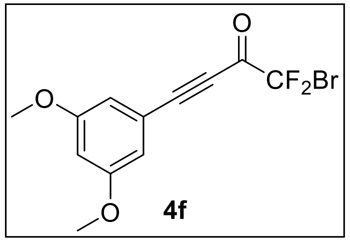

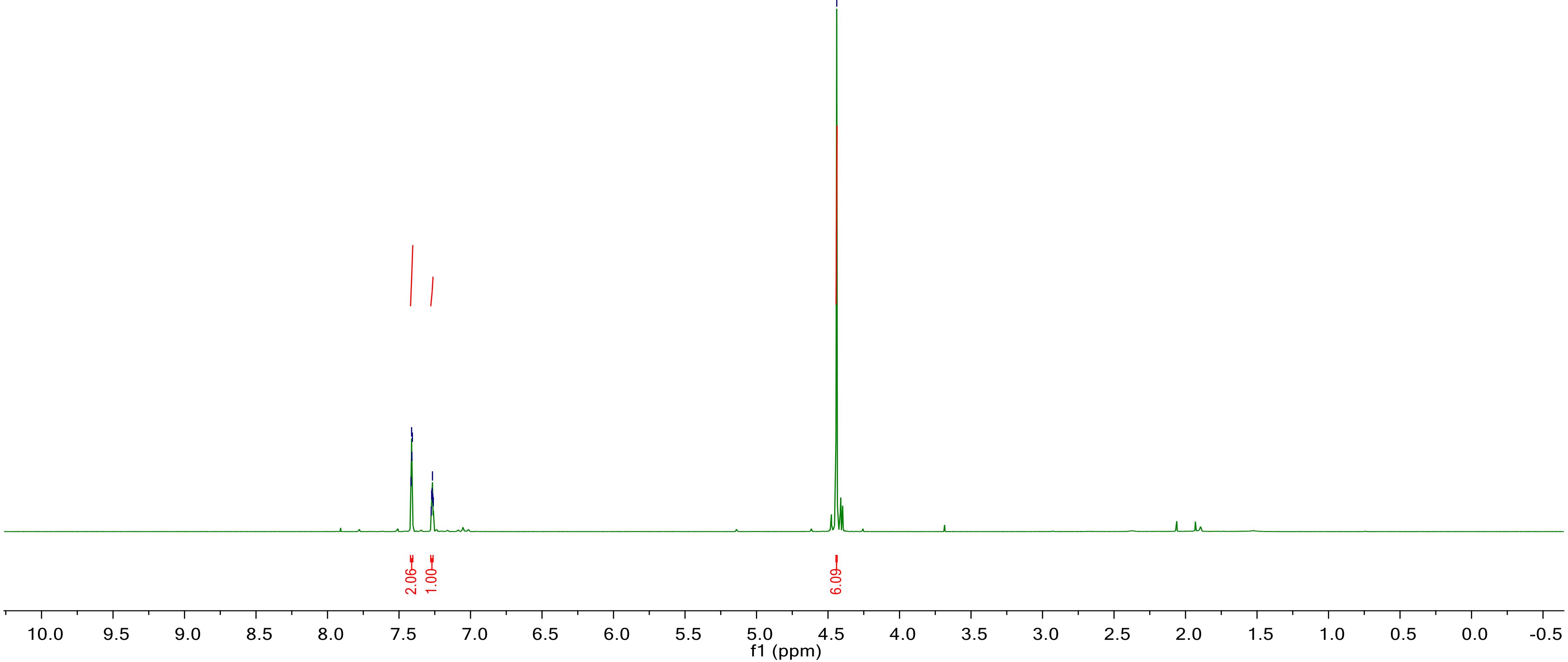

$^{19}F\{^{1}H\}$ NMR spectrum
Solvent:$CDCl_3$
Spectrometer Frequency: 376 MHz

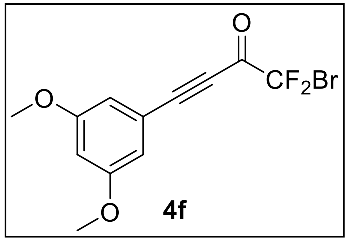

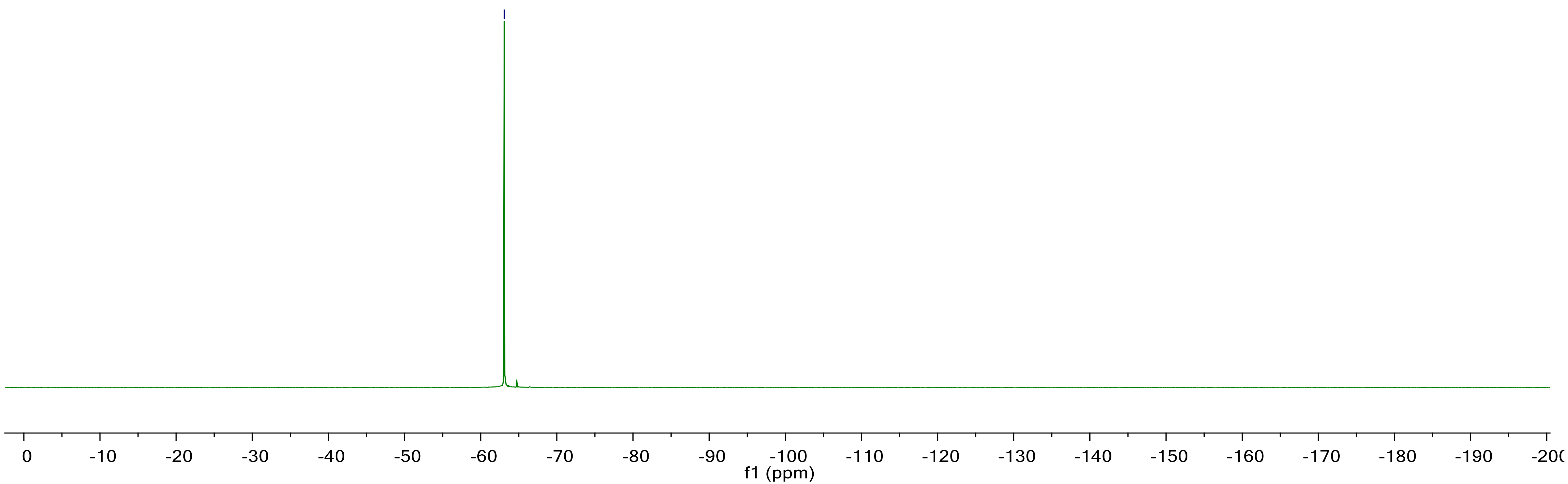

^{13}C {^{1}H} NMR spectrum
Solvent: $CDCl_3$
Spectrometer Frequency: 100 MHz

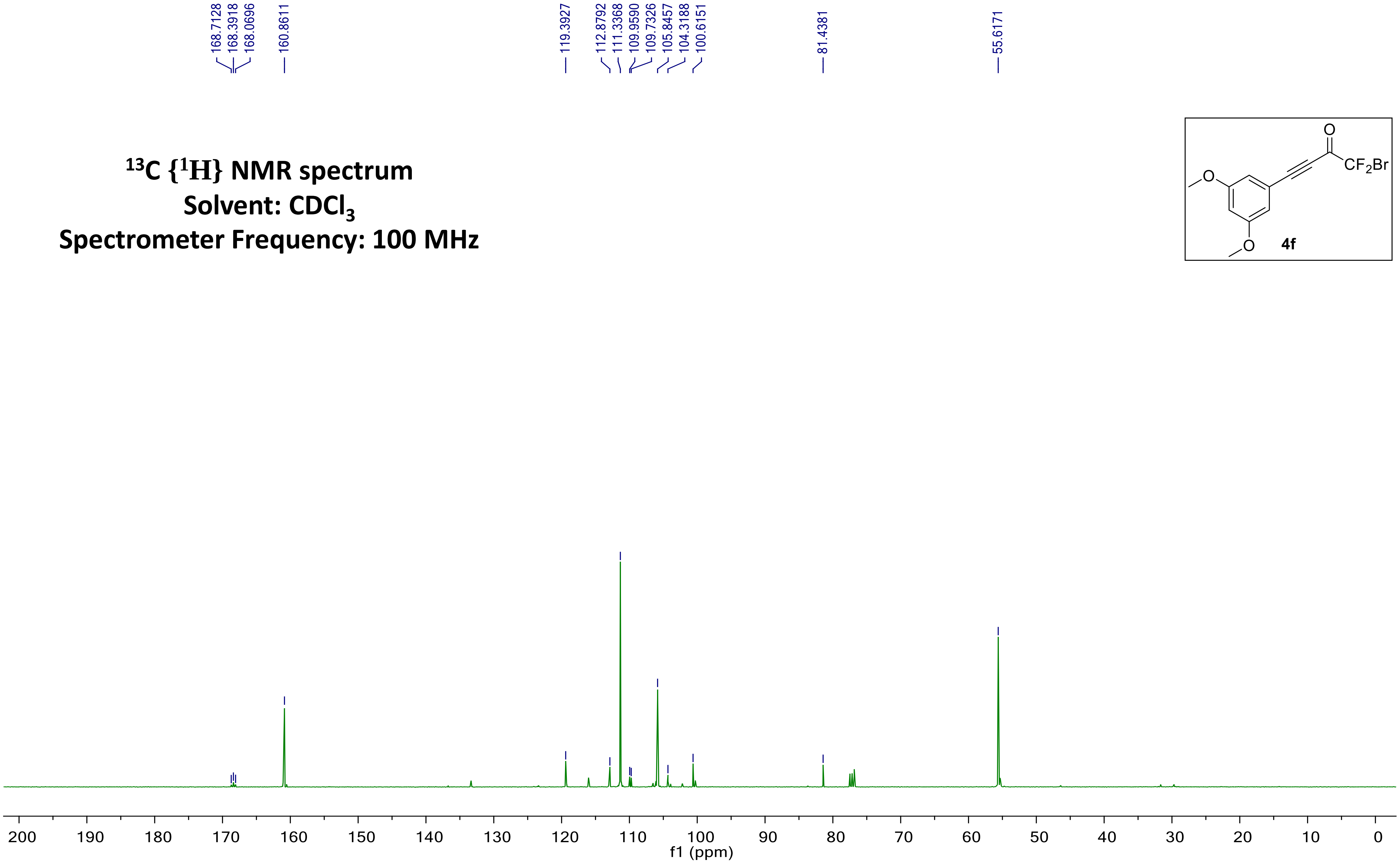

^{1}H NMR spectrum
Solvent: $CDCl_3$
Spectrometer Frequency: 400 MHz

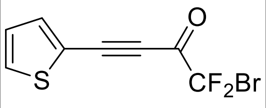
4g

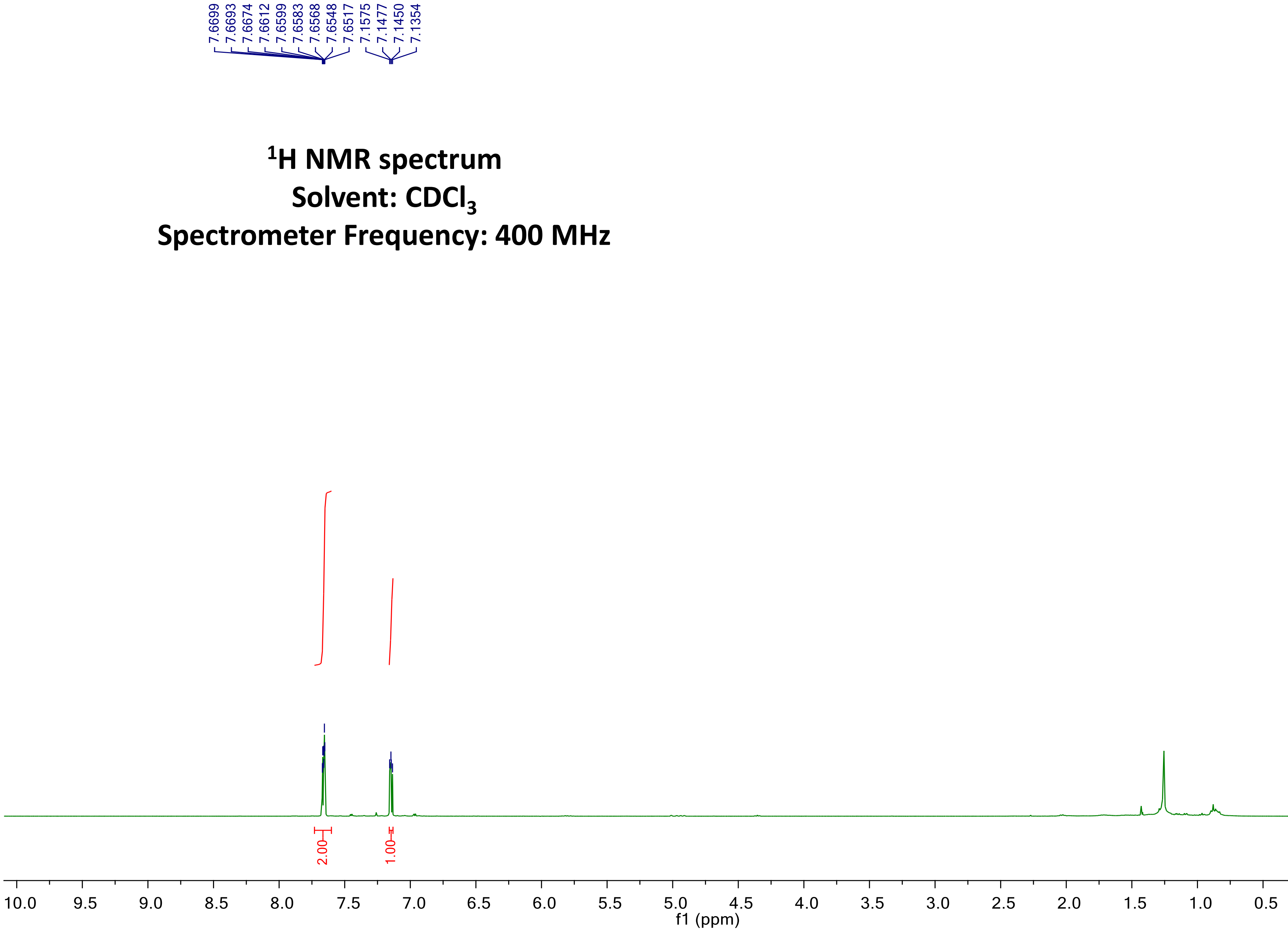

$^{19}F\{^1H\}$ NMR spectrum
Solvent:$CDCl_3$
Spectrometer Frequency: 376 MHz

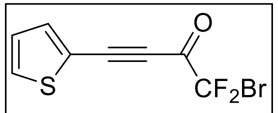

4g

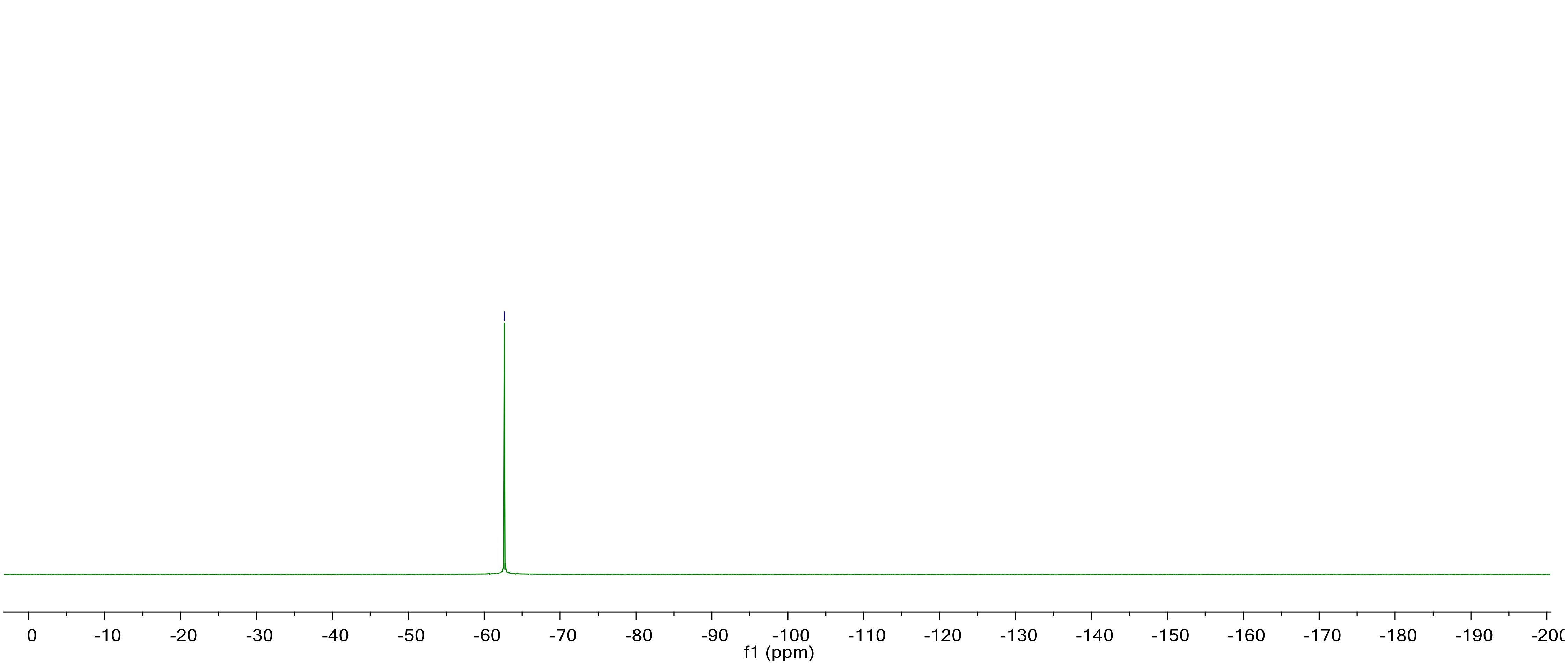

^{13}C {^{1}H} NMR spectrum
Solvent: $CDCl_3$
Spectrometer Frequency: 100 MHz

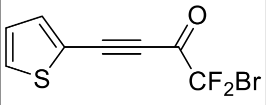

4g

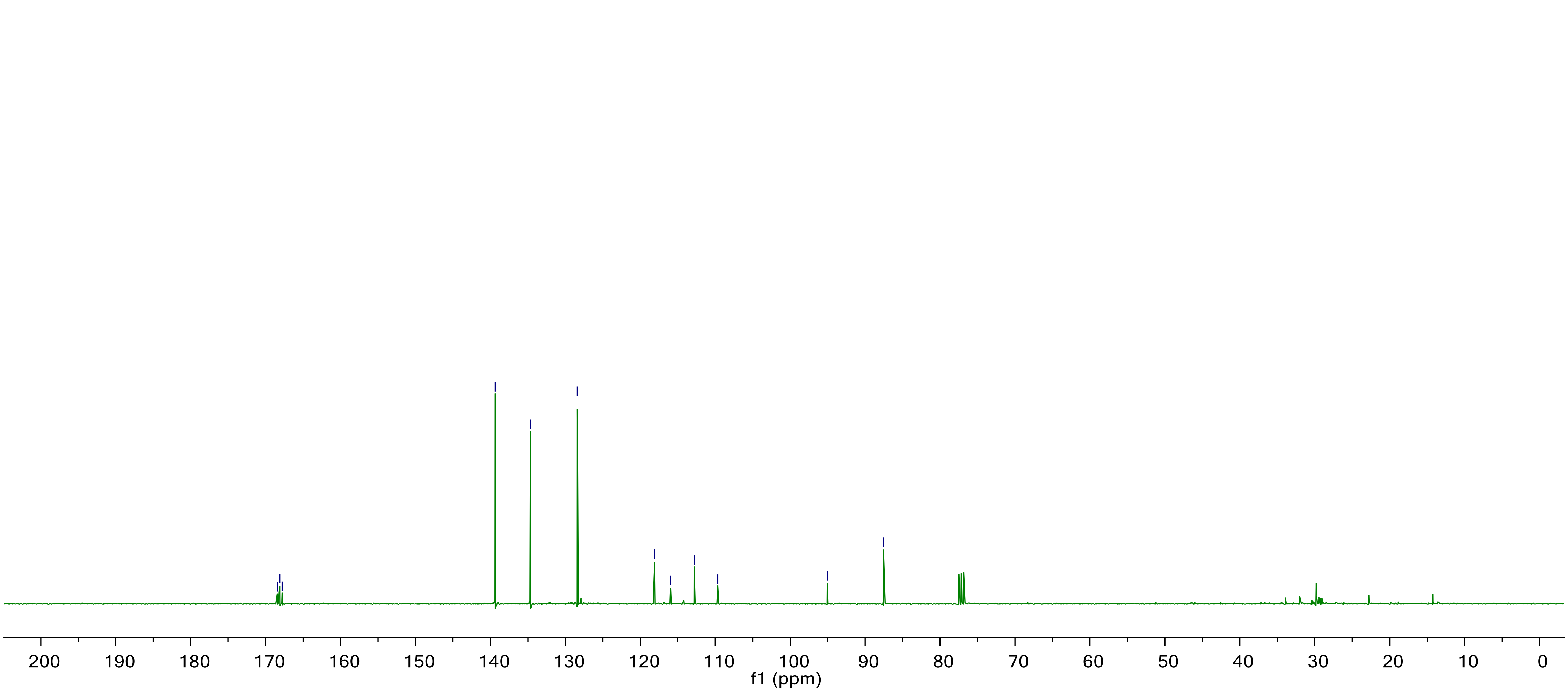

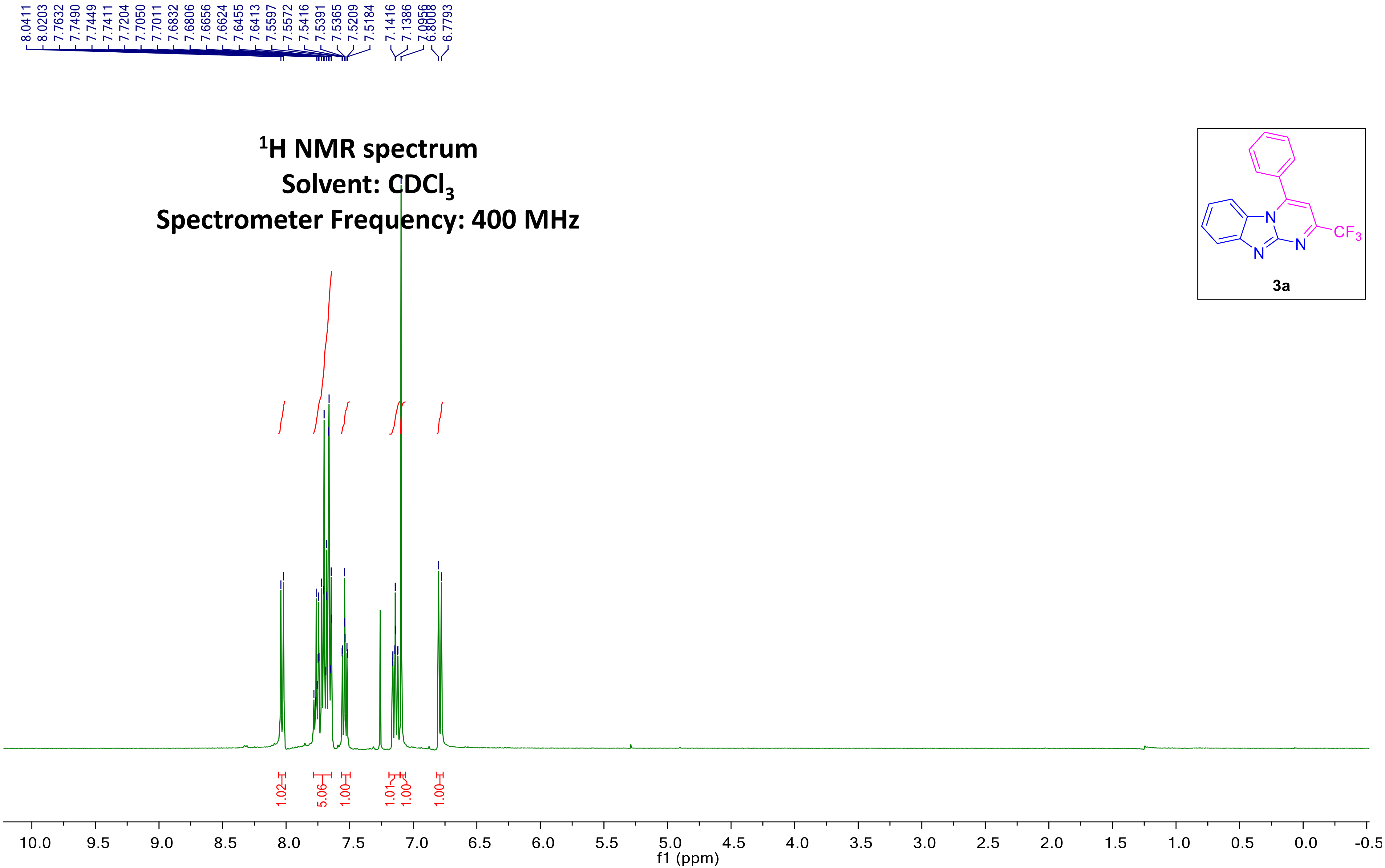

8.0411
8.0203
7.7632
7.7490
7.7449
7.7411
7.7204
7.7050
7.7011
7.6832
7.6806
7.6656
7.6624
7.6455
7.6413
7.5597
7.5572
7.5416
7.5391
7.5365
7.5209
7.5184
7.1416
7.1386
7.0956
6.8008
6.7793
^{1}H NMR spectrum
Solvent: $CDCl_3$
Spectrometer Frequency: 400 MHz
N
N
N
CF_3
3a
1.02
5.06
1.00
1.01
1.00
1.00
10.0 9.5 9.0 8.5 8.0 7.5 7.0 6.5 6.0 5.5 5.0 4.5 4.0 3.5 3.0 2.5 2.0 1.5 1.0 0.5 0.0 -0.5
f1 (ppm)

$^{19}F\{^{1}H\}$ NMR spectrum
Solvent:$CDCl_3$
Spectrometer Frequency: 376 MHz

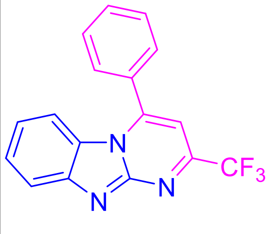

3a

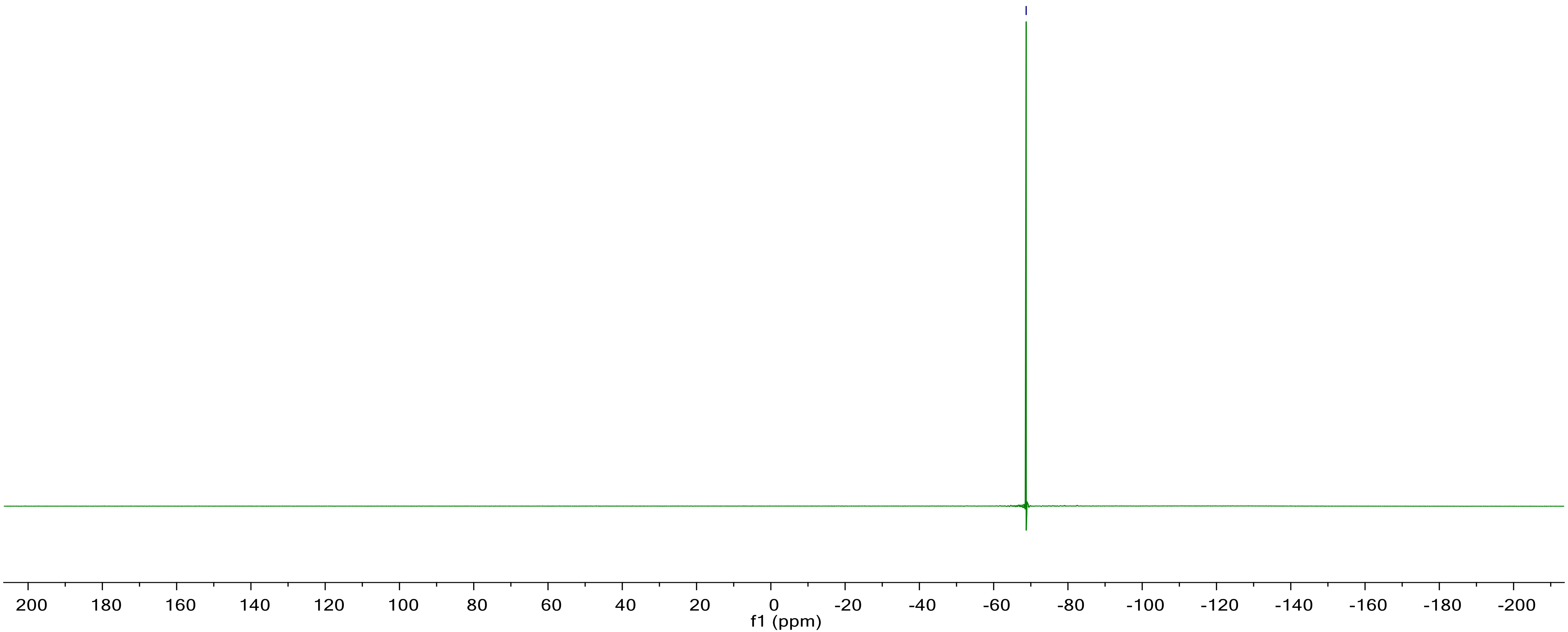

^{13}C {^{1}H} NMR spectrum
Solvent: $CDCl_3$
Spectrometer Frequency: 100 MHz

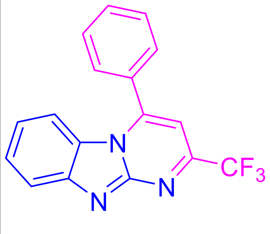

3a

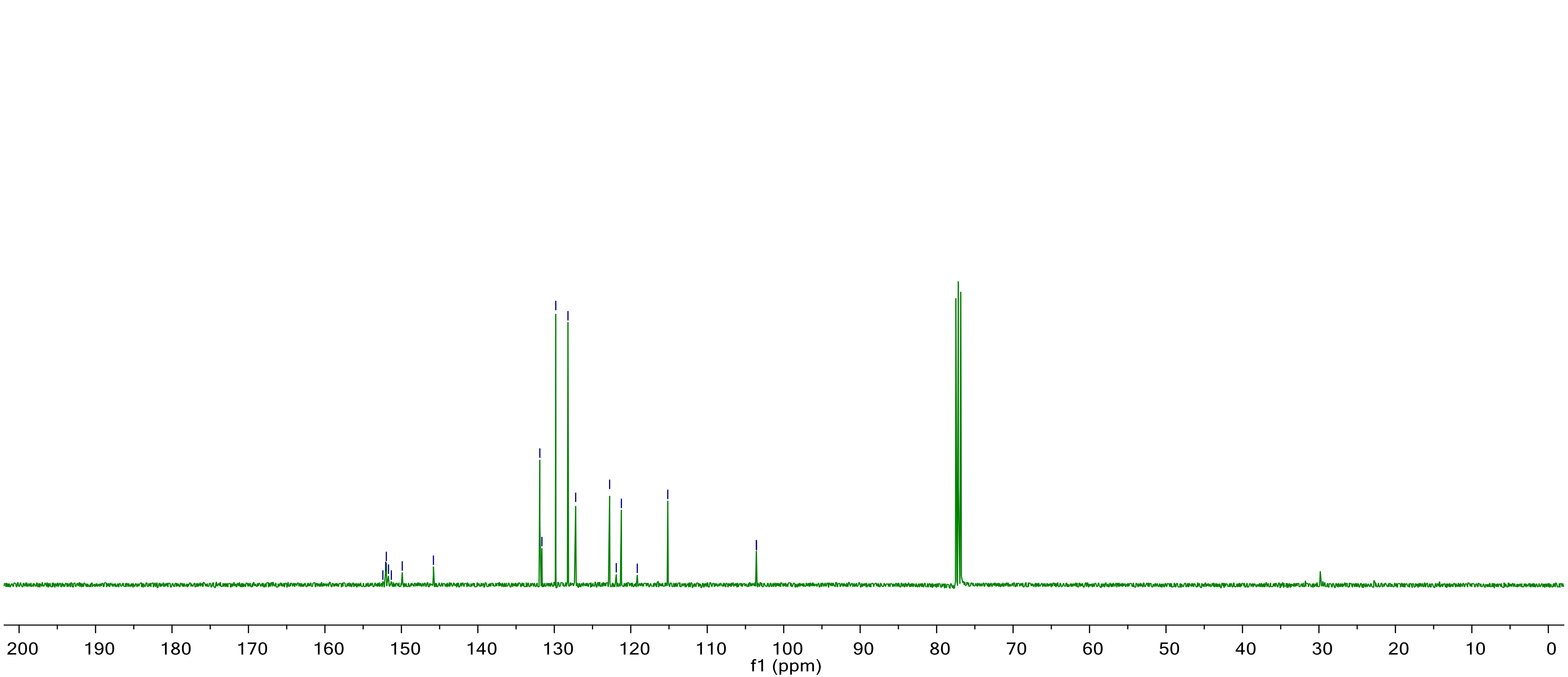

^{1}H NMR spectrum
Solvent: $CDCl_3$
Spectrometer Frequency: 400 MHz

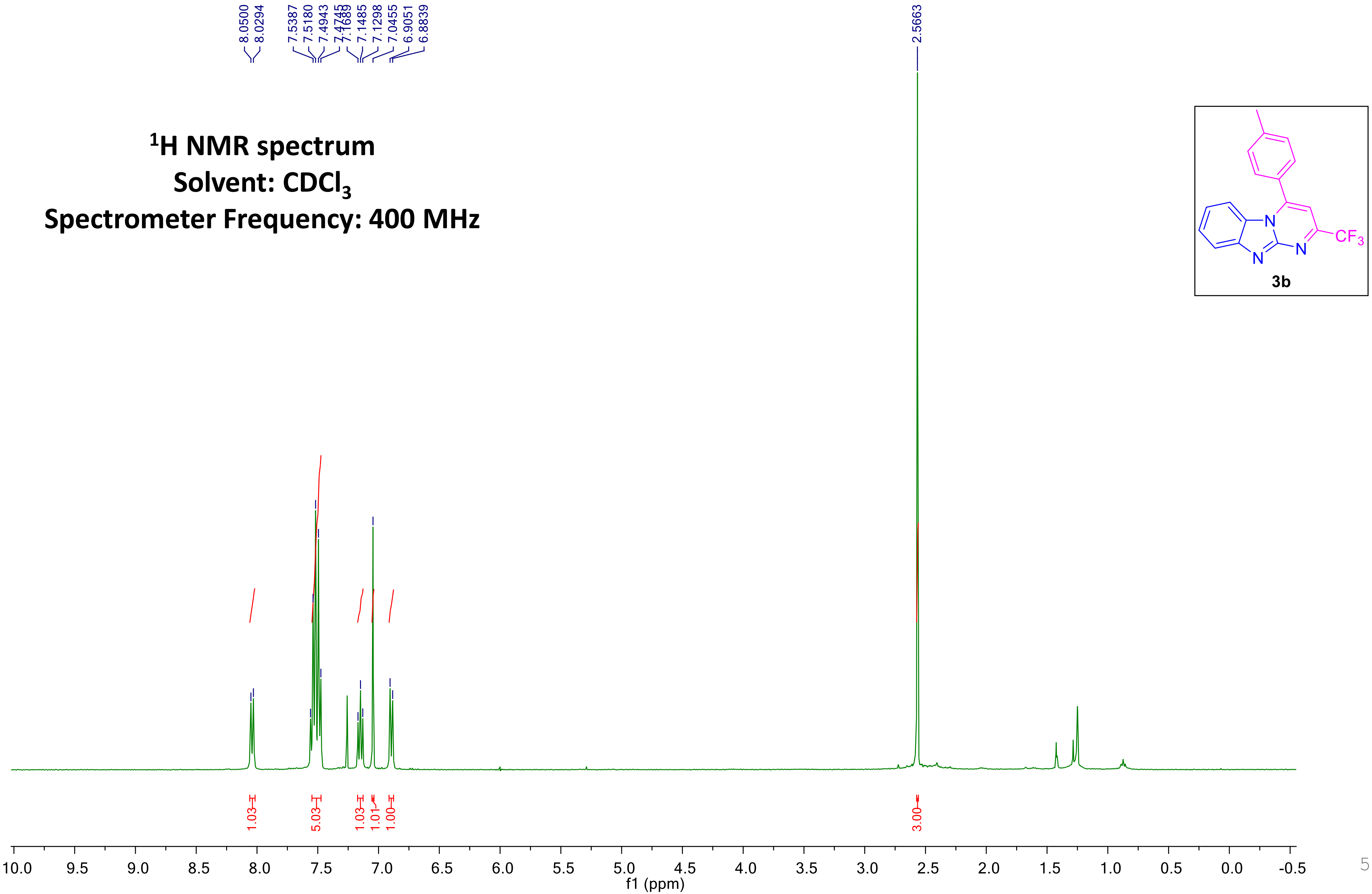

3b

$^{19}F\{^{1}H\}$ NMR spectrum
Solvent:$CDCl_3$
Spectrometer Frequency: 376 MHz

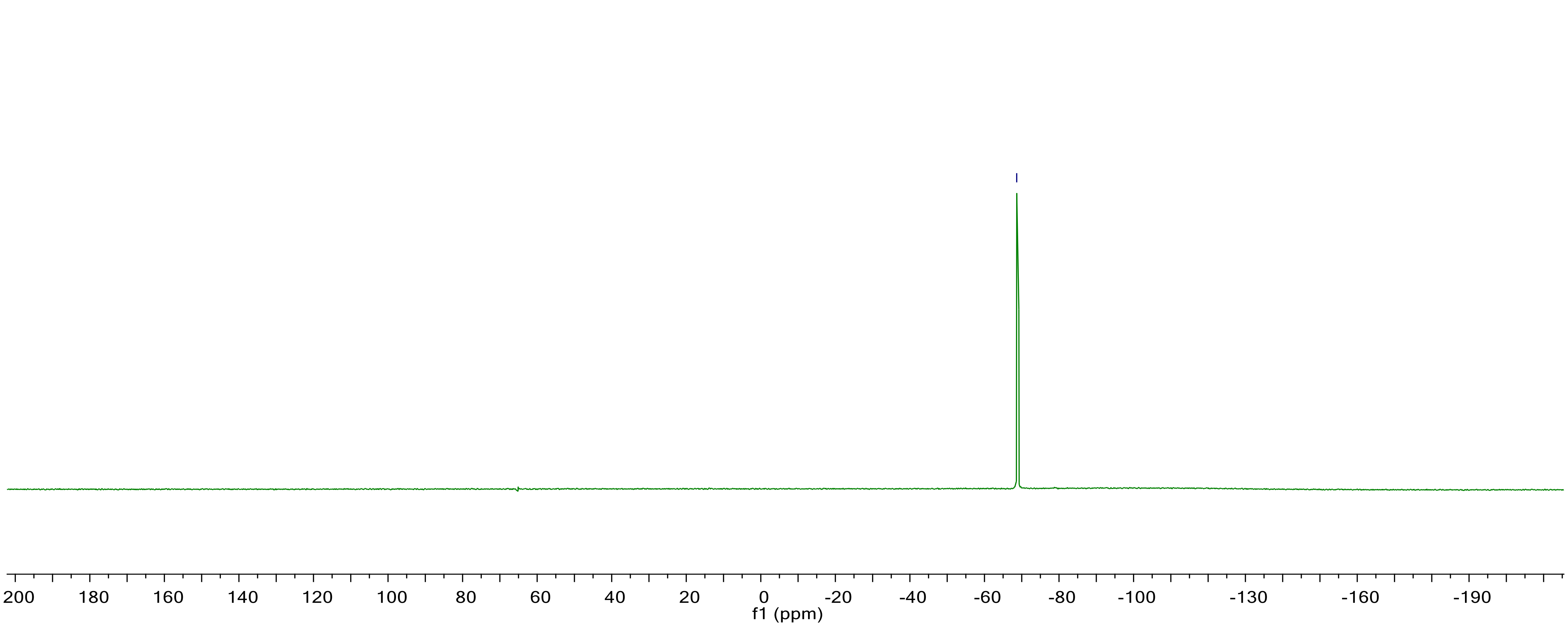

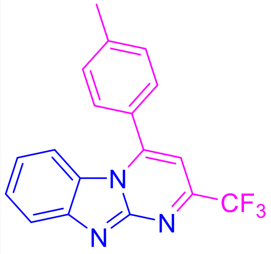

3b

^{13}C {^{1}H} NMR spectrum
Solvent: $CDCl_3$
Spectrometer Frequency: 100 MHz

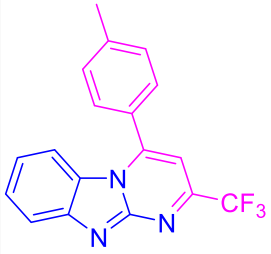

3b

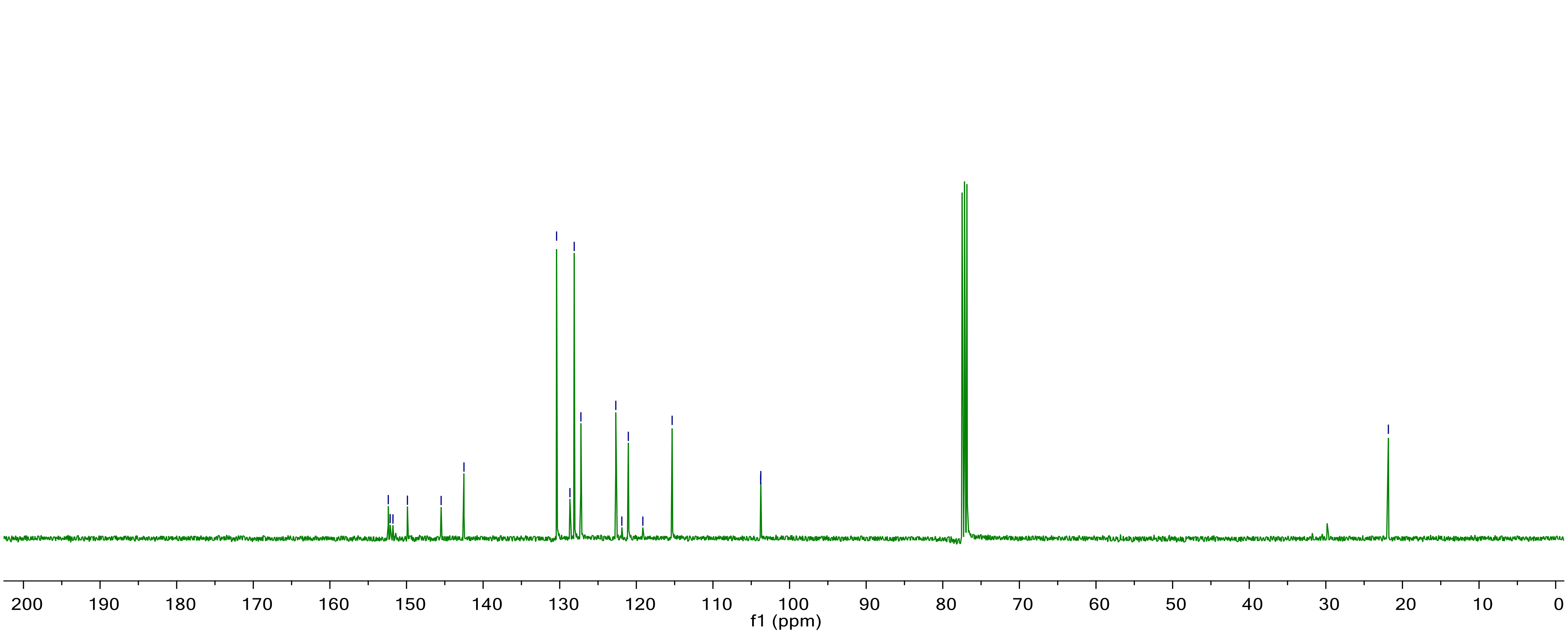

^{1}H NMR spectrum
Solvent: $CDCl_3$
Spectrometer Frequency: 400 MHz

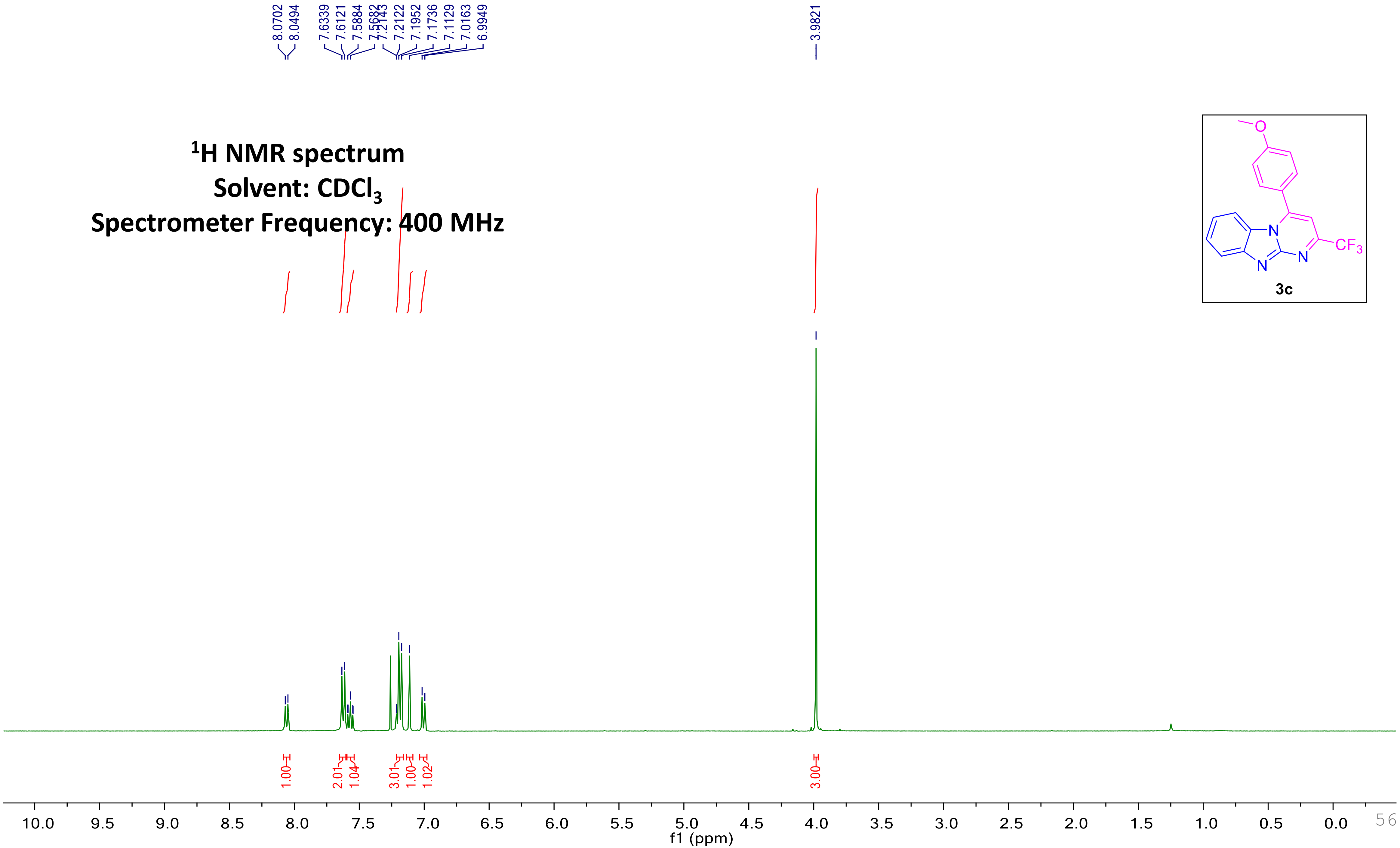

3c

$^{19}F\{^1H\}$ NMR spectrum
Solvent:$CDCl_3$
Spectrometer Frequency: 376 MHz

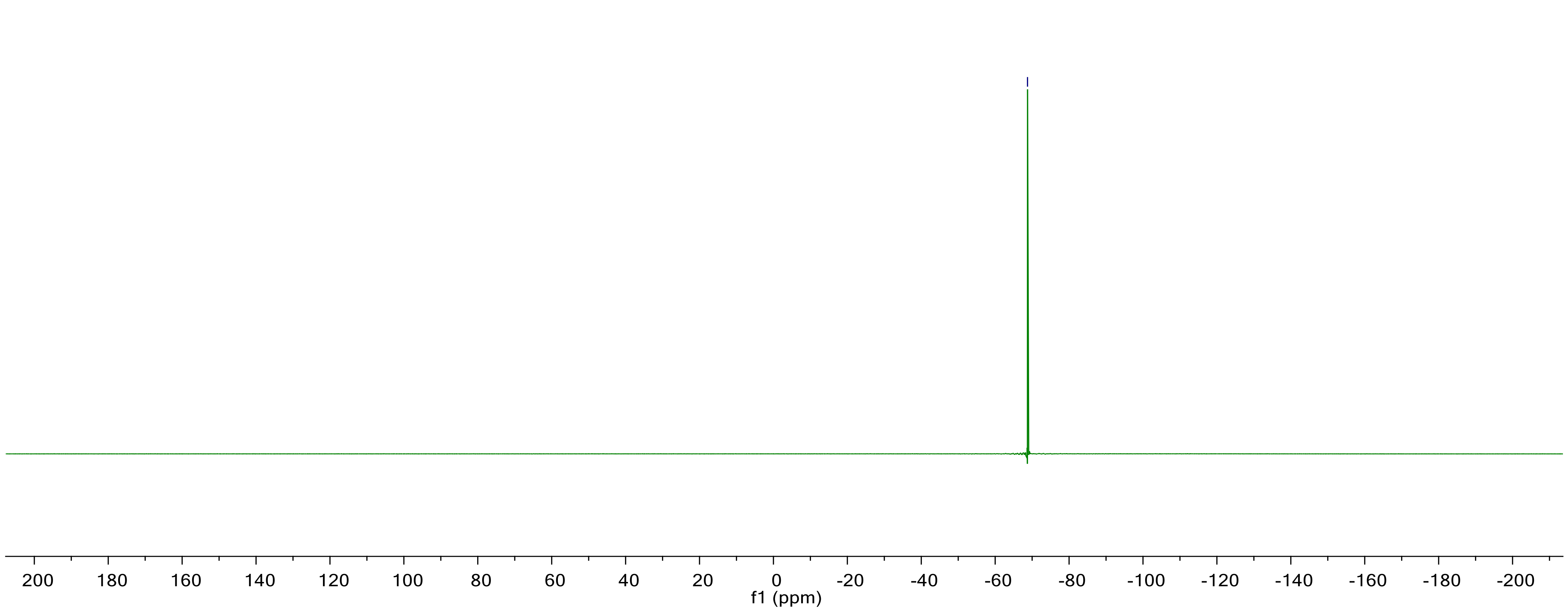

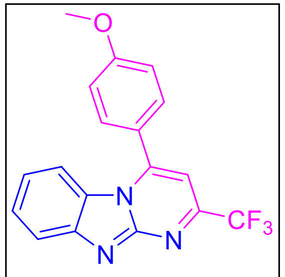

3c

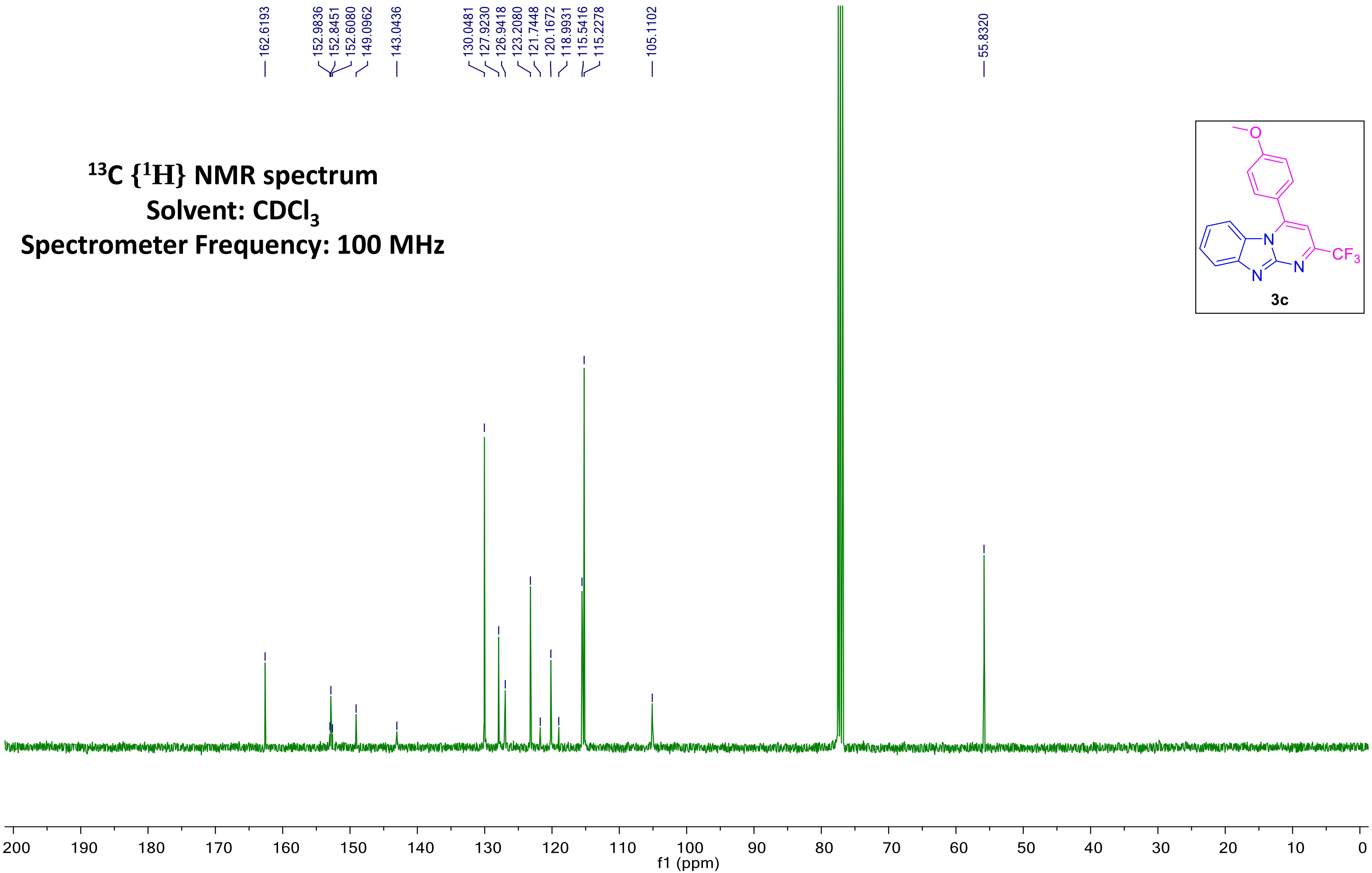
162.6193
152.9836
152.8451
152.6080
149.0962
143.0436
130.0481
127.9230
126.9418
123.2080
121.7448
120.1672
118.9931
115.5416
115.2278
105.1102
55.8320
13C {1H} NMR spectrum
Solvent: CDCl3
Spectrometer Frequency: 100 MHz
f1 (ppm)
CF3
N
N
N
O
3c

^{1}H NMR spectrum
Solvent: $CDCl_3$
Spectrometer Frequency: 400 MHz

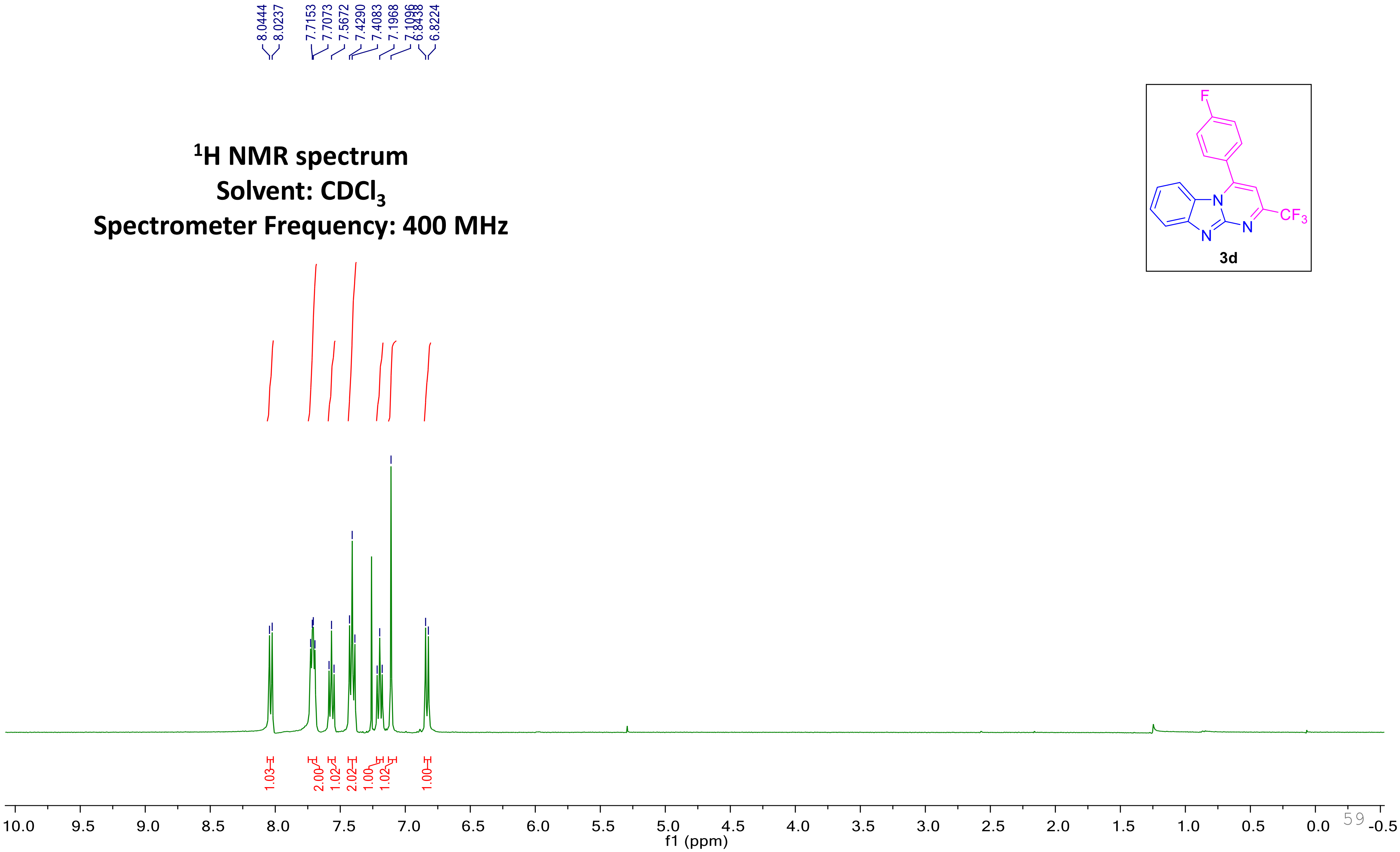

3d

$^{19}F\{^1H\}$ NMR spectrum

Solvent:$CDCl_3$

Spectrometer Frequency: 376 MHz

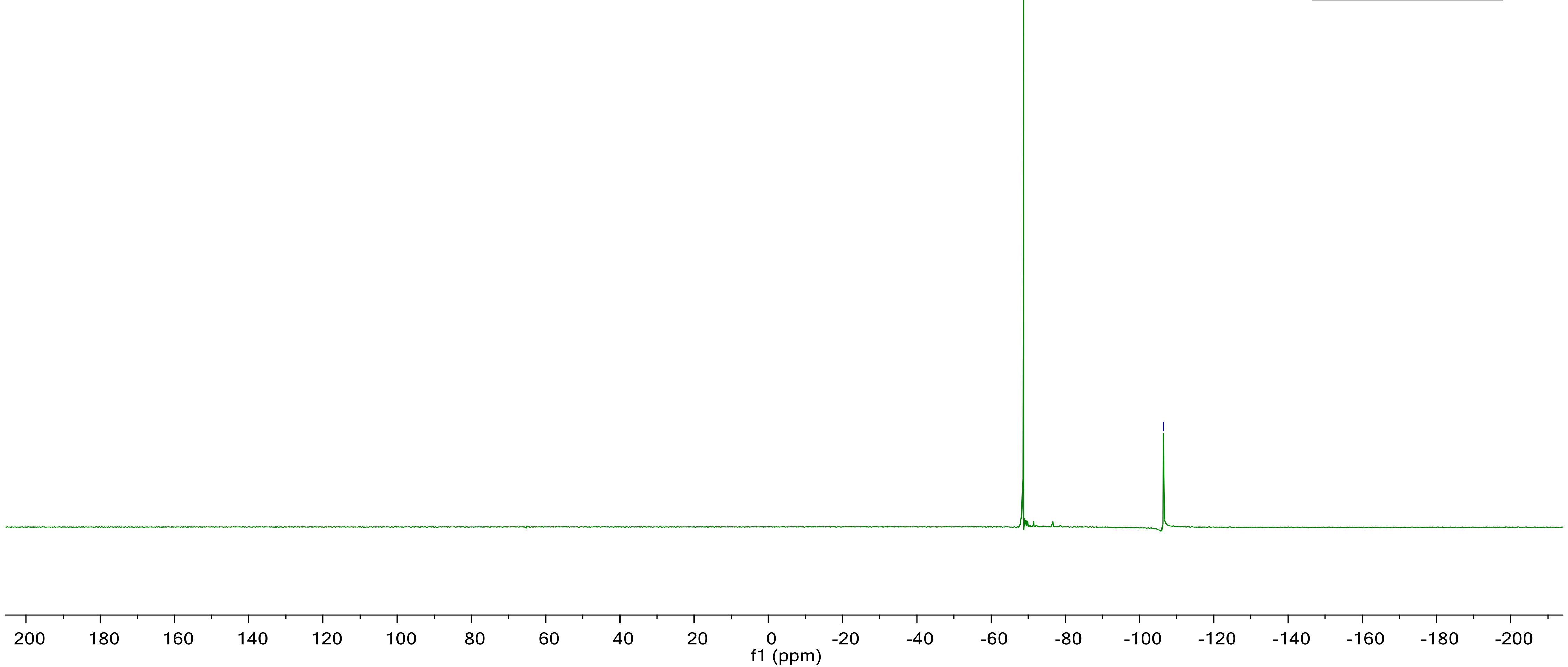

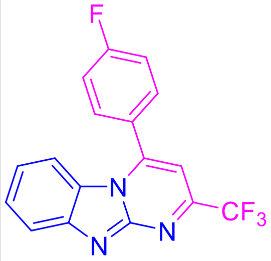

3d

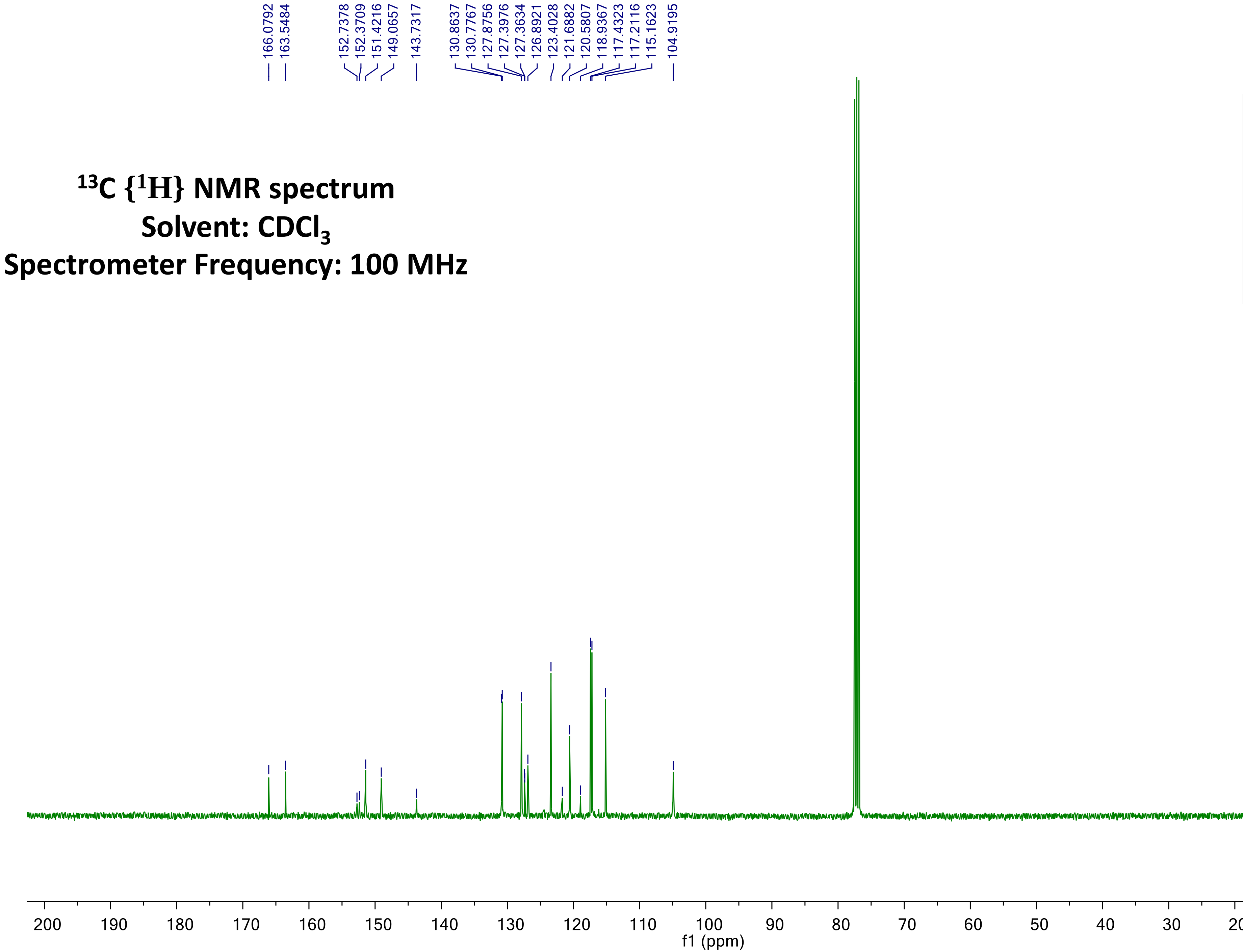
166.0792
163.5484
152.7378
152.3709
151.4216
149.0657
143.7317
130.8637
130.7767
127.8756
127.3976
127.3634
126.8921
123.4028
121.6882
120.5807
118.9367
117.4323
117.2116
115.1623
104.9195
13C {1H} NMR spectrum
Solvent: CDCl3
Spectrometer Frequency: 100 MHz
200 190 180 170 160 150 140 130 120 110 100 90 80 70 60 50 40 30 20 10 0
f1 (ppm)

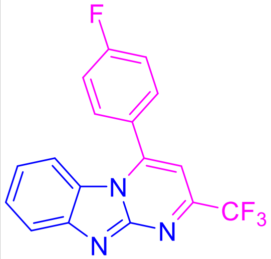
F
N
N
N
CF3

3d

^{1}H NMR spectrum
Solvent: $CDCl_3$
Spectrometer Frequency: 400 MHz

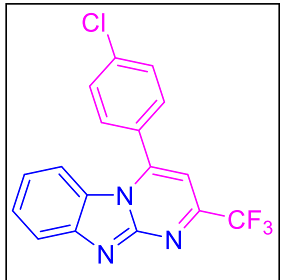

3e

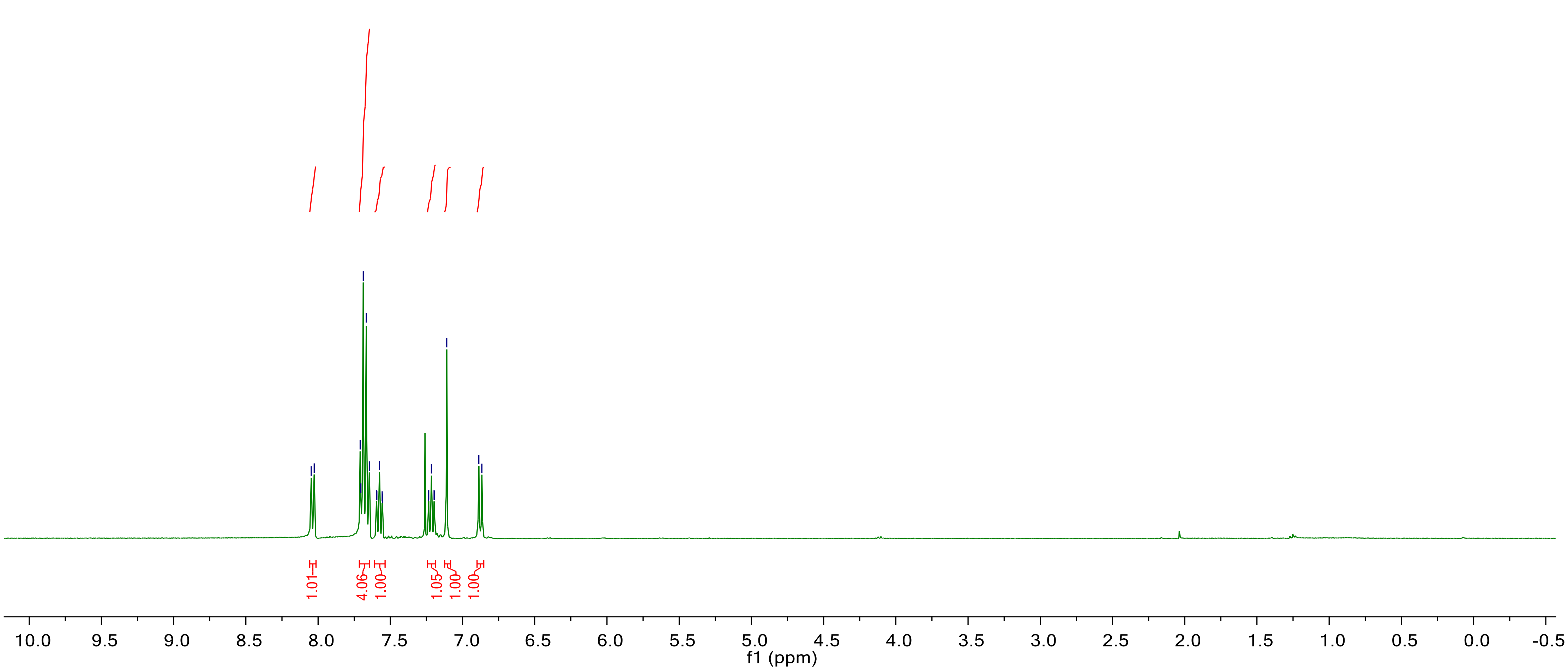

$^{19}F\{^{1}H\}$ NMR spectrum
Solvent:$CDCl_3$
Spectrometer Frequency: 376 MHz

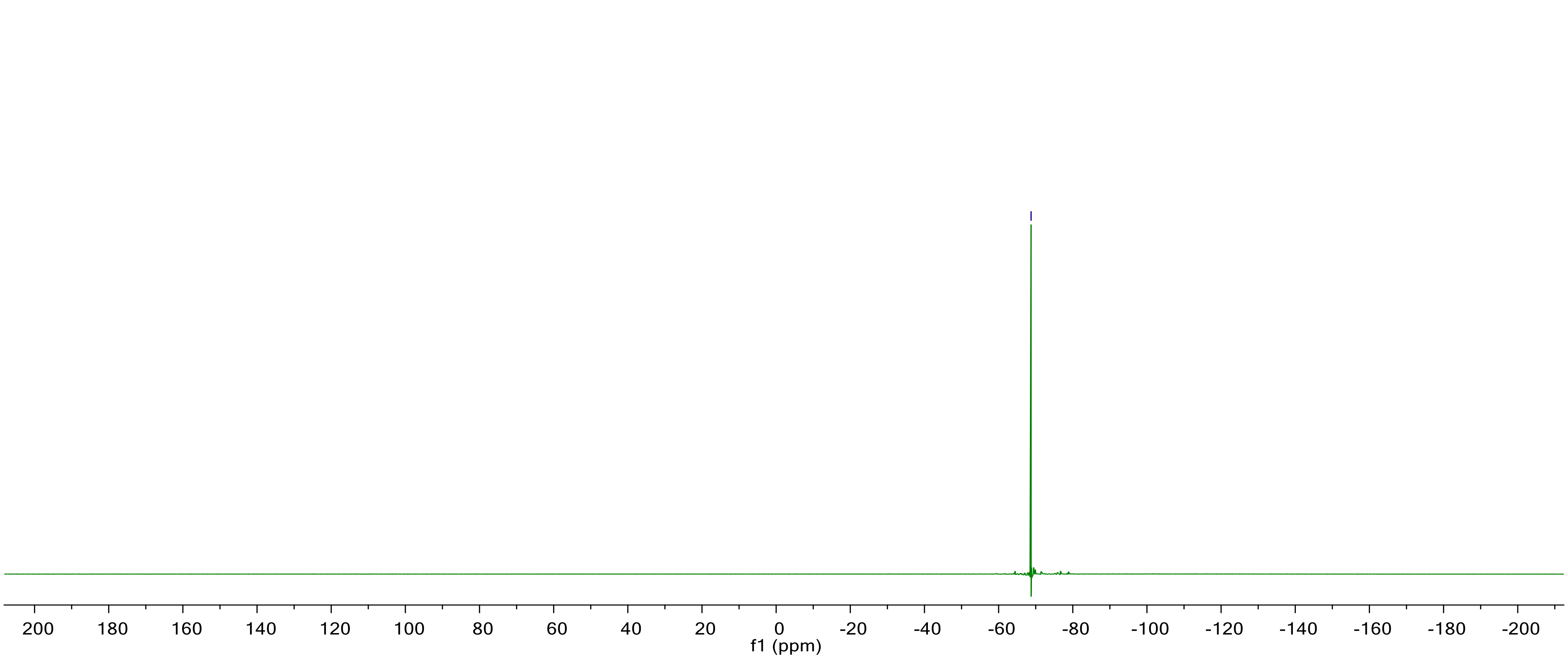

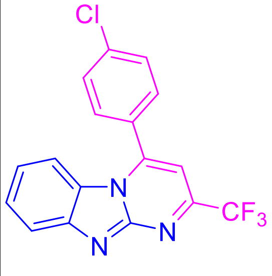

3e

^{13}C {^1H} NMR spectrum
Solvent: $CDCl_3$
Spectrometer Frequency: 100 MHz

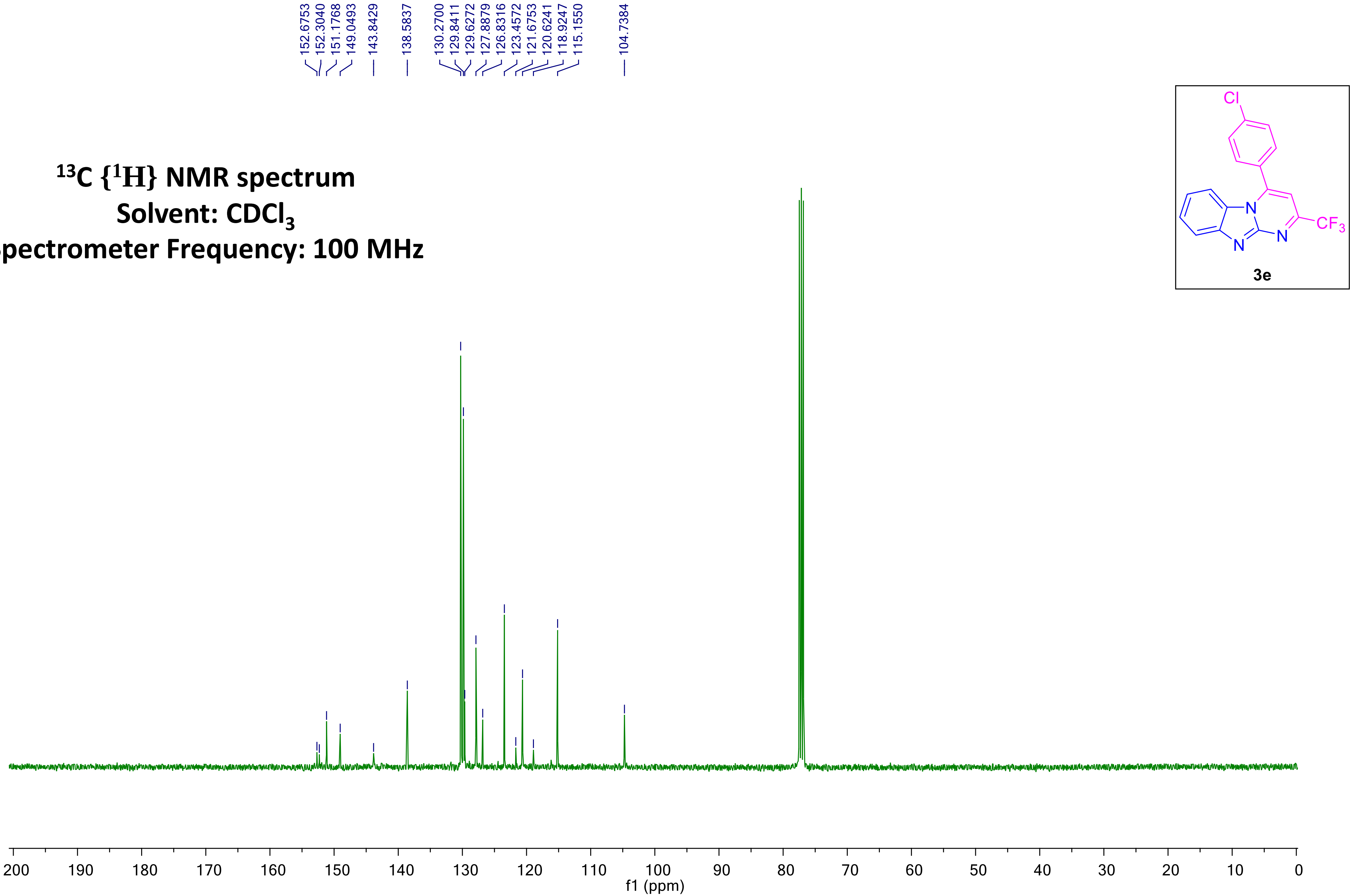

3e

^{1}H NMR spectrum
Solvent: $CDCl_3$
Spectrometer Frequency: 400 MHz

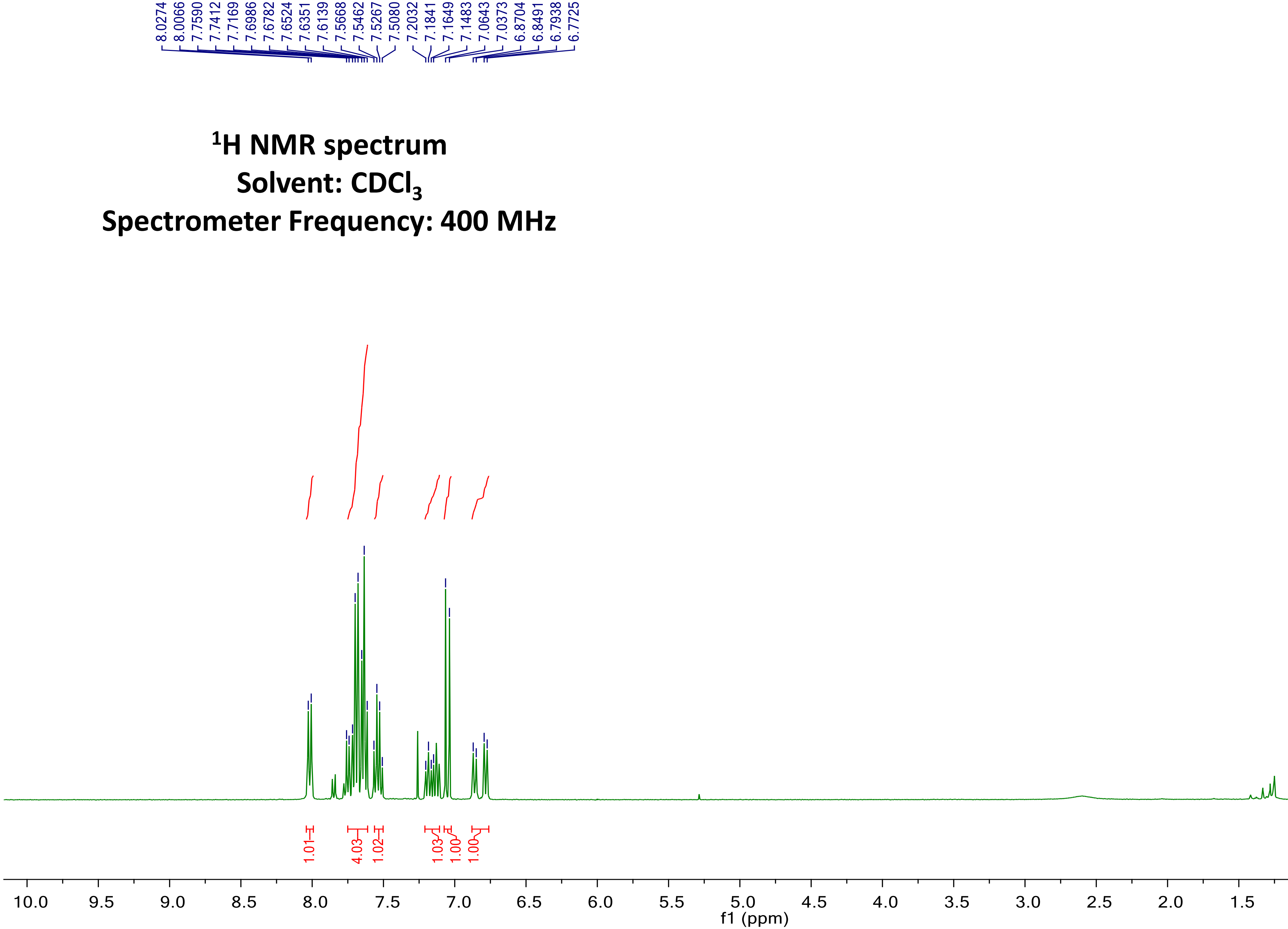

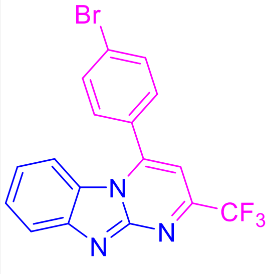

3f

$^{19}F\{^1H\}$ NMR spectrum
Solvent:$CDCl_3$
Spectrometer Frequency: 376 MHz

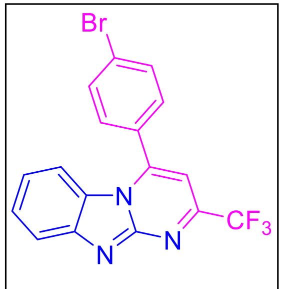

3f

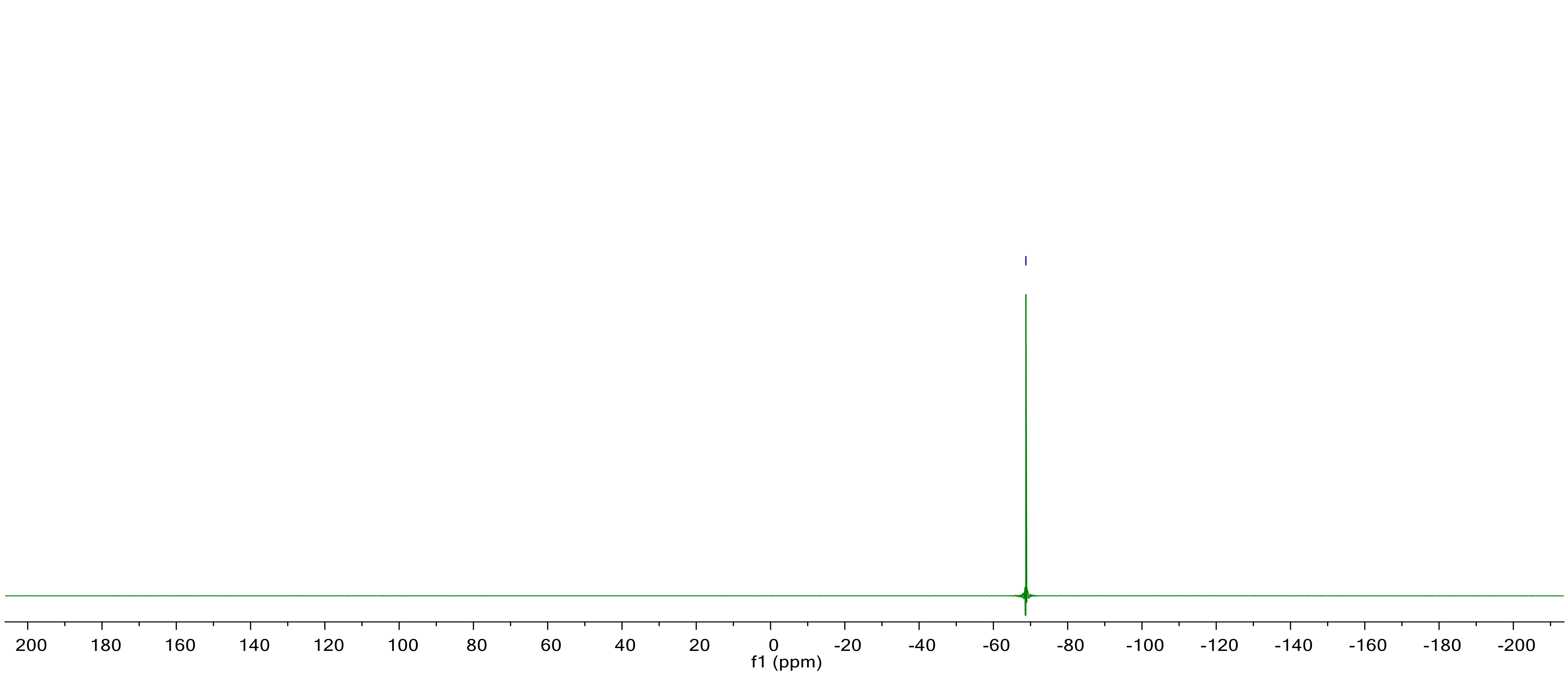

^{13}C {^{1}H} NMR spectrum
Solvent: $CDCl_3$
Spectrometer Frequency: 100 MHz

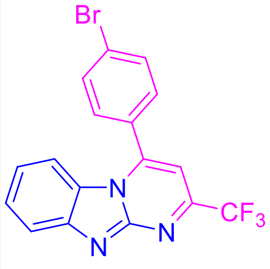

3f

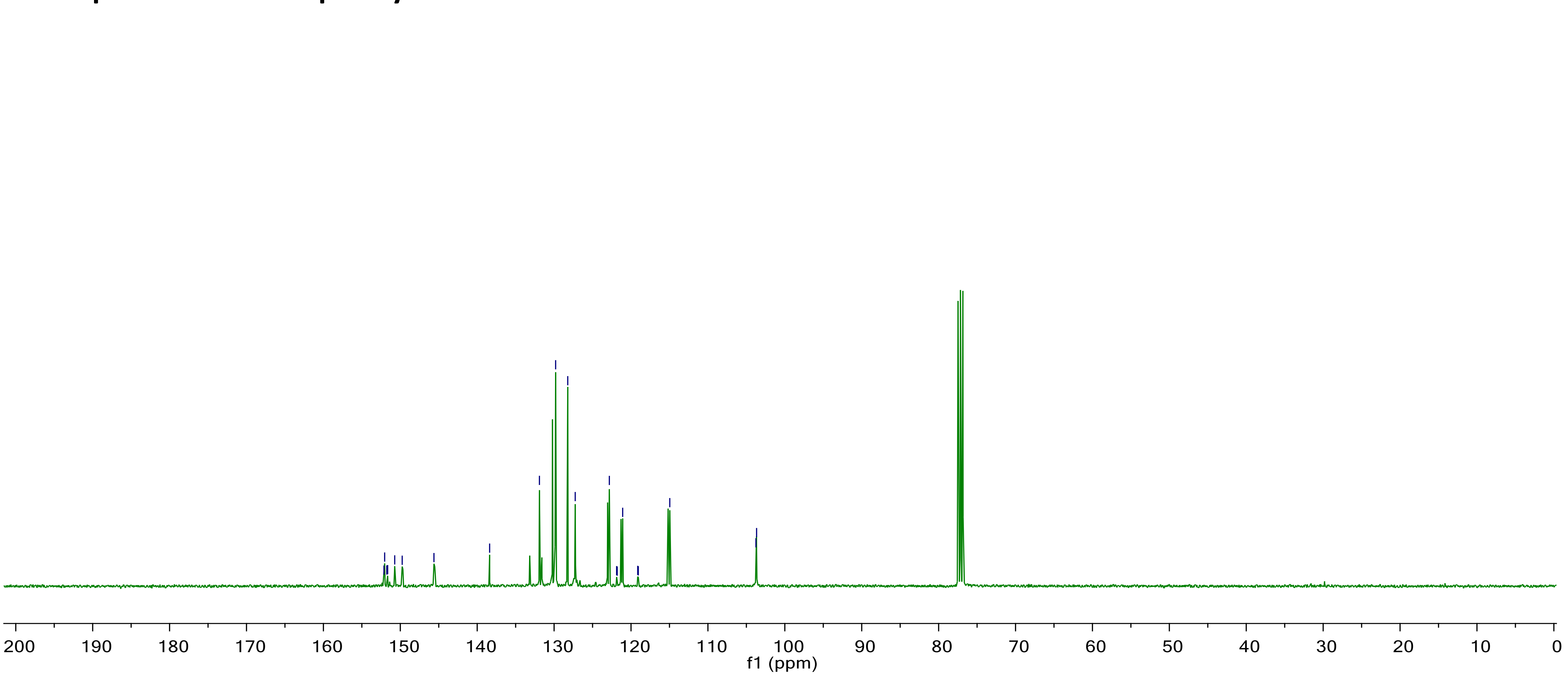

^{1}H NMR spectrum
Solvent: $CDCl_3$
Spectrometer Frequency: 400 MHz

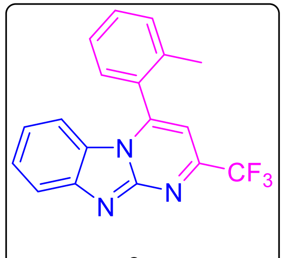

3g

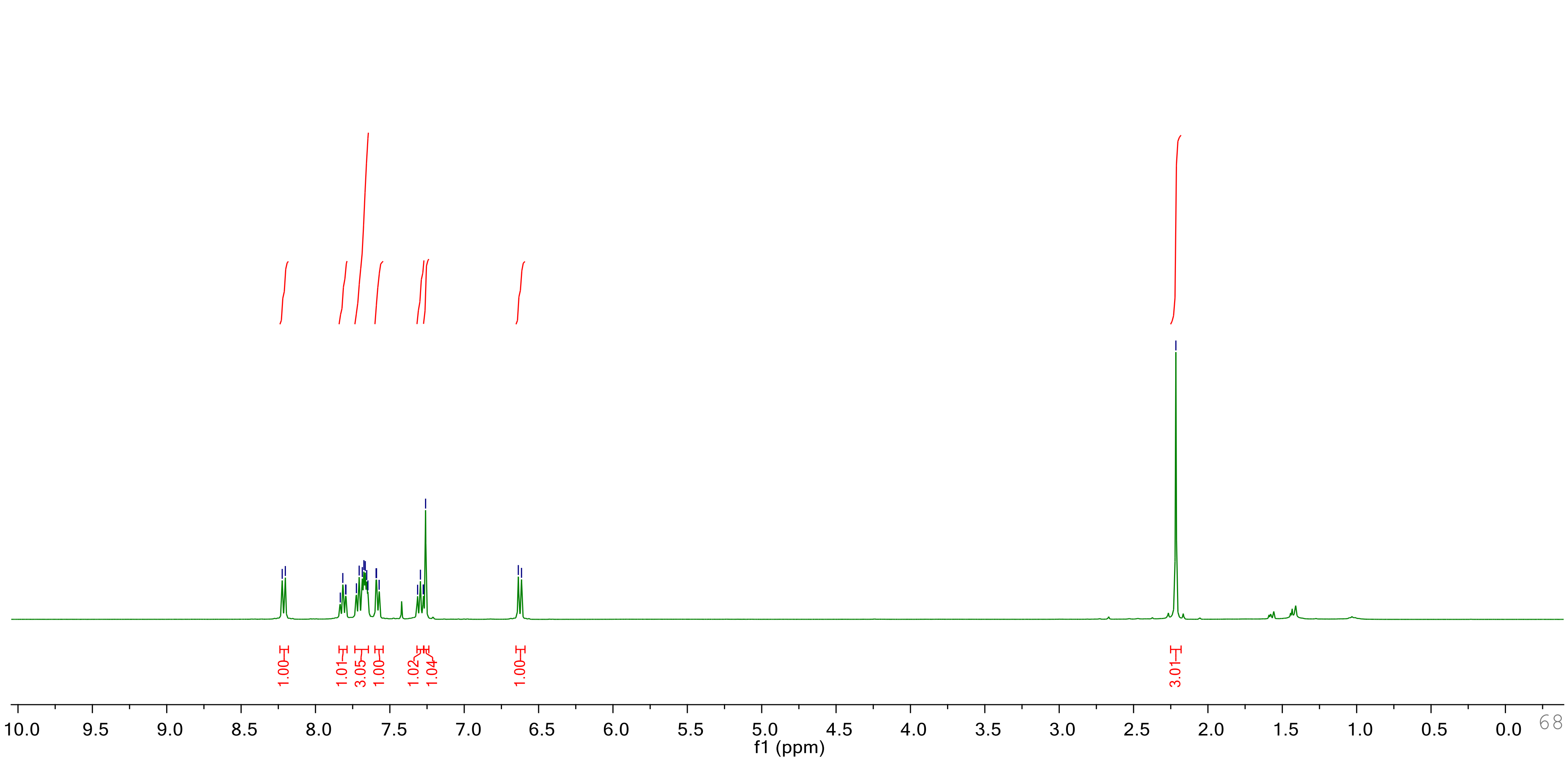

$^{19}F\{^{1}H\}$ NMR spectrum
Solvent:$CDCl_3$
Spectrometer Frequency: 376 MHz

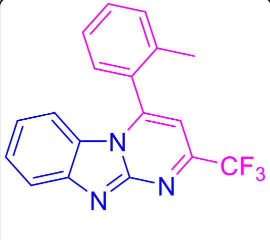

3g

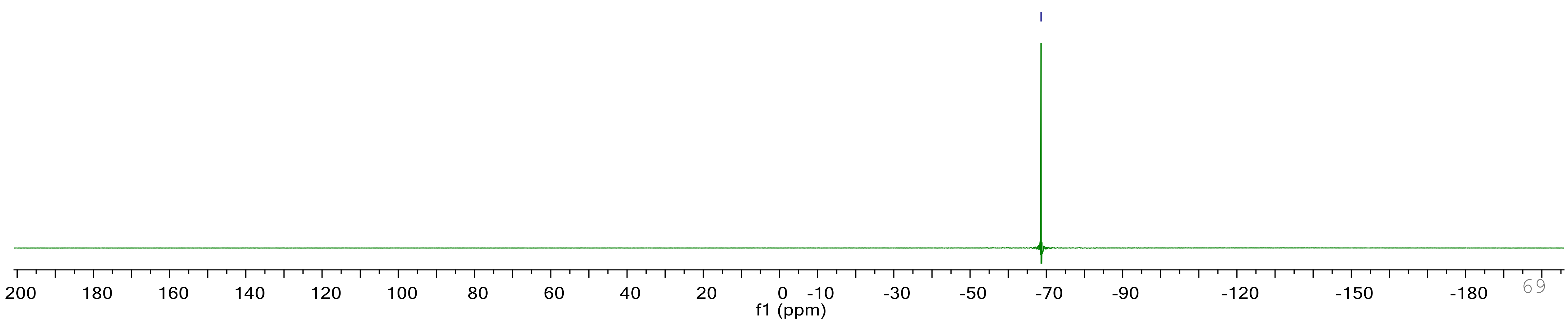

^{13}C {^{1}H} NMR spectrum
Solvent: $CDCl_3$
Spectrometer Frequency: 100 MHz

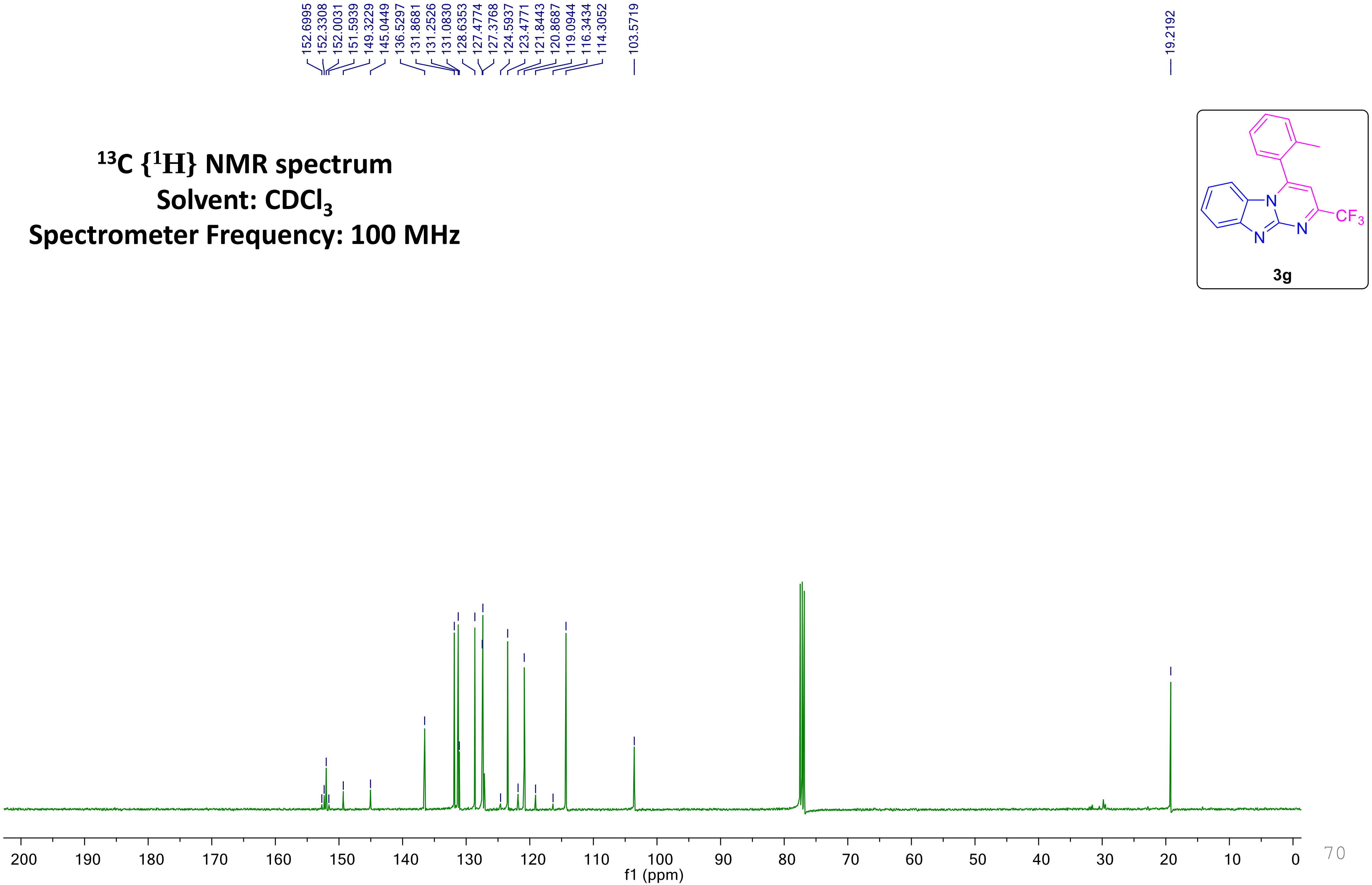

3g

^{1}H NMR spectrum
Solvent: $CDCl_3$
Spectrometer Frequency: 400 MHz

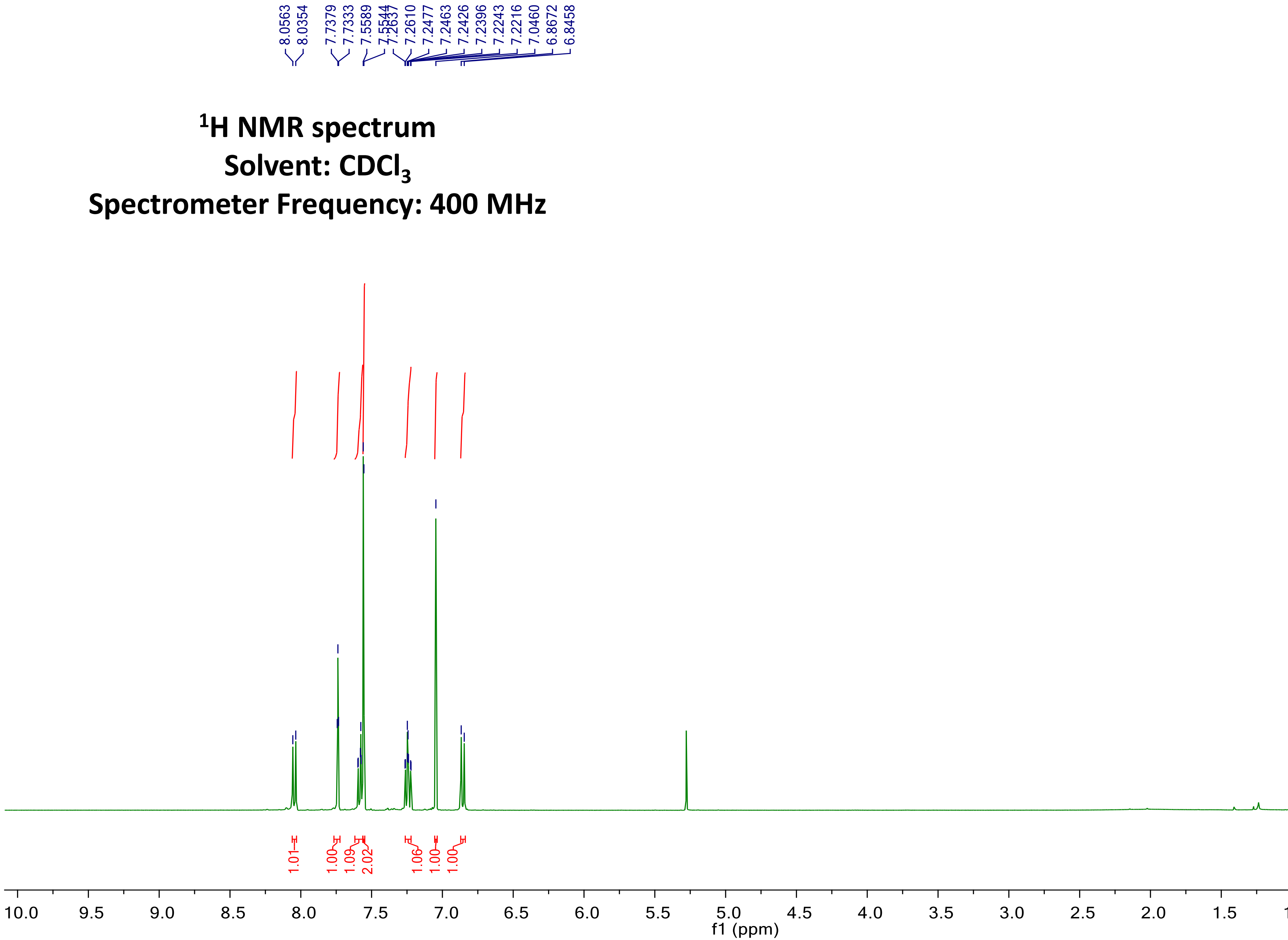

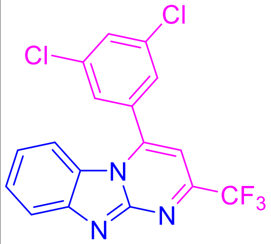

3h

$^{19}F\{^{1}H\}$ NMR spectrum
Solvent:$CDCl_3$
Spectrometer Frequency: 376 MHz

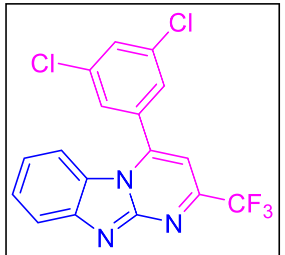

3h

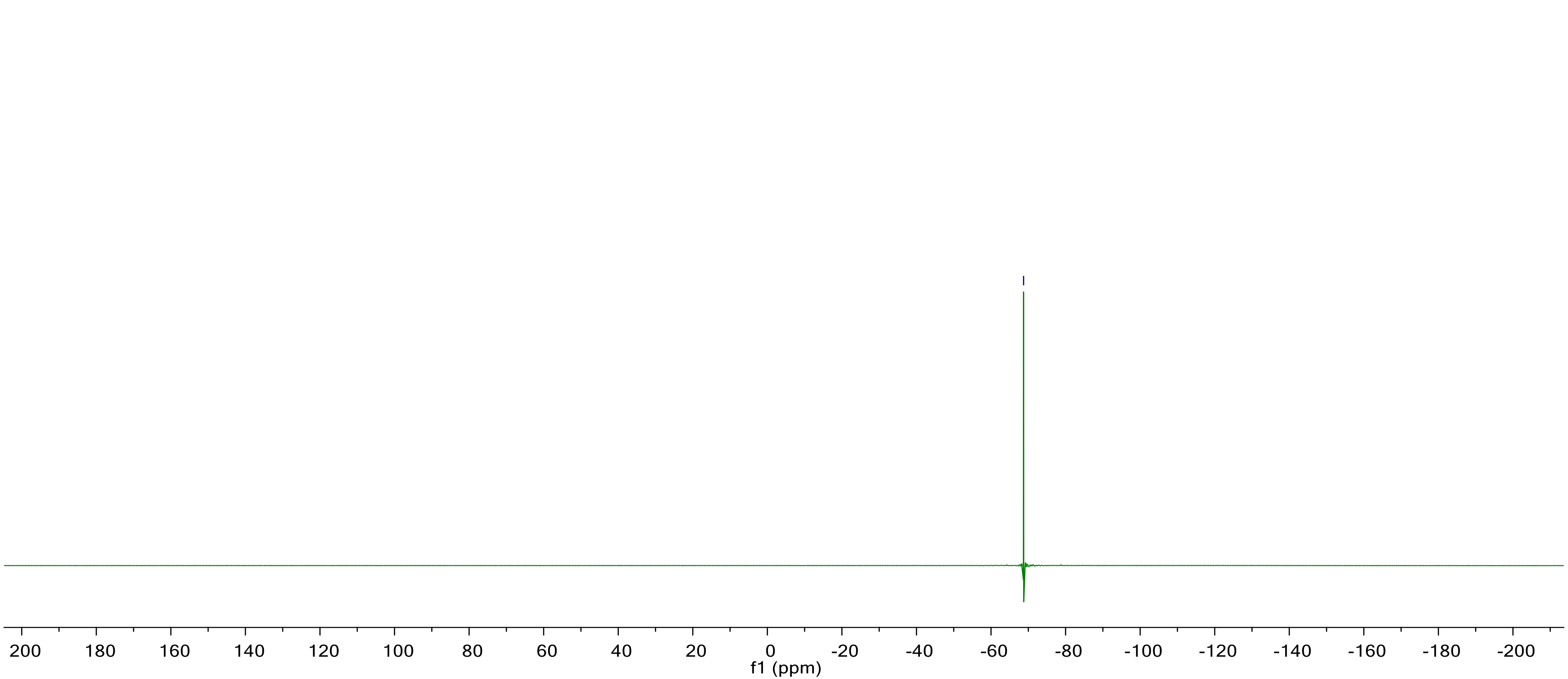

$^{13}C\ \{^{1}H\}$ **NMR spectrum**

Solvent: $CDCl_3$

Spectrometer Frequency: 100 MHz

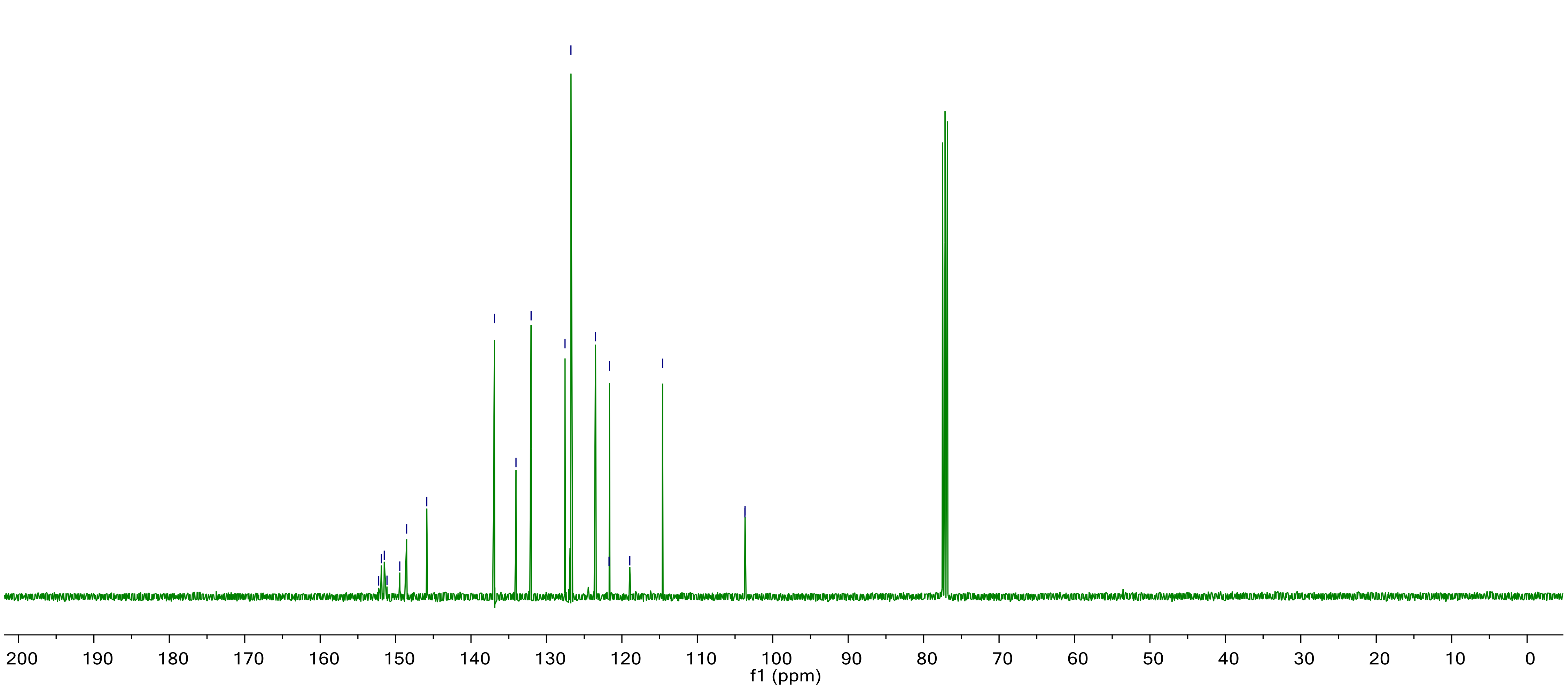

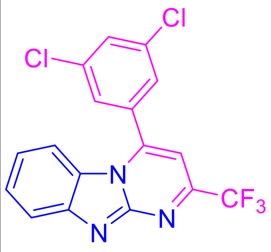

3h

^{1}H NMR spectrum
Solvent: $CDCl_3$
Spectrometer Frequency: 400 MHz

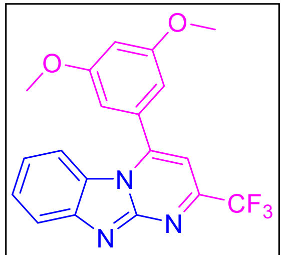

3i

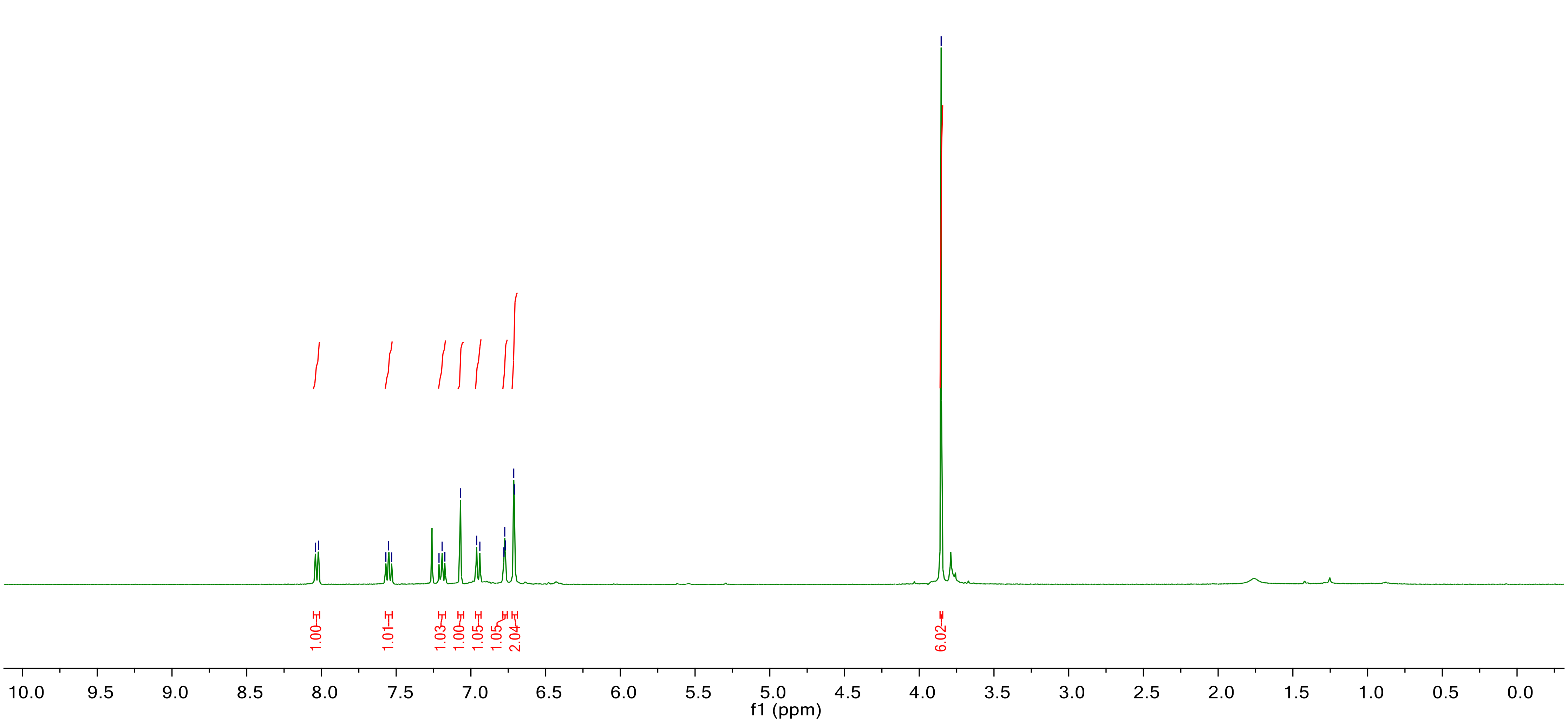

$^{19}F\{^{1}H\}$ NMR spectrum
Solvent:$CDCl_3$
Spectrometer Frequency: 376 MHz

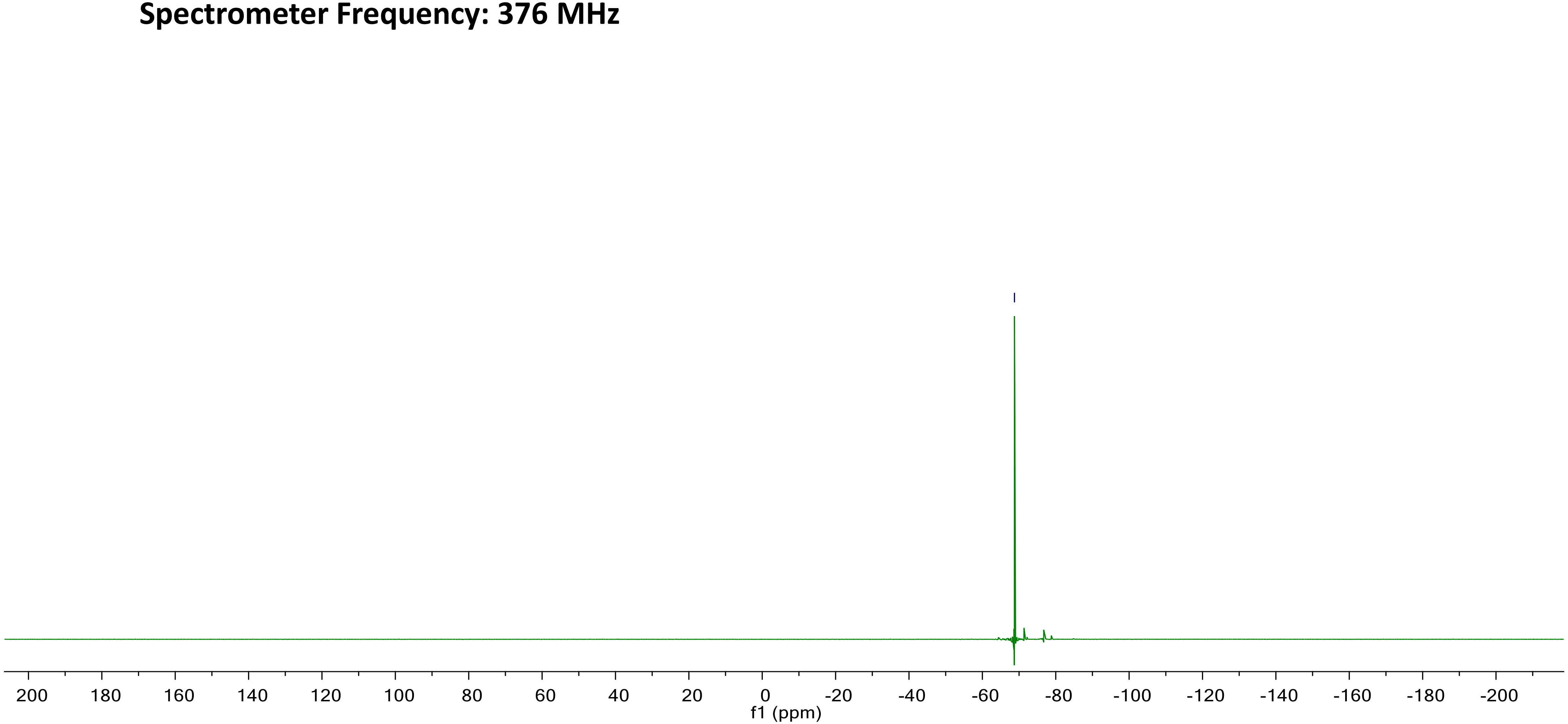

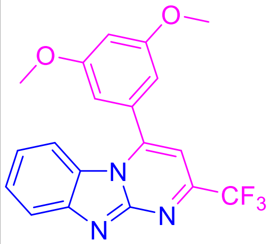

3i

^{13}C {^{1}H} NMR spectrum
Solvent: $CDCl_3$
Spectrometer Frequency: 100 MHz

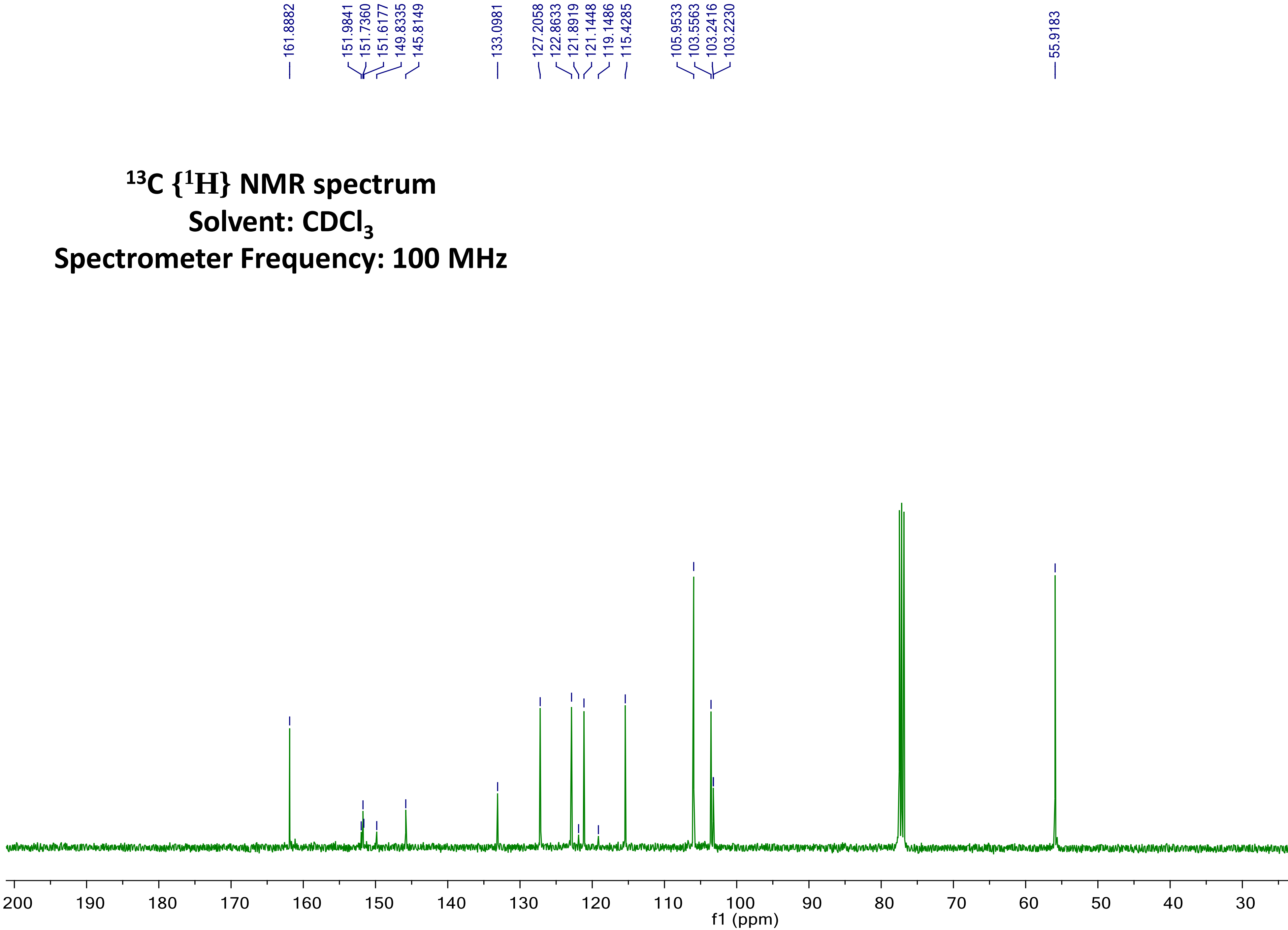

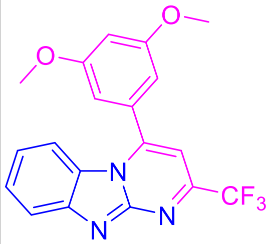

3i

^{1}H NMR spectrum
Solvent: $CDCl_3$
Spectrometer Frequency: 400 MHz

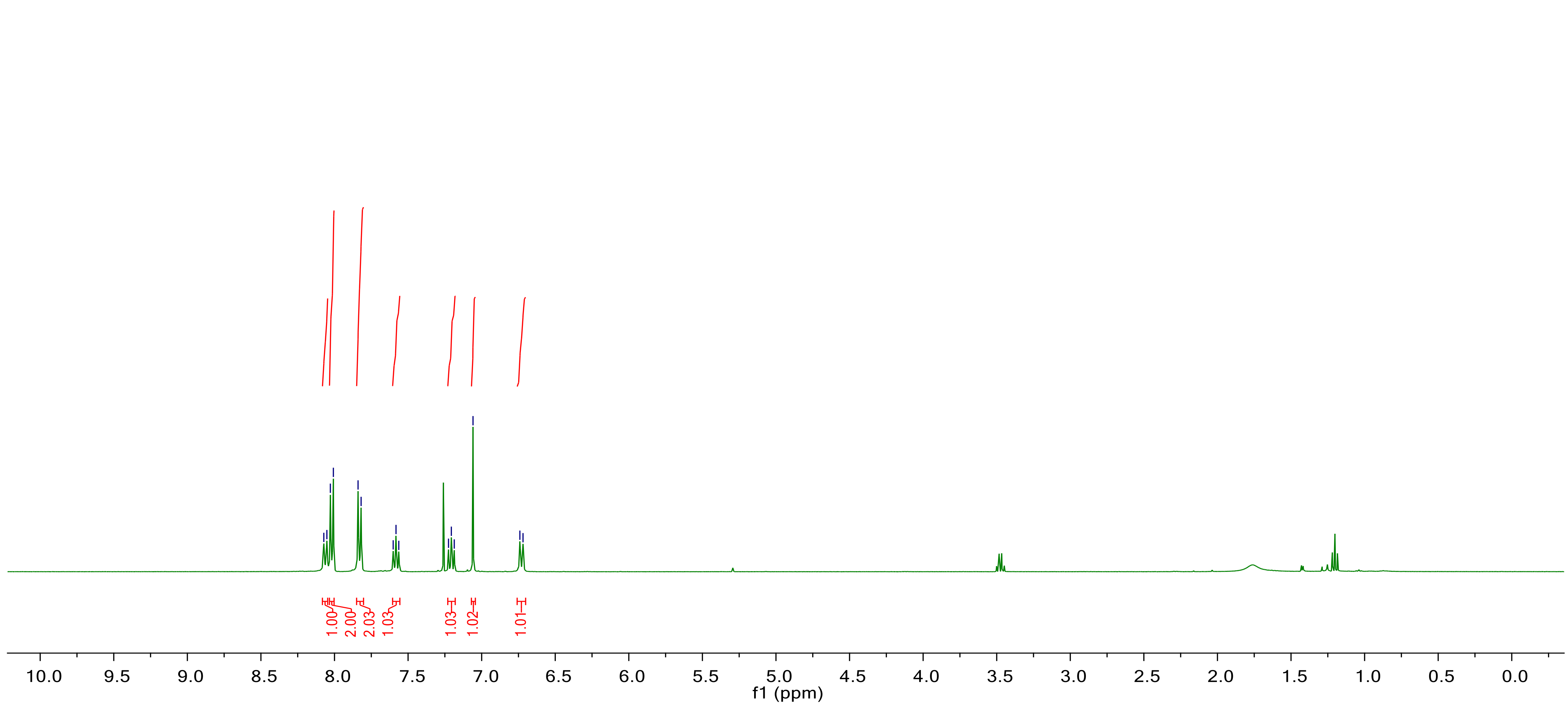

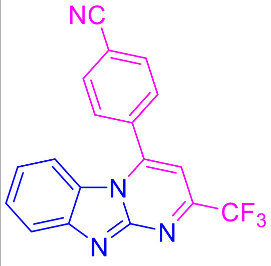

3j

$^{19}F\{^{1}H\}$ NMR spectrum
Solvent:$CDCl_3$
Spectrometer Frequency: 376 MHz

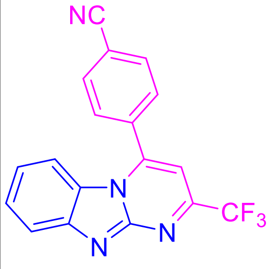

3j

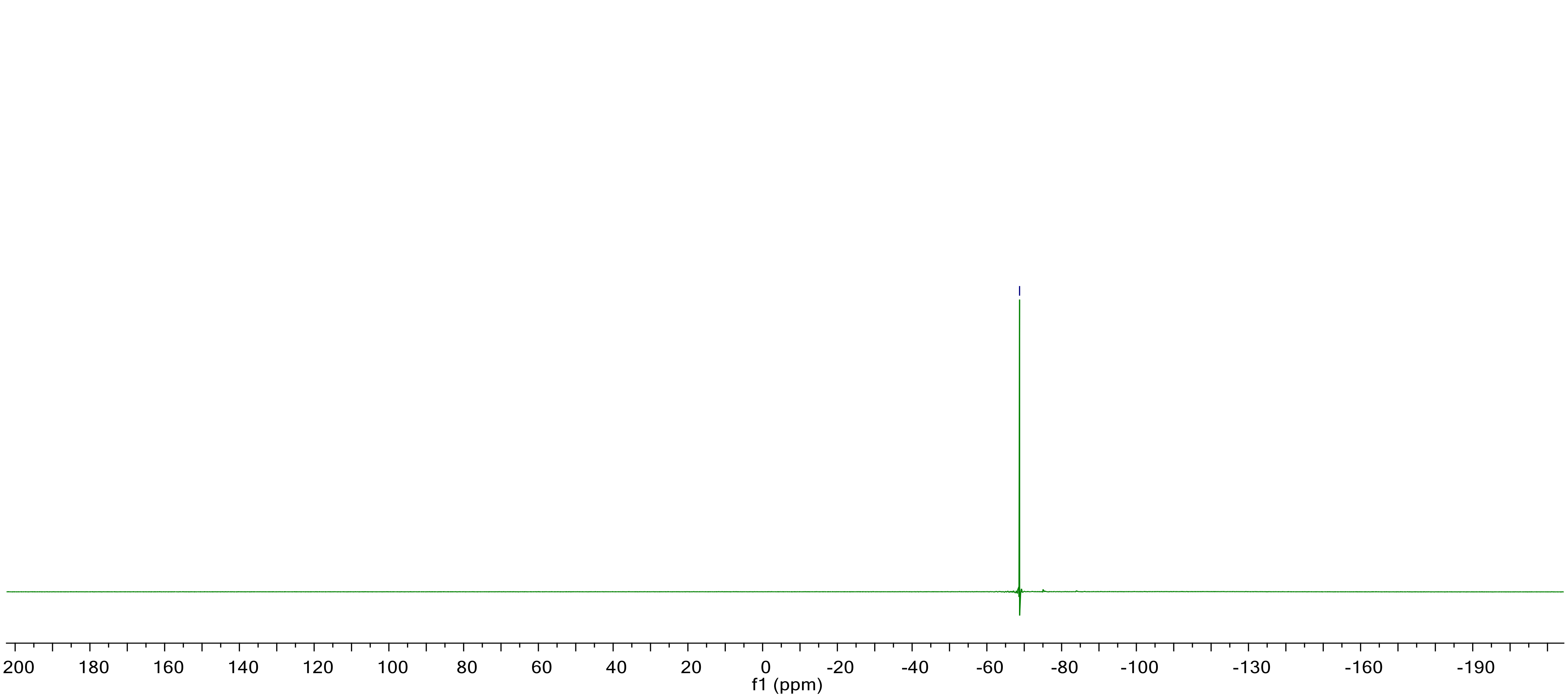

$^{13}C\ \{^{1}H\}$ NMR spectrum
Solvent: $CDCl_3$
Spectrometer Frequency: 100 MHz

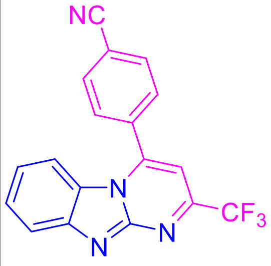

3j

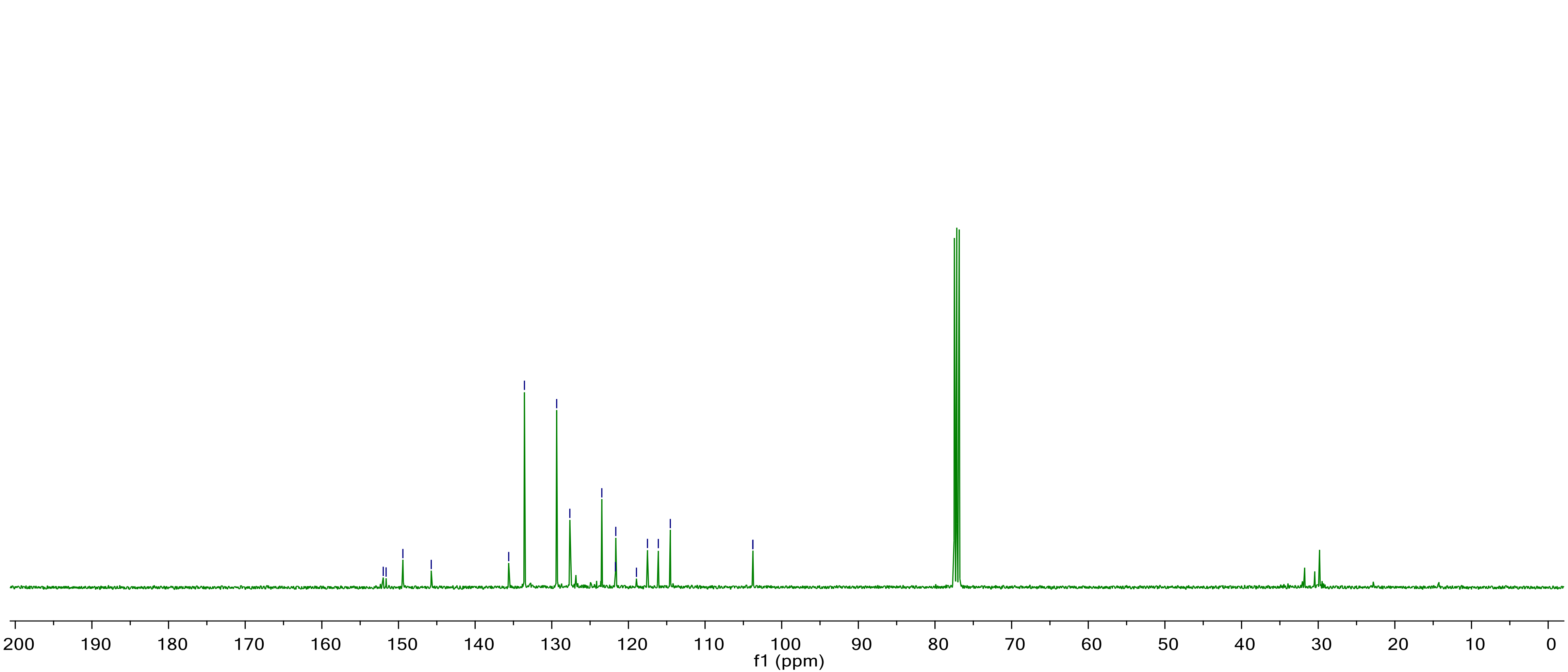

^{1}H NMR spectrum
Solvent: DMSO-d_6
Spectrometer Frequency: 400 MHz

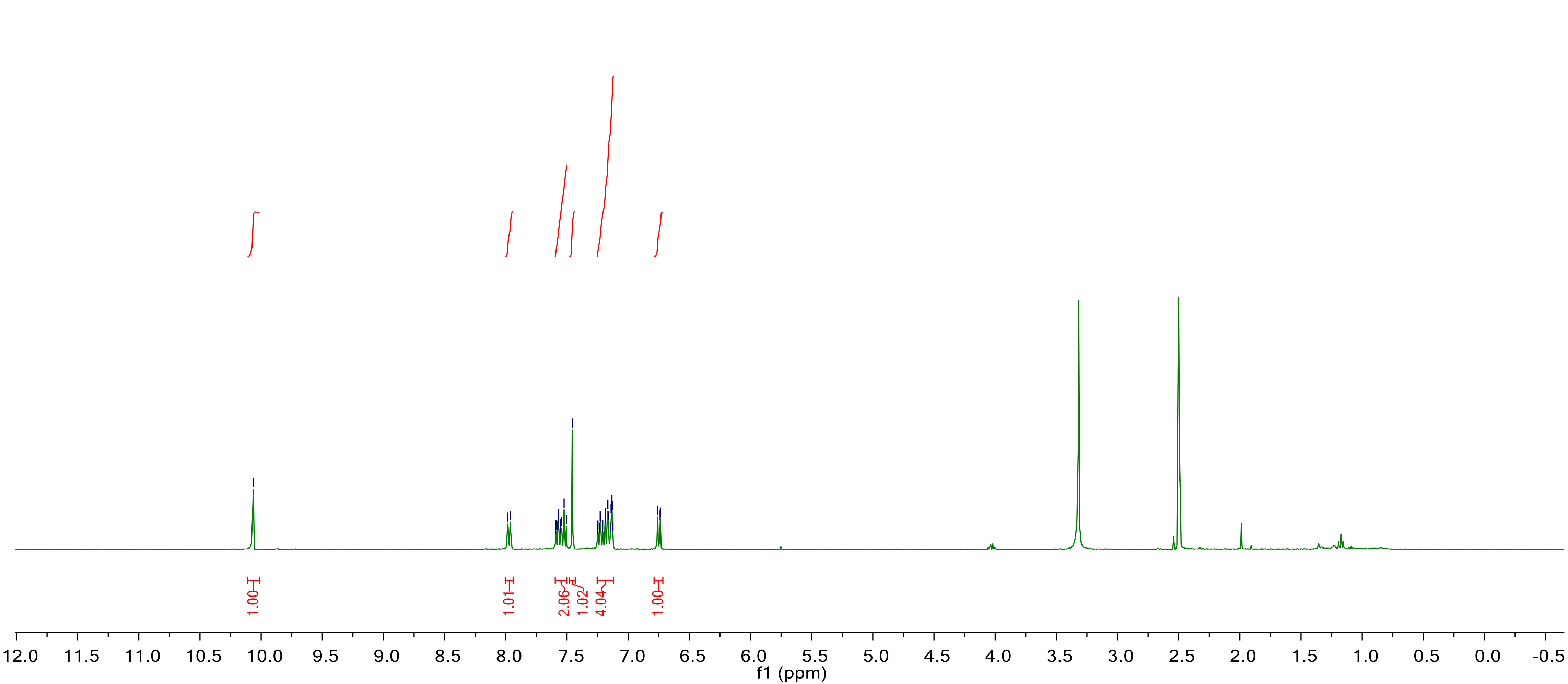

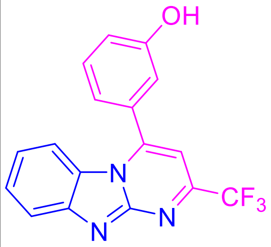

3k

$^{19}F\{^{1}H\}$ NMR spectrum
Solvent:DMSO-d_6
Spectrometer Frequency: 376 MHz

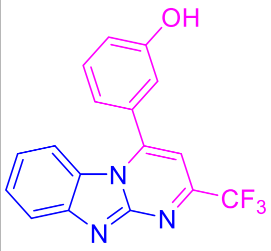

3k

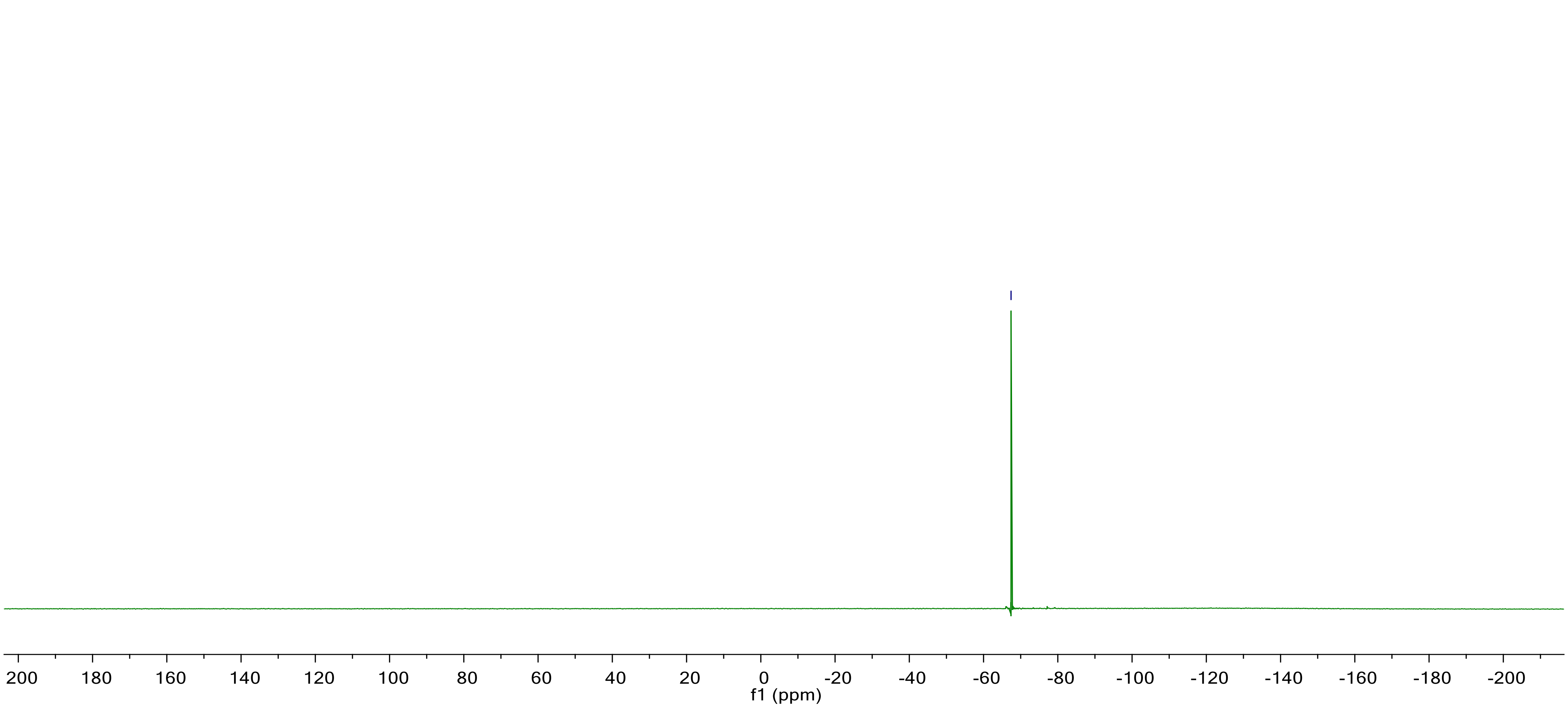

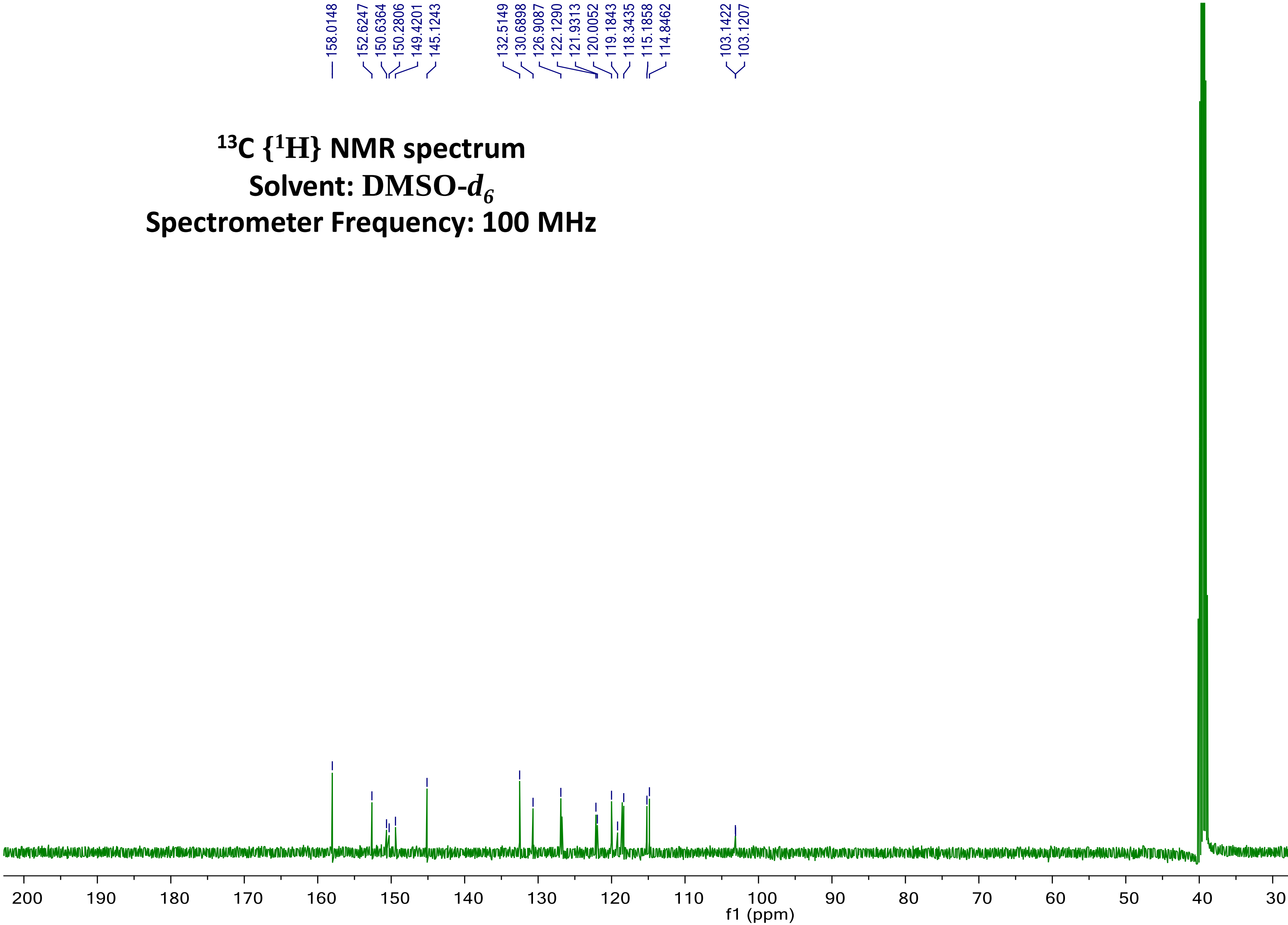

^{13}C {^{1}H} NMR spectrum
Solvent: DMSO-d_6
Spectrometer Frequency: 100 MHz

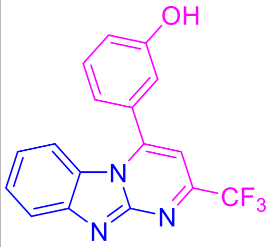

3k

^{1}H NMR spectrum
Solvent: DMSO-d_6
Spectrometer Frequency: 400 MHz

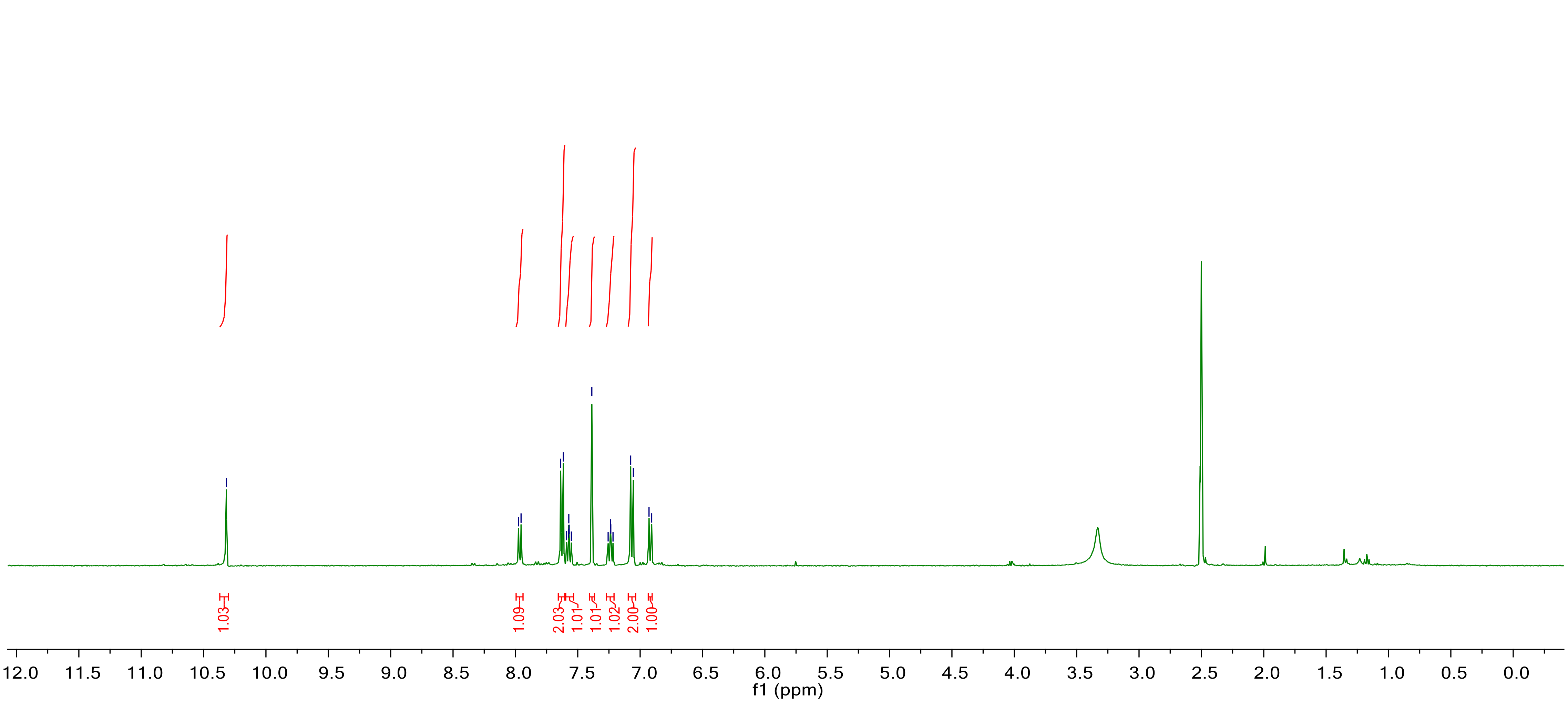

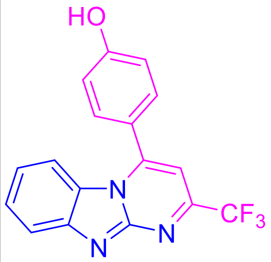

3l

$^{19}F\{^1H\}$ NMR spectrum
Solvent:DMSO-d_6
Spectrometer Frequency: 376 MHz

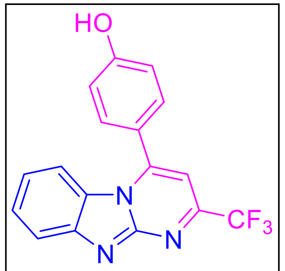

3l

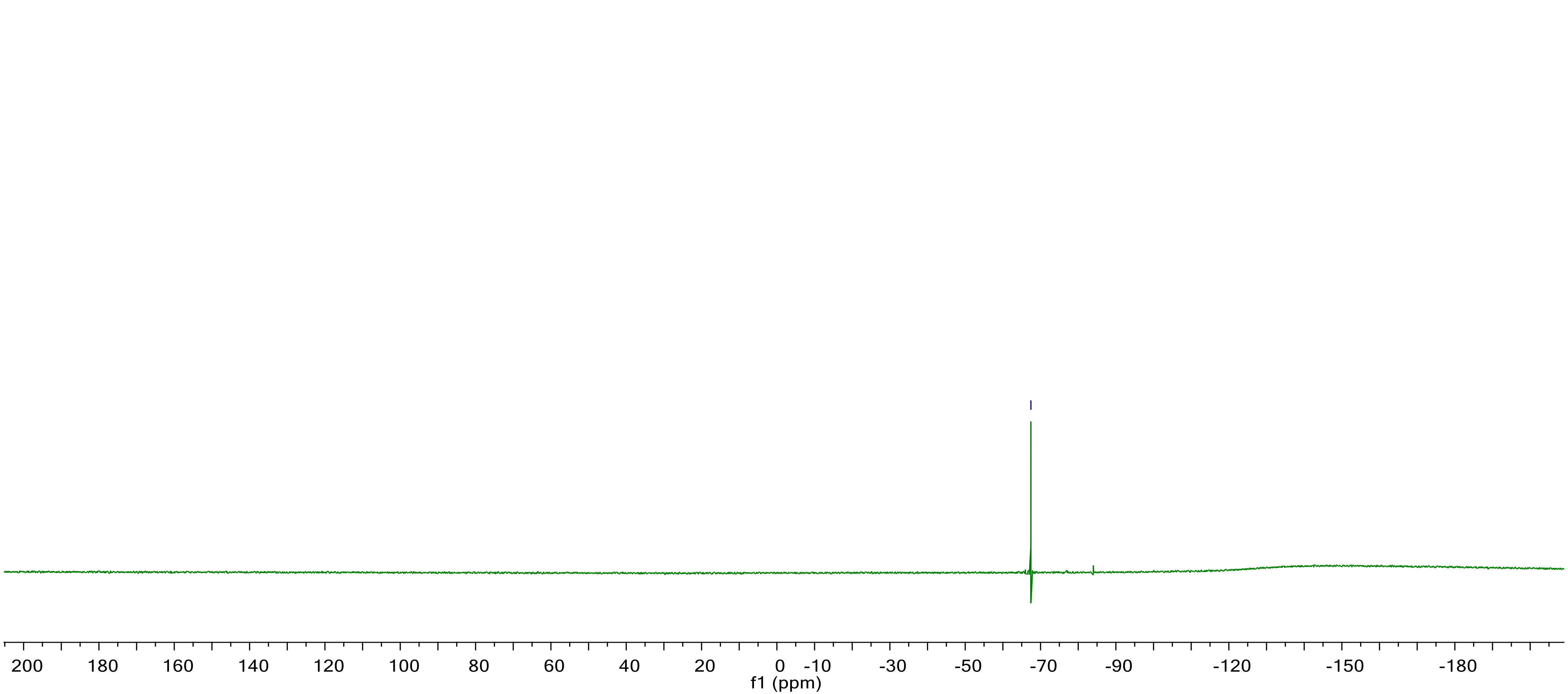

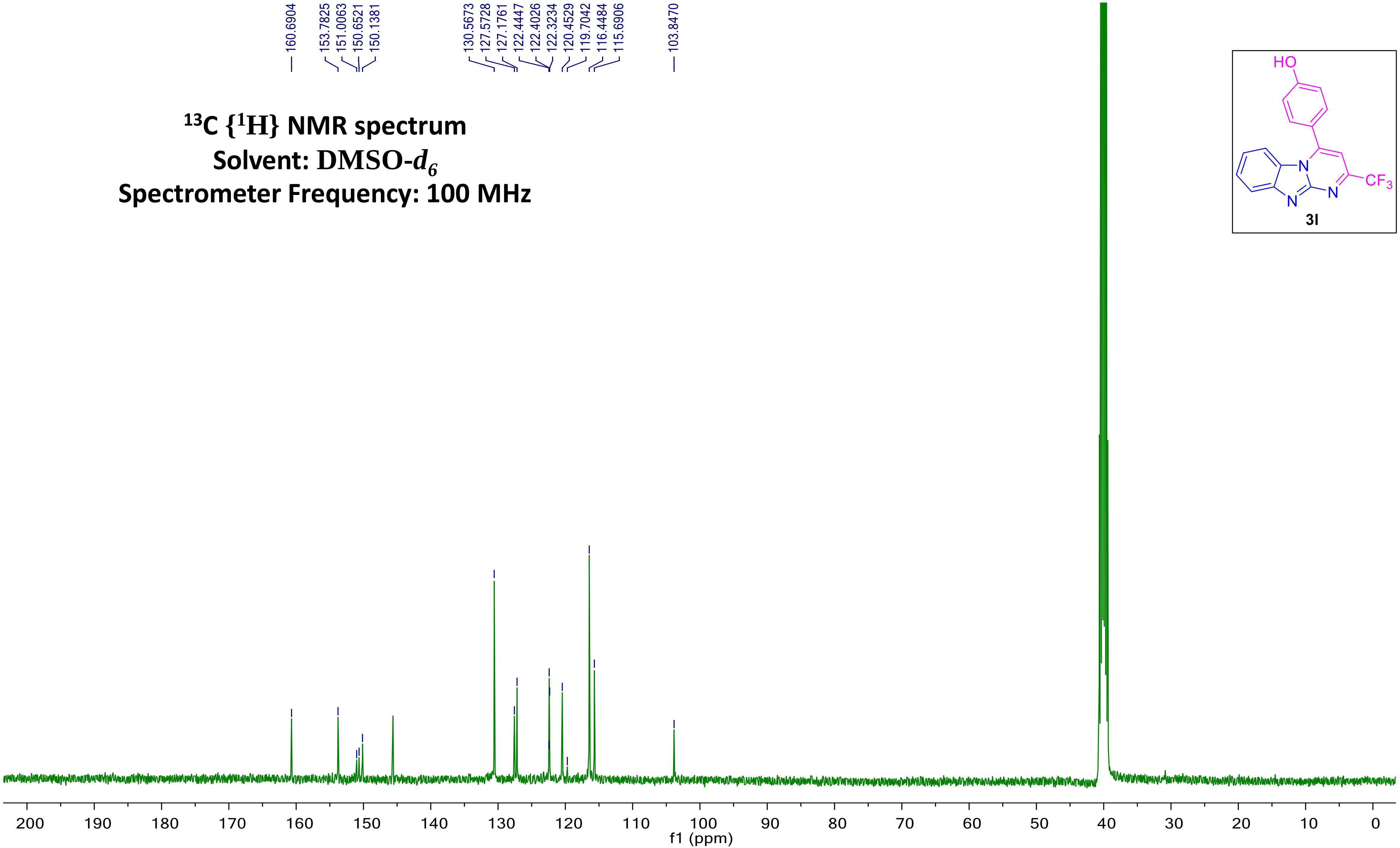
^{13}C {^{1}H} NMR spectrum
Solvent: DMSO-d_6
Spectrometer Frequency: 100 MHz
160.6904
153.7825
151.0063
150.6521
150.1381
130.5673
127.5728
127.1761
122.4447
122.4026
122.3234
120.4529
119.7042
116.4484
115.6906
103.8470
HO
N
N
N
CF_3
3l
200 190 180 170 160 150 140 130 120 110 100 90 80 70 60 50 40 30 20 10 0
f1 (ppm)

^{1}H NMR spectrum
Solvent: $CDCl_3$
Spectrometer Frequency: 400 MHz

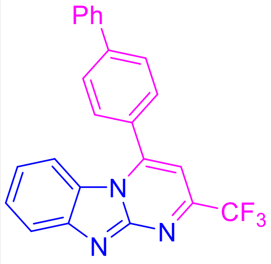

3m

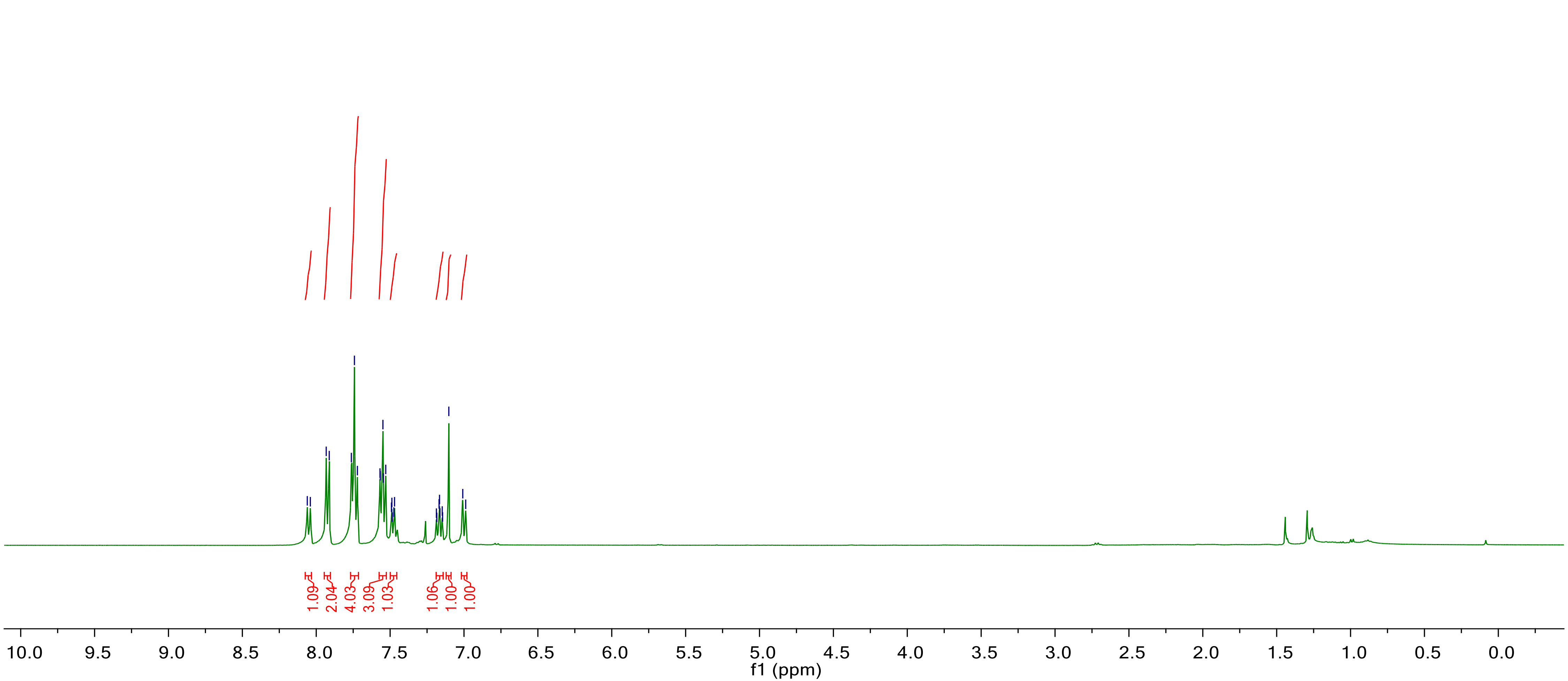

$^{19}F\{^{1}H\}$ NMR spectrum
Solvent:$CDCl_3$
Spectrometer Frequency: 376 MHz

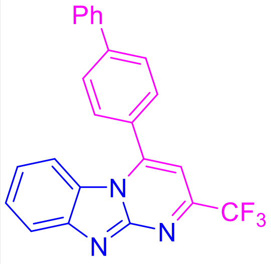

3m

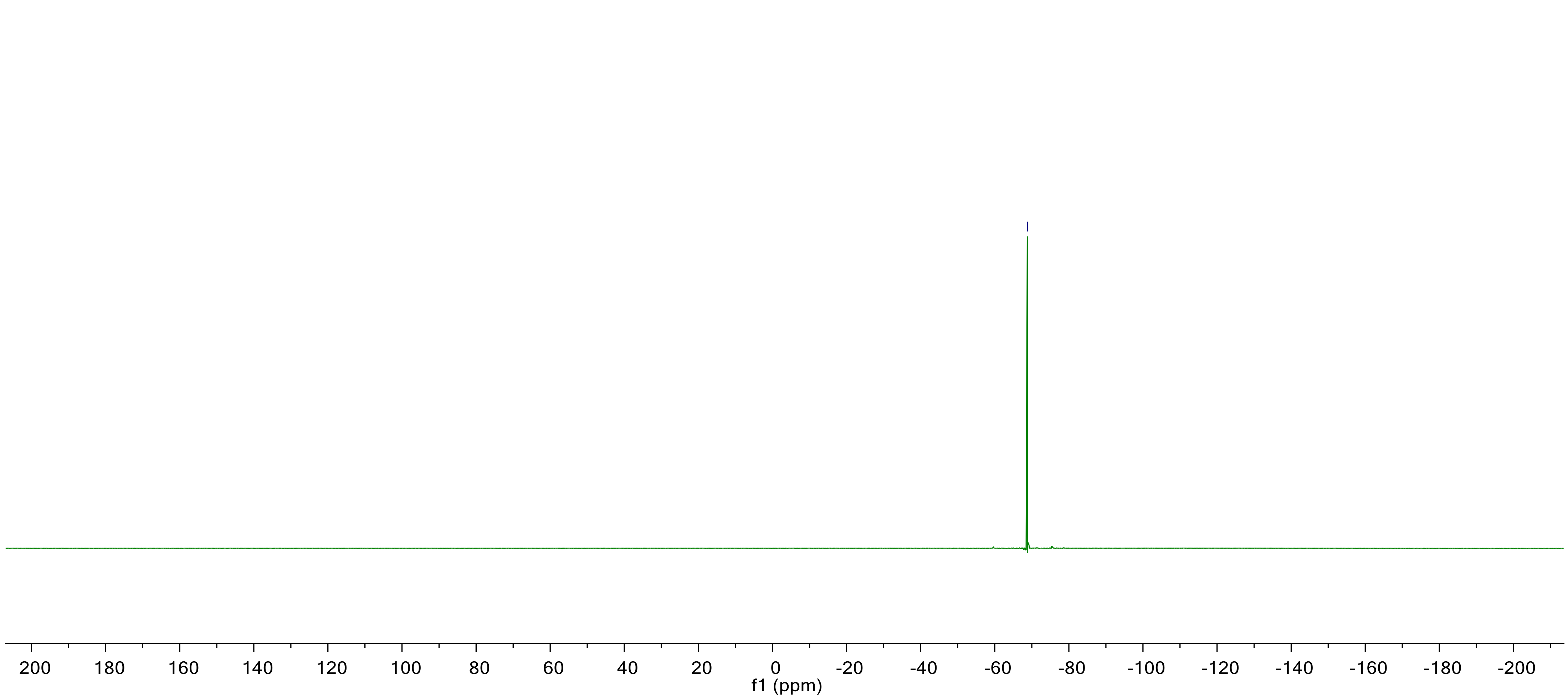

^{13}C {^{1}H} NMR spectrum
Solvent: $CDCl_3$
Spectrometer Frequency: 100 MHz

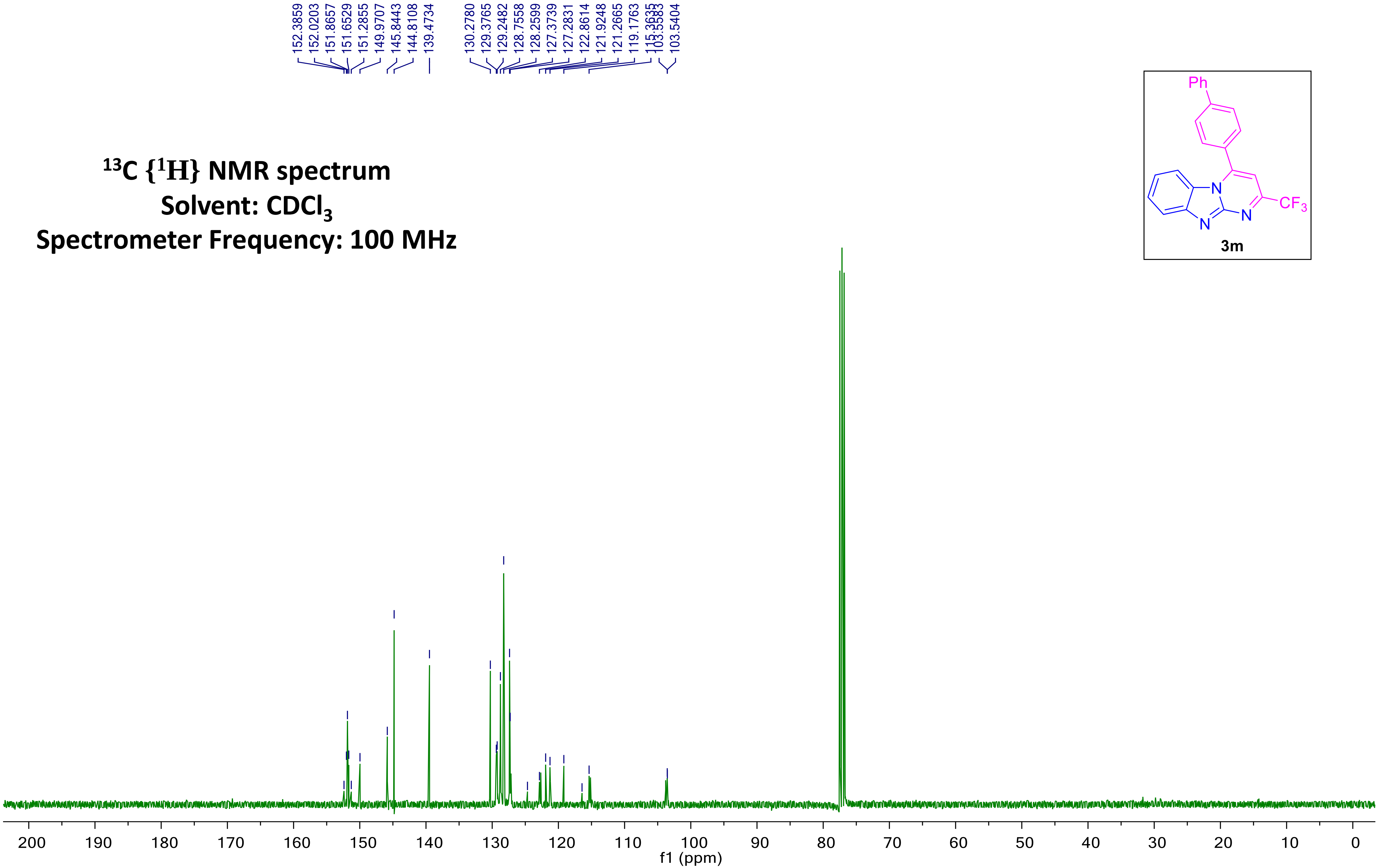

3m

^{1}H NMR spectrum
Solvent: $CDCl_3$
Spectrometer Frequency: 400 MHz

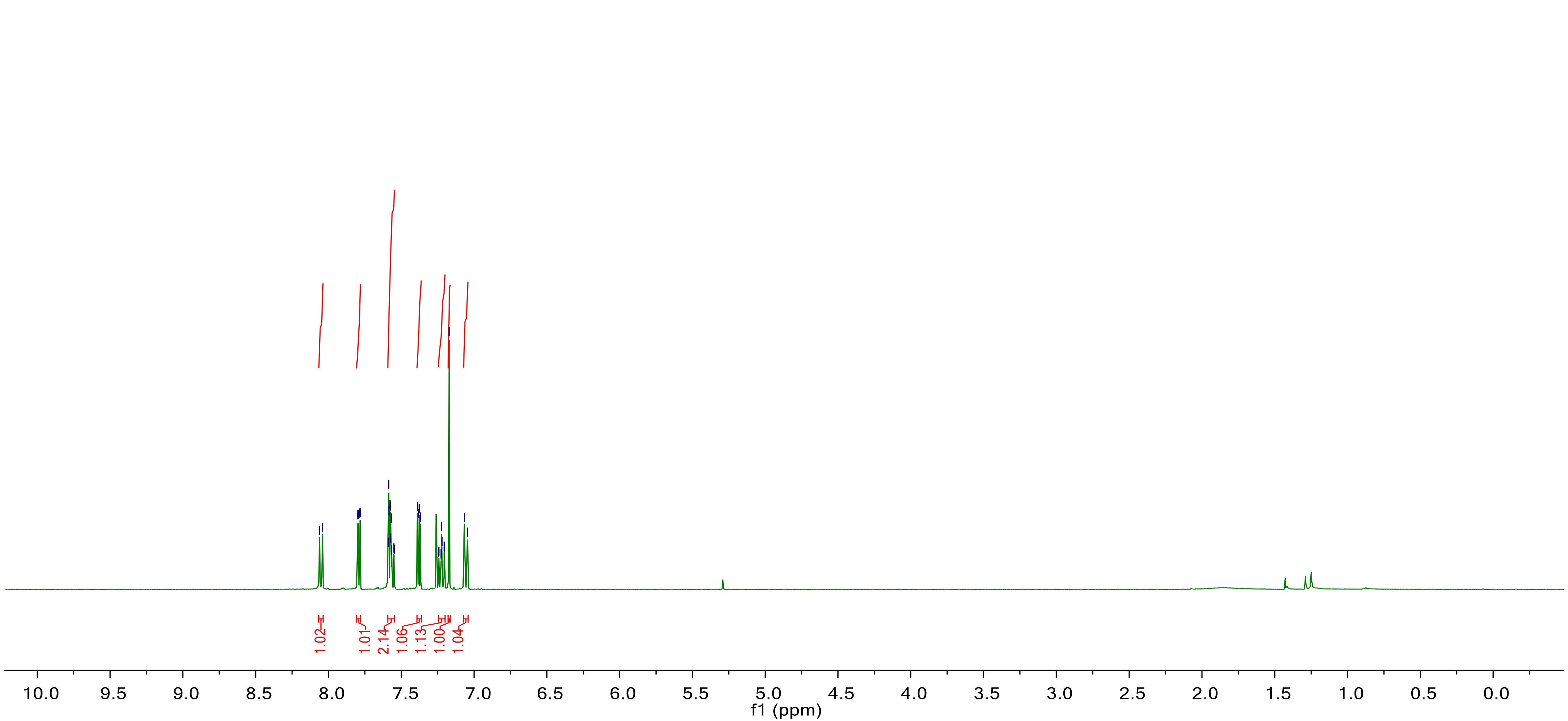

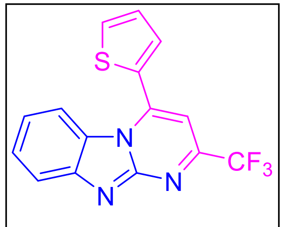

3n

$^{19}F\{^{1}H\}$ NMR spectrum
Solvent:$CDCl_3$
Spectrometer Frequency: 376 MHz

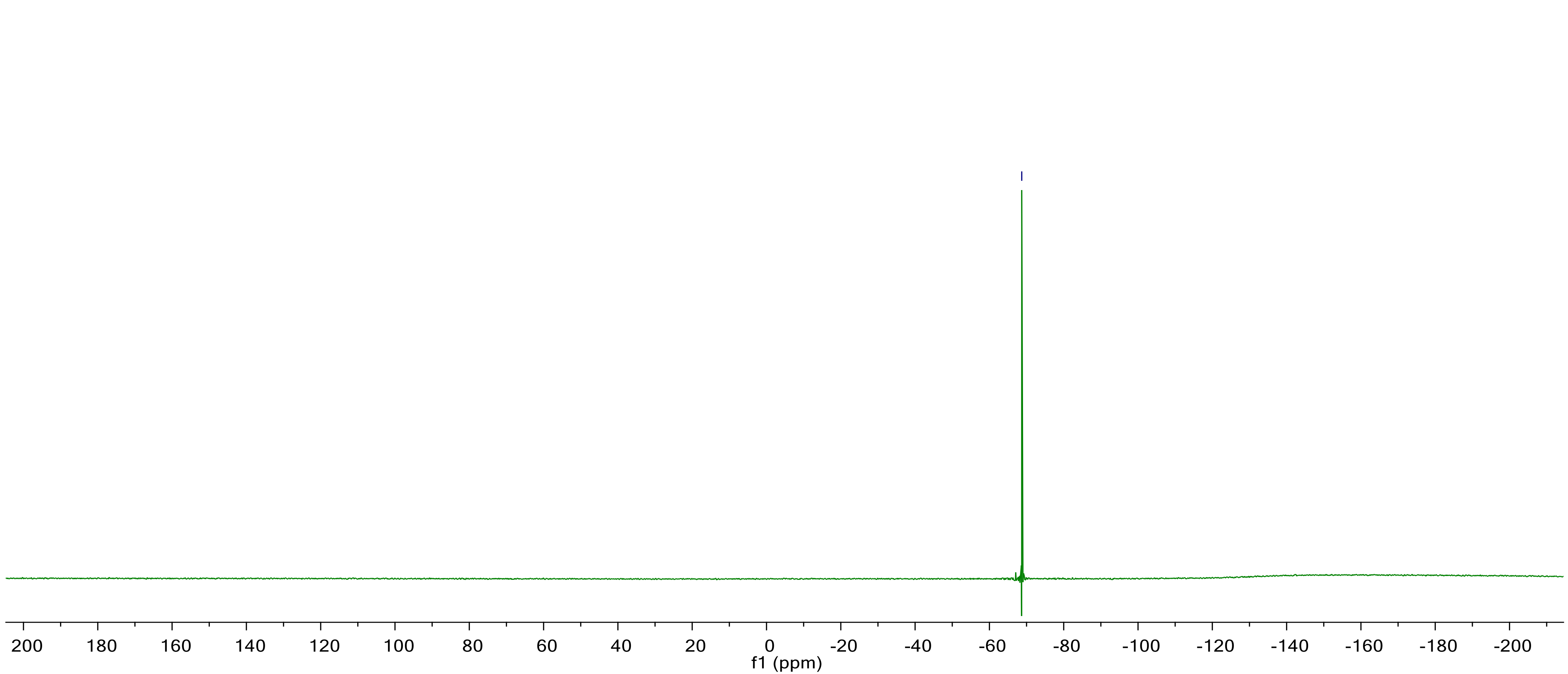

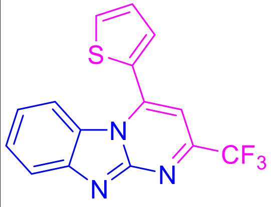

3n

^{13}C {^{1}H} NMR spectrum
Solvent: $CDCl_3$
Spectrometer Frequency: 100 MHz

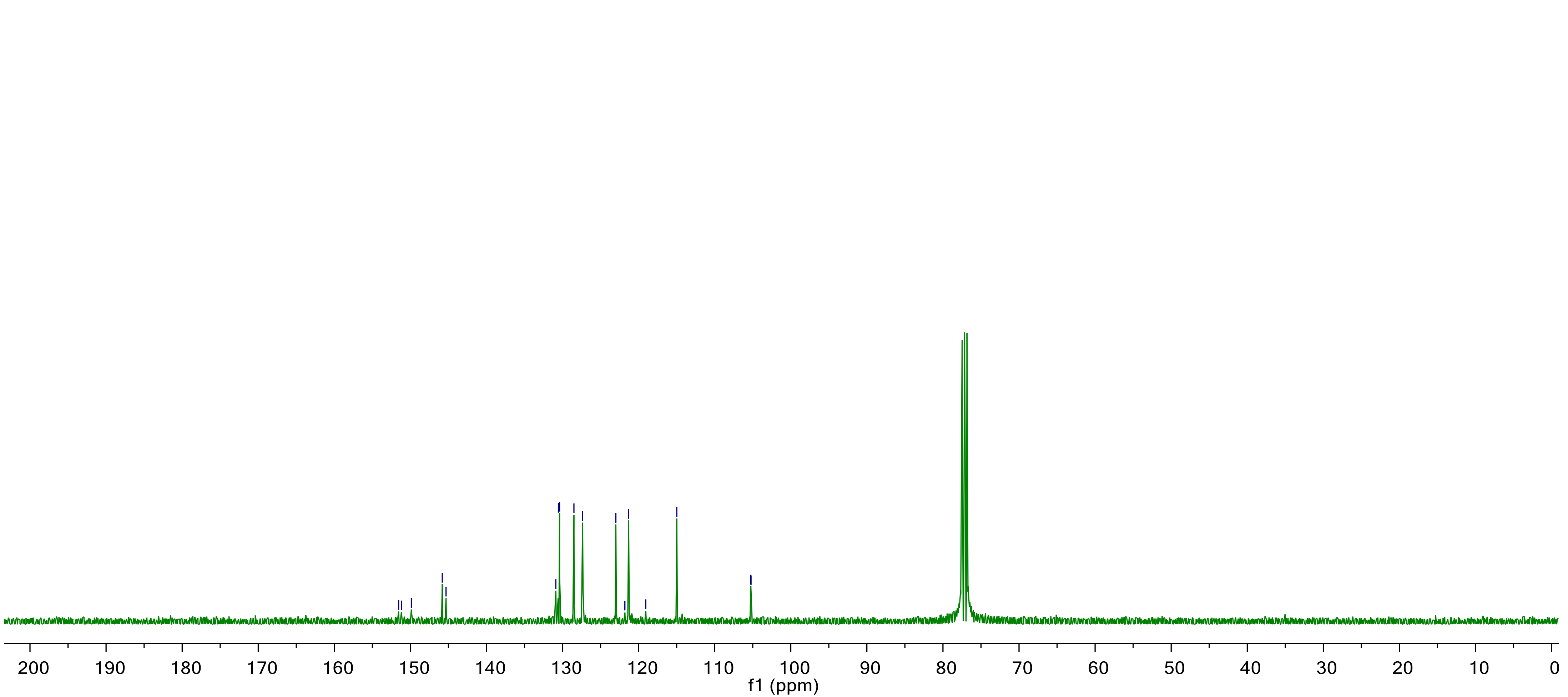

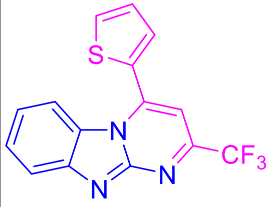

3n

^{1}H NMR spectrum
Solvent: $CDCl_3$
Spectrometer Frequency: 400 MHz

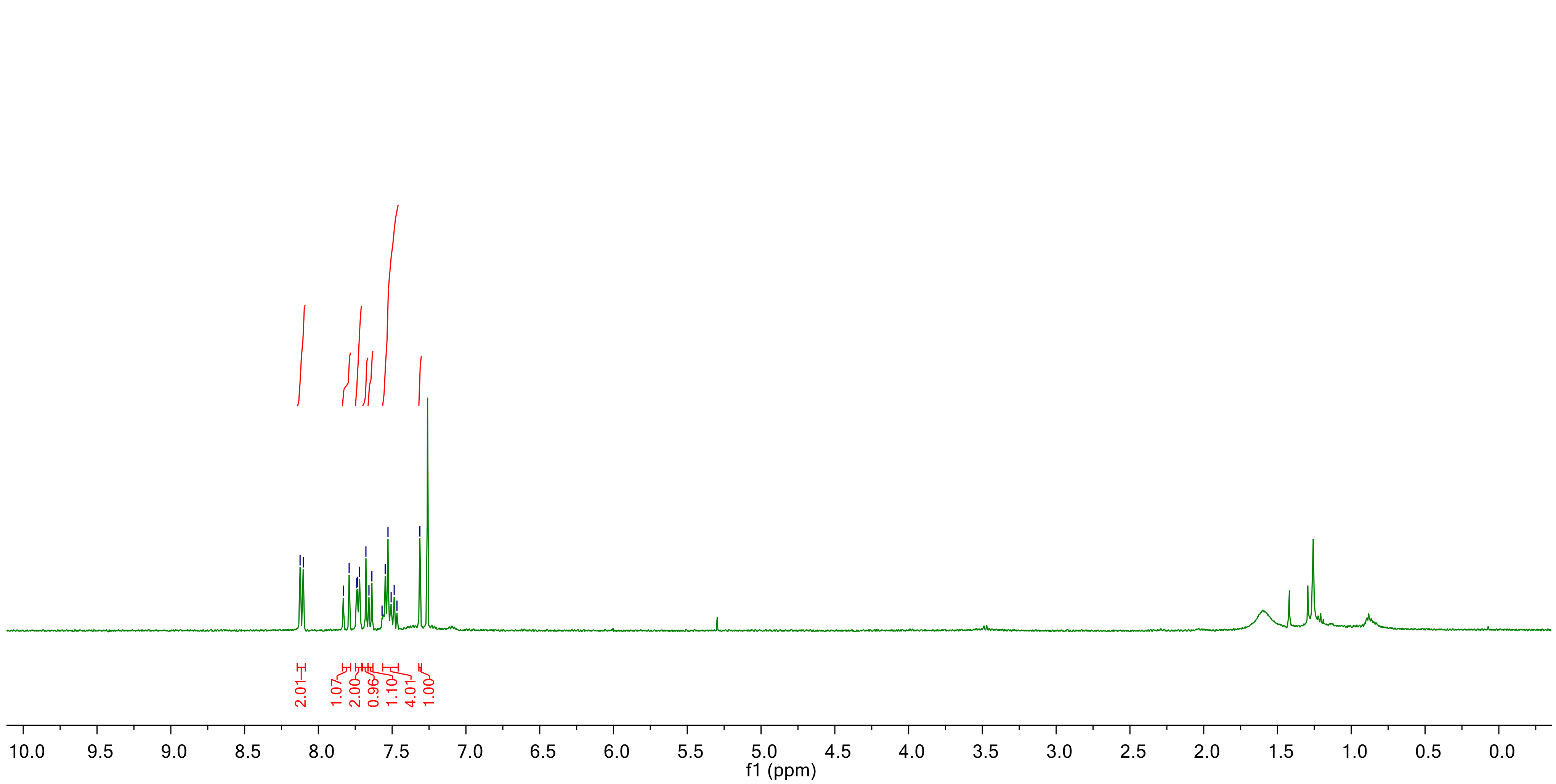

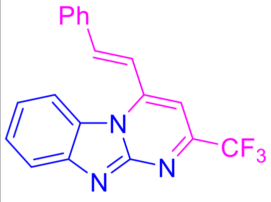

3o

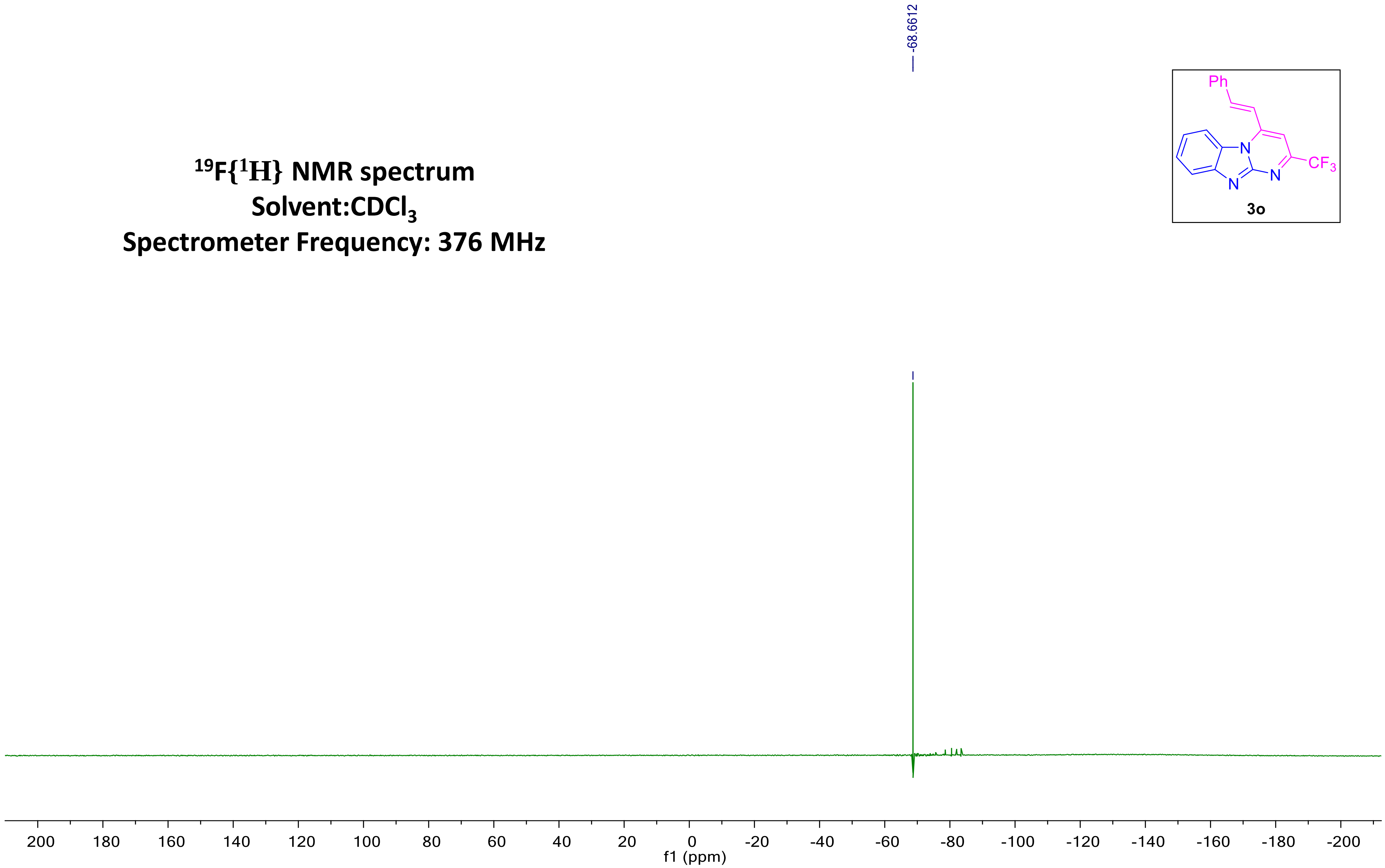

19F{1H} NMR spectrum
Solvent:CDCl3
Spectrometer Frequency: 376 MHz
-68.6612
Ph
N
N
N
CF3
3o
200
180
160
140
120
100
80
60
40
20
0
-20
-40
-60
-80
-100
-120
-140
-160
-180
-200
f1 (ppm)

$^{13}C\ \{^{1}H\}$ NMR spectrum
Solvent: $CDCl_3$
Spectrometer Frequency: 100 MHz

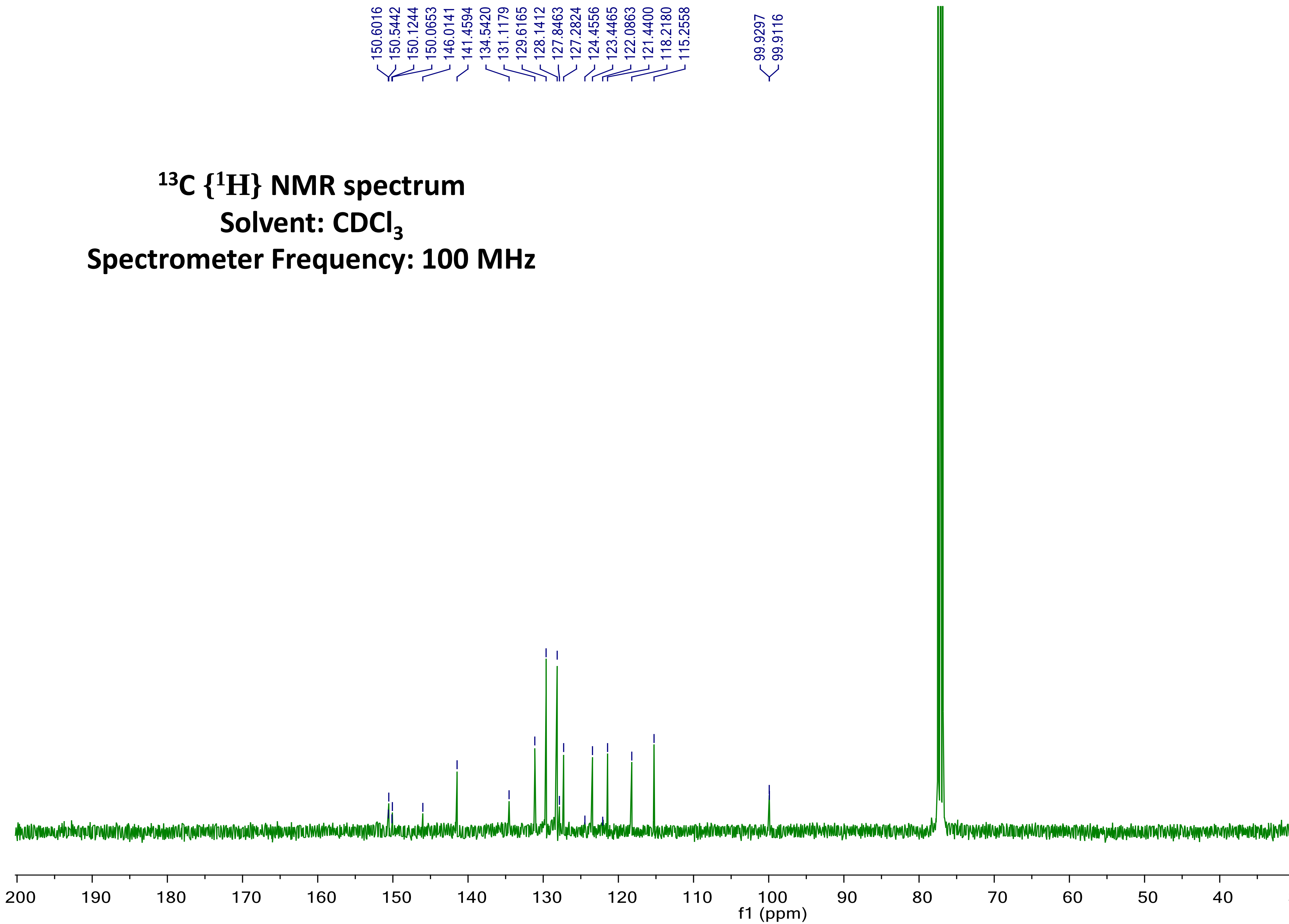

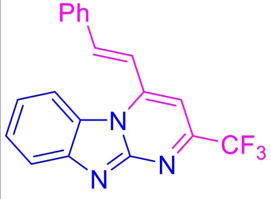

3o

^{1}H NMR spectrum
Solvent: $CDCl_3$
Spectrometer Frequency: 400 MHz

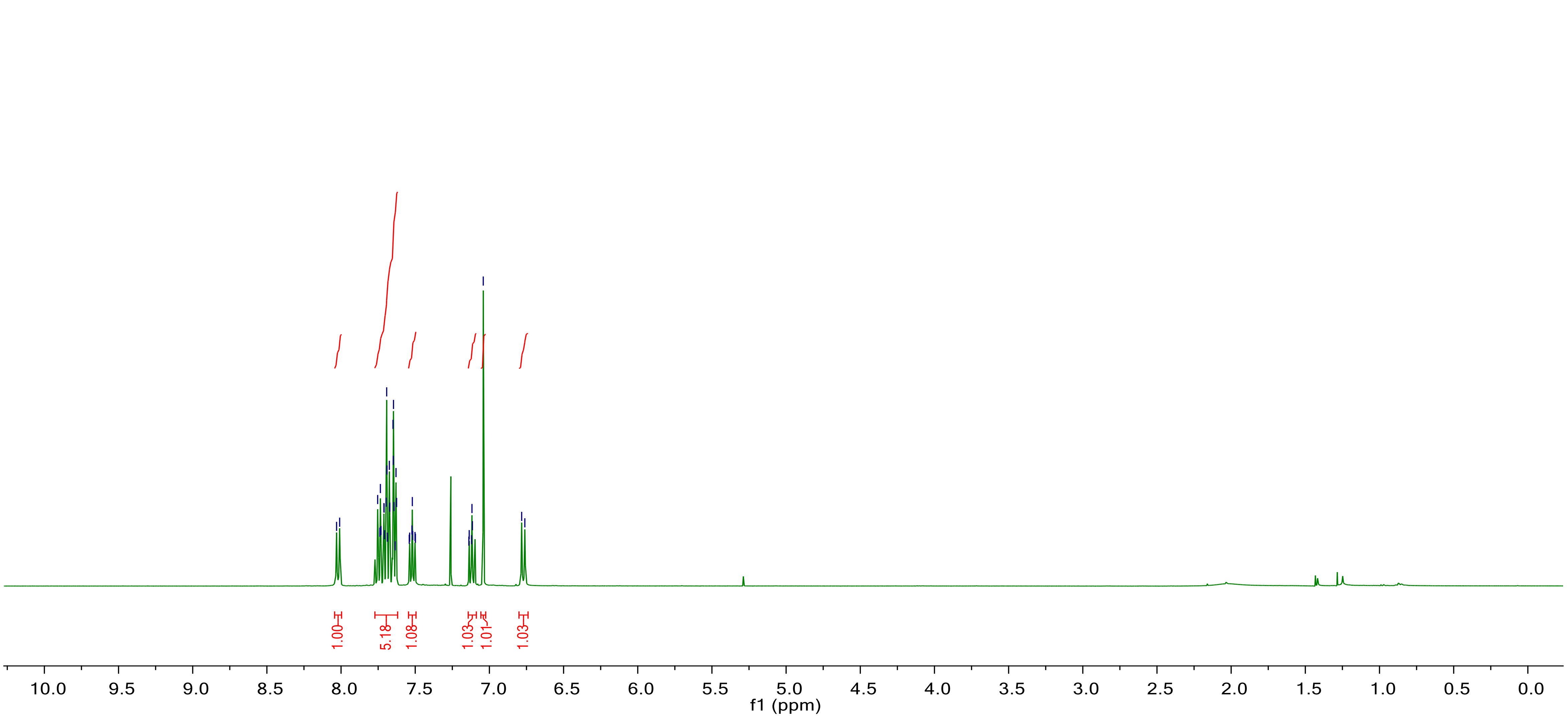

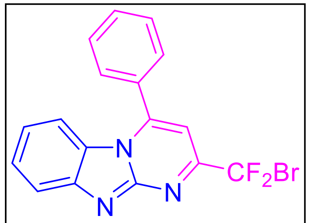

5a

$^{19}F\{^{1}H\}$ NMR spectrum
Solvent:$CDCl_3$
Spectrometer Frequency: 376 MHz

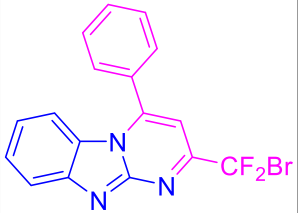

5a

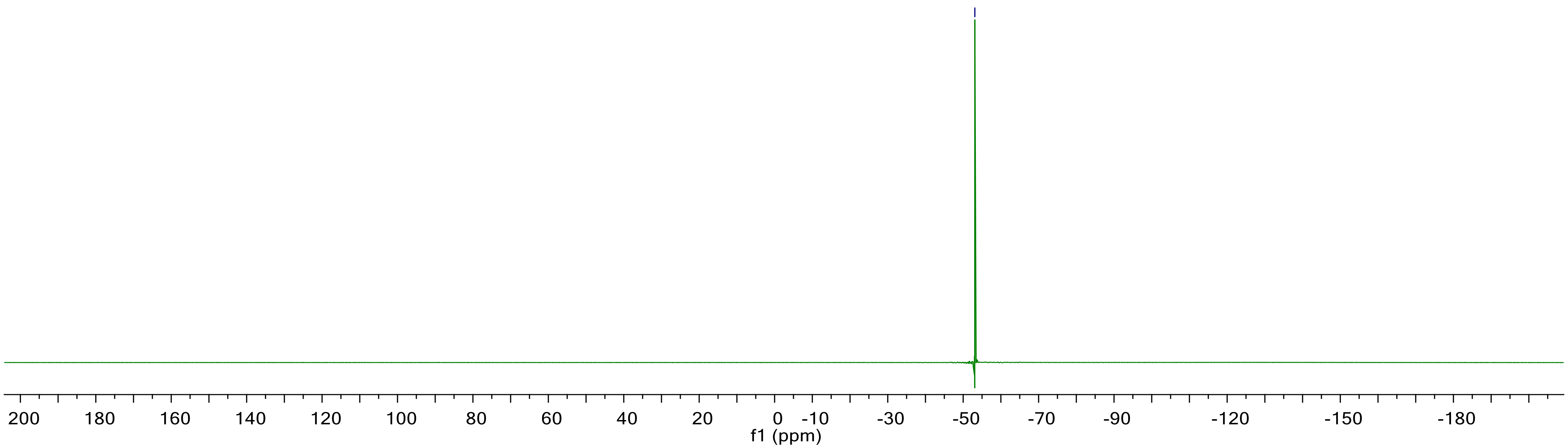

$^{13}C\ \{^{1}H\}$ NMR spectrum

Solvent: $CDCl_3$

Spectrometer Frequency: 100 MHz

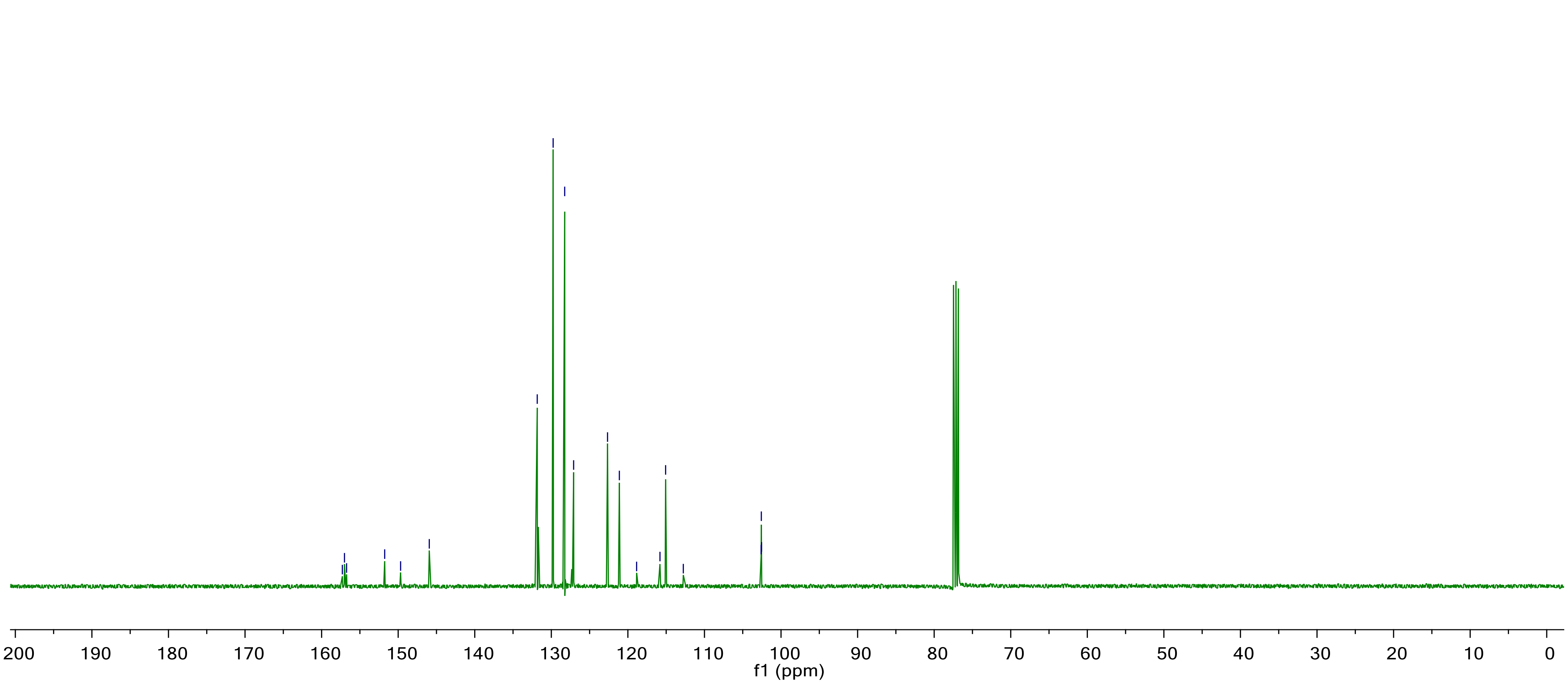

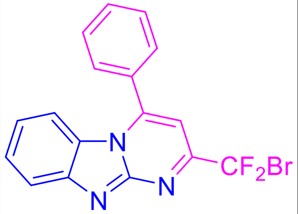

5a

^{1}H NMR spectrum

Solvent: $CDCl_3$

Spectrometer Frequency: 400 MHz

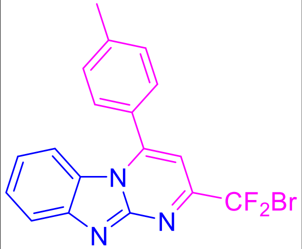

5b

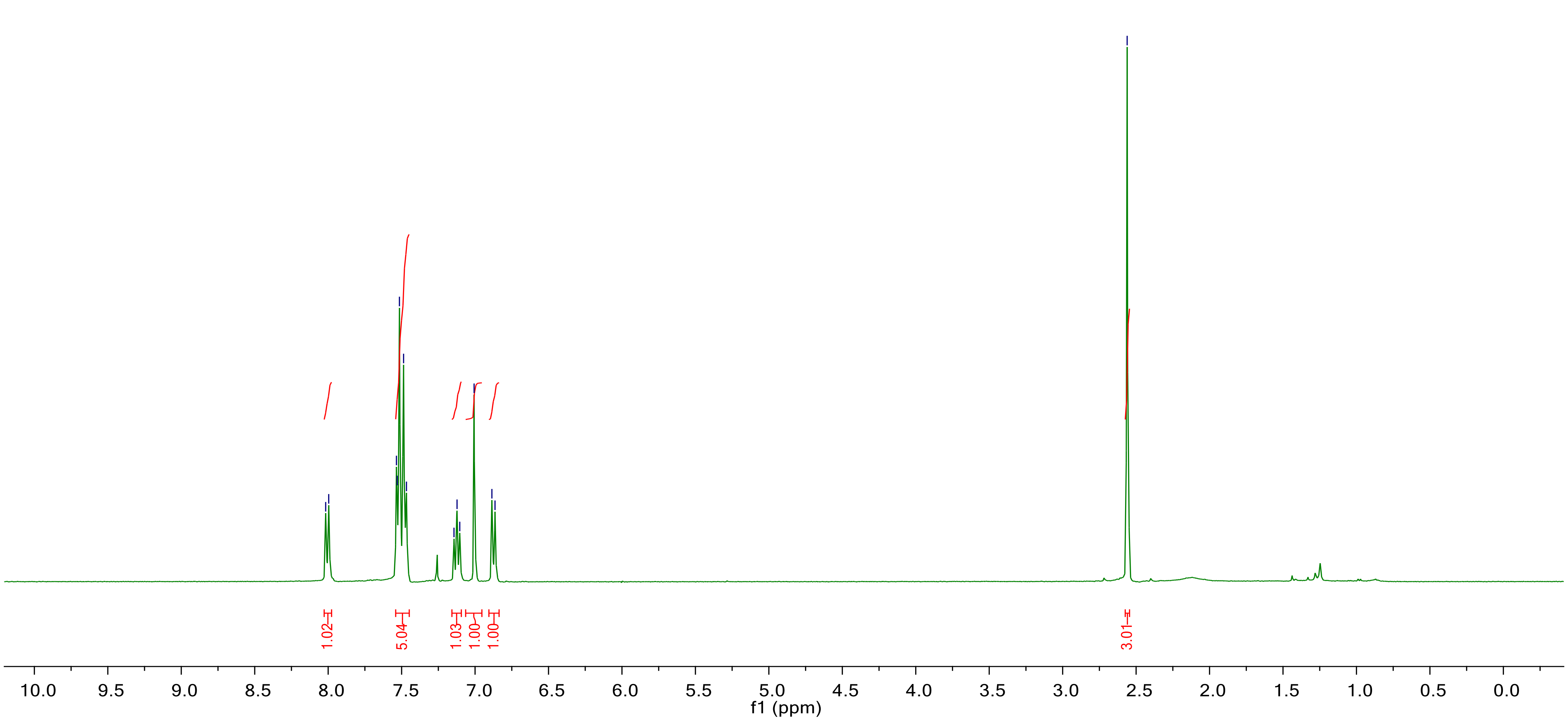

$^{19}F\{^{1}H\}$ NMR spectrum
Solvent:$CDCl_3$
Spectrometer Frequency: 376 MHz

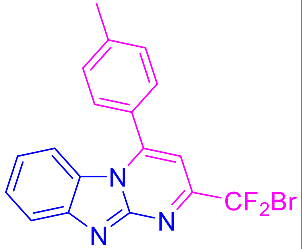

5b

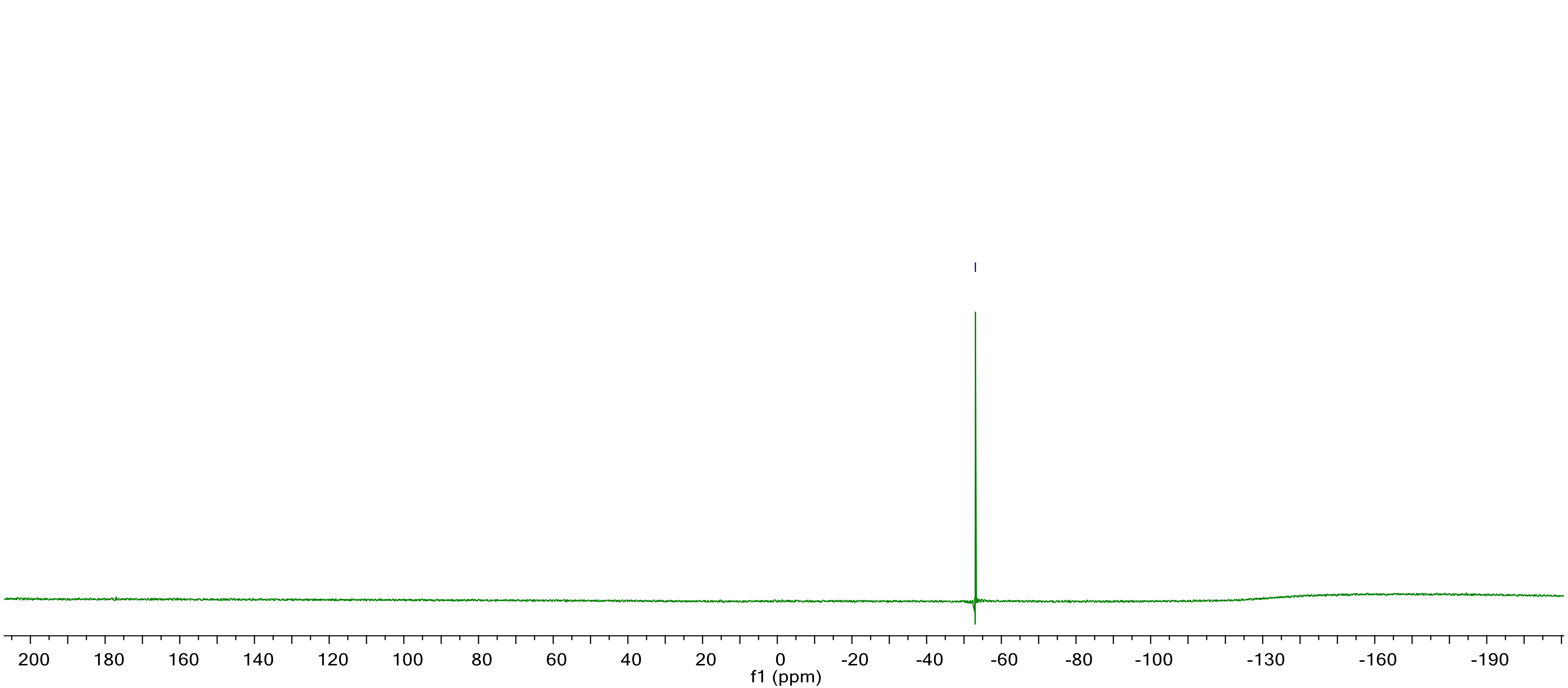

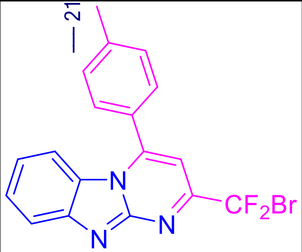

5b

^{13}C {^{1}H} NMR spectrum
Solvent: $CDCl_3$
Spectrometer Frequency: 100 MHz

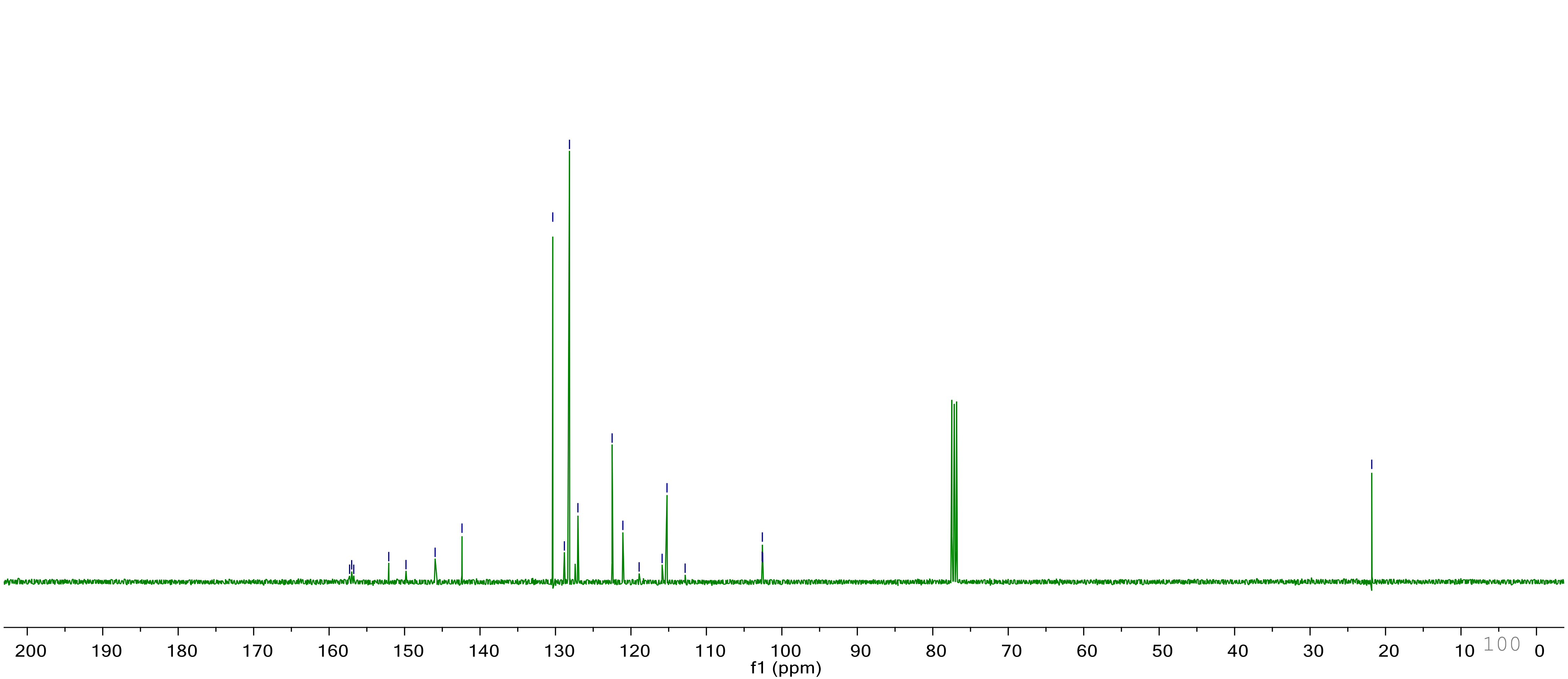

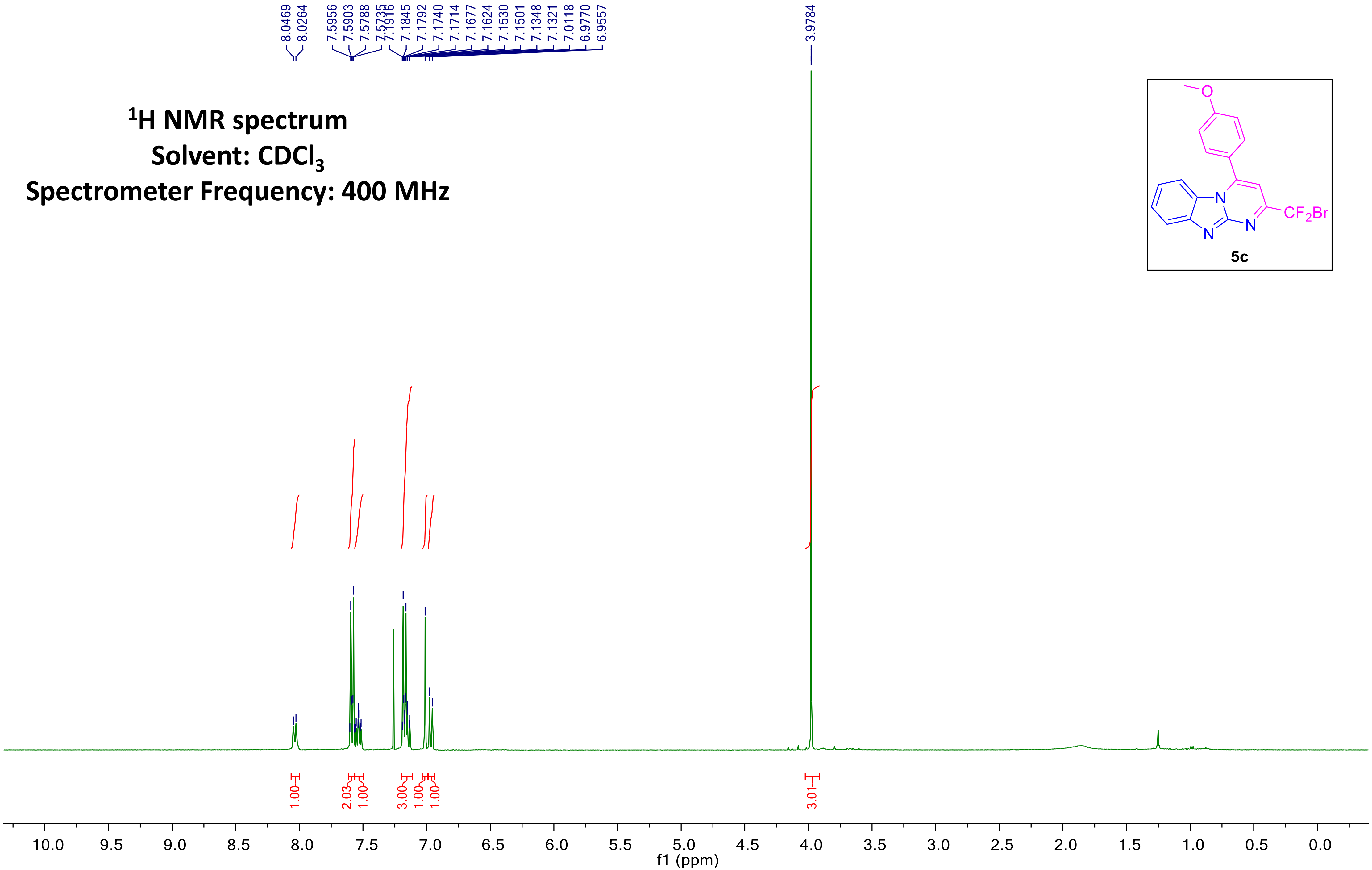
1H NMR spectrum
Solvent: CDCl3
Spectrometer Frequency: 400 MHz
8.0469
8.0264
7.5956
7.5903
7.5788
7.5735
7.1916
7.1845
7.1792
7.1740
7.1714
7.1677
7.1624
7.1530
7.1501
7.1348
7.1321
7.0118
6.9770
6.9557
3.9784
O
N
N
N
CF2Br
5c
1.00
2.03
1.00
3.00
1.00
1.00
3.01
10.0
9.5
9.0
8.5
8.0
7.5
7.0
6.5
6.0
5.5
5.0
4.5
4.0
3.5
3.0
2.5
2.0
1.5
1.0
0.5
0.0
f1 (ppm)

$^{19}F\{^{1}H\}$ NMR spectrum
Solvent:$CDCl_3$
Spectrometer Frequency: 376 MHz

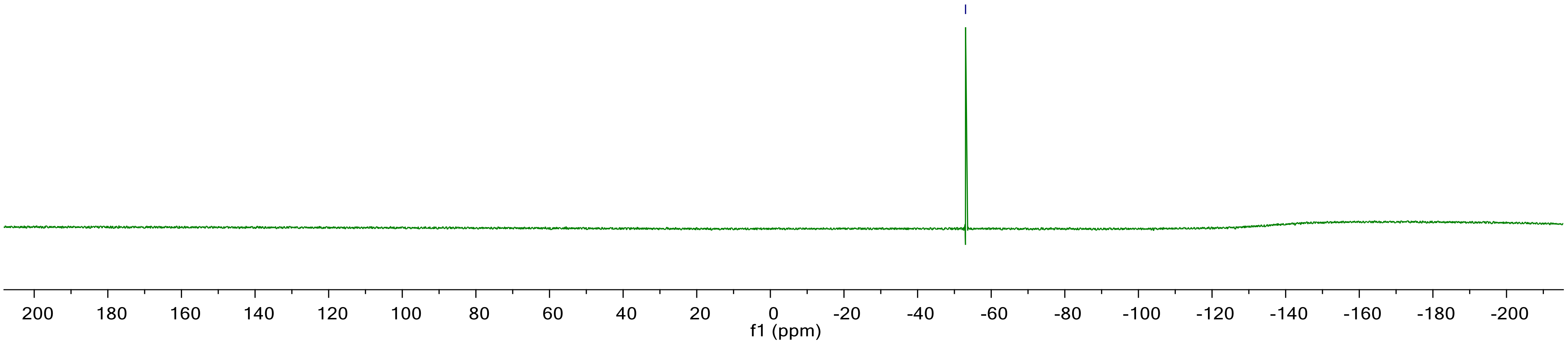

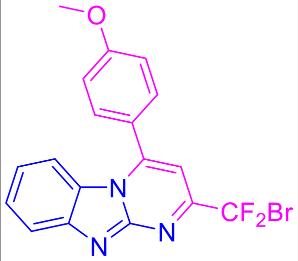

5c

$^{13}C\ \{^{1}H\}$ NMR spectrum
Solvent: $CDCl_3$
Spectrometer Frequency: 100 MHz

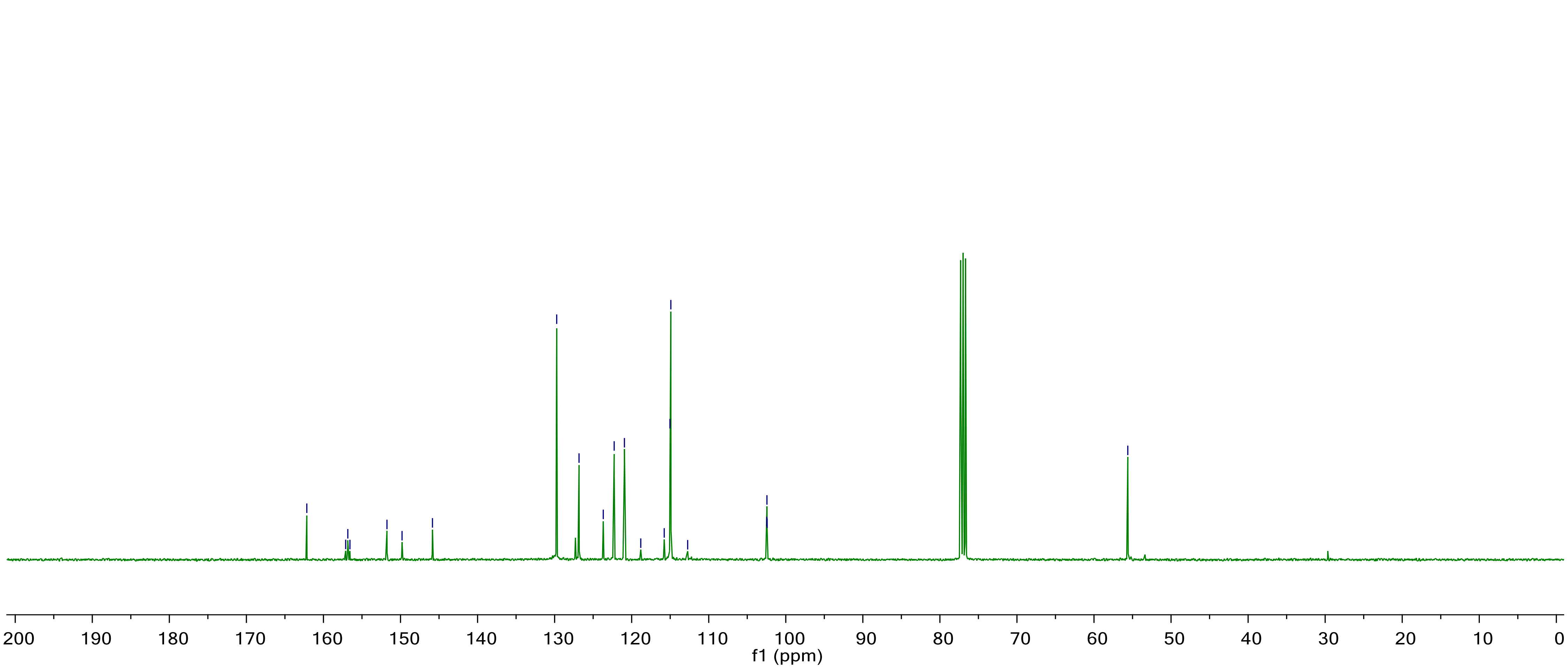

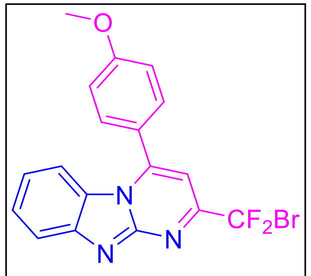

5c

^{1}H NMR spectrum
Solvent: $CDCl_3$
Spectrometer Frequency: 400 MHz

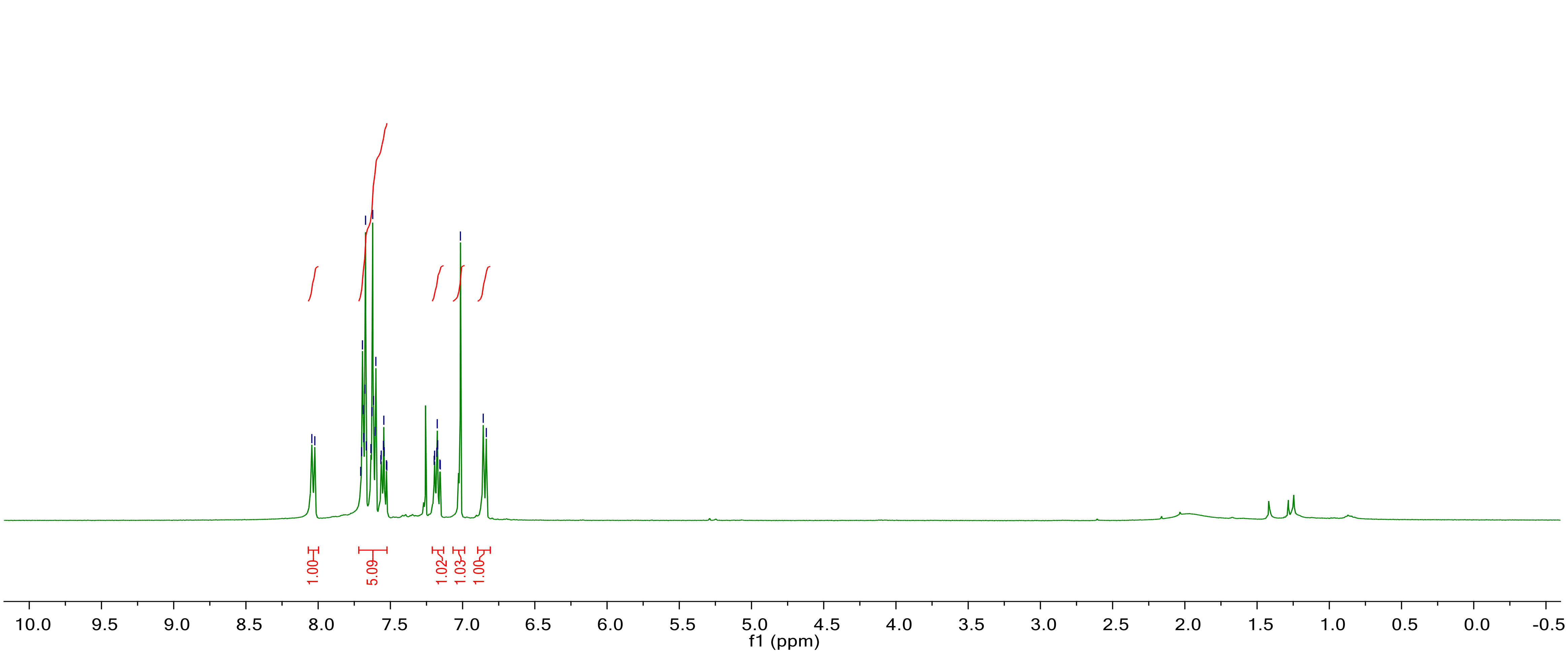

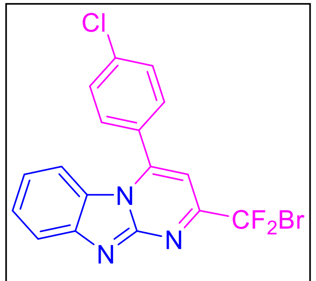

5d

$^{19}F\{^{1}H\}$ NMR spectrum
Solvent:$CDCl_3$
Spectrometer Frequency: 376 MHz

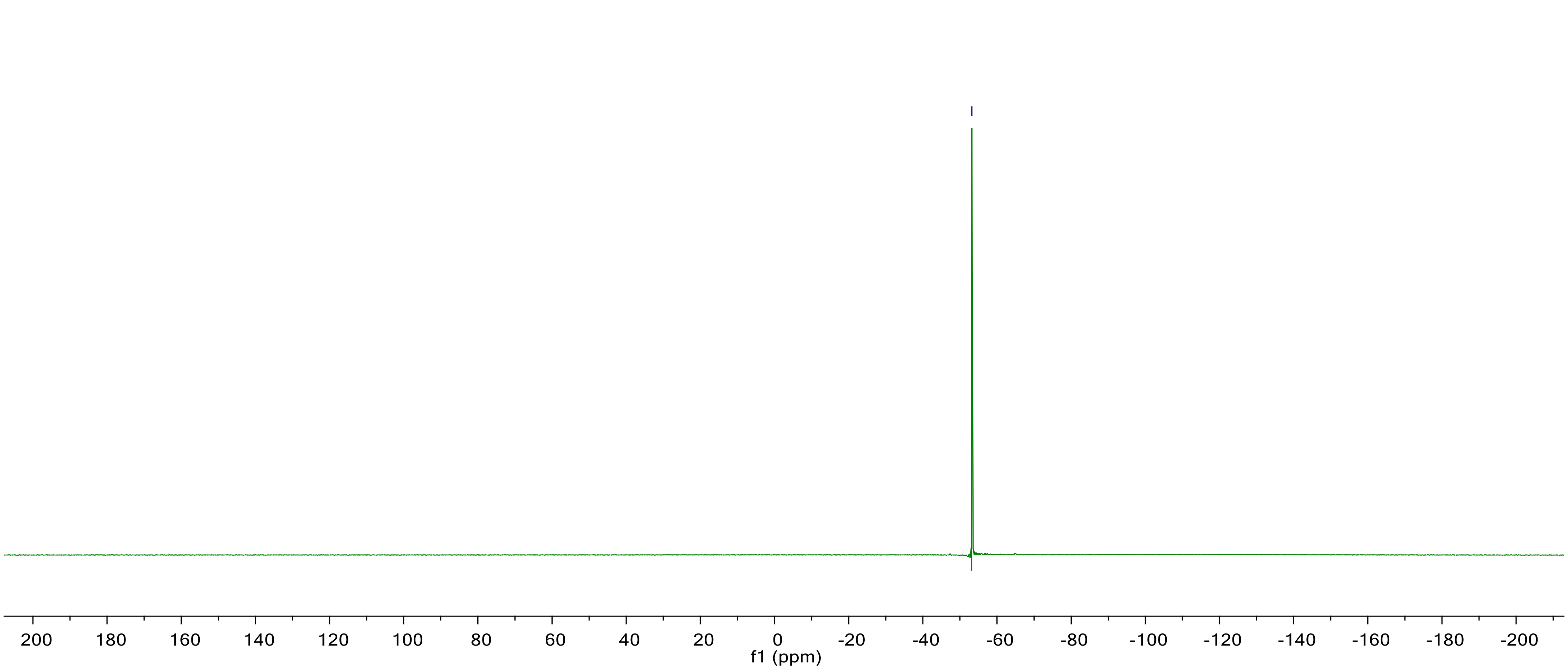

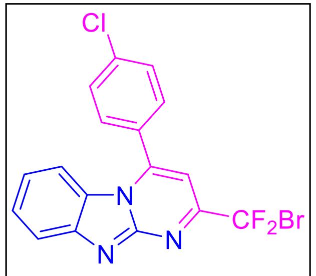

5d

^{13}C {^{1}H} NMR spectrum
Solvent: $CDCl_3$
Spectrometer Frequency: 100 MHz

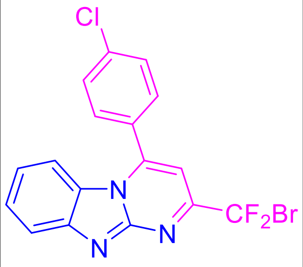

5d

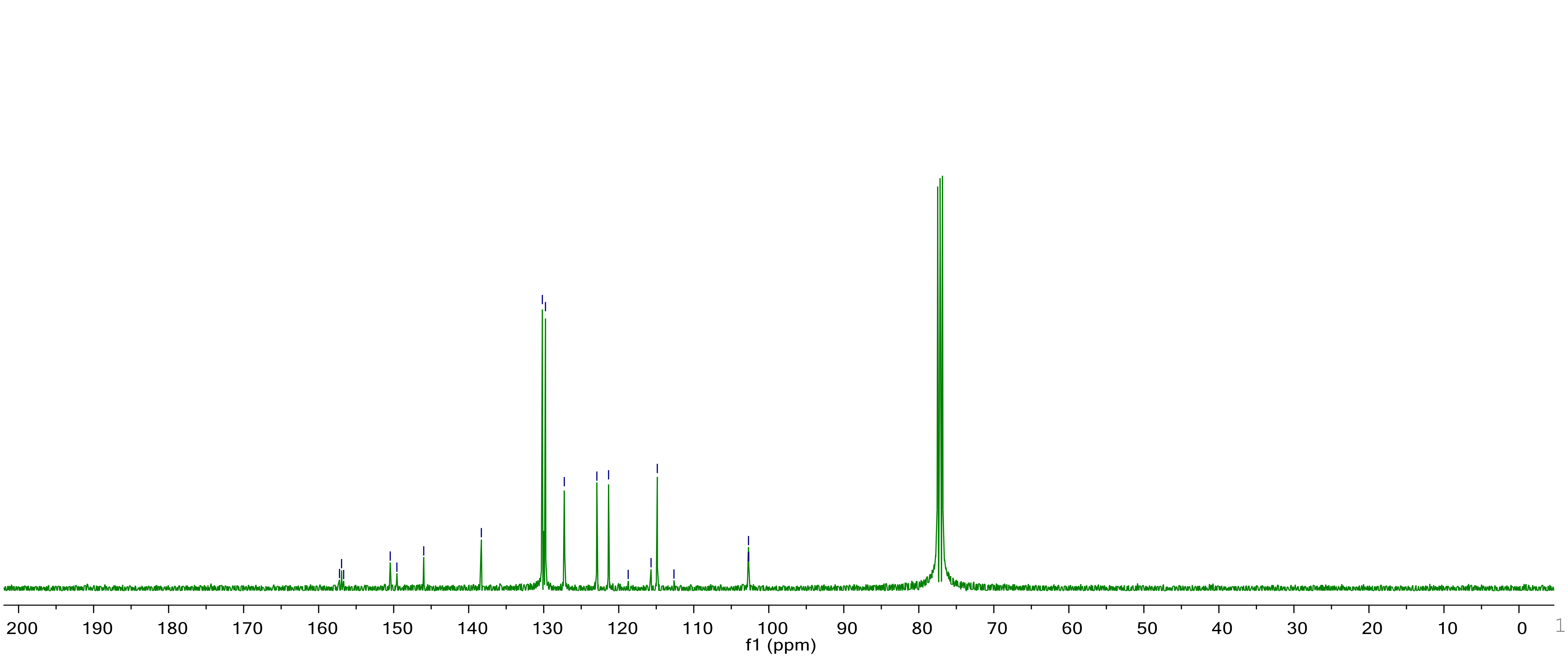

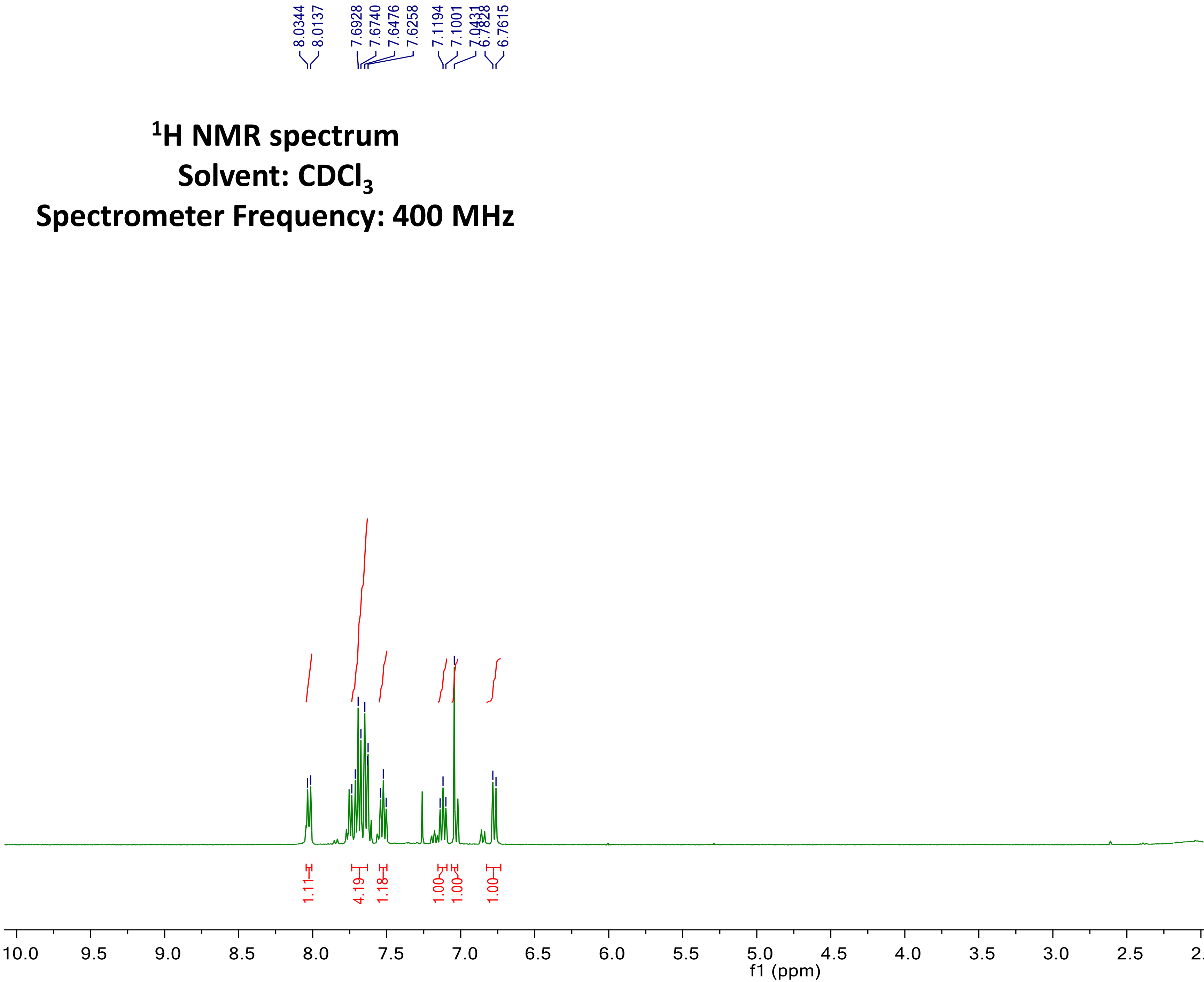

^{1}H NMR spectrum

Solvent: $CDCl_3$

Spectrometer Frequency: 400 MHz

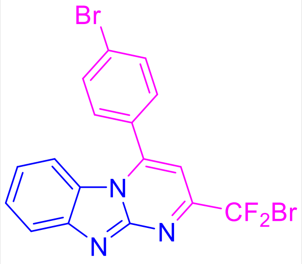

5e

$^{19}F\{^1H\}$ NMR spectrum
Solvent:$CDCl_3$
Spectrometer Frequency: 376 MHz

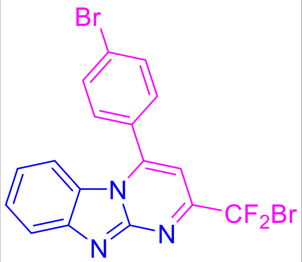

5e

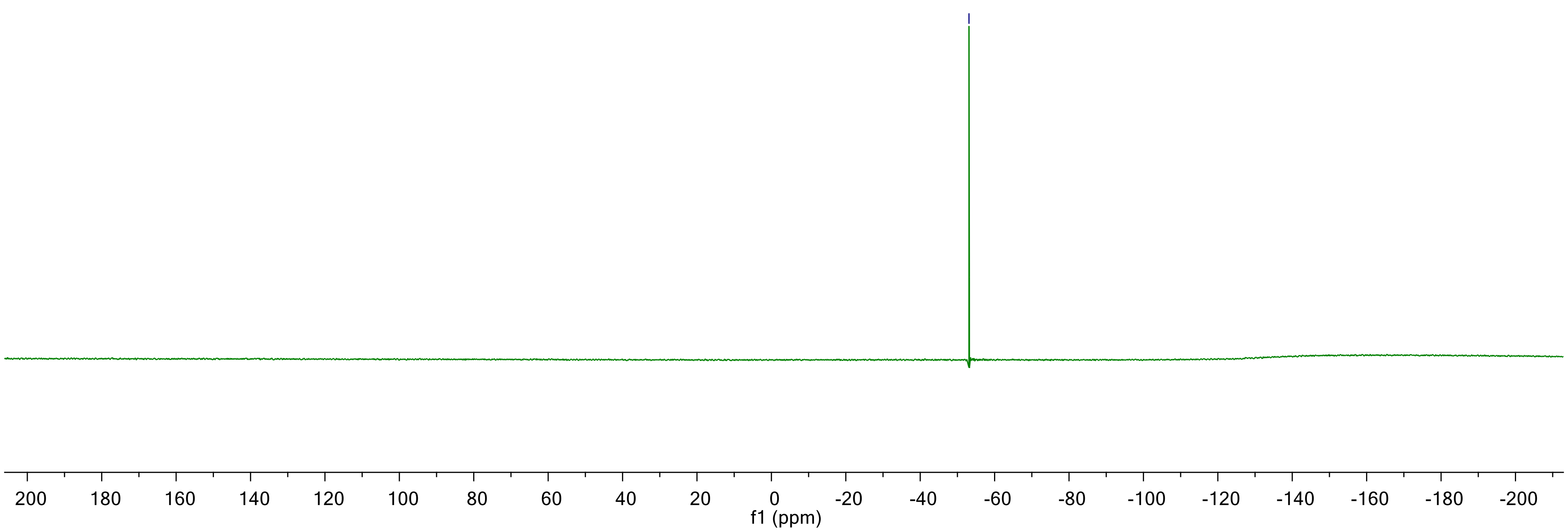

^{13}C {^{1}H} NMR spectrum
Solvent: $CDCl_3$
Spectrometer Frequency: 100 MHz

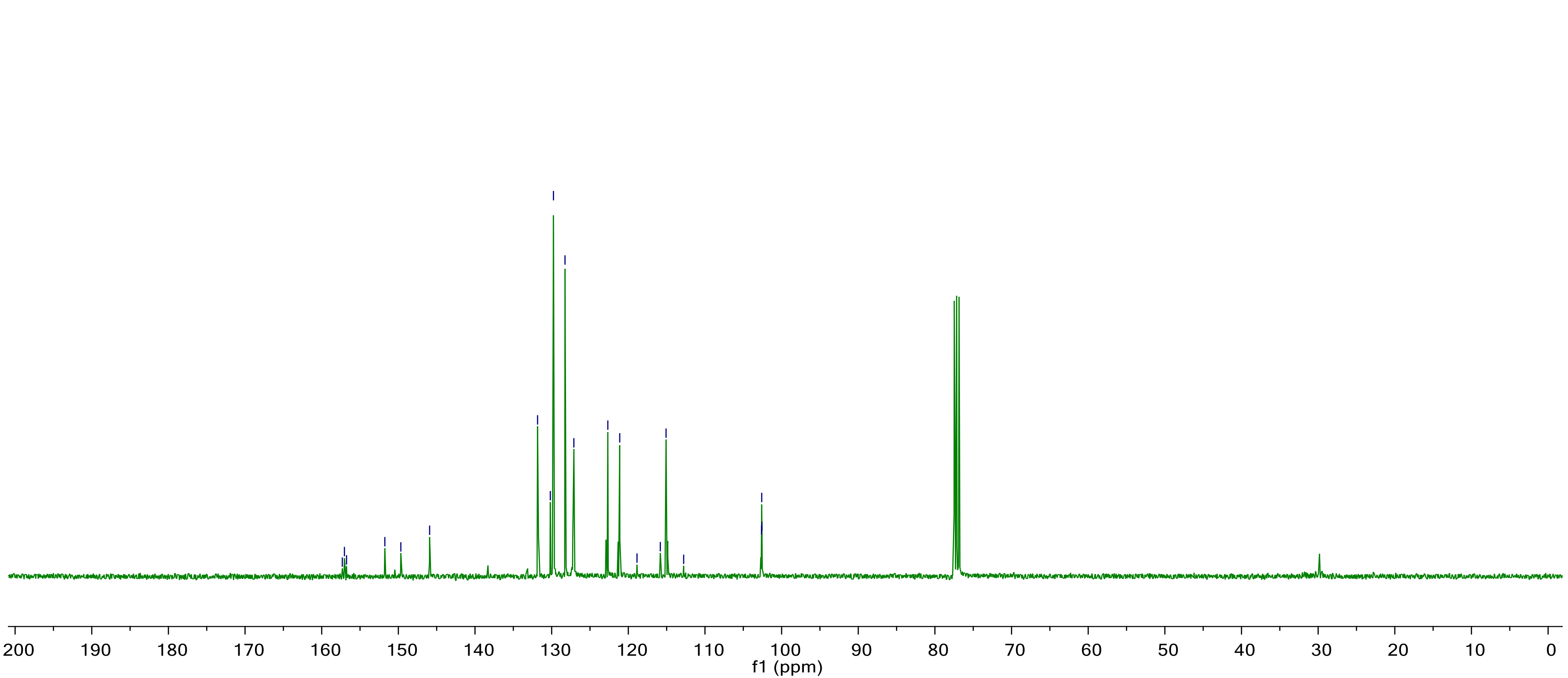

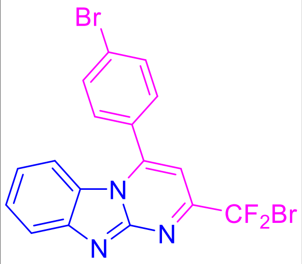

5e

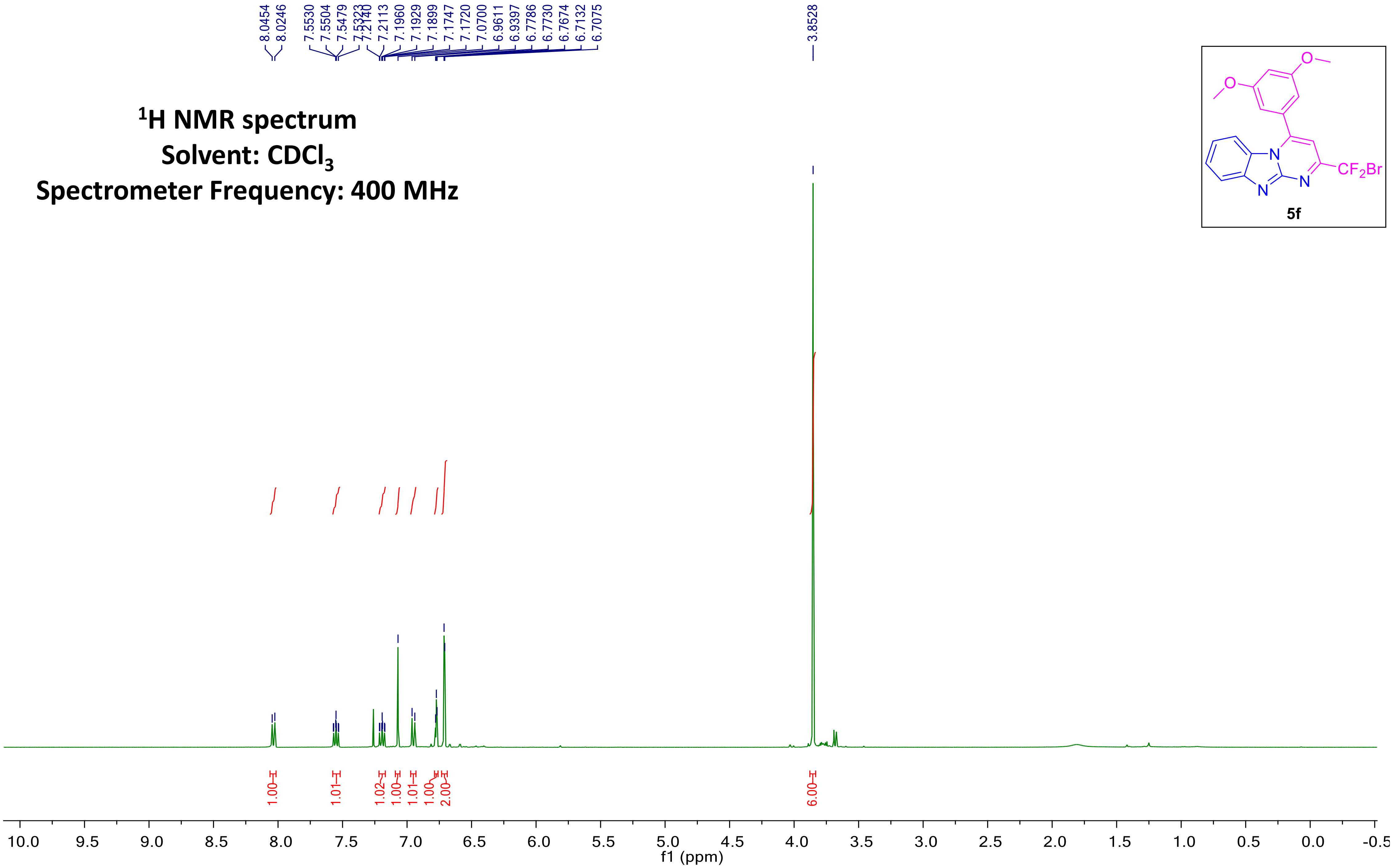

1H NMR spectrum
Solvent: CDCl3
Spectrometer Frequency: 400 MHz
8.0454
8.0246
7.5530
7.5504
7.5479
7.5323
7.2140
7.2113
7.1960
7.1929
7.1899
7.1747
7.1720
7.0700
6.9611
6.9397
6.7786
6.7730
6.7674
6.7132
6.7075
3.8528
1.00
1.01
1.02
1.00
1.01
1.00
2.00
6.00
CF2Br
N
N
N
O
O
5f
10.0
9.5
9.0
8.5
8.0
7.5
7.0
6.5
6.0
5.5
5.0
4.5
4.0
3.5
3.0
2.5
2.0
1.5
1.0
0.5
0.0
-0.5
f1 (ppm)

$^{19}F\{^1H\}$ NMR spectrum
Solvent:$CDCl_3$
Spectrometer Frequency: 376 MHz

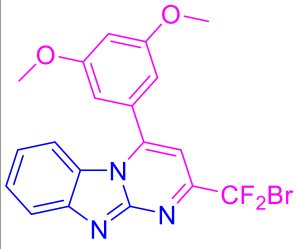

5f

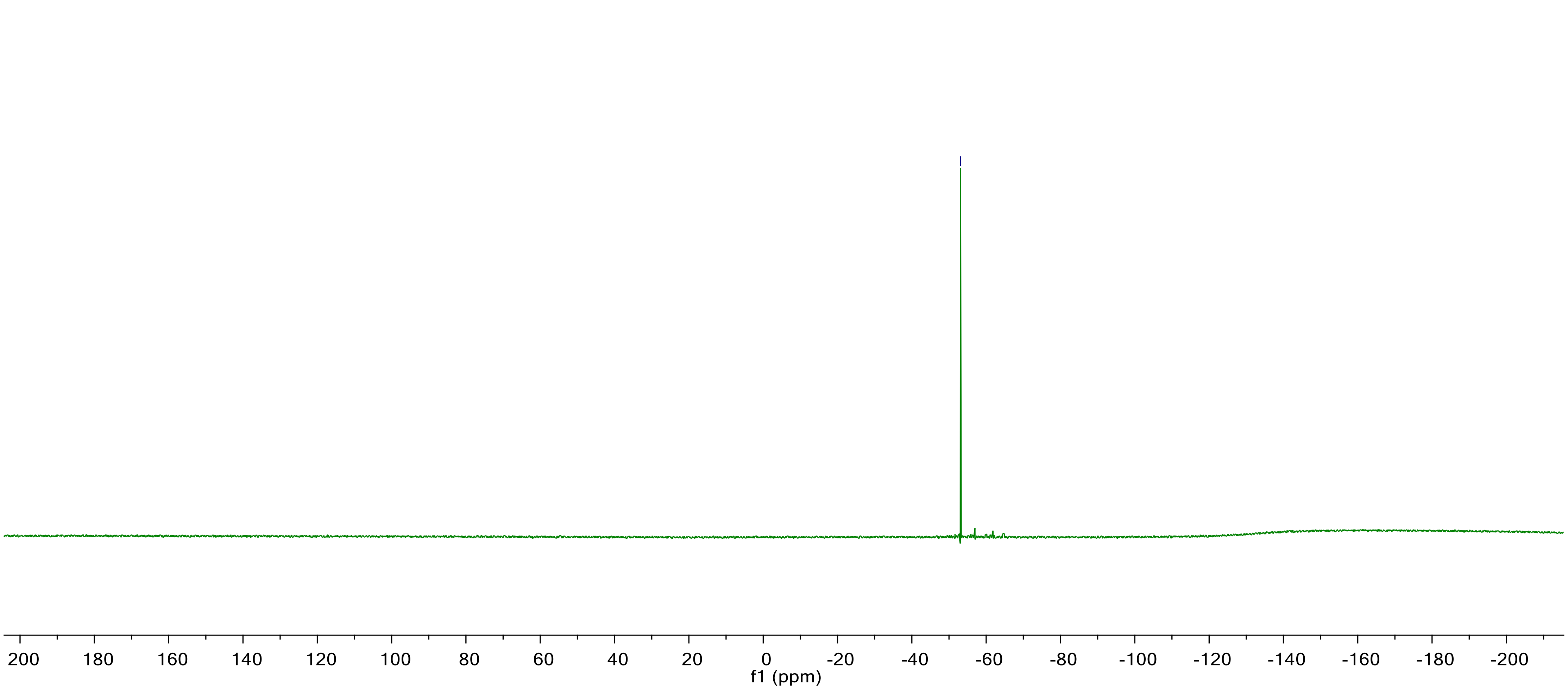

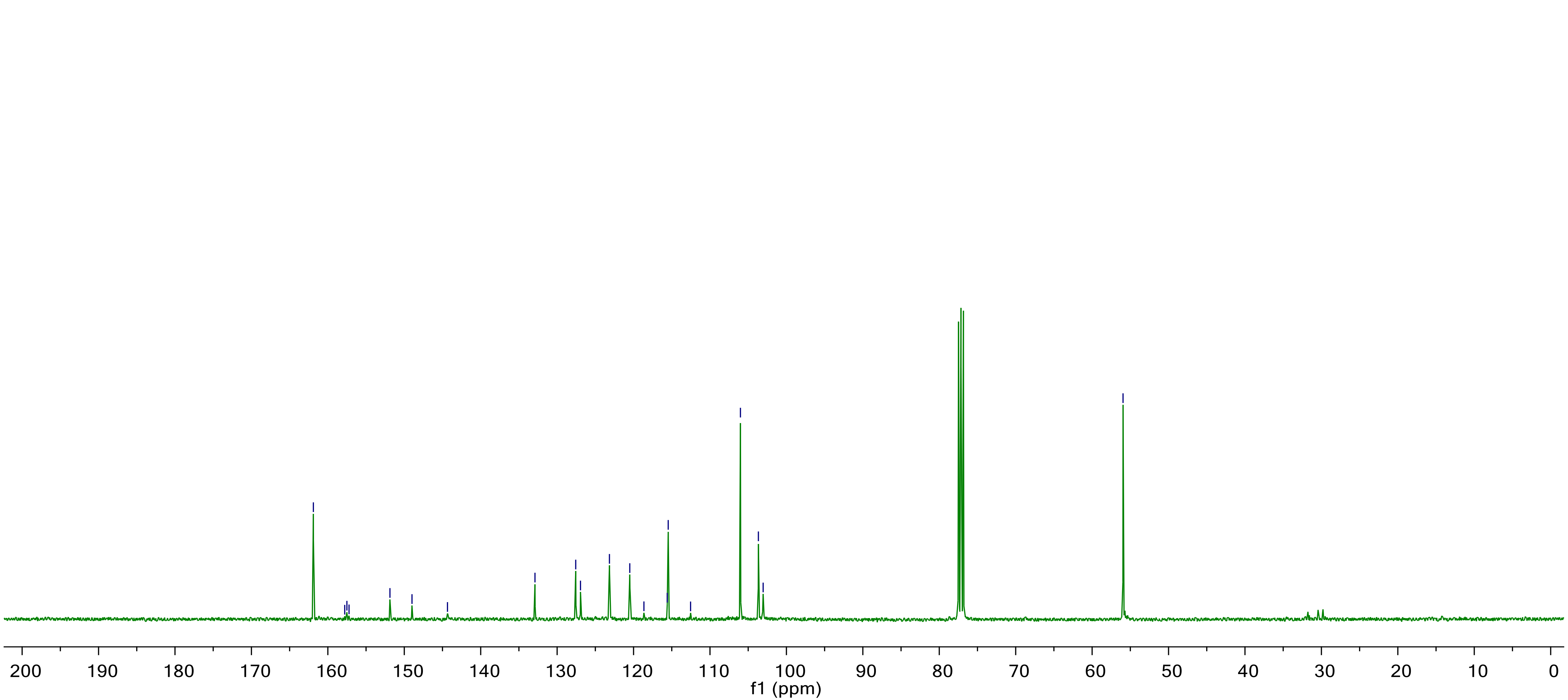

$^{13}C\ \{^{1}H\}$ NMR spectrum
Solvent: $CDCl_3$
Spectrometer Frequency: 100 MHz

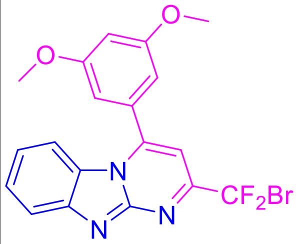

5f

^{1}H NMR spectrum
Solvent: $CDCl_3$
Spectrometer Frequency: 400 MHz

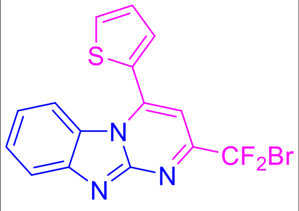

5g

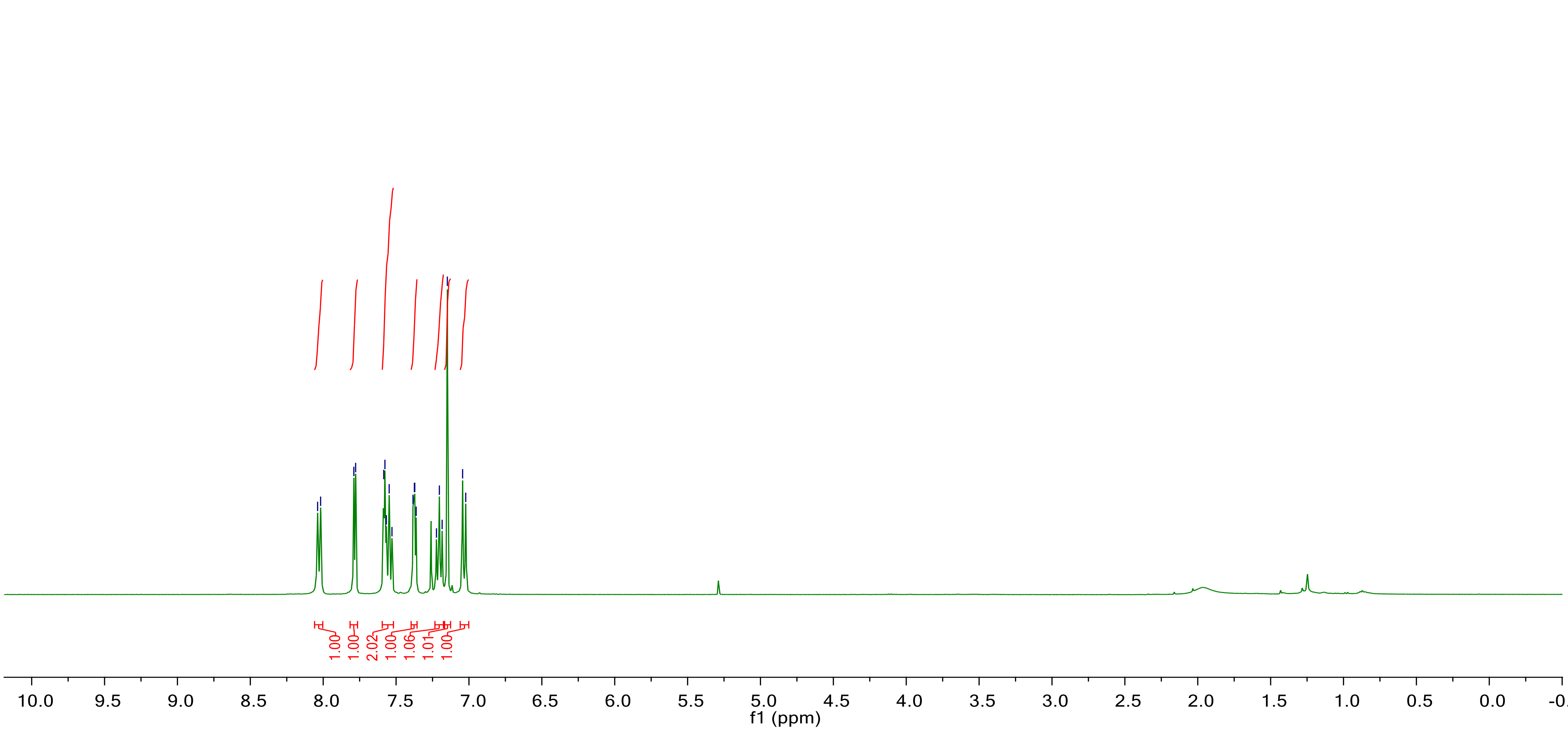

$^{19}F\{^1H\}$ NMR spectrum
Solvent:$CDCl_3$
Spectrometer Frequency: 376 MHz

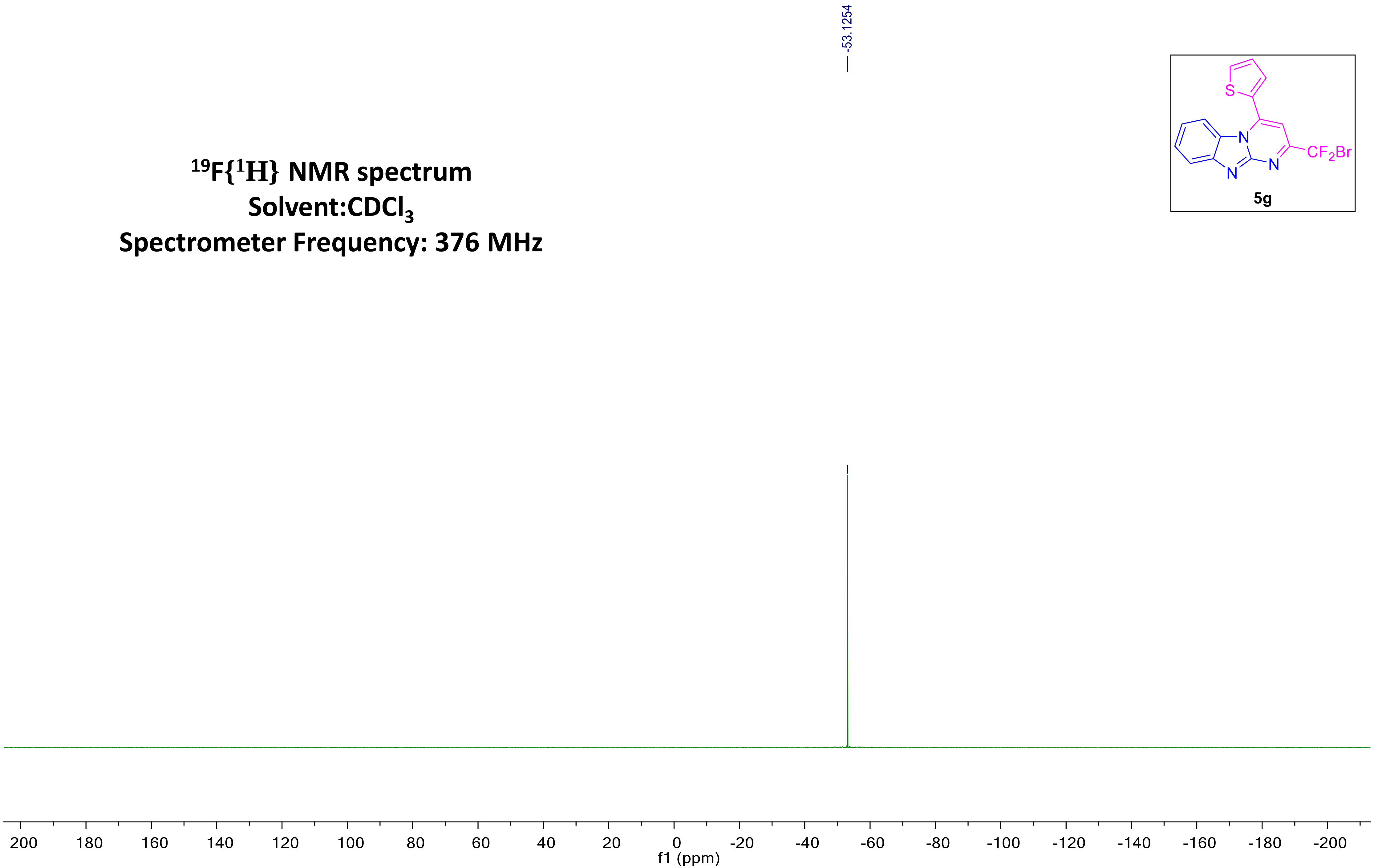

5g

$^{13}C\ \{^1H\}$ NMR spectrum
Solvent: $CDCl_3$
Spectrometer Frequency: 100 MHz

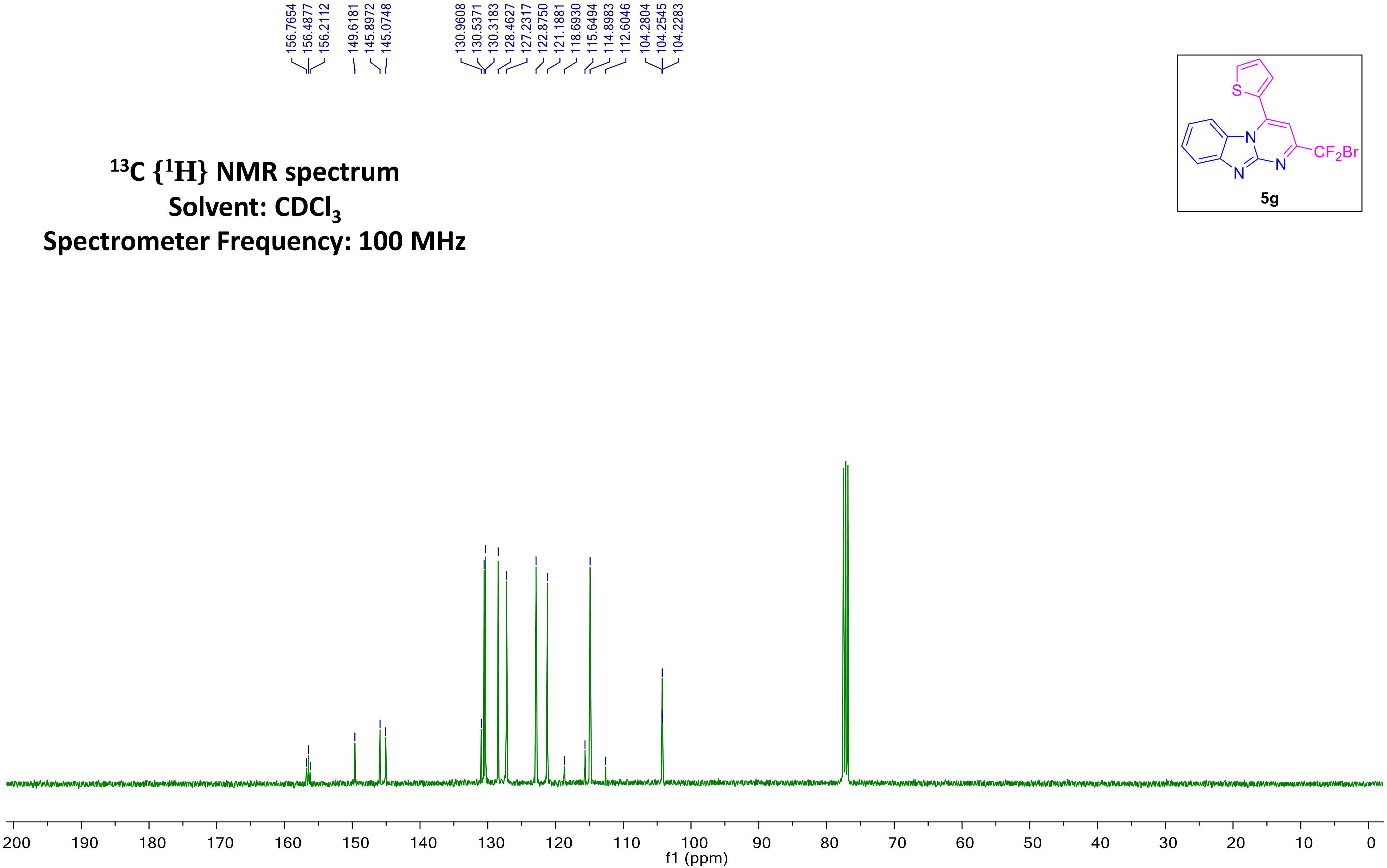

5g

^{1}H NMR spectrum
Solvent: $CDCl_3$
Spectrometer Frequency: 400 MHz

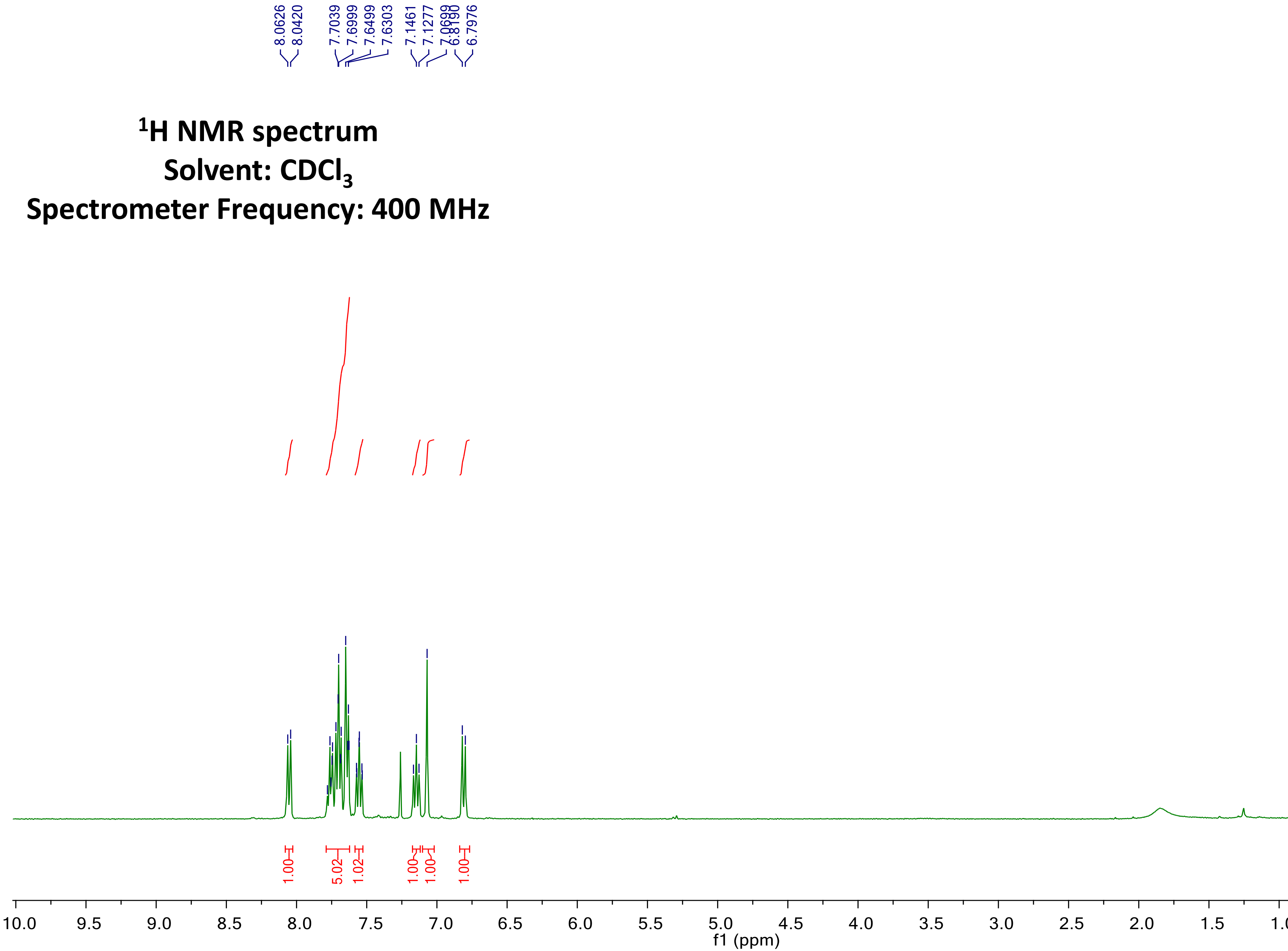

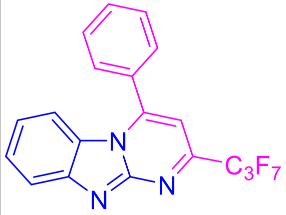

5i

$^{19}F\{^1H\}$ **NMR spectrum**

Solvent:$CDCl_3$

Spectrometer Frequency: 376 MHz

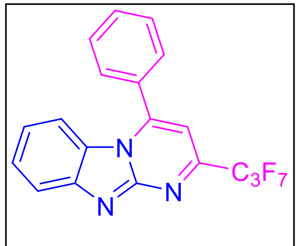

5i

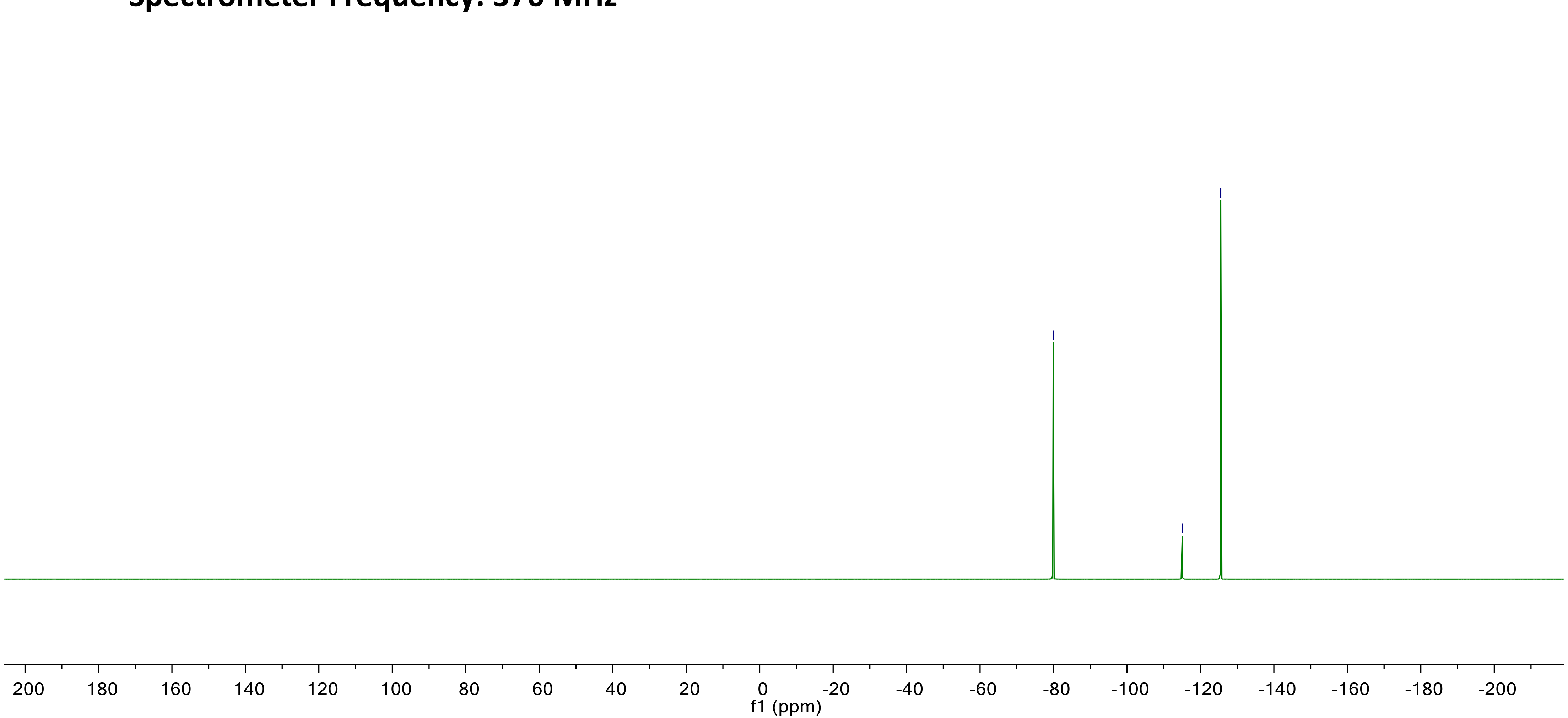

$^{13}C\ \{^1H\}$ NMR spectrum
Solvent: $CDCl_3$
Spectrometer Frequency: 100 MHz

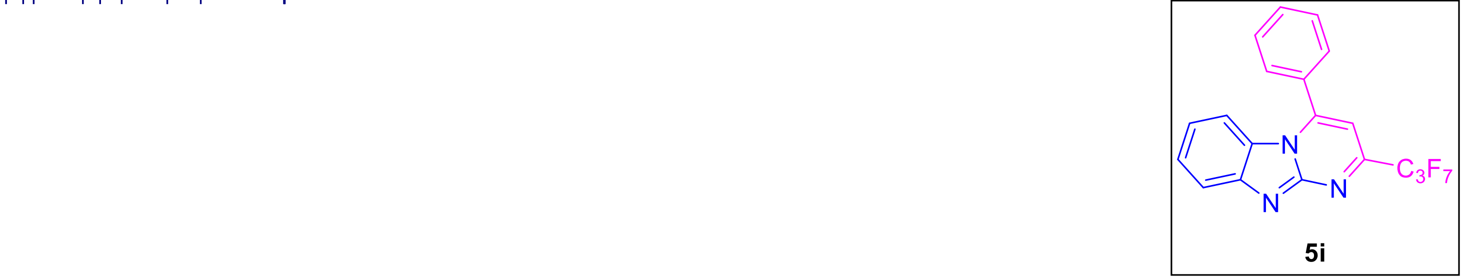

5i

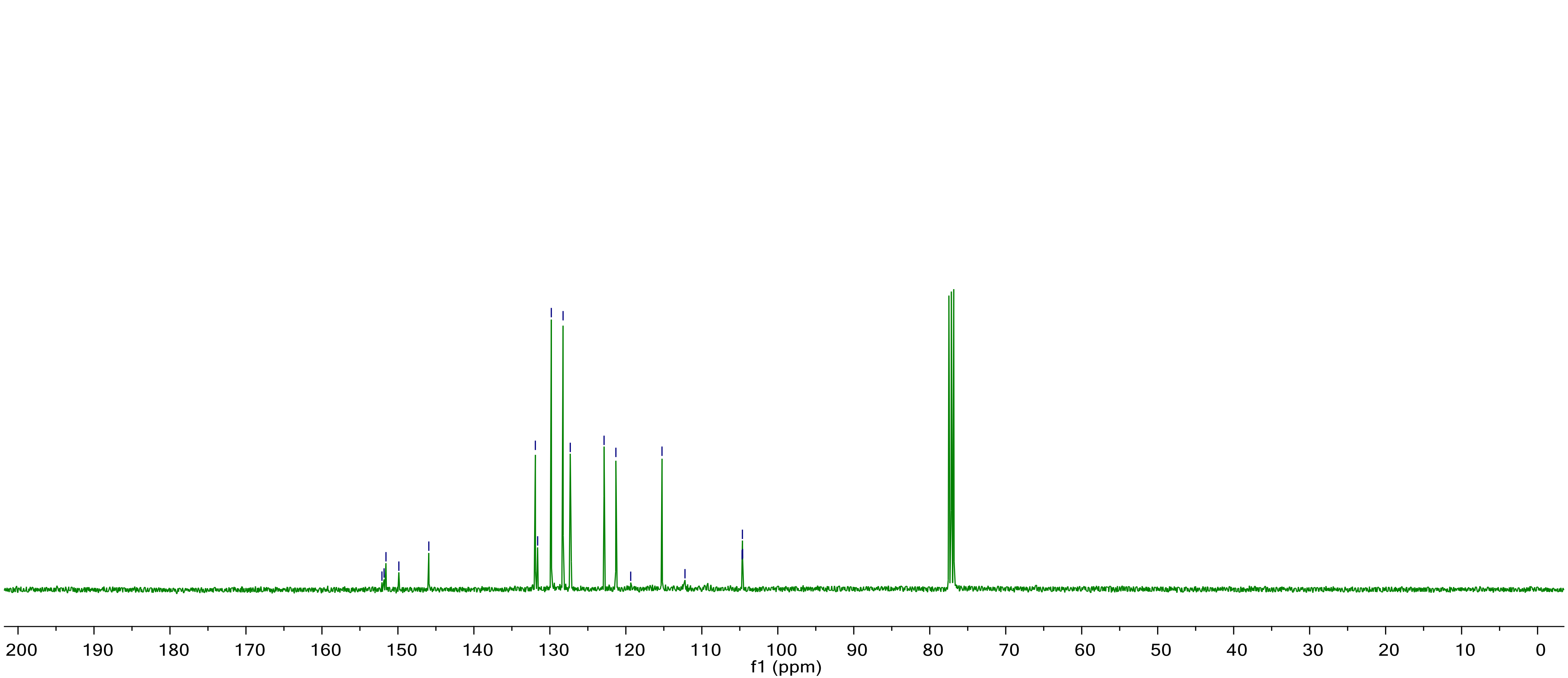

^{1}H NMR spectrum

Solvent: $CDCl_3$

Spectrometer Frequency: 400 MHz

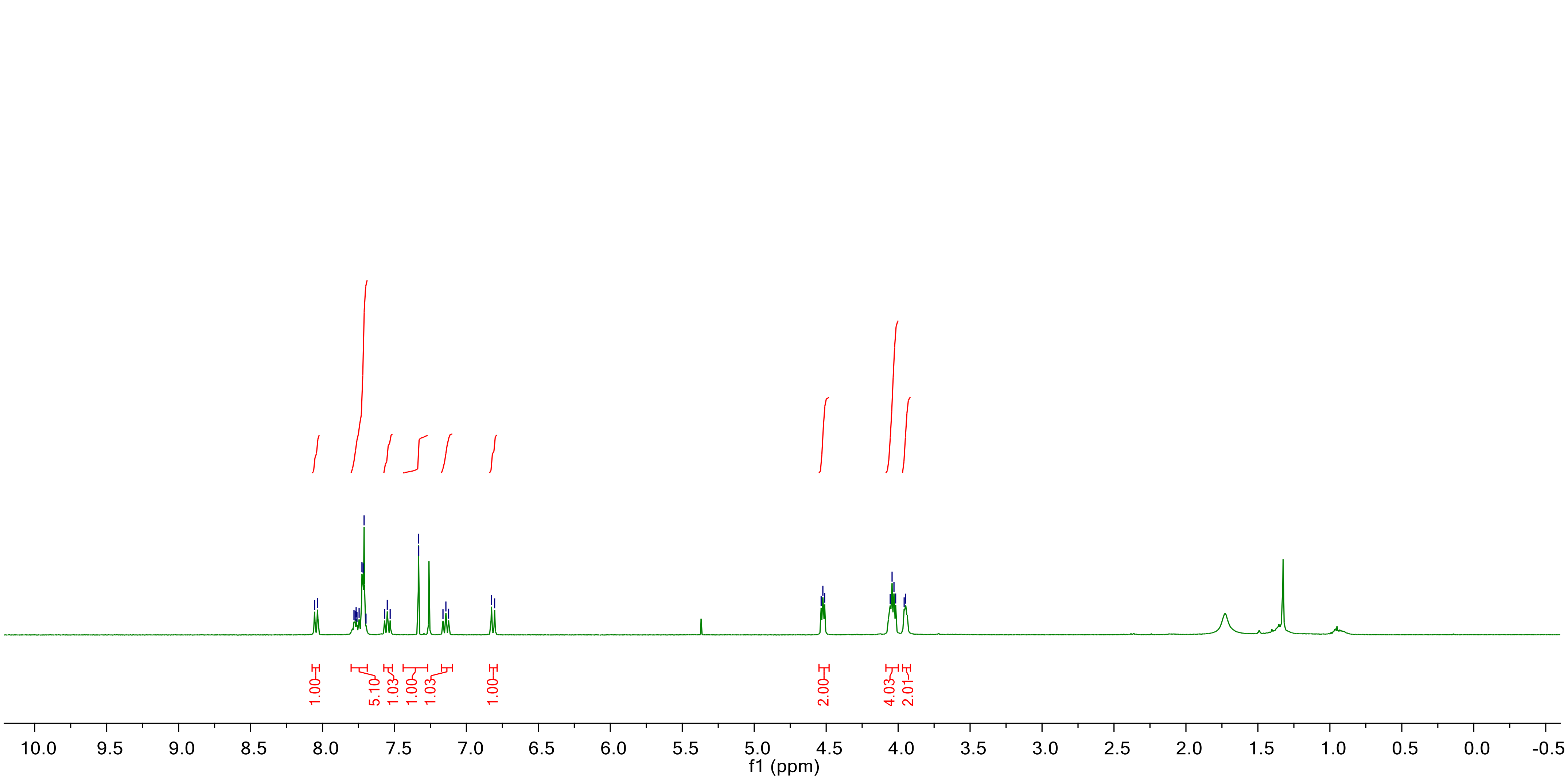

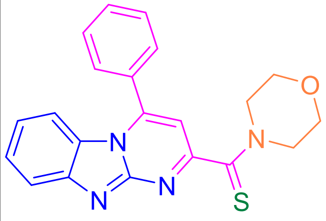

8

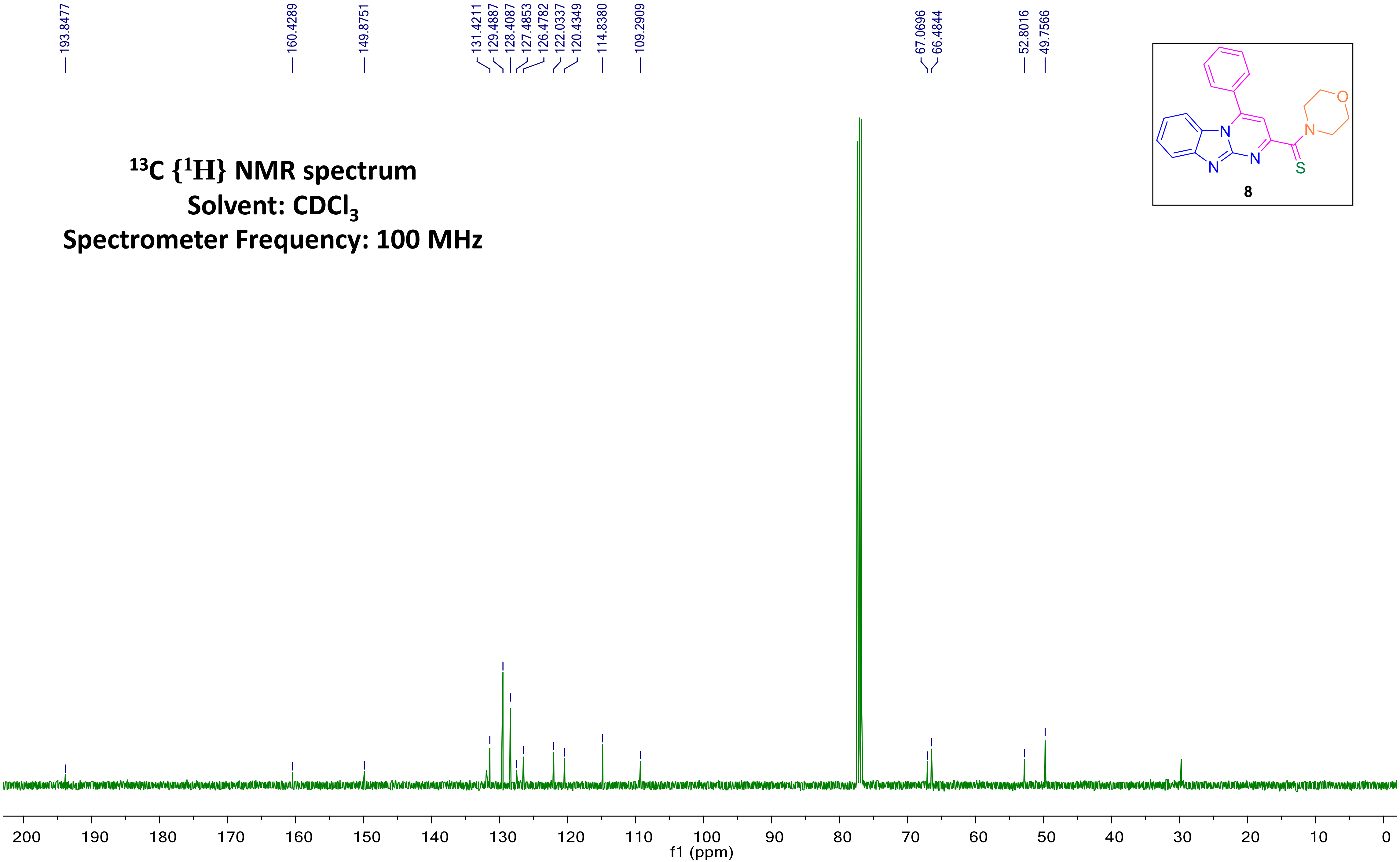
193.8477
160.4289
149.8751
131.4211
129.4887
128.4087
127.4853
126.4782
122.0337
120.4349
114.8380
109.2909
67.0696
66.4844
52.8016
49.7566
^{13}C {^{1}H} NMR spectrum
Solvent: $CDCl_3$
Spectrometer Frequency: 100 MHz
200 190 180 170 160 150 140 130 120 110 100 90 80 70 60 50 40 30 20 10 0
f1 (ppm)
N
N
N
N
O
S

8
